# Identification of Temporal Characteristic Networks of Peripheral Blood Changes in Alzheimer’s Disease Based on Weighted Gene Co-expression Network Analysis

**DOI:** 10.3389/fnagi.2019.00083

**Published:** 2019-05-21

**Authors:** Runhong Tang, Huayan Liu

**Affiliations:** Department of Neurology, First Affiliated Hospital, China Medical University, Shenyang, China

**Keywords:** Alzheimer’s disease, mild cognitive impairment, WGCNA, time serial expression analysis, peripheral blood, receiver operating characteristic curve

## Abstract

Alzheimer’s disease (AD) is a progressive neurodegenerative disease. The study of blood-based biomarkers has lasted for a long time in AD, because it supports the concept that peripheral changes are involved in AD pathology. But it is still unclear how peripheral blood is involved in the temporal characteristic molecular mechanisms of AD from aging to mild cognitive impairment (MCI) and which cells are responsible for the molecular mechanisms. The main purpose of our study is to gain a systematic and comprehensive understanding of temporal characteristic networks of peripheral blood in AD using whole blood samples with 329 case-control samples, including 104 normal elderly control subjects (CTL), 80 MCI who are susceptible to AD, and 145 AD, by the weighted gene co-expression network analysis (WGCNA). The module-trait relationships were constructed and module preservation was validated with independent datasets GSE63061, GSE97760, GSE18309, GSE29378, GSE28146, and GSE29652. Our results indicate that the down-regulated protein modification and ubiquitin degradation systems, and the up-regulated insulin resistance both play a major role in MCI, while the up-regulated inflammatory cascade dominates in AD, which is mainly mediated by monocytes, macrophages. Although there is mixed activation of M1 and M2 macrophages in all stages of AD, the immune neutral state or M2 polarization may predominate in MCI, and M1 polarization may predominate in AD. Moreover, we found that TRPV2, NDUFV1, ATF4, HSPA8, STAT3 and LUC7L3 may mediate the pathological changes in MCI, while SIRPA, LAMP-2, NDUFB5, HSPA8, STAT3 and FPR2 may mediate the conversion from MCI-AD or the pathological changes in AD, which provide a basis for the treatment based on the peripheral blood system. In addition, we also found that the combined diagnosis based on a panel of genes from the red, blue, and brown modules have a moderate diagnostic effect on distinguishing MCI and AD from CTL, suggesting that those panels of genes may be used for detection of MCI and prediction of this conversion from MCI to AD. Our research emphasizes that pathological changes, based on temporal characteristics of peripheral blood, provide a theoretical basis for targeted peripheral treatment based on appropriate times and identified several diagnostic markers.

## Introduction

Alzheimer’s disease (AD), the most common type of dementia, affects more than 50 million people worldwide (Hodson, 2018). More importantly, the prevalence of AD is expected to triple in the next 40 years, which will place a heavy burden on society and the healthcare system (Hebert et al., 2013). The amyloid cascade hypothesis still dominates the etiology of AD, which reveals the temporal characteristics of AD brain pathological changes: that β-amyloid (Aβ) deposition occurs early, followed by changes in brain function and metabolism, and alterations in biomarkers of neurodegeneration, such as tau-mediated neuronal injury and structural changes, eventually lead to cognitive impairment (Mattsson et al., 2009; Jack et al., 2010; García-Ribas et al., 2014). Clinically, AD is divided into three different stages: asymptomatic or pre-clinical stage, mild cognitive impairment (MCI), and AD stage (Dubois et al., 2010; Gold and El Khoury, 2015). It is universally accepted that AD has a long preclinical or asymptomatic stage, which starts approximately 15–20 years before the onset of clinical symptoms (Sperling et al., 2011). Meanwhile, the failures of all phase III clinical trials targeting protein-modified drugs for AD may suggest the fact that the enrolled participants are really too advanced to obtain clinical benefit (Egan et al., 2018; Honig et al., 2018). Thus, early diagnosis and treatment are important for delaying the progression of AD.

Although accurate diagnosis has been achieved by novel CSF biomarkers (Clark et al., 2003) or positron emission tomography Aβ trace (Schoonenboom et al., 2008), which are both too invasive or expensive to be used in large-scale clinical screening. Thus, peripheral blood is prioritized to identify reliable biomarkers for early diagnosis of AD. In fact, the study of blood-based biomarkers has been going on for a long time in AD, because it supports the concept of peripheral blood involvement in AD pathology, even prior to the occurrence of recognizable symptoms (Gladkevich et al., 2004; Rye et al., 2011). AD patients present mitochondrial dysfunction (Lombardi et al., 1999; Lunnon et al., 2012) in both the central nervous system (CNS) and periphery systems. In addition, the expression of some genes from the peripheral blood may be correlated with the burden of Aβ in brain (Avagyan et al., 2009). However, molecular mechanisms involved in temporal characteristics of AD and diagnostic markers based on the peripheral blood have not been extensively investigated. Therefore, the peripheral blood may provide an ideal “window” for the CNS to identify some genes involved in the pathogenesis, diagnosis, and progression of AD.

Weighted gene co-expression network analysis (WGCNA), a bioinformatics analysis method, has been proven to effectively detect the complex module-trait relationships (Langfelder and Horvath, 2008). The distinct advantage for WGCNA is that it can cluster genes into a model or network according to weight correlation coefficient between genes, and then analyses the correlation between modules and sample characteristics (including clinical features, surgical methods, treatment methods, etc.). WGCNA built a bridge between sample characteristics and changes in gene expression, providing insights into a systematic signaling network that may be associated with interested phenotype (Liao et al., 2017; Ma et al., 2017).

This study aimed to gain comprehensively molecular insights into the peripheral whole blood involved in temporal characteristics of AD, to identify responsible cells involved in these molecular mechanisms, and to find key genes involved in diagnosis and intervention of AD at an early stage. We constructed module-trait relationships by WGCNA using 329 samples including 104 control subjects (CTL), 80 MCI who are susceptible to AD, and 145 AD. Then, we identified seven modules associated with different stages of AD and module preservation for each of the seven modules was validated with independent datasets. Next, we tested the overlap between the module and cell signature gene lists using GeneOverlap and performed functional enrichment analysis. Finally, we obtained the hub genes of modules using cytohubba plugin in Cytoscape and performed receiver operating characteristic curve analysis to detect its diagnostic power.

## Materials and Methods

### Microarray Data Processing

The human whole blood mRNA expression dataset GSE63060 provided by AddNeuroMed Cohort, which is a large cross-European prospective biomarker study, was downloaded from the Gene Expression Omnibus (GEO) database (Barrett et al., 2013). Approximately 2.5 ml whole blood was collected in a PAXgene blood RNA vacutainer tube and held at −20°C for 24 h, then at −80°C until RNA extraction. In total, 329 case-control samples were included with 104 CTL, 80 MCI who are susceptible to AD, and 145 AD. Human whole blood mRNA were hybridized on Illumina HumanHT-12 V3.0 expression bead chip and expression profile data was generated using the limma package in R. The remaining 325 samples were included in the subsequent WGCNA after detecting the outliers. Probes with the first 25% coefficient of variation were used for WGCNA analysis.

### Weighted Gene Co-expression Network Analysis (WGCNA)

To provide a comprehensive analysis of the human whole blood for AD, we constructed a weighted gene co-expression network using the WGCNA package in R. Details are as follows. First, the raw file was converted into expression profile data by the limma package, then the probes with the first 25% coefficient of variation and the included individuals were selected, in which the latter was decided using a sample cluster tree to remove the outliers. Second, soft threshold was obtained based on the connections between genes obeying the scale-free networks. Third, the correlation matrix and the adjacency matrix were constructed. Next, the hierarchical clustering tree was established based on similar expression patterns using the dynamic tree cut method. Finally, the module trait relationships were formed and the modules closely related to the traits were identified, then the corresponding module gene information was extracted for further analysis.

### Module Preservation Analysis

In order to validate the reliability of the identified modules associated with different stages of AD, we performed the module preservation analysis using peripheral whole blood or blood monocyte datasets, including GSE63061, GSE97760, and GSE18309, and hippocampal datasets, including GSE29378, GSE28146, as well as astrocytes enriched samples GSE29652. The details of the above datasets are shown in Table 1. Included probes with the first 25% coefficient of variation in dataset GSE63060 were used as an input to assess the extent of module preservation in each dataset. The extent of module preservation was quantified by a Zsummary value provided by Langfelder et al. (2011), in which Zsummary < 2 indicates without preservation, 2 < Zsummary < 10 indicates weak-moderate preservation, and Zsummary > 10 indicates high preservation.

**Table 1:** Baseline characteristics of datasets.

Study	GEO accession	Platform ID	Sample type	Cases/controls		
				Number	Age (±SD)	Gender (F/M)
Timmons	GSE63060	GPL6947	Whole blood	329	74.2 (6.5)	200/129
Timmons	GSE63061	GPL10558	Whole blood	388	77.2 (6.8)	135/253
Fu	GSE97760	GPL16699	Whole blood	19	75.7 (12.7)	19/0
Chen	GSE18309	GPL570	Peripheral blood mononuclear cells	9	-	-
Miller	GSE29378	GPL6947	Hippocampus	63	79.2 (8.3)	25/38
Blalock	GSE28146	GPL570	Hippocampus	30	86.3 (7.7)	18/12
Heath	GSE29652	GPL570	Human (Astrocyte)	18	-	-

### Cell Signature Modules and Functional Enrichment Analysis

Cell signature gene lists were downloaded from the literature of Aran et al. (2017) and Wang et al. (2017), and were used to identify the modules specific to cell signature by GeneOverlap (Zougman et al., 2011) in R. The overlap between modules related to different stages of AD and cell signature gene lists was identified by Fisher’s exact test and *p*-value < 0.05 was used to determine cell signature modules with statistical significance, in which a *P* value closer to zero means the overlap is more important, otherwise it means no correlation. Then, all the above modules were used to perform functional enrichment analysis using the Database for Annotation, Visualization and Integrated Discovery (DAVID). GO terms and KEGG pathways (Ashburner et al., 2000) were selected with *P*-Value < 0.05 and the top records were extracted for further analysis.

### Identification of Hub Genes and Receiver Operating Characteristic Curve Analysis (ROC)

Hub genes with the largest intra-module connectivity were identified and visualized by Cytoscape, in which those hub genes were ordered by the degree of intra-module connectivity. The top 15 hub genes from each module were used to perform the receiver operating characteristic curve analysis using IBM SPSS statistics 22 in datasets GSE63060 and GSE63061, in which the former is the training dataset and the latter is the validation dataset.

## Results

### Modules Associated Different Stages of AD

**Figure 1:**
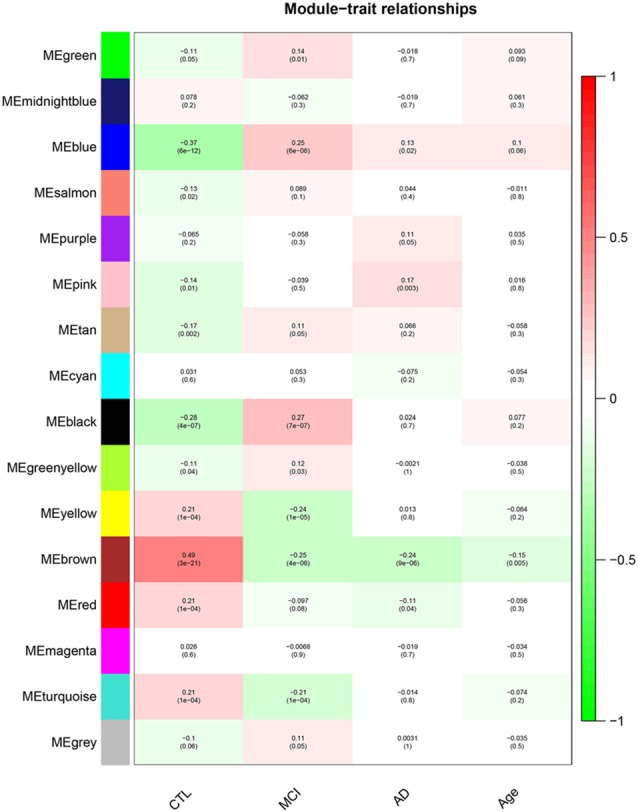
Module–Trait relationships. Correlation between module eigengene (ME) expression levels and control subjects (CTL), mild cognitive impairment (MCI), Alzheimer’s disease (AD) and Age in each module. Pearson correlation is reported with the *p*-value given inside the bracket.

**Figure 2:**
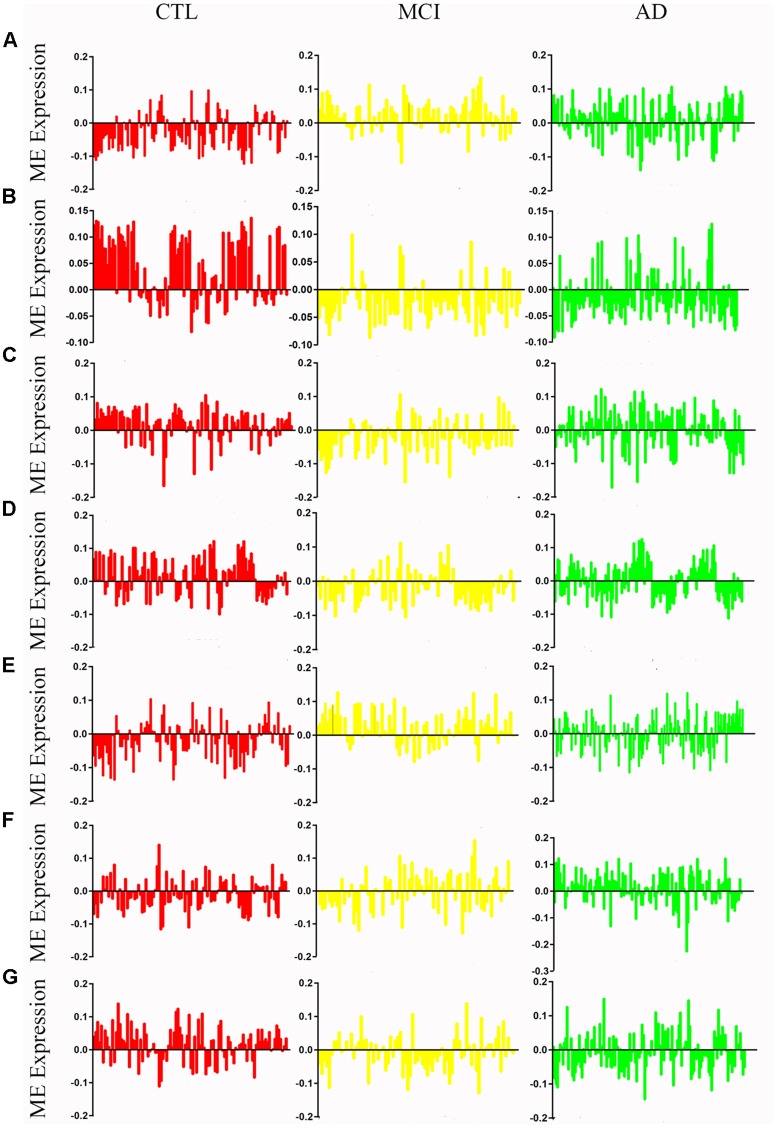
Mean eigengene (ME) expression values across different stages. The samples are grouped in to Control (CTL, red), mild congnitive impairment (MCI, yellow) and Alzheimer’s disease (AD, green). **(A)** Blue, **(B)** brown, **(C)** yellow, **(D)** turquoise, **(E)** black, **(F)** pink, **(G)** red.

After removing outliers, a total of 325 samples, including 104 CTL, 80 MCI who susceptible to AD, and 141 AD, were used to perform the co-expression network using 7,764 genes by WGCNA package in R. First, we have chosen the power 7 as the soft-threshold, in which the connections between the genes in the network were close to the scale-free network where scale-free topology fit index was up to 0.9 and the average connectivity degree was near to 0 (Supplementary Figure S1). We further validated whether the network constructed using our selected power value follows the scale-free network (Supplementary Figure S2). Second, the co-expression modules were constructed and 16 distinct modules were identified (Supplementary Figure S3). Then, in order to identify modules associated with different stages of AD, we constructed the module-trait relationships (Figure 1). There were multiple modules related to different stages of AD. For example, the blue and brown modules had similar changes in MCI and AD compared with CTL. The black, yellow, and turquoise modules were significantly related to the MCI, which may participate in the pathogenesis of early stage AD. The pink and red modules were specifically related to the advanced stage of AD which may be involved in the progression and transition of MCI to AD. The ME expression level (Figure 2) of each module associated was with different stages of AD, indicating obvious inter-stage differences in the ME expression level. In addition, we plotted a scatterplot of GS vs. MM for above modules, respectively, to again validate the correlation between module and different stages of AD (Supplementary Figure S4).

### Module Preservation Analysis

**Figure 3:**
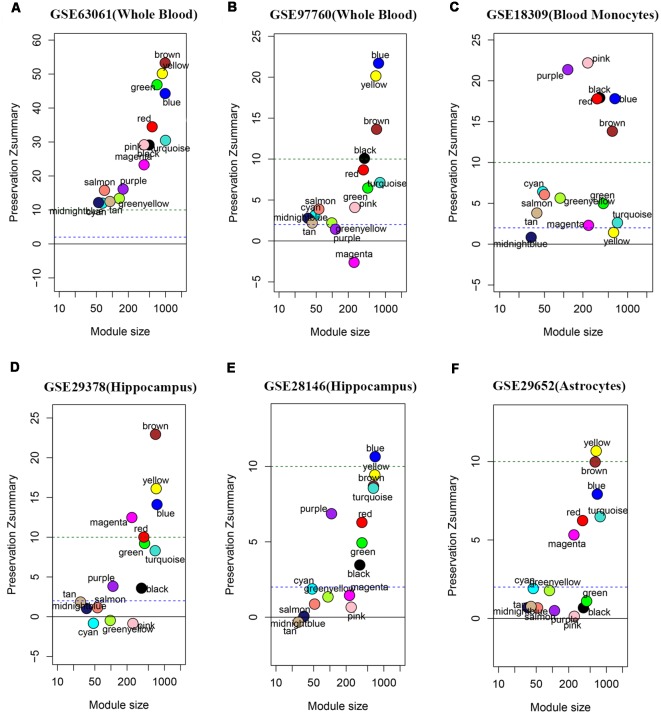
Module persevation analysis. The module preservation analysis was performed using peripheral whole or blood or blood monocytes datasets including **(A)** GSE63061, **(B)** GSE97760, and **(C)** GSE18309 and hippocampal datasets including **(D)** GES29378 and **(E)** GSE28146 as well as astrocytes enriched samples **(F)** GSE29652. Zsummary < 2 represents without preservation, 2 < Zsummary < 10 represents weak-moderate preservation, Zsummary > 10 represents high preservation.

We performed the module preservation analysis using peripheral whole blood or blood monocyte datasets, including GSE63061, GSE97760, and GSE18309, and hippocampal datasets, including GSE29378 and GSE28146, as well as astrocytes enriched samples GSE29652. Our results suggest that modules associated with different stages of AD show moderate-high preservation in datasets of peripheral whole blood or blood mononuclear cells and brain (Figure 3). Details are as follows. Our results from datasets of peripheral whole blood and hippocampus show that most of the modules associated different stages of AD present mediate-high preservation, suggesting peripheral blood might reflect the pathology of AD. Our result from a dataset of blood monocytes indicates that the blue, brown, black, pink, red modules show high preservation, while yellow and turquoise show weak-preservation.

### Cell Signature Modules and Functional Enrichment Analysis

**Figure 4:**
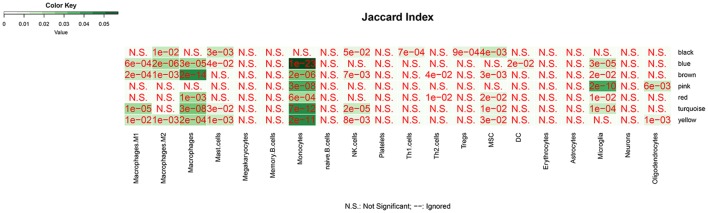
The overlap between cell-signature genes and module associated with dfferent stages of AD. The *P* value is closer to zero that means the more important of the overlap.

We analyzed the overlap of modules associated with different stages of AD and cell signature genes (Figure 4). The yellow, turquoise, and black modules are mostly linked to monocytes, macrophages, Th1.cells, and microglia. Functional enrichment analysis of those modules shows that the GO terms were mainly enriched in the cellular metabolic and catabolic process, protein modification and protein ubiquitination process, RNA processing, ribonucleoprotein complex biogenesis, cellular response, and adhesion; and that the KEGG pathways were mainly focused on metabolic pathways, NF-kappa B signaling pathway and different neurodegenerative diseases. The blue and brown modules are also mainly correlated with monocytes and macrophages. Functional enrichment analysis shows that the GO terms were enriched in protein phosphorylation, modification and transport, cellular response to lipopolysaccharide and positive regulation of interleukin-4 production, translation, antigen processing, and presentation of exogenous peptide antigen *via* MHC class I, and TAP-dependent and ATP synthesis coupled proton transport. Functional enrichment analysis also shows that the KEGG pathways were focused on pentose phosphate pathway, endocytosis and insulin resistance, oxidative phosphorylation and neurodegenerative diseases. The Pink and red module are mainly linked to microglia and macrophages. Finally, functional enrichment analysis shows that the GO terms were mainly enriched in translation process, SRP-dependent co-translational protein targeting to membrane, negative regulation of NF-kappa B transcription factor activity and apoptotic process, and inflammatory responses such as inflammatory cytokine secretion and inflammatory associated signal cascade; and that KEGG contained Toll-like receptor signaling pathway, Fc gamma R-mediated phagocytosis, TNF signaling pathway, NOD-like receptor signaling and MAPK signaling pathway, leukocyte transendothelial migration, phagosome, HIF-1 signaling pathway and different neurodegenerative diseases. It is worth noting that yellow, turquoise, blue, and black modules are simultaneously related to M2 macrophages and M1 macrophages or Th1 cells. There was no correlation between each module and erythrocytes, platelets, B cells, astrocytes, or neurons. All of the above GO terms and KEGG pathways of different modules were depicted in Table 2.

**Table 2:** Enrichment of Gene Ontology (GO) terms and KEGG pathways associated with mild cognitive impairment (MCI) and Alzheimer’s disease (AD)-specific modules.

Module (genes)	Biological process	KEGG pathway
Black (492)	RNA processing (1.7E-8), ribonucleoprotein complex biogenesis (8.8E-8), regulation of cell cycle (4.8E-7)	RNA transport (1.3E-5)
Blue (998)	leukocyte activation (2.0E-28), vesicle-mediated transport (4.6E-28), leukocyte activation involved in immune response (1.2E-27)	Lysosome (2.5E-8), Autophagy (4.2E-7), Endocytosis (1.6E-7), Pentose phosphate pathway (4.1E-7), Insulin resistance (6.2E-6)
Brown (987)	translation (3.1E-24), antigen processing and presentation of exogenous peptide antigen *via* MHC class I, TAP-dependent (2.4E-8) SRP dependent cotranslational protein targeting to membrane (1.9E-21)	Ribosome (7.6E-30), Oxidative phosphorylation (2.4E-21) Parkinson’s disease (3.3E-13), Alzheimer’s disease (7.9E-12)
Pink (404)	cytokine secretion (4.69E-5), positive regulation of oxidative stress-induced neuron death (5.2E-5), production of molecular mediator involved in inflammatory response (6.0E-5)	NOD-like receptor signaling pathway (4.3E-5), Fc gamma R-mediated phagocytosis (3.2E-4), Phagosome (8.0E-5), TNF signaling pathway (1.2E-4)
Red (565)	peptide biosynthetic process (3.4E-15), protein targeting to ER (3.6E-15), SRP-dependent cotranslational protein targeting to membrane (1.4E-5)	Ribosome (2.1E-16), Leukocyte transendothelial migration (1.2E-2), Phagosome (2.2E-2), Parkinsons disease (2.2E-2)
Turquoise (1140)	RNA processing (3.4E-19), mRNA metabolic process (1.3E-17), RNA splicing (1.3E-13), cellular response to glucose starvation (6.92E-05)	Metabolic pathways (2.1E-3), NF-kappa B signaling pathway (5.3E-3), Spliceosome (5.6E-3)
Yellow (874)	cellular catabolic process (2.4E-10), cellular catabolic process (6.6E-10), protein modification process (1.3E-8)	Ubiquitin mediated proteolysis (3.6E-5)

### Identification of Hub Genes and Receiver Operating Characteristic Curve Analysis

**Figure 5:**
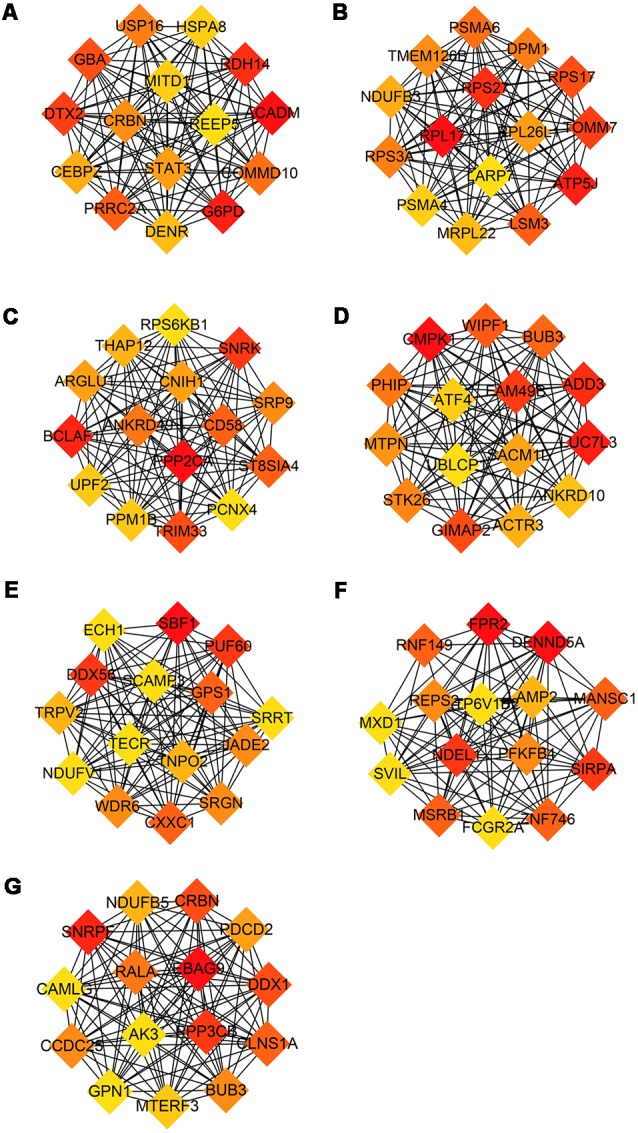
Hub genes from each module associated with different stage of AD. Hub genes with the largest intra-module connectivity was identified by cytohubba plugin in Cytoscape an ordered by the degree of intra-module connectivity. The gradient from orange to red represents the correlation from low to high. **(A)** Blue, **(B)** brown, **(C)** yellow, **(D)** turquoise, **(E)** black, **(F)** pink, **(G)** red.

The modules correlated with different stages of AD were visualized by Cytoscape and the hub genes calculated by cytohubba plugin in Cytoscape (Figure 5). All the above hub genes were used to perform ROC analysis to determine the diagnostic power for distinguishing AD and MCI from control. Our results show that the AUC of the combined diagnostic effect of all hub genes from each module is significantly higher than that of a single hub gene. The results of the former are shown in Table 3, and of the latter are shown in Supplementary Figure S1. To blue, brown, and pink modules, the AUC of combined diagnosis of all hub genes for differentiating AD and MCI from control were all more than 0.8 in the training dataset, while more than 0.7 in the validation dataset. However, with the exception of PRRC2A (0.749, 95%CI (0.679–0.820), the ROC of a single hub gene never reached 0.7. For turquoise, pink, and yellow modules, the AUC of combined diagnosis of all hub genes for differentiating AD and MCI from control were all more than 0.7 in the training dataset, while more than 0.6 in the validation dataset. However, the AUC of only WDR6 (0.735, 95%CI (0.663–0.806) was more than 0.7.

**Table 3:** The AUC of combined diagnosis of all hub genes from each module.

Module	GSE63060 AUC (95%CI)		GSE63061 AUC (95%CI)	
	AD-CTL	MCI-CTL	AD-CTL	MCI-CTL
black	0.813 (0.758, 0.868)	0.720 (0.649, 0.791)	0.729 (0.670, 0.788)	0.728 (0.665, 0.792)
blue	0.822 (0.769, 0.875)	0.834 (0.776, 0.892)	0.723 (0.663, 0.823)	0.730 (0.667, 0.794)
brown	0.875 (0.832, 0.918)	0.841 (0.785, 0.897)	0.717 (0.654, 0.780)	0.775 (0.717, 0.833)
pink	0.809 (0.747, 0.870)	0.772 (0.714, 0.831)	0.722 (0.663, 0.782)	0.638 (0.570, 0.707)
red	0.837 (0.786, 0.888)	0.814 (0.754, 0.874)	0.706 (0.645, 0.767)	0.727 (0.665, 0.790)
turquoise	0.689 (0.618, 0.760)	0.767 (0.698, 0.835)	0.677 (0.614, 0.740)	0.670 (0.602, 0.738)
yellow	0.793 (0.735, 0.853)	0.804 (0.741, 0.868)	0.692 (0.629, 0.754)	0.710 (0.645, 0.775)

## Discussion

Modules associated with MCI were the yellow, turquoise, and black modules, which are mainly enriched in monocytes, macrophages, Th1 cells, and microglia, mediating the down-regulation of metabolic and catabolic process, ubiquitin-mediated proteolysis, and NF-kappa B signaling pathway, as well as the up-regulation of RNA processing, ribonucleoprotein complex biogenesis, and RNA transport, et al. Specifically speaking, the yellow and turquoise modules with moderate-high preservation in validation datasets (except for monocyte dataset) suggest that RNA metabolism and inflammatory signaling pathway were down-regulated in both microglia and peripheral blood monocytes and macrophages, while ubiquitin-mediated proteolysis and protein modification process were down-regulated in peripheral blood monocytes and macrophages. The black module with high preservation in peripheral whole blood or monocyte datasets indicates that ribonucleoprotein complex biogenesis and RNA processing were up-regulated in regulatory T cells and Th1 cells. It is worth emphasizing that those modules are almost simultaneously involved in M1 and M2 macrophages or regulatory T cells and Th1 cells, which suggests that mixed activation of the peripheral blood immune system may exist in MCI. In these modules, we found that a part of the hub genes may be involved in the pathology, development and diagnosis of AD. For example, Enoyl-CoA hydratase 1 (ECH1), a member of the hydratase/isomerase superfamily, was proven to be a promising marker for early diagnosis of AD (Long et al., 2016). TRPV2, one of the receptors for cannabidiol, may be involved in neuroprotective and immunomodulatory effects (Noreen et al., 2018). NDUFV1 was validated to be involved in the pathogenesis of AD (Zhang et al., 2015). ATF4, a required element for the activity of PS1 promoting the production of Aβ, was proven to be a potential therapeutic target for AD (Wei et al., 2015). LUC7L3, the BAD domain of U1-70K interacting with Tau from AD brains, mediates co-aggregation with the pathological AD-specific Tau isoforms (Bishof et al., 2018). All of the above results suggest that the upregulation of protein synthesis level and downregulation of protein modification may promote protein aggregation. Meanwhile, the down-regulation of ubiquitination process exacerbates the accumulation of protein aggregation. These two processes result in an imbalance in production and clearance of protein aggregation, synergistically promoting the deposition of protein aggregation. It is well known that the neurodegenerative diseases are characterized by the accumulation of protein aggregates in distinct brain areas (Gerakis and Hetz, 2018) and are now defined as protein-misfolded disorders (PMDs) (Beal, 2002; Ren et al., 2014; Boland et al., 2018). The misfolded proteins can be refolded to restore the protein’s normal conformation by protein modification. Alternatively, if they cannot be refolded, they will be transported to the intracellular degradation system that includes the ubiquitin proteasomal system (UPS), autophagy-lysosomal pathway (ALP), and the interaction of molecular chaperones with UPS or ALP (Tramutola et al., 2016; Zhang et al., 2017) for degradation. Any disturbance to these systems causes proteins to accumulate, resulting in the pathological process of AD. Once the protein modification and ubiquitination degradation process malfunctions, uncontrolled abnormal deposition of protein occur.

Modules associated with AD were the red and pink modules, which are mainly enriched in microglia, monocytes, and macrophages, mediating the down-regulation of the ribosome, peptide biosynthetic process, and leukocyte transendothelial migration, as well as the up-regulation of cytokine secretion, NOD-like receptor signaling pathway, Fc gamma R-mediated phagocytosis, TNF signaling pathway, and positive regulation of oxidative stress-induced neuron death. Specifically speaking, the red module with moderate-high preservation in validation datasets suggests that ribosome, peptide biosynthetic process, and leukocyte transendothelial migration were down-regulated in both microglia and peripheral blood monocytes and macrophages. The pink module has moderate-high preservation in peripheral whole blood or mononcyte datasets but weak preservation in hippocampus datasets, suggesting that the inflammatory pathway and oxidative stress-induced neuron death were up-regulated in both microglia and peripheral blood monocytes and macrophages. In these modules, we also found that some hub genes may play an important role in the progression of the disease. For instance, SIRPA, a receptor for macrophage CD47, regulates phenotypic polarization of macrophages by tumor necrosis factor (Ye et al., 2016; Alvey et al., 2017). LAMP-2 regulates Aβ degradation (Ma et al., 2017) and can serve as a potential AD-specific marker in cerebrospinal fluid (Armstrong et al., 2014). NDUFB5 genes may predict the occurrence and development of AD (Wang et al., 2017). FPR2 regulates microglia/macrophages polarization in response to different inflammatory stimuli (Slowik et al., 2012; Yu and Ye, 2015). All of the above results may reflect the phenomena that inflammatory activation of microglia and monocytes/macrophages predominates in the AD stage The existence of age-dependent phenotypic change of microglial activation has been demonstrated in an animal model, switching from an alternative activation state with a phagocytic capability to a classically activated phenotype with pro-inflammatory cytokine production (Jimenez et al., 2008). This switch coincided with high levels of soluble Aβ oligomers and a significant pyramidal neurodegeneration (Goldeck et al., 2016). Meanwhile, this switch was validated in subjects with MCI or AD at baseline and follow-up was completed using 11C-(R) PK1119529 (Fan et al., 2017). The M2-to-M1 phenotypic switches for the peripheral immune cells has also been reported to be involved in the development and progression of some CNS diseases (Kigerl et al., 2009; Perego et al., 2011; Hu et al., 2012). M2 macrophage transplantation in patients with AD alleviates brain inflammation and improves cognitive dysfunction (Zhu et al., 2016).

Modules associated with all stages of AD were the blue and brown modules, which are mainly enriched in monocytes and macrophages, mediating the down-regulation of translation, antigen processing and presentation of exogenous peptide antigen *via* MHC class I, TAP-dependent, oxidative phosphorylation, and ribosome, as well as the up-regulation of leukocyte activation, vesicle-mediated transport, insulin resistance, lysosome, autophagy and endocytosis, etc. It is worth noting that the ME expression level of blue was up-regulated mainly in the MCI stage. Combined with cell signature modules and functional enrichment analysis, the results indicate that, although there is a mixed activation of M1 and M2 phenotype at each stage, the former may predominate in AD stage, while the latter predominates in the MCI stage. HSPA8 as a molecular chaperone, within the constitutively expressed heat shock protein 70 family, that binds to the nascent polypeptide to promote proper folding of misfolded proteins and to promote degrading by chaperone-mediated autophagy (Silva et al., 2014; Catarino et al., 2017). Stat3 mediates the polarization of macrophages to exert anti-inflammatory and neuroprotective effects (Bai et al., 2018). All of the above results indicate that the insulin resistance occurs in the whole stages of AD and compensatory phagocytosis plays a major role in the MCI stage. There is increasing evidence that insulin resistance has been identified as the pathology of AD at the early stage (Liu et al., 2011; Talbot et al., 2012). Insulin resistance increases key enzymes that produce Aβ and phosphorylate tau, such as BACE1, γ-secretase and GSK3 β (Son et al., 2012; Vandal et al., 2014), while it reduces levels of key enzymes that degrade Aβ, such as IDE (Newsholme et al., 2009), accompanied by oxidative stress (Kulstad et al., 2006), which damages synaptic remodeling and ultimately leads to memory impairment (Searcy et al., 2012). Meanwhile, the increase of Aβ and inflammatory factors (Takeda et al., 2010; Choudhary et al., 2011), in turn, promotes insulin resistance. This is a vicious circle in the progression of AD. Thus, insulin resistance may act as a trigger for AD and repairing insulin resistance may help delay the progression of AD.

The AUC of combined diagnostic effect of all hub genes from each module is significantly higher than that of a single hub gene in both training and validated datasets. The AUC of all hub genes from the red, blue, and brown modules are all more than 0.8 in the training dataset, while more than 0.7 in the validated dataset, suggesting that those panels of genes may be used for detection of MCI and prediction of this conversion from MCI to AD. Based on the ROC of a single gene, only the ROC of PRRC2A and WDR6 in the training dataset exceeded 0.7, while the ROC of PRRC2A and WDR6 in the validation dataset only exceeded 0.6, suggesting that individual genes do not achieve sufficient diagnostic efficacy and cannot be used as the sole diagnostic criteria.

**Figure 6:**
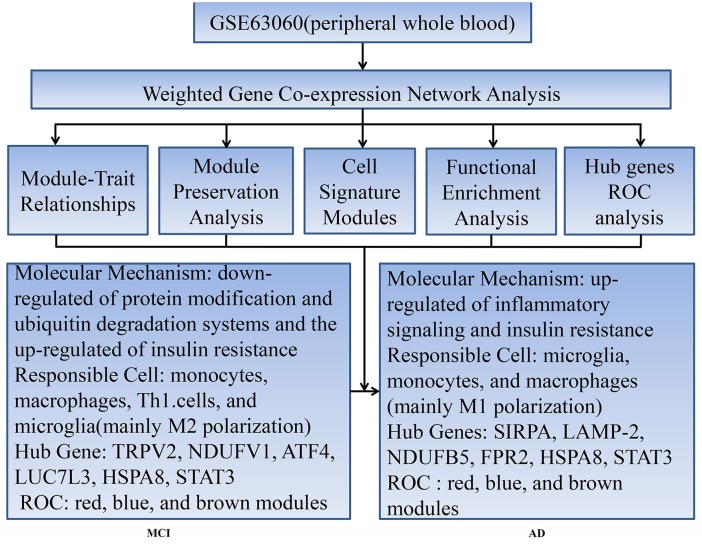
Workflow and main conclusions.

Our workflow and main conclusions are shown in Figure 6. In summary, first of all, our findings support the view that changes in peripheral blood are involved in temporal characteristic mechanisms of AD. Our results indicate that the down-regulated protein modification and ubiquitin degradation systems and the up-regulated insulin resistance all play a major role in MCI, while the up-regulated inflammatory cascade dominates in AD, which is mainly mediated by monocytes, macrophages, and microglia. Although there is mixed activation of M1 and M2 macrophages in all stages of AD, the immune neutral state or M2 polarization may predominate in MCI, and M1 polarization may predominate in AD. Moreover, we found that TRPV2, NDUFV1, ATF4, HSPA8, STAT3 and LUC7L3 may mediate the pathological changes in MCI, while SIRPA, LAMP-2, NDUFB5, HSPA8, STAT3 and FPR2 may mediate the conversion from MCI-AD or the pathological changes in AD, which provide a basis for the treatment based on the peripheral blood system. In addition, we also found that the combined diagnosis based on a panel of genes from the modules has a moderate diagnostic effect on distinguishing MCI and AD from CTL. In short, the pathological changes based on temporal characteristics of peripheral blood provide a theoretical basis for appropriate peripheral dose-based peripheral treatment at the appropriate time.

There are some limitations in our study: (1) cell type specific genes are based on reference datasets, and do not completely reflect cell type proportions. Changes in monocytes can occur in AD compared to controls, and this change within the same cell type will be lost or incorrectly interpreted using the currently used approach of assigning cell specificity to particular genes; and (2) since this is a cross-sectional study rather than a longitudinal study, the above results cannot fully represent the pathological changes from CTL-MCI-AD, and further experimental verification is needed.

## Author Contributions

HL and RT: study design. RT: literature search, data analysis and article writing. HL: article revision. Both authors approved the final version of the article.

## Conflict of Interest Statement

The authors declare that the research was conducted in the absence of any commercial or financial relationships that could be construed as a potential conflict of interest.

## References

[B101] Alvey C. M. Spinler K. R. Irianto J. Pfeifer C. R. Hayes B. Xia Y.. (2017). SIRPA-inhibited, marrow-derived macrophages engorge, accumulate, and differentiate in antibody-targeted regression of solid tumors. Curr. Biol. 27, 2065.e6–2077.e6. 10.1016/j.cub.2017.06.005 28669759 PMC5846676

[B1] Aran D. Hu Z. Butte A. J. (2017). xCell: digitally portraying the tissue cellular heterogeneity landscape. Genome Biol. 18:220. 10.1186/s13059-017-1349-1 29141660 PMC5688663

[B102] Armstrong A. Mattsson N. Appelqvist H. Janefjord C. Sandin L. Agholme L.. (2014). Lysosomal network proteins as potential novel CSF biomarkers for Alzheimer’s disease. Neuromolecular Med. 16, 150–160. 10.1007/s12017-013-8269-3 24101586 PMC3918123

[B2] Ashburner M. Ball C. A. Blake J. A. Botstein D. Butler H. Cherry J. M.. (2000). Gene ontology: tool for the unification of biology. The Gene Ontology Consortium. Nat. Genet. 25, 25–29. 10.1038/75556 10802651 PMC3037419

[B103] Avagyan H. Goldenson B. Tse E. Masoumi A. Porter V. Wiedau-Pazos M.. (2009). Immune blood biomarkers of Alzheimer disease patients. J. Neuroimmunol. 210, 67–72. 10.1016/j.jneuroim.2009.02.015 19329192

[B3] Bai H. Zhang Q. F. Duan J. J. Yu D. J. Liu L. J. (2018). Downregulation of signal transduction and STAT3 expression exacerbates oxidative stress mediated by NLRP3 inflammasome. Neural Regen. Res. 13, 2147–2155. 10.4103/1673-5374.241470 30323145 PMC6199955

[B5] Barrett T. Wilhite S. E. Ledoux P. Evangelista C. Kim I. F. Tomashevsky M.. (2013). NCBI GEO: archive for functional genomics data sets—update. Nucleic Acids Res. 41, D991–D995. 10.1093/nar/gks1193 23193258 PMC3531084

[B7] Beal M. F. (2002). Oxidatively modified proteins in aging and disease. Free Radic. Biol. Med. 32, 797–803. 10.1016/s0891-5849(02)00780-3 11978481

[B8] Bishof I. Dammer E. B. Duong D. M. Kundinger S. R. Gearing M. Lah J. J.. (2018). RNA-binding proteins with basic-acidic dipeptide (BAD) domains self-assemble and aggregate in Alzheimer’s disease. J. Biol. Chem. 293, 11047–11066. 10.1074/jbc.ra118.001747 29802200 PMC6052236

[B9] Boland B. Yu W. H. Corti O. Mollereau B. Henriques A. Bezard E.. (2018). Promoting the clearance of neurotoxic proteins in neurodegenerative disorders of ageing. Nat. Rev. Drug Discov. 17, 660–688. 10.1038/nrd.2018.109 30116051 PMC6456907

[B10] Catarino S. Pereira P. Girão H. (2017). Molecular control of chaperone-mediated autophagy. Essays Biochem. 61, 663–674. 10.1042/ebc20170057 29233876

[B11] Choudhary S. Sinha S. Zhao Y. Banerjee S. Sathyanarayana P. Shahani S.. (2011). NF-kappaB-inducing kinase (NIK) mediates skeletal muscle insulin resistance: blockade by adiponectin. Endocrinology 152, 3622–3627. 10.1210/en.2011-1343 21846802 PMC3176647

[B12] Clark C. M. Xie S. Chittams J. Ewbank D. Peskind E. Galasko D.. (2003). Cerebrospinal fluid tau and β-amyloid: how well do these biomarkers reflect autopsy-confirmed dementia diagnoses? Arch. Neurol. 60, 1696–1702. 10.1001/archneur.60.12.1696 14676043

[B104] Dubois B. Feldman H. H. Jacova C. Cummings J. L. Dekosky S. T. Barberger-Gateau P.. (2010). Revising the definition of Alzheimer’s disease: a new lexicon. Lancet Neurol. 9, 1118–1127. 10.1016/S1474-4422(10)70223-4 20934914

[B17] Egan M. F. Kost J. Tariot P. N. Aisen P. S. Cummings J. L. Vellas B.. (2018). Randomized trial of verubecestat for mild-to-moderate Alzheimer’s disease. N. Engl. J. Med. 378, 1691–1703. 10.1056/NEJMoa1706441 29719179 PMC6776074

[B18] Fan Z. Brooks D. J. Okello A. Edison P. (2017). An early and late peak in microglial activation in Alzheimer’s disease trajectory. Brain 140, 792–803. 10.1093/brain/aww349 28122877 PMC5837520

[B19] García-Ribas G. López-Sendón Moreno J. L. García-Caldentey J. (2014). Biomarkers in Alzheimer’s disease. Rev. Neurol. 58, 308–317. 10.33588/rn.5807.2013394 24677154

[B20] Gerakis Y. Hetz C. (2018). A decay of the adaptive capacity of the unfolded protein response exacerbates Alzheimer’s disease. Neurobiol. Aging 63, 162–164. 10.1016/j.neurobiolaging.2017.09.012 29042130

[B21] Gladkevich A. Kauffman H. F. Korf J. (2004). Lymphocytes as a neural probe: potential for studying psychiatric disorders. Prog. Neuropsychopharmacol. Biol. Psychiatry 28, 559–576. 10.1016/j.pnpbp.2004.01.009 15093964

[B105] Gold M. El Khoury J. (2015). β-amyloid, microglia, and the inflammasome in Alzheimer’s disease. Semin. Immunopathol. 37, 607–611. 10.1007/s00281-015-0518-0 26251237 PMC4618770

[B22] Goldeck D. Witkowski J. M. Fulop T. Pawelec G. (2016). Peripheral immune signatures in Alzheimer disease. Curr. Alzheimer Res. 13, 739–749. 10.2174/1567205013666160222112444 26899580

[B24] Hebert L. E. Weuve J. Scherr P. A. Evans D. A. (2013). Alzheimer disease in the United States (2010–2050) estimated using the 2010 census. Neurology 80, 1778–1783. 10.1212/WNL.0b013e31828726f5 23390181 PMC3719424

[B25] Hodson R. (2018). Alzheimer’s disease. Nature 559:S1. 10.1038/d41586-018-05717-6 30046078

[B26] Honig L. S. Vellas B. Woodward M. Boada M. Bullock R. Borrie M.. (2018). Trial of solanezumab for mild dementia due to Alzheimer’s disease. N. Engl. J. Med. 378, 321–330. 10.1056/NEJMoa1705971 29365294

[B27] Hu X. Li P. Guo Y. Wang H. Leak R. K. Chen S.. (2012). Microglia/macrophage polarization dynamics reveal novel mechanism of injury expansion after focal cerebral ischemia. Stroke 43, 3063–3070. 10.1161/strokeaha.112.659656 22933588

[B28] Jack C. R. Jr. Knopman D. S. Jagust W. J. Shaw L. M. Aisen P. S. Weiner M. W.. (2010). Hypothetical model of dynamic biomarkers of the Alzheimer’s pathological cascade. Lancet Neurol. 9, 119–128. 10.1016/s1474-4422(09)70299-6 20083042 PMC2819840

[B29] Jimenez S. Baglietto-Vargas D. Caballero C. Moreno-Gonzalez I. Torres M. Sanchez-Varo R.. (2008). Inflammatory response in the hippocampus of PS1M146L/APP751SL mouse model of Alzheimer’s disease: age-dependent switch in the microglial phenotype from alternative to classic. J. Neurosci. 28, 11650–11661. 10.1523/JNEUROSCI.3024-08.2008 18987201 PMC6671312

[B30] Kigerl K. A. Gensel J. C. Ankeny D. P. Alexander J. K. Donnelly D. J. Popovich P. G. (2009). Identification of two distinct macrophage subsets with divergent effects causing either neurotoxicity or regeneration in the injured mouse spinal cord. J. Neurosci. 29, 13435–13444. 10.1523/JNEUROSCI.3257-09.2009 19864556 PMC2788152

[B31] Kulstad J. J. Green P. S. Cook D. G. Watson G. S. Reger M. A. Baker L. D.. (2006). Differential modulation of plasma β-amyloid by insulin in patients with Alzheimer disease. Neurology 66, 1506–1510. 10.1212/01.wnl.0000216274.58185.09 16717209

[B32] Langfelder P. Horvath S. (2008). WGCNA: an R package for weighted correlation network analysis. BMC Bioinformatics 9:559. 10.1186/1471-2105-9-559 19114008 PMC2631488

[B33] Langfelder P. Luo R. Oldham M. C. Horvath S. (2011). Is my network module preserved and reproducible? PLoS Comput. Biol. 7:e1001057. 10.1371/journal.pcbi.1001057 21283776 PMC3024255

[B34] Liao X. Huang K. Huang R. Liu X. Han C. Yu L.. (2017). Genome-scale analysis to identify prognostic markers in patients with early-stage pancreatic ductal adenocarcinoma after pancreaticoduodenectomy. Onco. Targets. Ther. 10, 4493–4506. 10.2147/ott.s142557 28979141 PMC5602474

[B35] Liu Y. Liu F. Grundke-Iqbal I. Iqbal K. Gong C. X. (2011). Deficient brain insulin signalling pathway in Alzheimer’s disease and diabetes. J. Pathol. 225, 54–62. 10.1002/path.2912 21598254 PMC4484598

[B106] Lombardi V. R. García M. Rey L. Cacabelos R. (1999). Characterization of cytokine production, screening of lymphocyte subset patterns and *in vitro* apoptosis in healthy and Alzheimer’s disease (AD) individuals. J. Neuroimmunol. 97, 163–171. 10.1016/s0165-5728(99)00046-6 10408971

[B36] Long J. Pan G. Ifeachor E. Belshaw R. Li X. (2016). Discovery of novel biomarkers for Alzheimer’s disease from blood. Dis. Markers 2016:4250480. 10.1155/2016/4250480 27418712 PMC4932164

[B107] Lunnon K. Ibrahim Z. Proitsi P. Lourdusamy A. Newhouse S. Sattlecker M.. (2012). Mitochondrial dysfunction and immune activation are detectable in early Alzheimer’s disease blood. J. Alzheimers Dis. 30, 685–710. 10.3233/jad-2012-111592 22466004

[B37] Ma C. Lv Q. Teng S. Yu Y. Niu K. Yi C. (2017). Identifying key genes in rheumatoid arthritis by weighted gene co-expression network analysis. Int. J. Rheum. Dis. 20, 971–979. 10.1111/1756-185X.13063 28440025

[B38] Mattsson N. Zetterberg H. Hansson O. Andreasen N. Parnetti L. Jonsson M.. (2009). CSF biomarkers and incipient Alzheimer disease in patients with mild cognitive impairment. JAMA 302, 385–393. 10.1001/jama.2009.1064 19622817

[B39] Newsholme P. Morgan D. Rebelato E. Oliveira-Emilio H. C. Procopio J. Curi R.. (2009). Insights into the critical role of NADPH oxidase(s) in the normal and dysregulated pancreatic β cell. Diabetologia 52, 2489–2498. 10.1007/s00125-009-1536-z 19809798

[B40] Noreen N. Muhammad F. Akhtar B. Azam F. Anwar M. I. (2018). Is cannabidiol a promising substance for new drug development? A review of its potential therapeutic applications. Crit. Rev. Eukaryot. Gene Expr. 28, 73–86. 10.1615/critreveukaryotgeneexpr.2018021528 29773016

[B42] Perego C. Fumagalli S. De Simoni M. G. (2011). Temporal pattern of expression and colocalization of microglia/macrophage phenotype markers following brain ischemic injury in mice. J. Neuroinflammation 8:174. 10.1186/1742-2094-8-174 22152337 PMC3251548

[B43] Ren R. J. Dammer E. B. Wang G. Seyfried N. T. Levey A. I. (2014). Proteomics of protein post-translational modifications implicated in neurodegeneration. Transl. Neurodegener. 3:23. 10.1186/2047-9158-3-23 25671099 PMC4323146

[B44] Rye P. D. Booij B. B. Grave G. Lindahl T. Kristiansen L. Andersen H. M.. (2011). A novel blood test for the early detection of Alzheimer’s disease. J. Alzheimers Dis. 23, 121–129. 10.3233/JAD-2010-101521 20930265

[B46] Schoonenboom N. S. van der Flier W. M. Blankenstein M. A. Bouwman F. H. Van Kamp G. J. Barkhof F.. (2008). CSF and MRI markers independently contribute to the diagnosis of Alzheimer’s disease. Neurobiol. Aging 29, 669–675. 10.1016/j.neurobiolaging.2006.11.018 17208336

[B47] Searcy J. L. Phelps J. T. Pancani T. Kadish I. Popovic J. Anderson K. L.. (2012). Long-term pioglitazone treatment improves learning and attenuates pathological markers in a mouse model of Alzheimer’s disease. J. Alzheimers Dis. 30, 943–961. 10.3233/JAD-2012-111661 22495349 PMC3378773

[B49] Silva P. N. Furuya T. K. Braga I. L. Rasmussen L. T. Labio R. W. Bertolucci P. H.. (2014). Analysis of HSPA8 and HSPA9 mRNA expression and promoter methylation in the brain and blood of Alzheimer’s disease patients. J. Alzheimers Dis. 38, 165–170. 10.3233/JAD-130428 23948933

[B108] Slowik A. Merres J. Elfgen A. Jansen S. Mohr F. Wruck C. J.. (2012). Involvement of formyl peptide receptors in receptor for advanced glycation end products (RAGE)—and amyloid β 1–42-induced signal transduction in glial cells. Mol. Neurodegener. 7:55. 10.1186/1750-1326-7-55 23164356 PMC3519738

[B50] Son S. M. Song H. Byun J. Park K. S. Jang H. C. Park Y. J.. (2012). Accumulation of autophagosomes contributes to enhanced amyloidogenic APP processing under insulin-resistant conditions. Autophagy 8, 1842–1844. 10.4161/auto.21861 22931791 PMC3541299

[B109] Sperling R. A. Aisen P. S. Beckett L. A. Bennett D. A. Craft S. Fagan A. M.. (2011). Toward defining the preclinical stages of Alzheimer’s disease: recommendations from the National Institute on Aging-Alzheimer’s Association workgroups on diagnostic guidelines for Alzheimer’s disease. Alzheimers Dement. 7, 280–292. 10.1016/j.jalz.2011.03.003 21514248 PMC3220946

[B51] Takeda S. Sato N. Uchio-Yamada K. Sawada K. Kunieda T. Takeuchi D.. (2010). Diabetes-accelerated memory dysfunction via cerebrovascular inflammation and Aβ deposition in an Alzheimer mouse model with diabetes. Proc. Natl. Acad. Sci. U S A 107, 7036–7041. 10.1073/pnas.1000645107 20231468 PMC2872449

[B52] Talbot K. Wang H. Y. Kazi H. Han L. Y. Bakshi K. P. Stucky A.. (2012). Demonstrated brain insulin resistance in Alzheimer’s disease patients is associated with IGF-1 resistance, IRS-1 dysregulation and cognitive decline. J. Clin. Invest. 122, 1316–1338. 10.1172/JCI59903 22476197 PMC3314463

[B53] Tramutola A. Di Domenico F. Barone E. Perluigi M. Butterfield D. A. (2016). It is all about (U)biquitin: role of altered ubiquitin-proteasome system and UCHL1 in Alzheimer disease. Oxid. Med. Cell. Longev. 2016:2756068. 10.1155/2016/2756068 26881020 PMC4736377

[B54] Vandal M. White P. J. Tremblay C. St-Amour I. Chevrier G. Emond V.. (2014). Insulin reverses the high-fat diet-induced increase in brain Aβ and improves memory in an animal model of Alzheimer disease. Diabetes 63, 4291–4301. 10.2337/db14-0375 25008180

[B55] Wang Z. Yan X. Zhao C. (2017). Dynamical differential networks and modules inferring disrupted genes associated with the progression of Alzheimer’s disease. Exp. Ther. Med. 14, 2969–2975. 10.3892/etm.2017.4905 28966679 PMC5613183

[B56] Wei N. Zhu L. Q. Liu D. (2015). ATF4: a novel potential therapeutic target for Alzheimer’s disease. Mol. Neurobiol. 52, 1765–1770. 10.1007/s12035-014-8970-8 25381575

[B110] Ye X. Zhang J. Lu R. Zhou G. (2016). Signal regulatory protein α associated with the progression of oral leukoplakia and oral squamous cell carcinoma regulates phenotype switch of macrophages. Oncotarget 7, 81305–81321. 10.18632/oncotarget.12874 27793032 PMC5348394

[B111] Yu Y. Ye R. D. (2015). Microglial β receptors in Alzheimer’s disease. Cell. Mol. Neurobiol. 35, 71–83. 10.1007/s10571-014-0101-6 25149075 PMC11486233

[B58] Zhang Y. Chen X. Zhao Y. Ponnusamy M. Liu Y. (2017). The role of ubiquitin proteasomal system and autophagy-lysosome pathway in Alzheimer’s disease. Rev. Neurosci. 28, 861–868. 10.1515/revneuro-2017-0013 28704199

[B57] Zhang L. Guo X. Q. Chu J. F. Zhang X. Yan Z. R. Li Y. Z. (2015). Potential hippocampal genes and pathways involved in Alzheimer’s disease: a bioinformatic analysis. Genet. Mol. Res. 14, 7218–7232. 10.4238/2015.june.29.15 26125932

[B59] Zhu D. Yang N. Liu Y. Y. Zheng J. Ji C. Zuo P. P. (2016). M2 macrophage transplantation ameliorates cognitive dysfunction in amyloid-β-treated rats through regulation of microglial polarization. J. Alzheimers Dis. 52, 483–495. 10.3233/JAD-151090 27003214

[B60] Zougman A. Mann M. Wisniewski J. R. (2011). Identification and characterization of a novel ubiquitous nucleolar protein ‘NARR’ encoded by a gene overlapping the rab34 oncogene. Nucleic Acids Res. 39, 7103–7113. 10.1093/nar/gkr273 21586586 PMC3167632

