# Supplementary figures and images for: Identification of Temporal Characteristic Networks of Peripheral Blood Changes in Alzheimer’s Disease Based on Weighted Gene Co-expression Network Analysis (part 1 of 2)
Source: Front Aging Neurosci. 2019 May 21;11:83. doi: 10.3389/fnagi.2019.00083 (PMC6537635; doi:10.3389/fnagi.2019.00083)

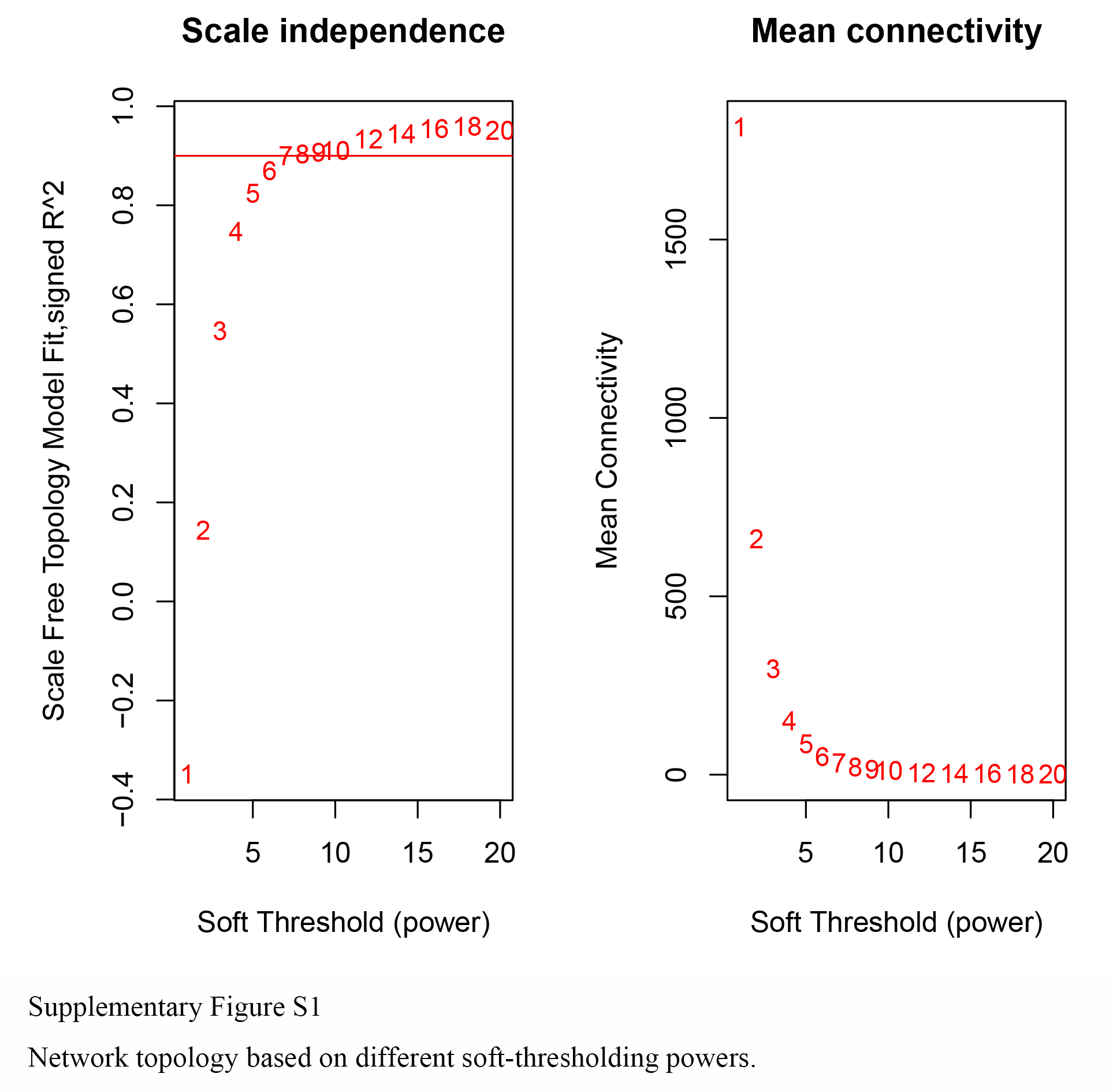

Supplement: Supplementary file 1 [file Image_1.tif]

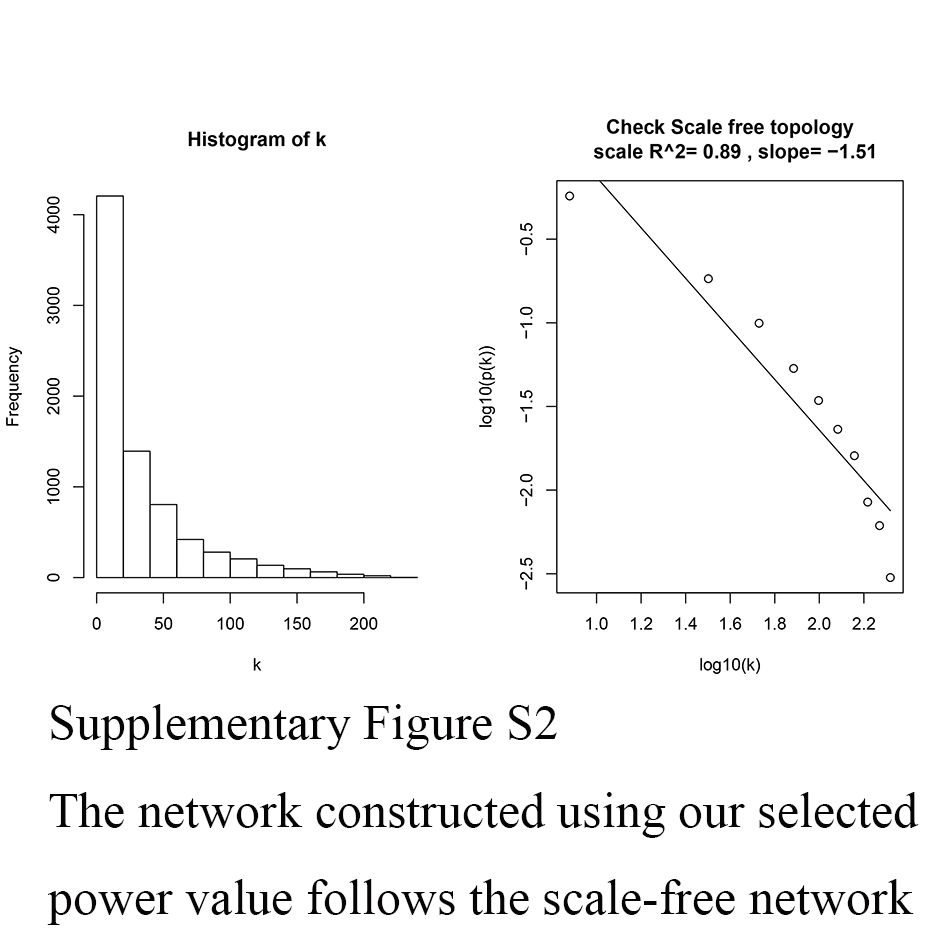

Supplement: Supplementary file 2 [file Image_2.tif]

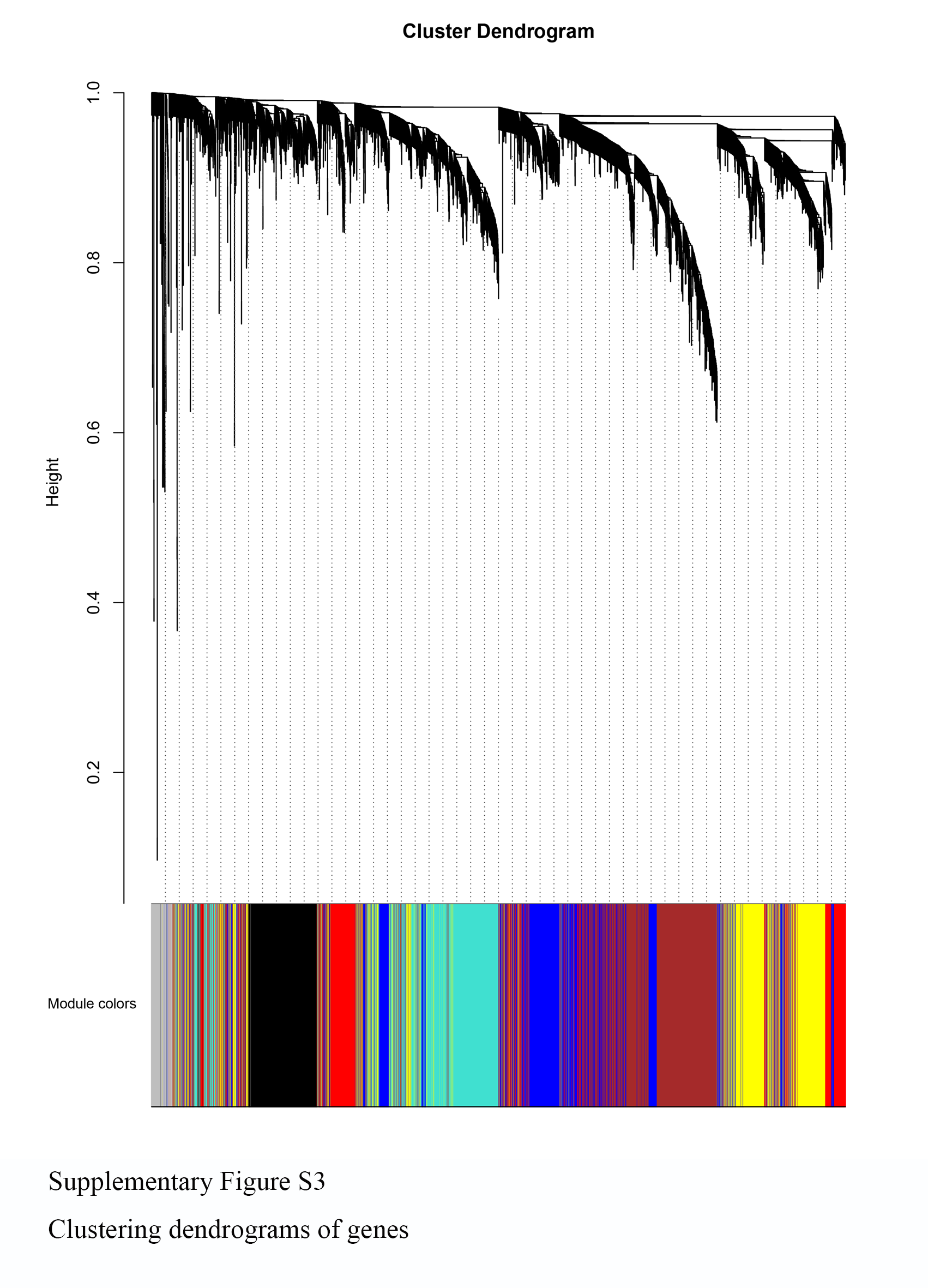

Supplement: Supplementary file 3 [file Image_3.tif]

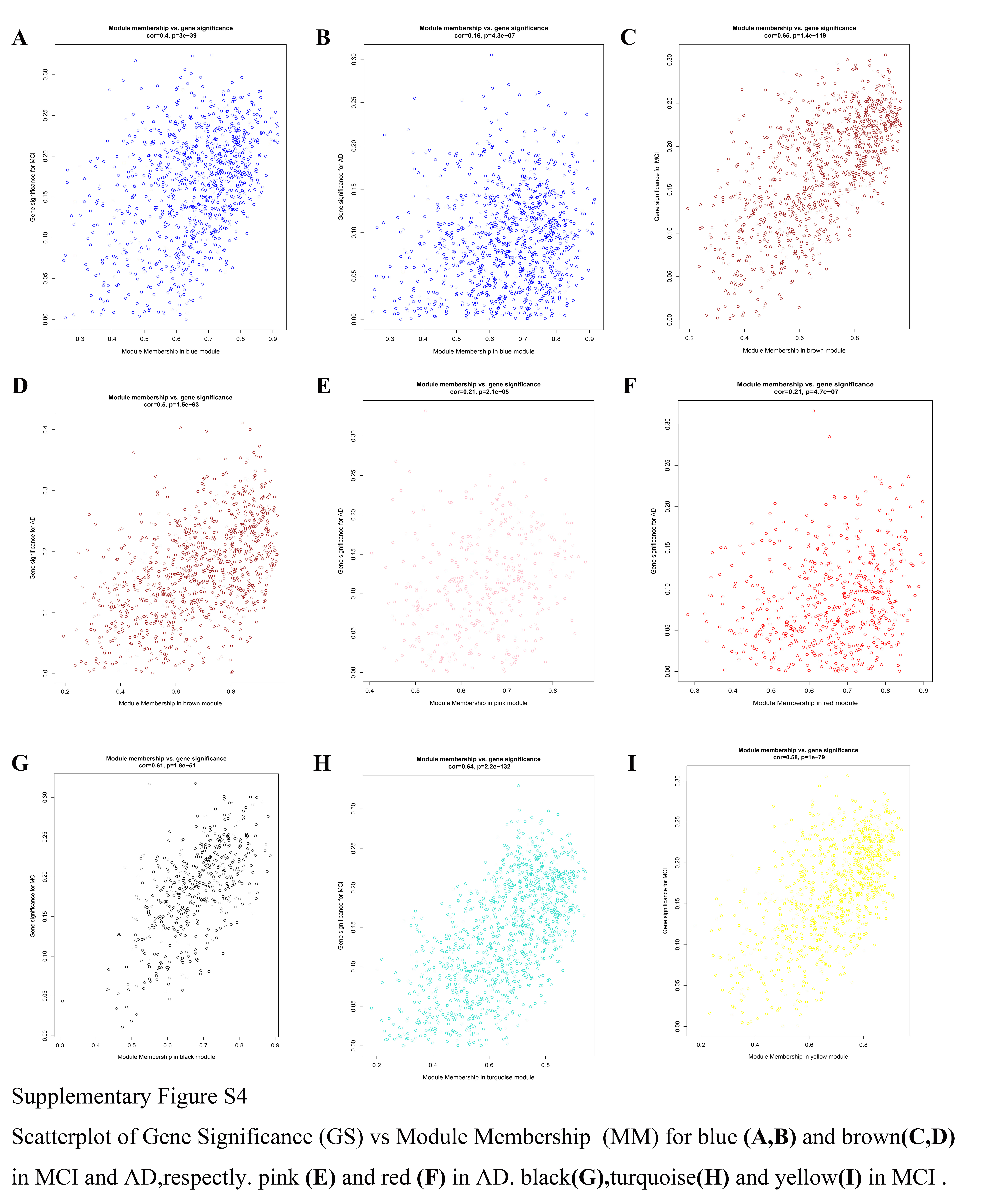

Supplement: Supplementary file 4 [file Image_4.jpg]

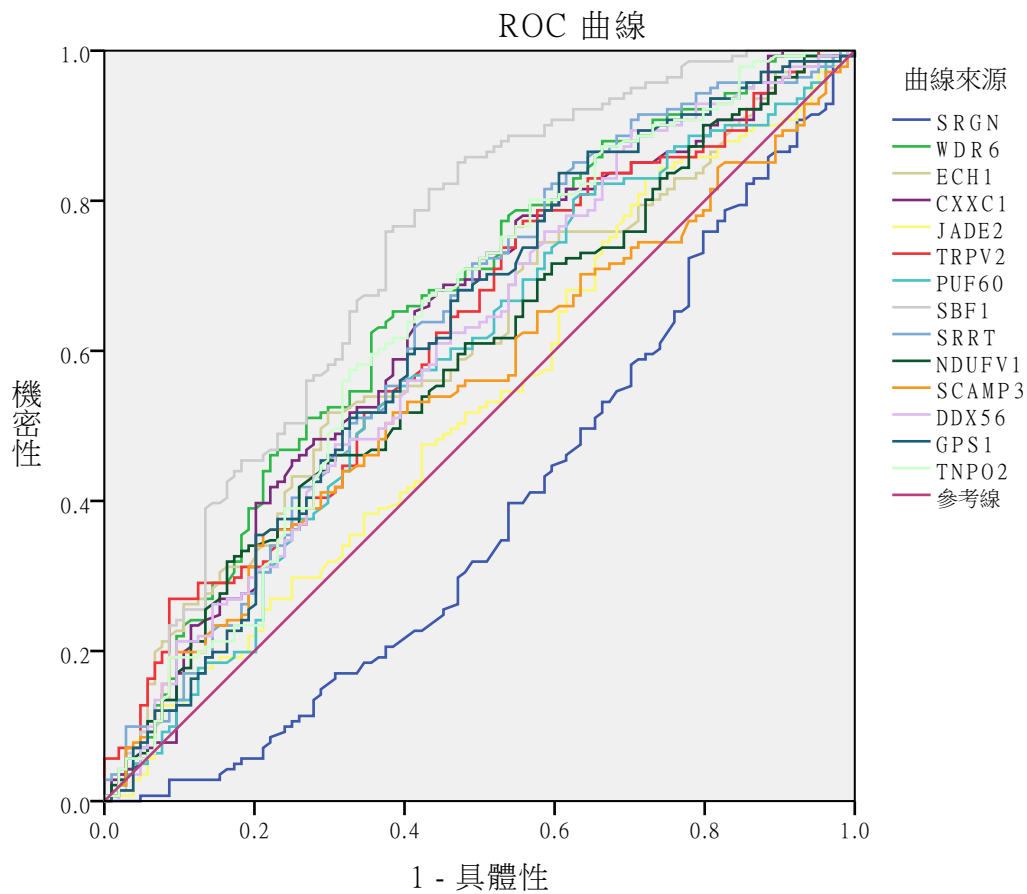

同分產生的對數區段。

Supplement: Supplementary file 5 [file Data_Sheet_1.ZIP › Supplementary Materials S1/ROC/ROC GSE63060 BLACK AD-CTL DG .pdf]

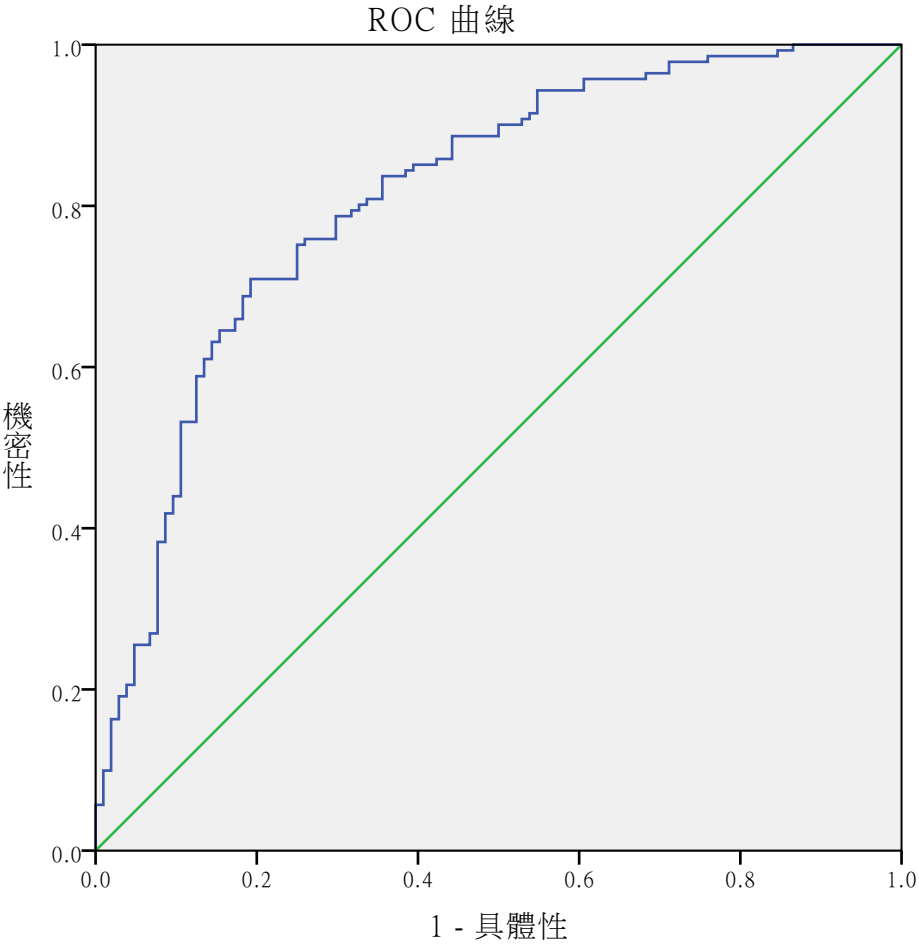

Supplement: Supplementary file 5 [file Data_Sheet_1.ZIP › Supplementary Materials S1/ROC/ROC GSE63060 BLACK AD-CTL LH .pdf]

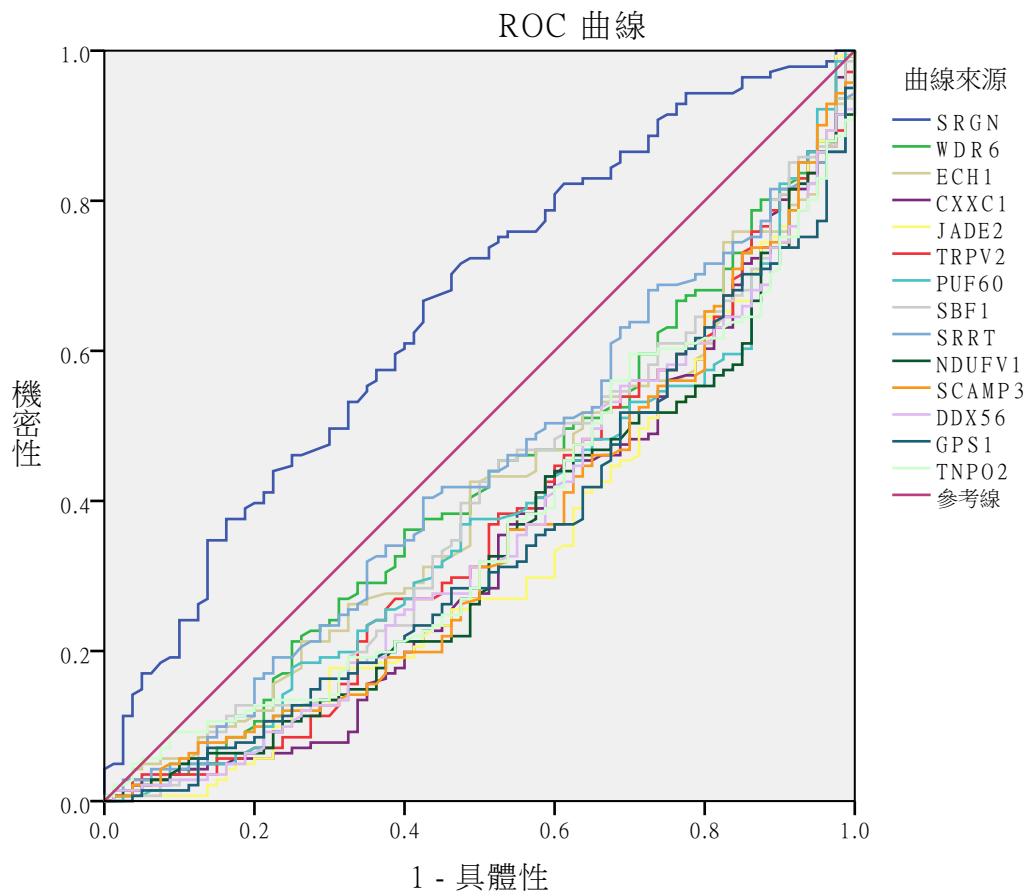

同分產生的對數區段。

Supplement: Supplementary file 5 [file Data_Sheet_1.ZIP › Supplementary Materials S1/ROC/ROC GSE63060 BLACK AD-MCI DG BG.pdf]

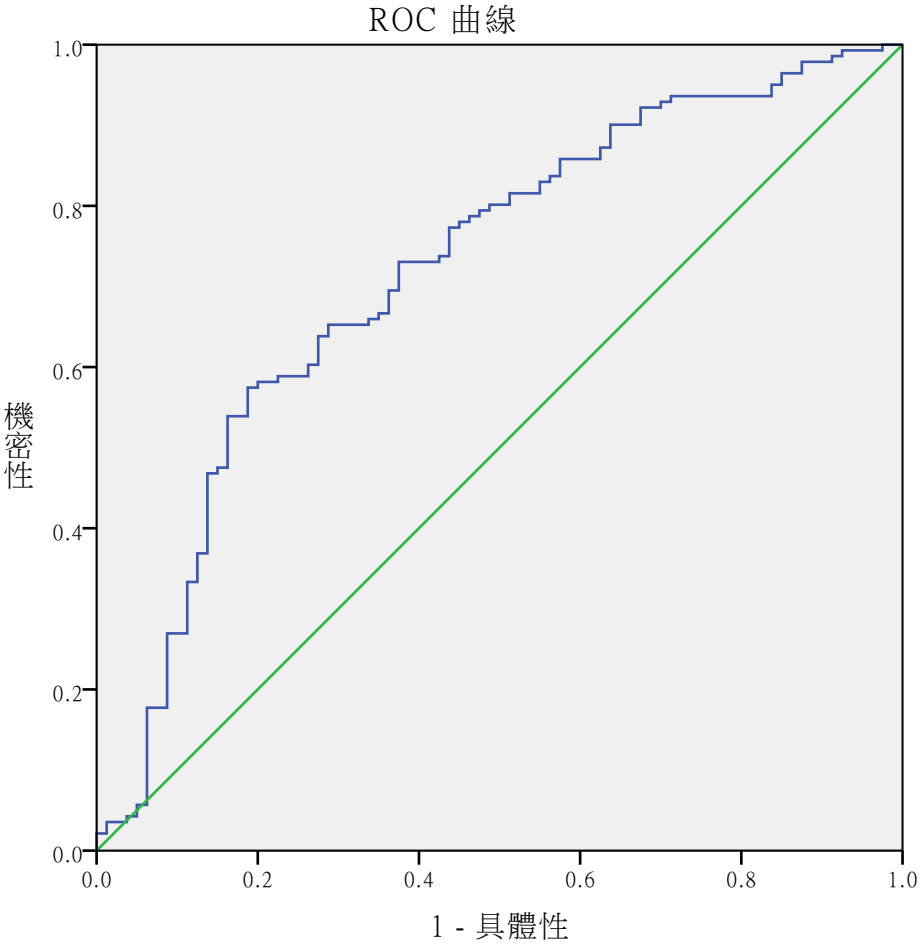

Supplement: Supplementary file 5 [file Data_Sheet_1.ZIP › Supplementary Materials S1/ROC/ROC GSE63060 BLACK AD-MCI LH .pdf]

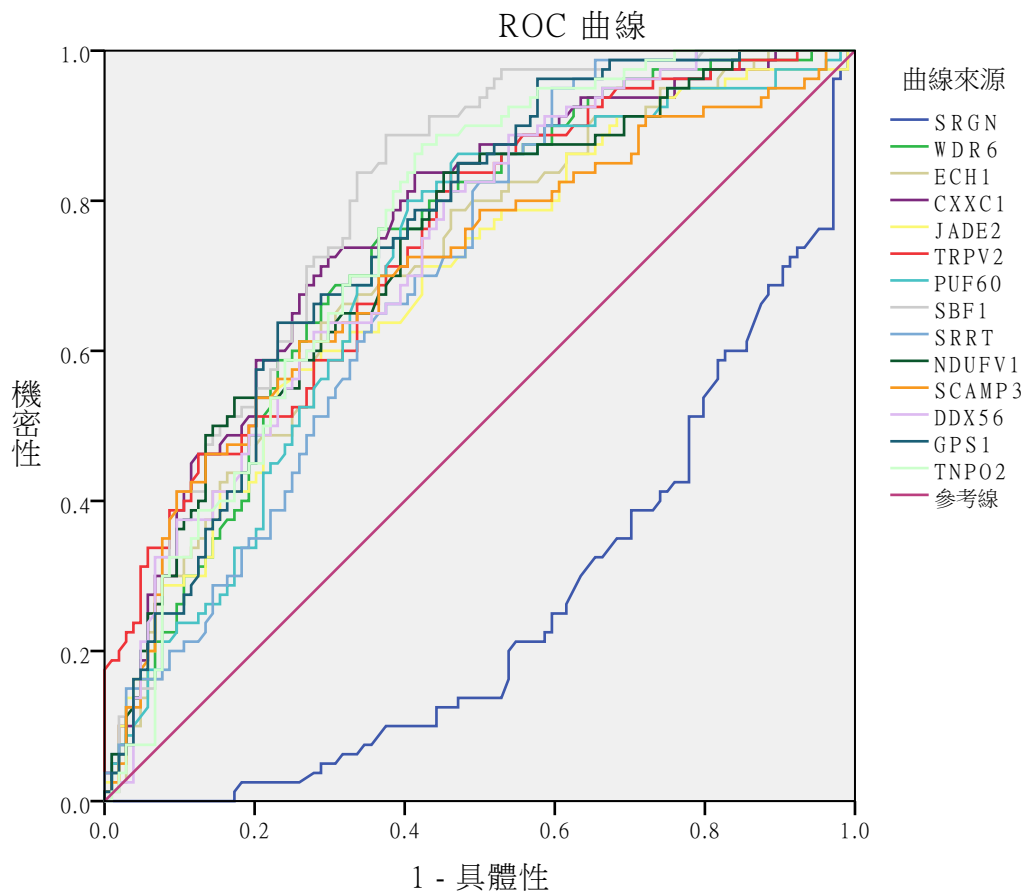

同分產生的對數區段。

Supplement: Supplementary file 5 [file Data_Sheet_1.ZIP › Supplementary Materials S1/ROC/ROC GSE63060 BLACK MCI-CTL DG.pdf]

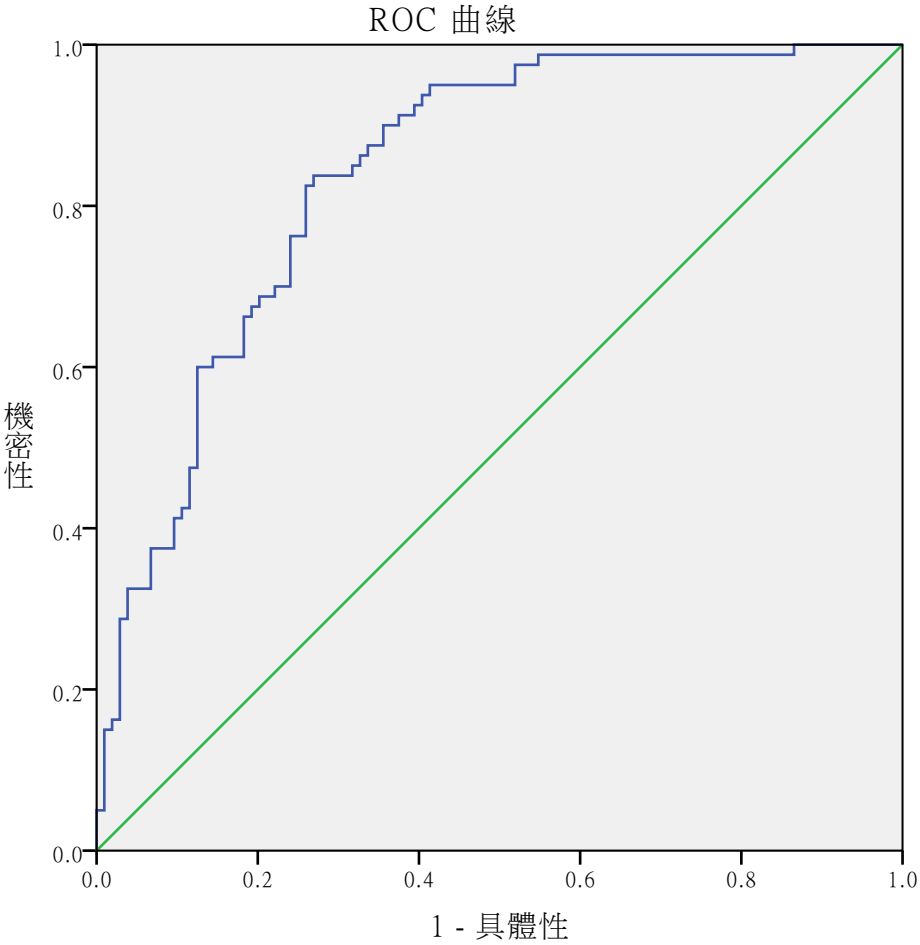

Supplement: Supplementary file 5 [file Data_Sheet_1.ZIP › Supplementary Materials S1/ROC/ROC GSE63060 BLACK MCI-CTL LH .pdf]

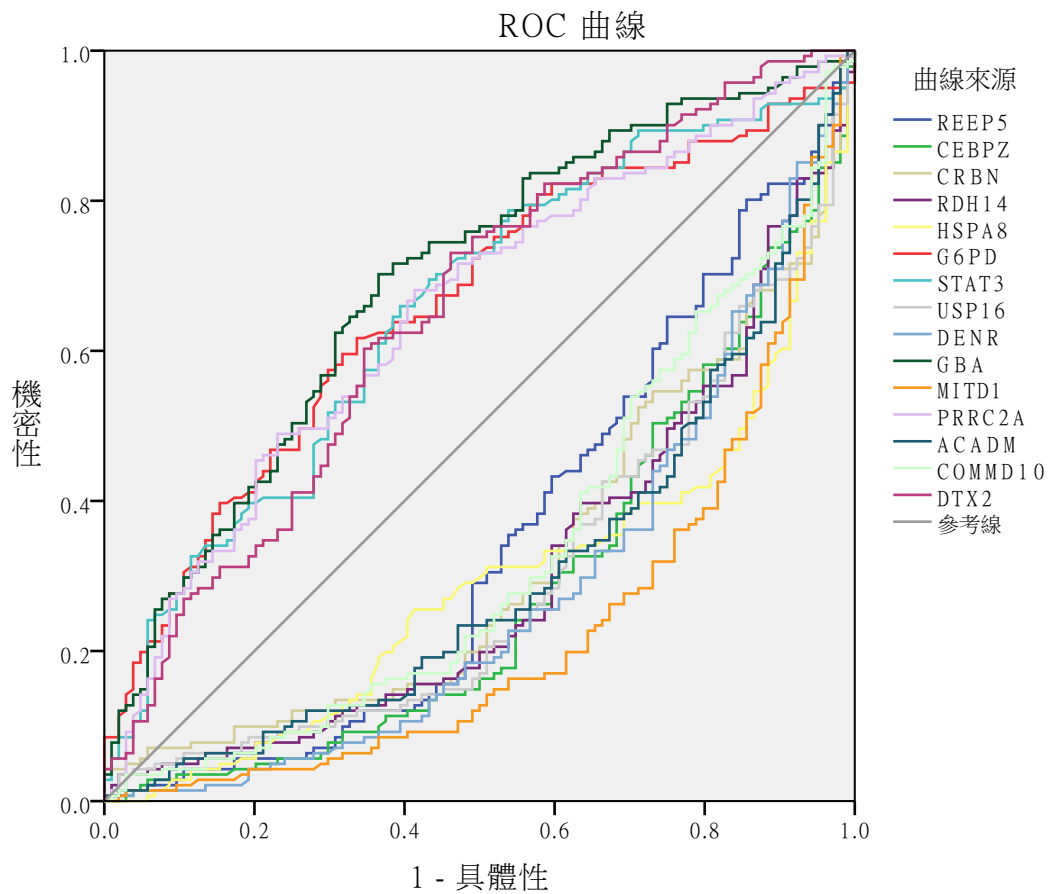

同分產生的對數區段。

Supplement: Supplementary file 5 [file Data_Sheet_1.ZIP › Supplementary Materials S1/ROC/ROC GSE63060 BLUE AD-CTL DG.pdf]

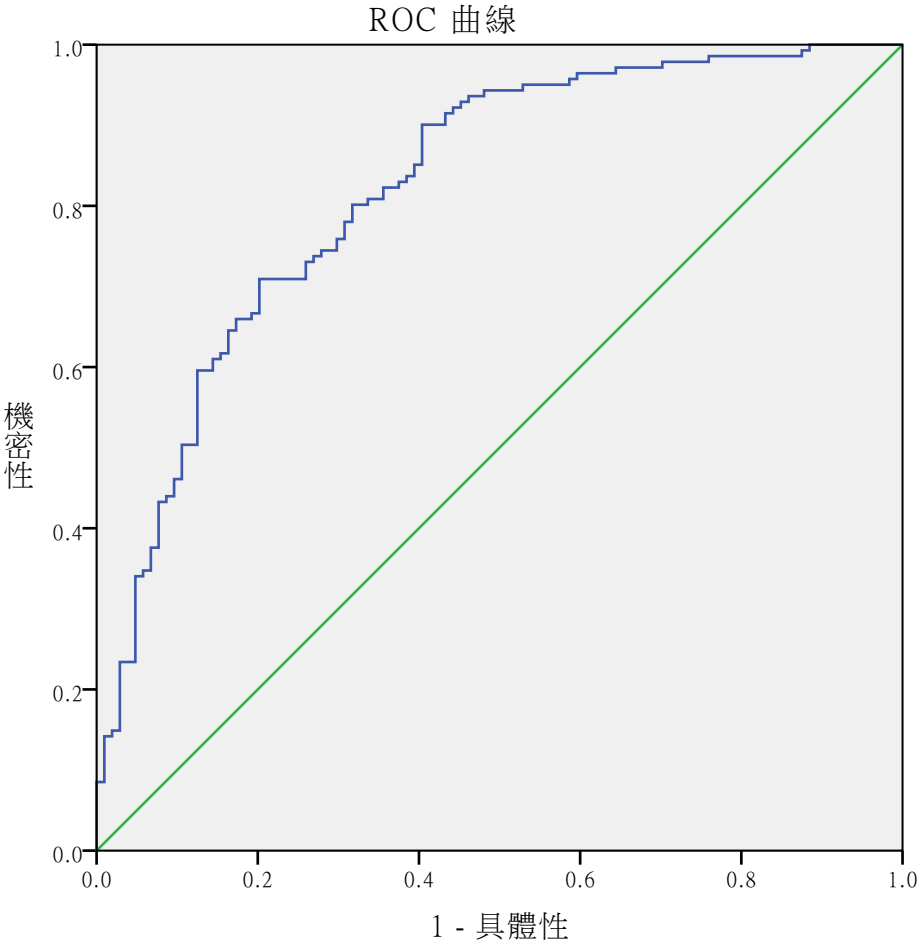

Supplement: Supplementary file 5 [file Data_Sheet_1.ZIP › Supplementary Materials S1/ROC/ROC GSE63060 BLUE AD-CTL LH.pdf]

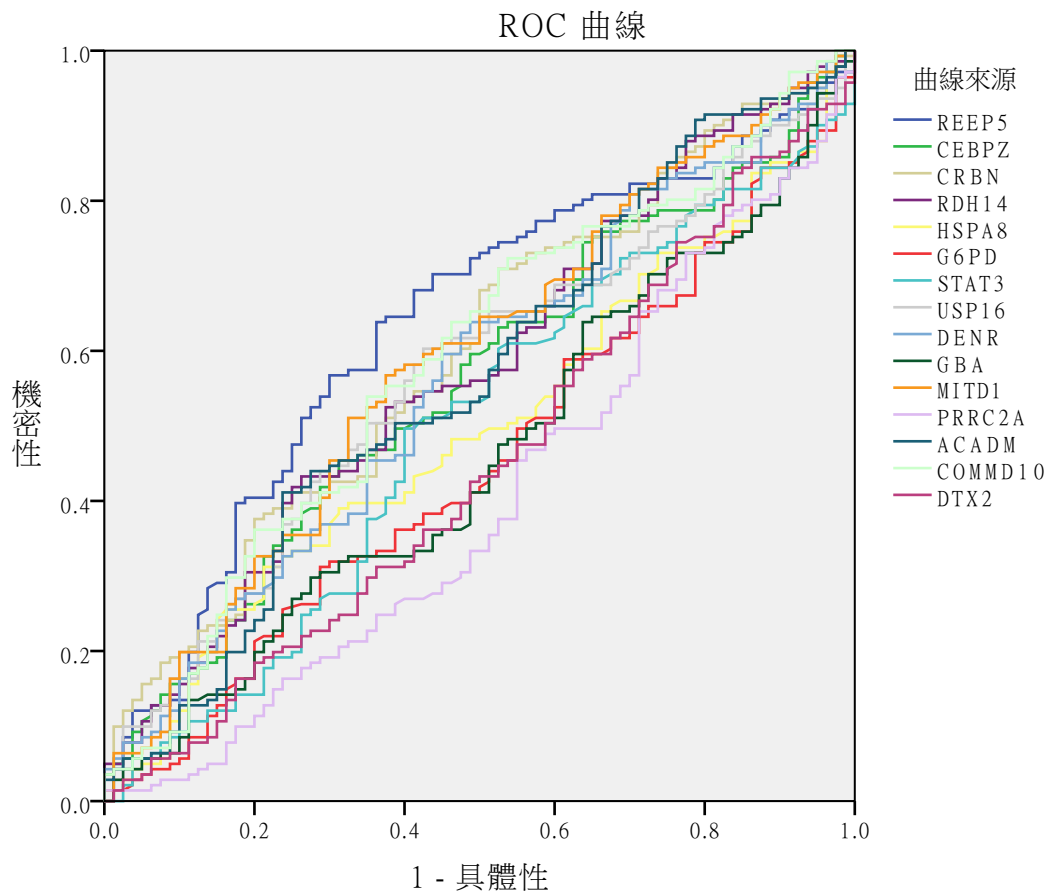

同分產生的對數區段。

Supplement: Supplementary file 5 [file Data_Sheet_1.ZIP › Supplementary Materials S1/ROC/ROC GSE63060 BLUE AD-MCI DG .pdf]

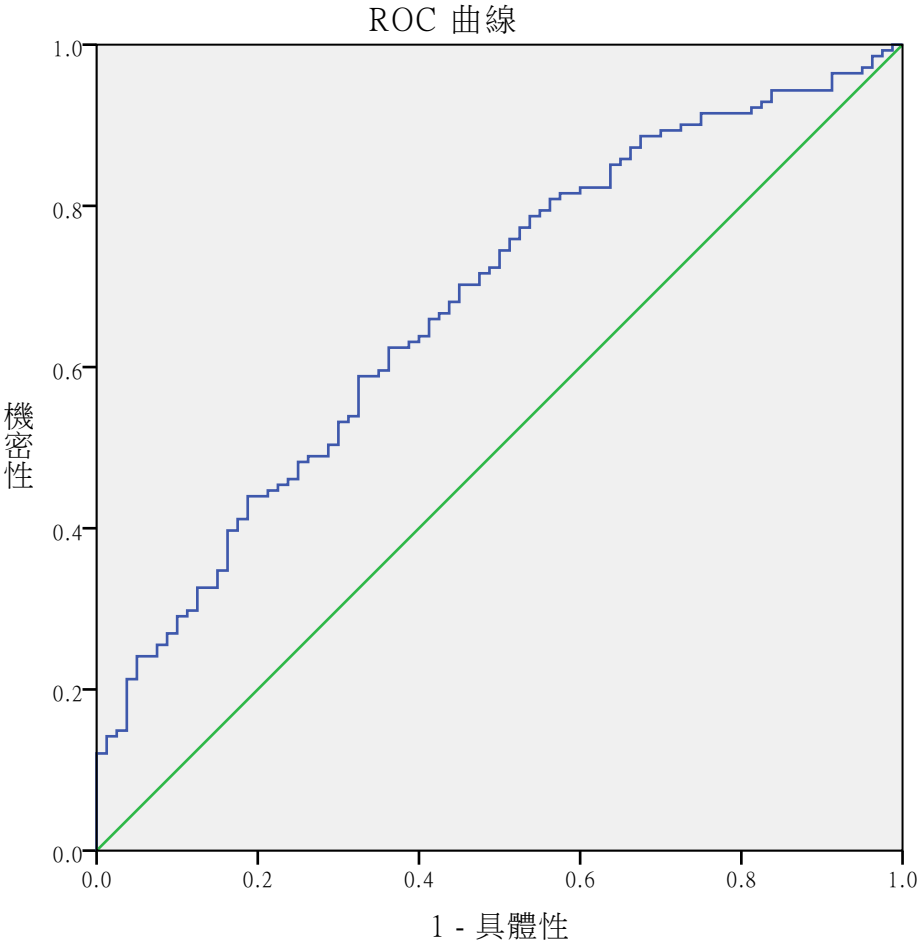

Supplement: Supplementary file 5 [file Data_Sheet_1.ZIP › Supplementary Materials S1/ROC/ROC GSE63060 BLUE AD-MCI LH.pdf]

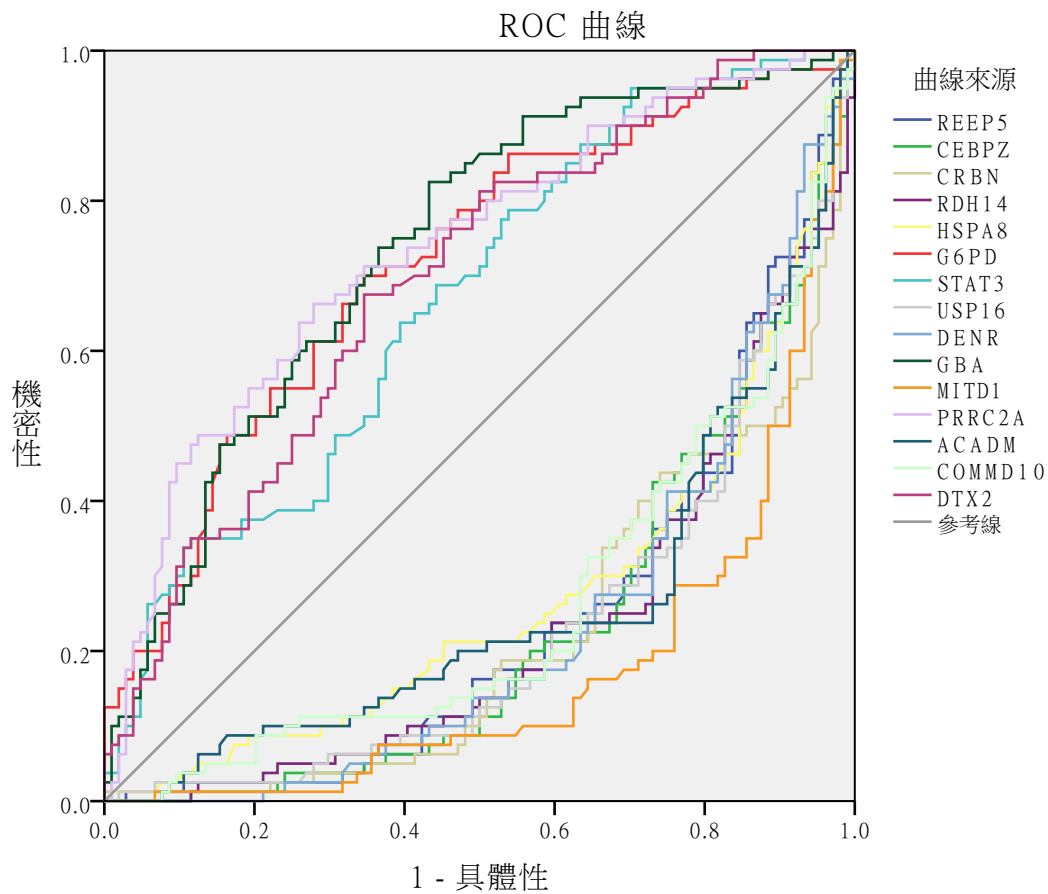

同分產生的對數區段。

Supplement: Supplementary file 5 [file Data_Sheet_1.ZIP › Supplementary Materials S1/ROC/ROC GSE63060 BLUE MCI-CTL DG.pdf]

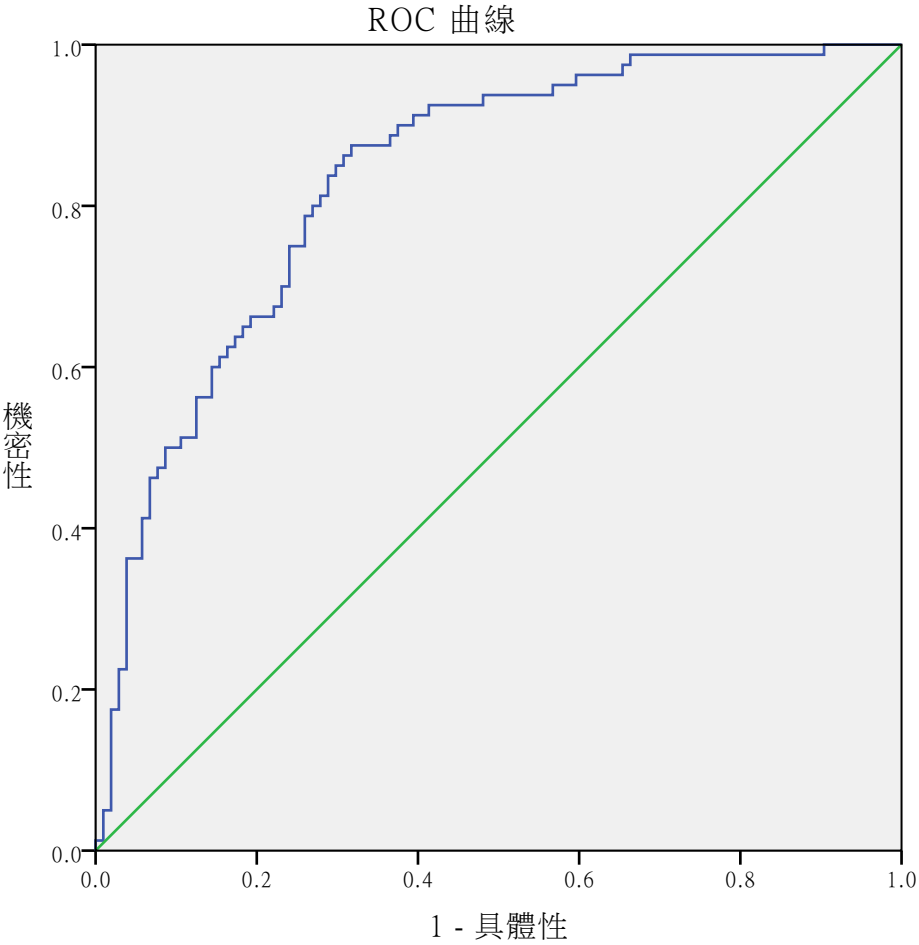

Supplement: Supplementary file 5 [file Data_Sheet_1.ZIP › Supplementary Materials S1/ROC/ROC GSE63060 BLUE MCI-CTL LH .pdf]

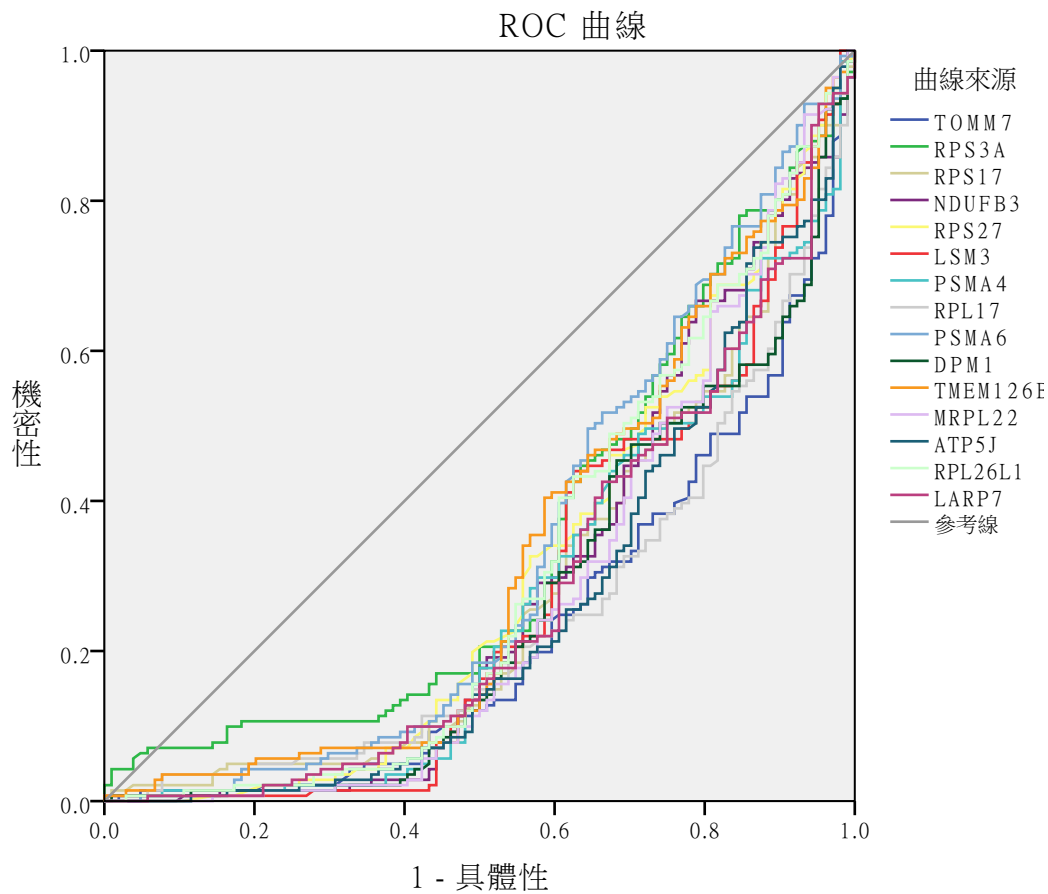

同分產生的對數區段。

Supplement: Supplementary file 5 [file Data_Sheet_1.ZIP › Supplementary Materials S1/ROC/ROC GSE63060 BROWN AD-CTL DG.pdf]

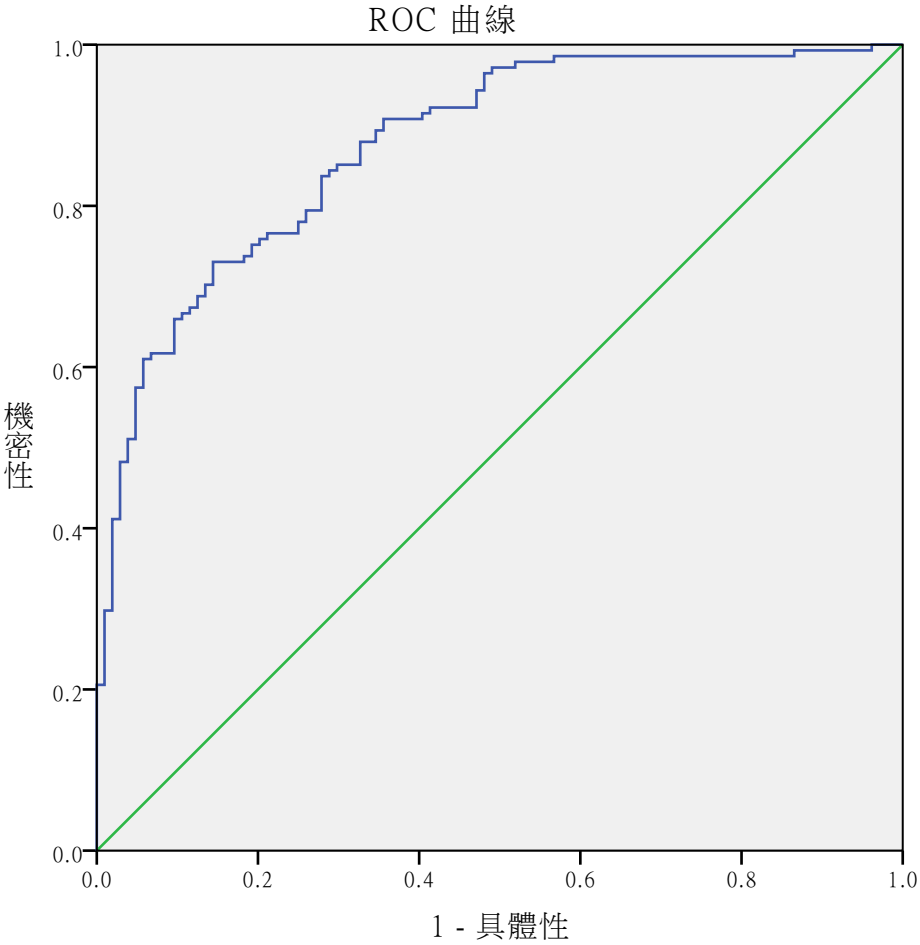

Supplement: Supplementary file 5 [file Data_Sheet_1.ZIP › Supplementary Materials S1/ROC/ROC GSE63060 BROWN AD-CTLLH.pdf]

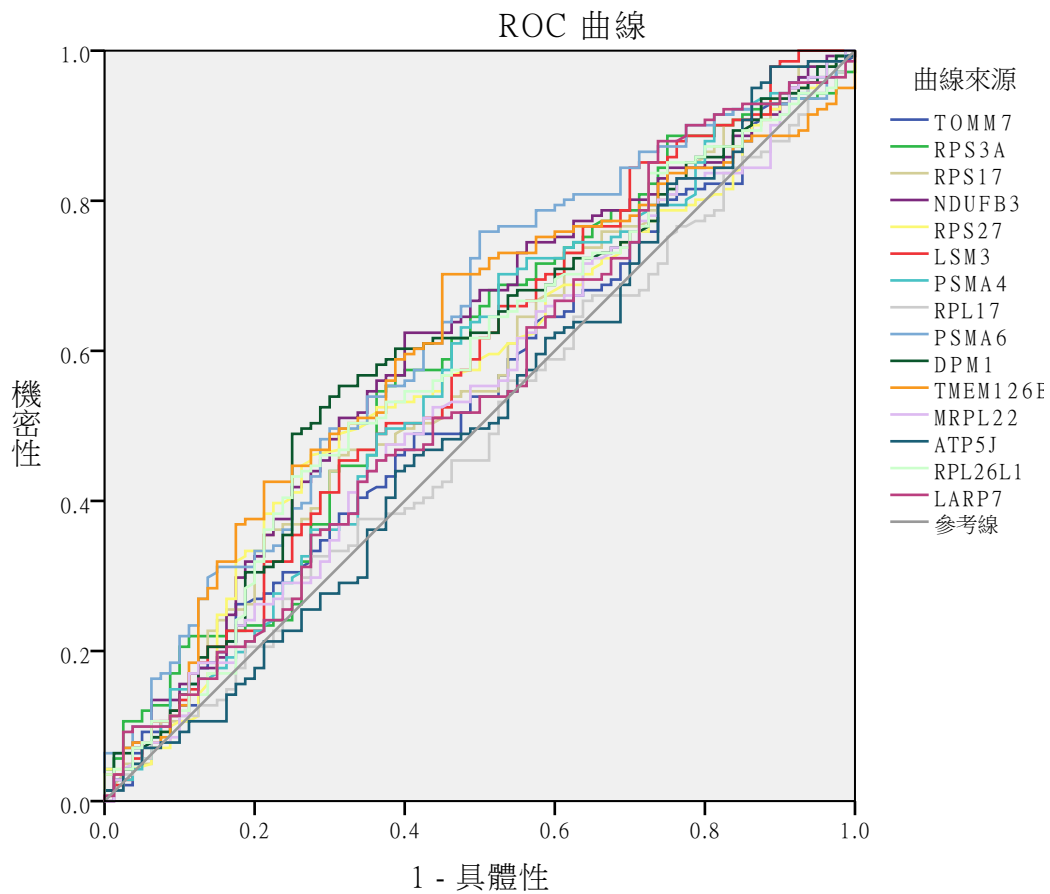

同分產生的對數區段。

Supplement: Supplementary file 5 [file Data_Sheet_1.ZIP › Supplementary Materials S1/ROC/ROC GSE63060 BROWN AD-MCI DG.pdf]

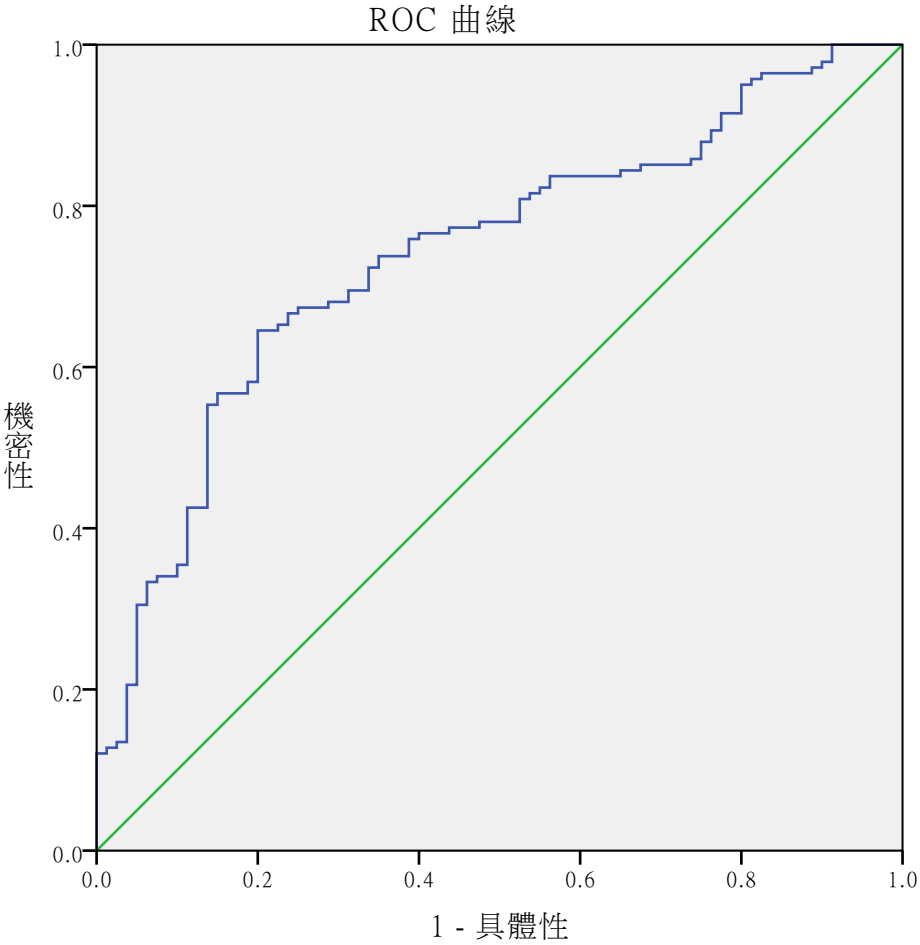

Supplement: Supplementary file 5 [file Data_Sheet_1.ZIP › Supplementary Materials S1/ROC/ROC GSE63060 BROWN AD-MCI LH.pdf]

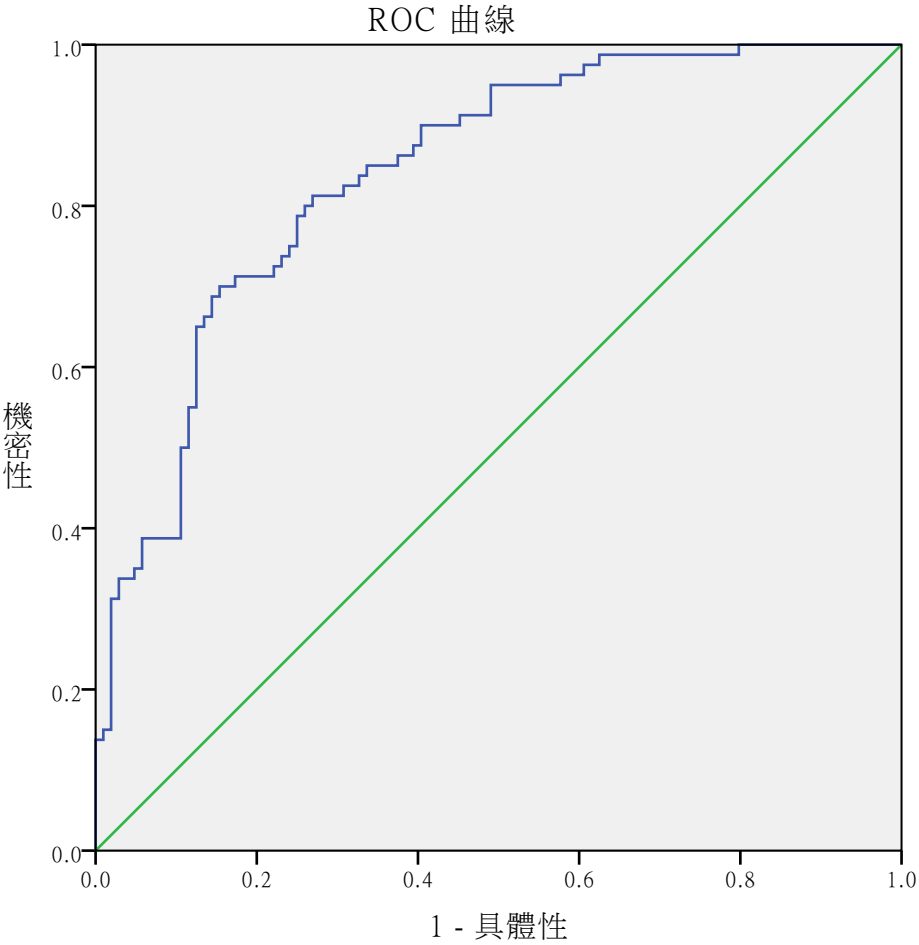

Supplement: Supplementary file 5 [file Data_Sheet_1.ZIP › Supplementary Materials S1/ROC/ROC GSE63060 BROWN MCI-CTL LH.pdf]

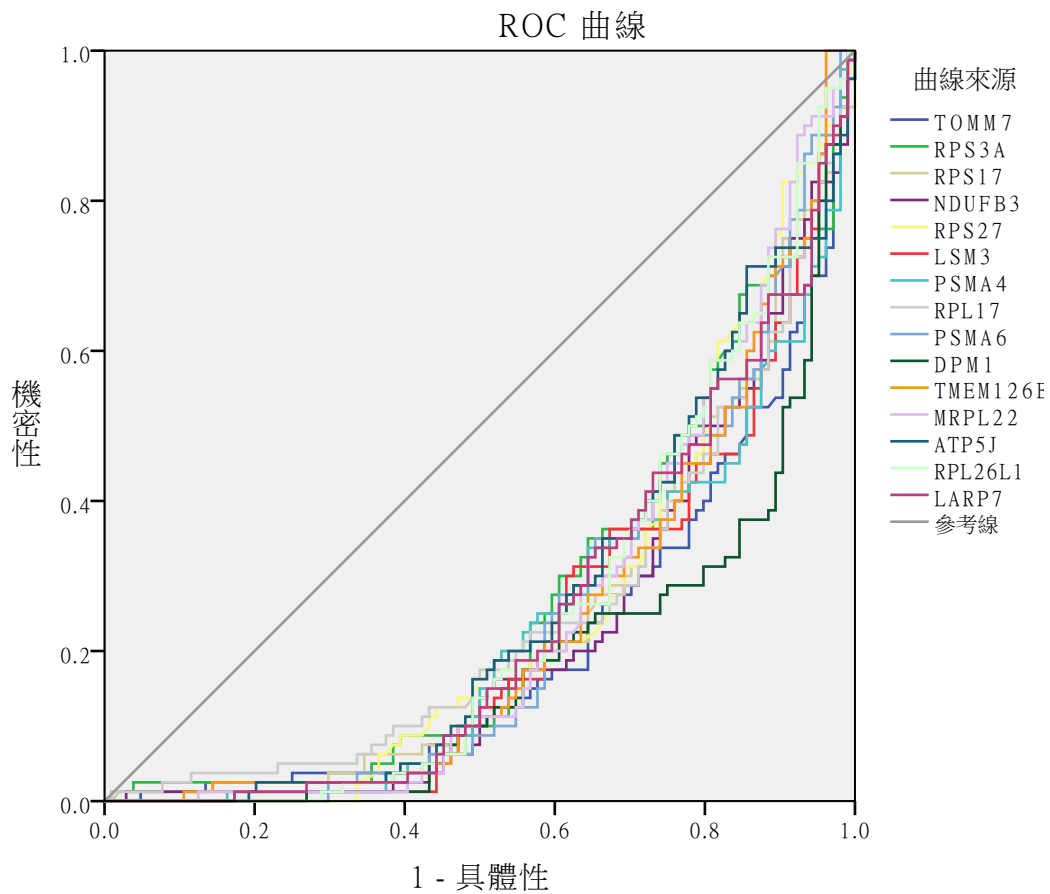

同分產生的對數區段。

Supplement: Supplementary file 5 [file Data_Sheet_1.ZIP › Supplementary Materials S1/ROC/ROC GSE63060 BROWN MCI-CTLDG.pdf]

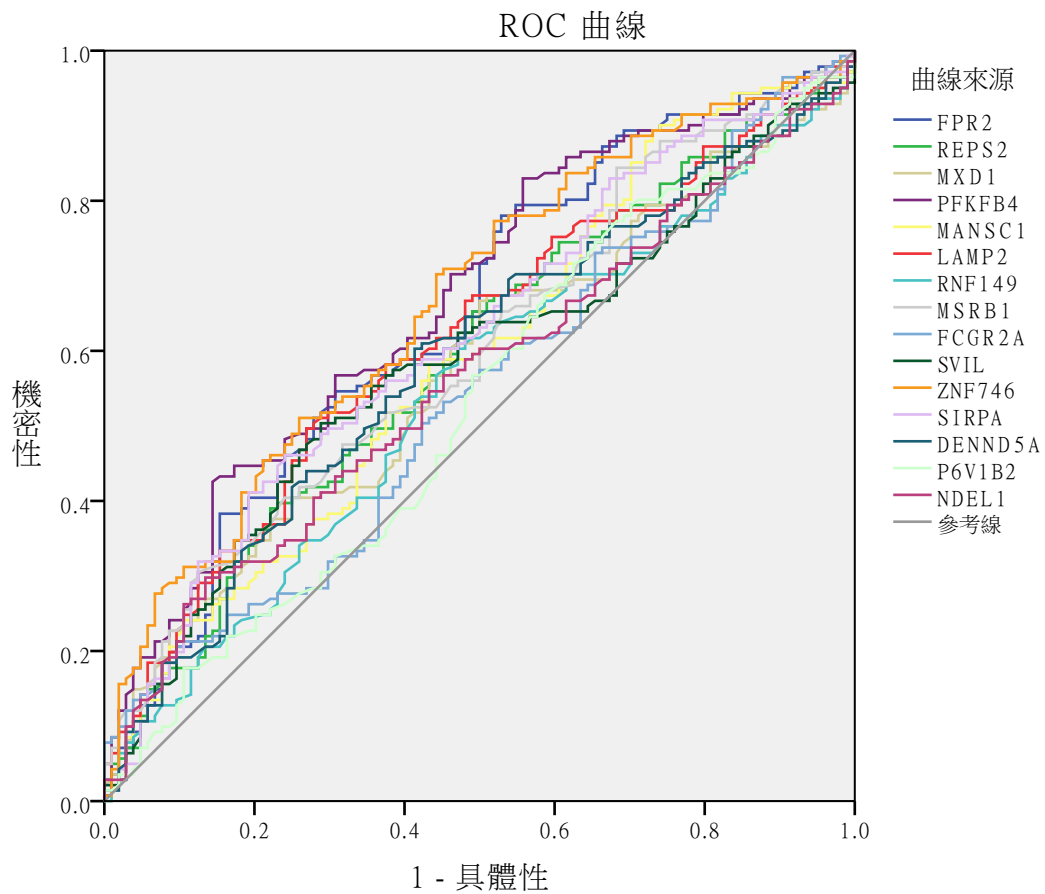

同分產生的對數區段。

Supplement: Supplementary file 5 [file Data_Sheet_1.ZIP › Supplementary Materials S1/ROC/ROC GSE63060 PINK AD-CTL DG.pdf]

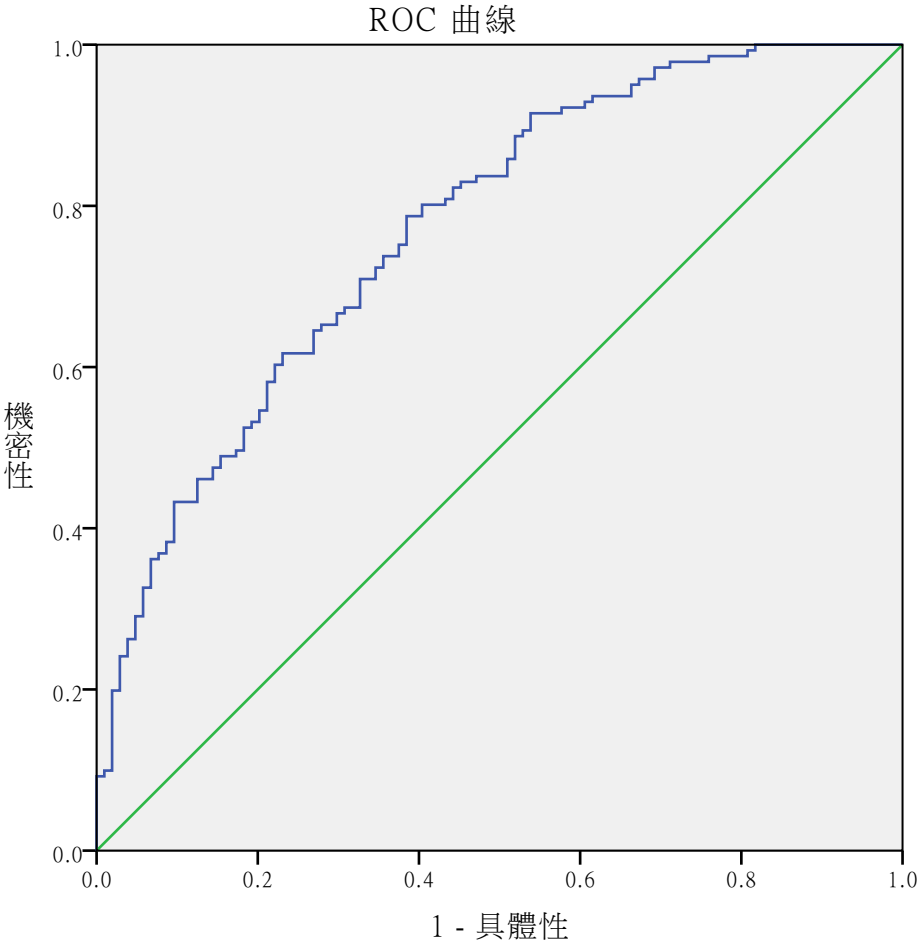

Supplement: Supplementary file 5 [file Data_Sheet_1.ZIP › Supplementary Materials S1/ROC/ROC GSE63060 PINK AD-CTL LH.pdf]

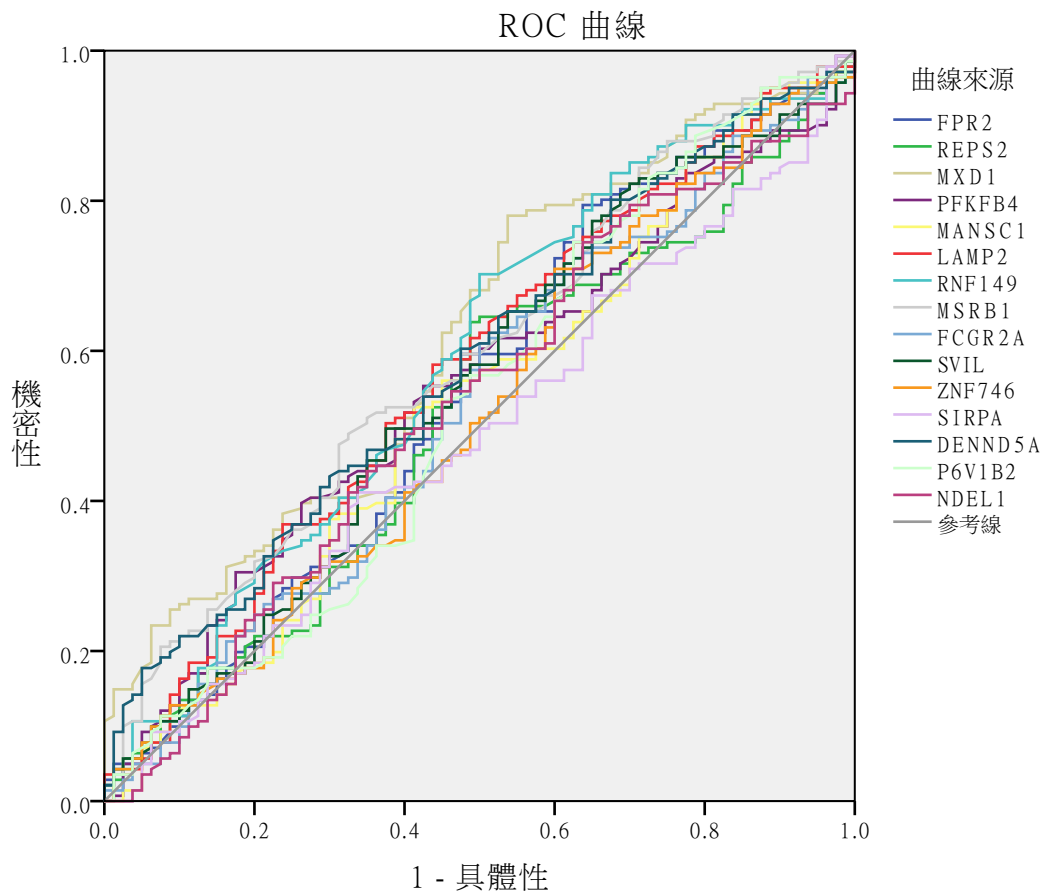

同分產生的對數區段。

Supplement: Supplementary file 5 [file Data_Sheet_1.ZIP › Supplementary Materials S1/ROC/ROC GSE63060 PINK AD-MCI DG .pdf]

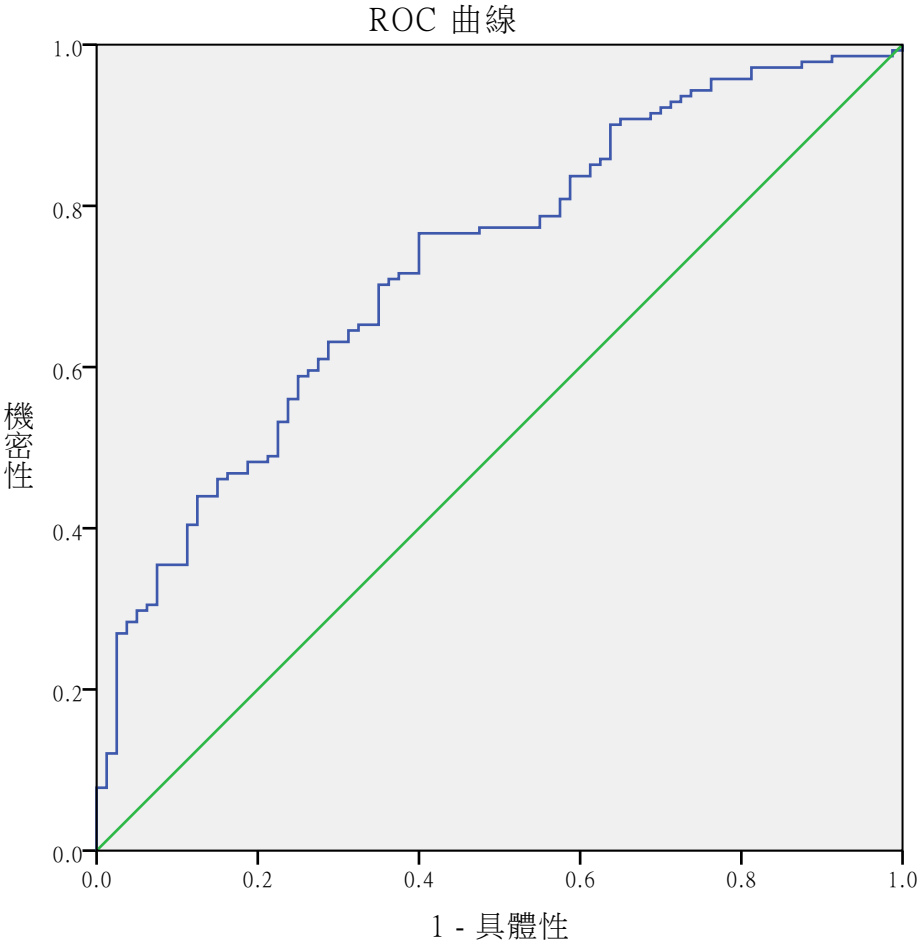

Supplement: Supplementary file 5 [file Data_Sheet_1.ZIP › Supplementary Materials S1/ROC/ROC GSE63060 PINK AD-MCI LH.pdf]

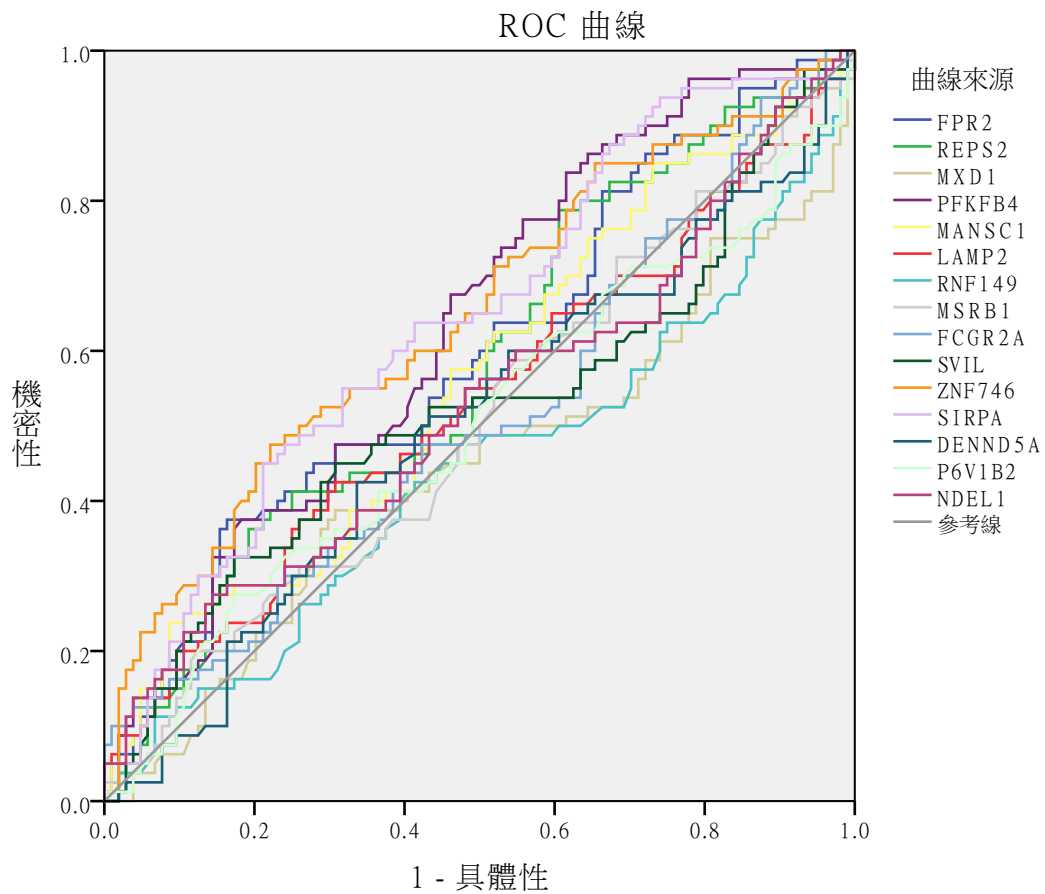

同分產生的對數區段。

Supplement: Supplementary file 5 [file Data_Sheet_1.ZIP › Supplementary Materials S1/ROC/ROC GSE63060 PINK MCI-CTL DG.pdf]

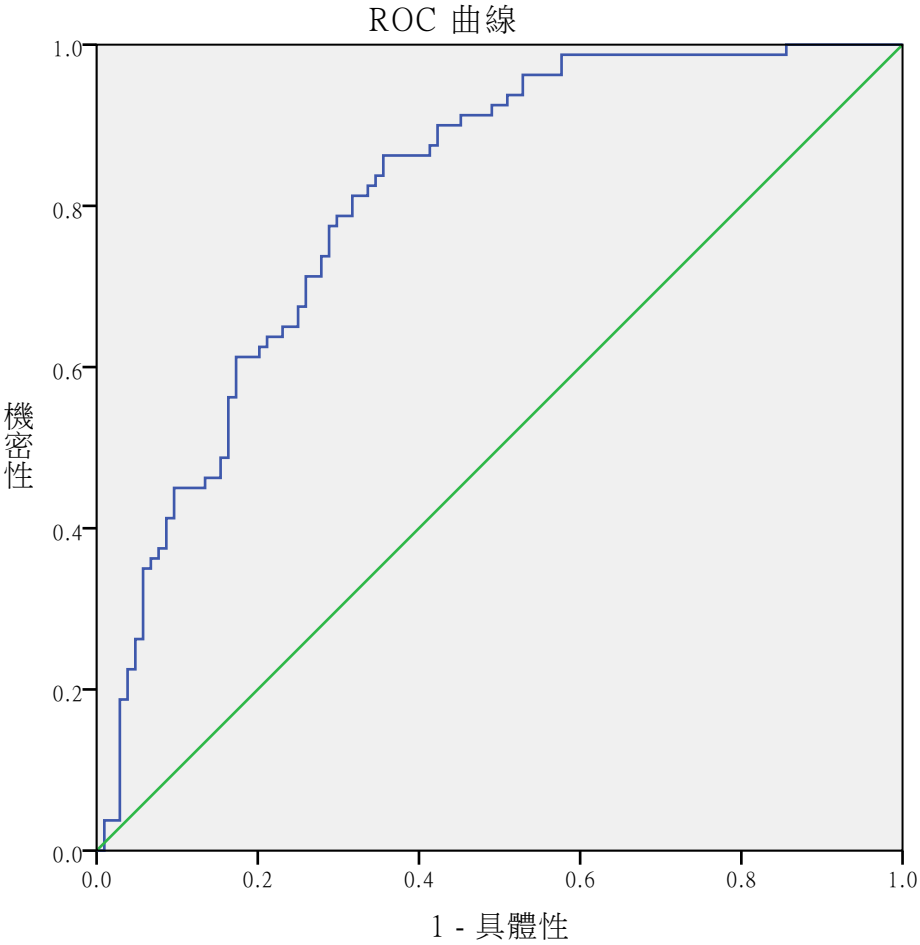

Supplement: Supplementary file 5 [file Data_Sheet_1.ZIP › Supplementary Materials S1/ROC/ROC GSE63060 PINK MCI-CTL LH .pdf]

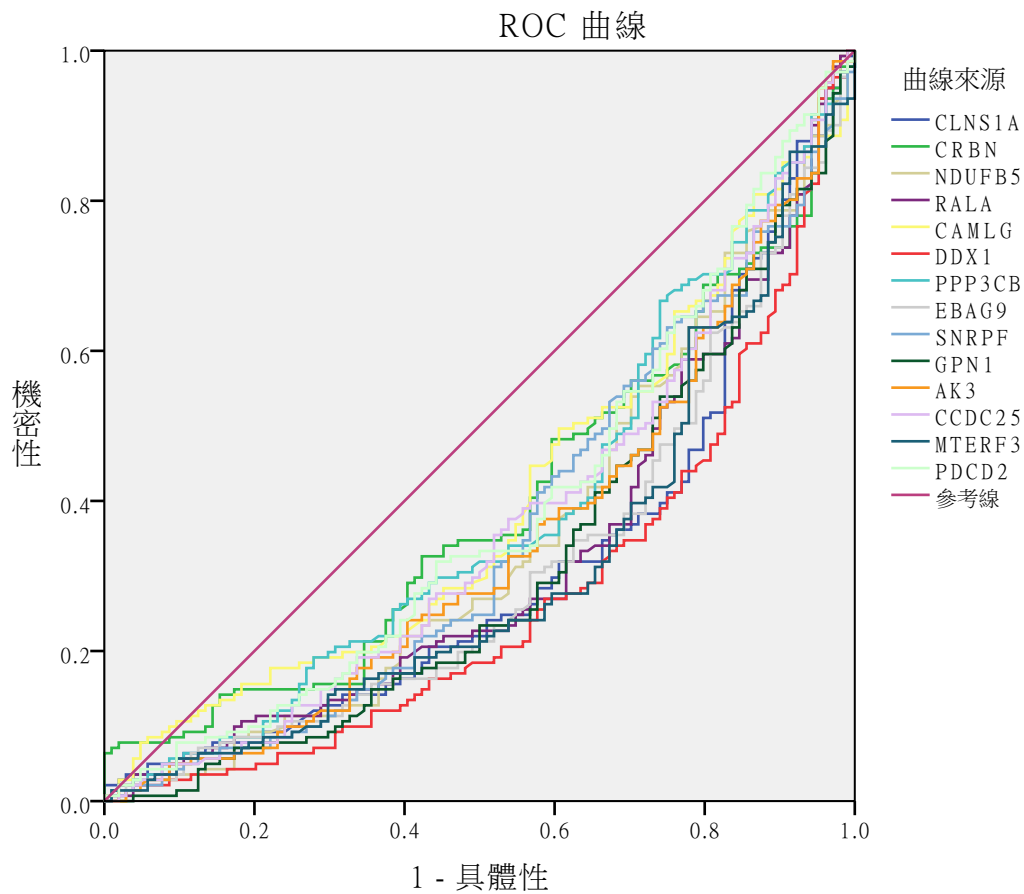

同分產生的對數區段。

Supplement: Supplementary file 5 [file Data_Sheet_1.ZIP › Supplementary Materials S1/ROC/ROC GSE63060 RED AD-CTL DG.pdf]

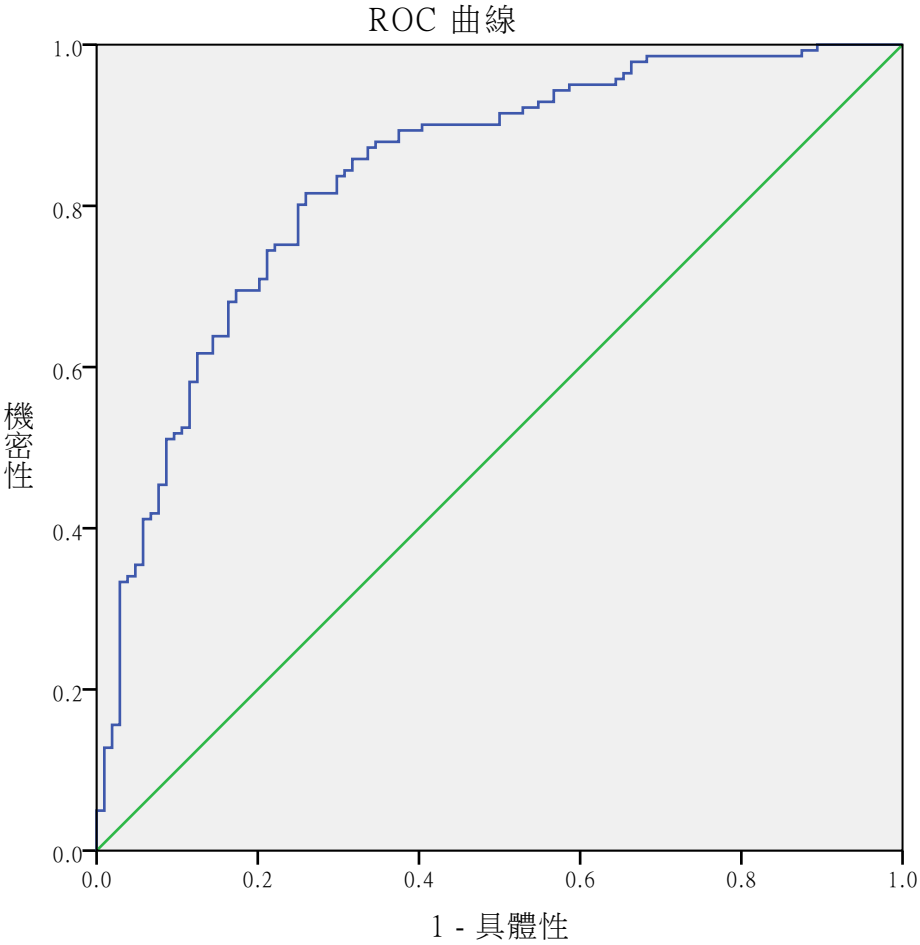

Supplement: Supplementary file 5 [file Data_Sheet_1.ZIP › Supplementary Materials S1/ROC/ROC GSE63060 RED AD-CTL LH .pdf]

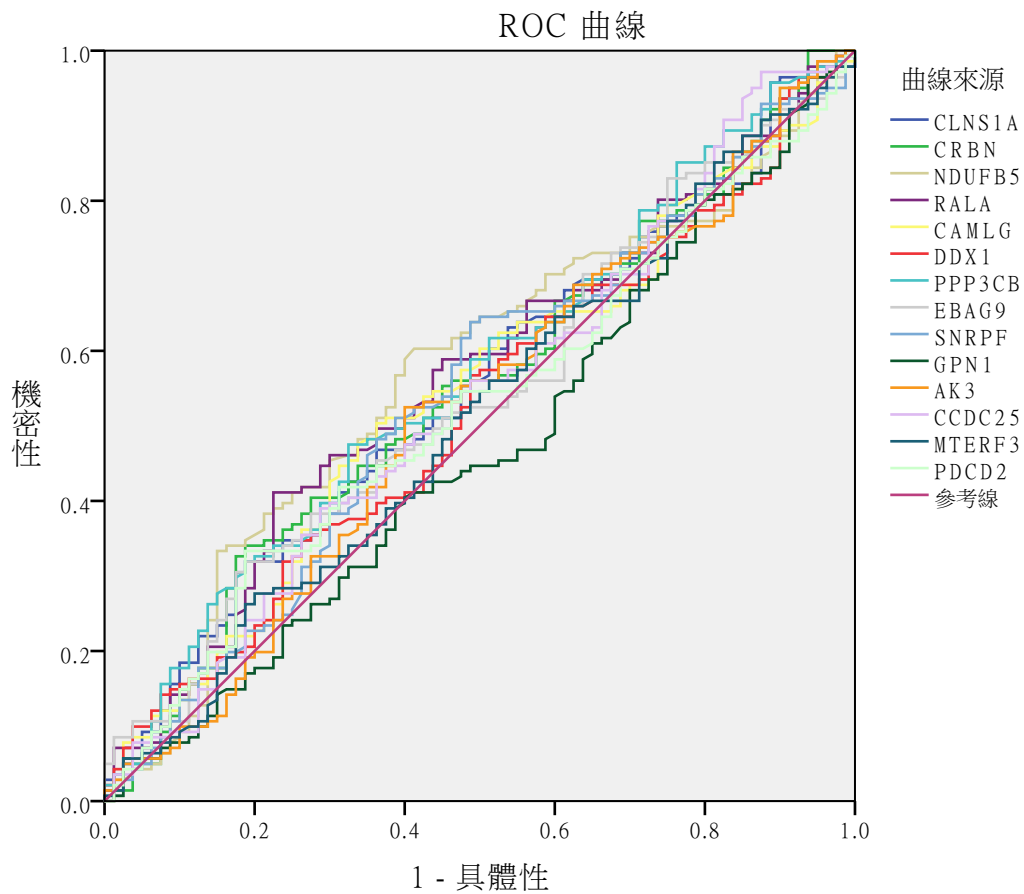

同分產生的對數區段。

Supplement: Supplementary file 5 [file Data_Sheet_1.ZIP › Supplementary Materials S1/ROC/ROC GSE63060 RED AD-MCI DG.pdf]

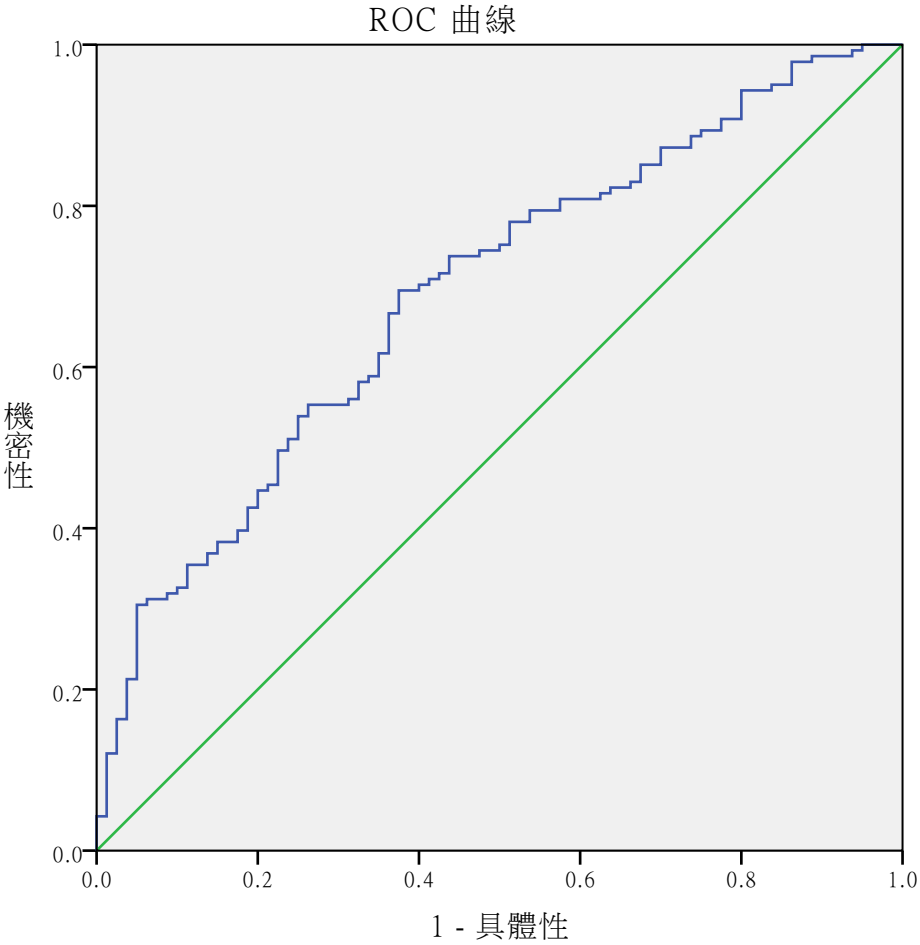

Supplement: Supplementary file 5 [file Data_Sheet_1.ZIP › Supplementary Materials S1/ROC/ROC GSE63060 RED AD-MCI LH.pdf]

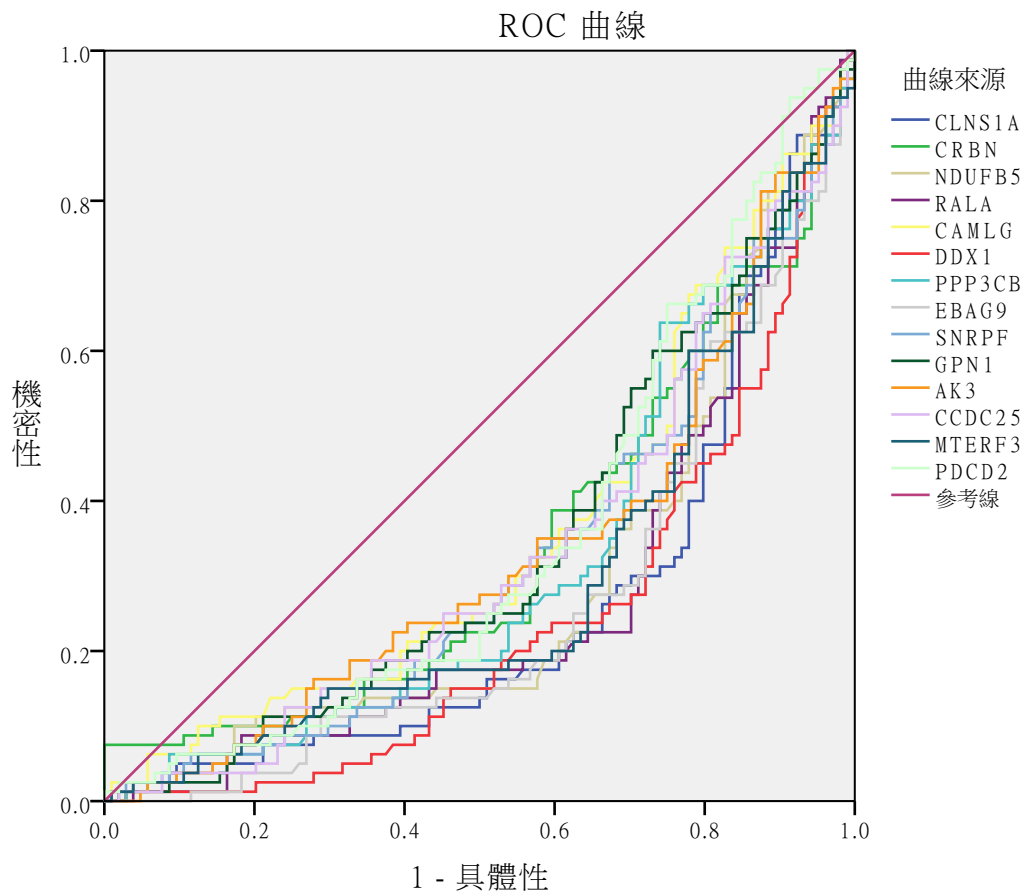

同分產生的對數區段。

Supplement: Supplementary file 5 [file Data_Sheet_1.ZIP › Supplementary Materials S1/ROC/ROC GSE63060 RED MCI-CTL DDG.pdf]

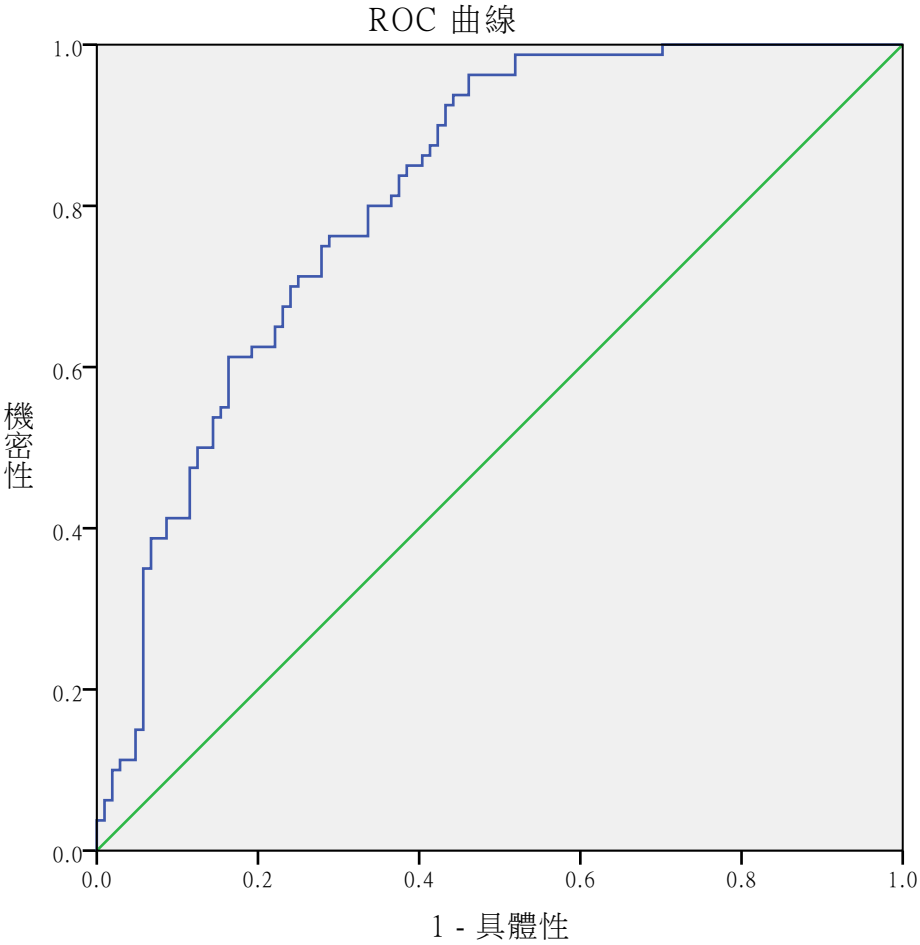

Supplement: Supplementary file 5 [file Data_Sheet_1.ZIP › Supplementary Materials S1/ROC/ROC GSE63060 RED MCI-CTL LH.pdf]

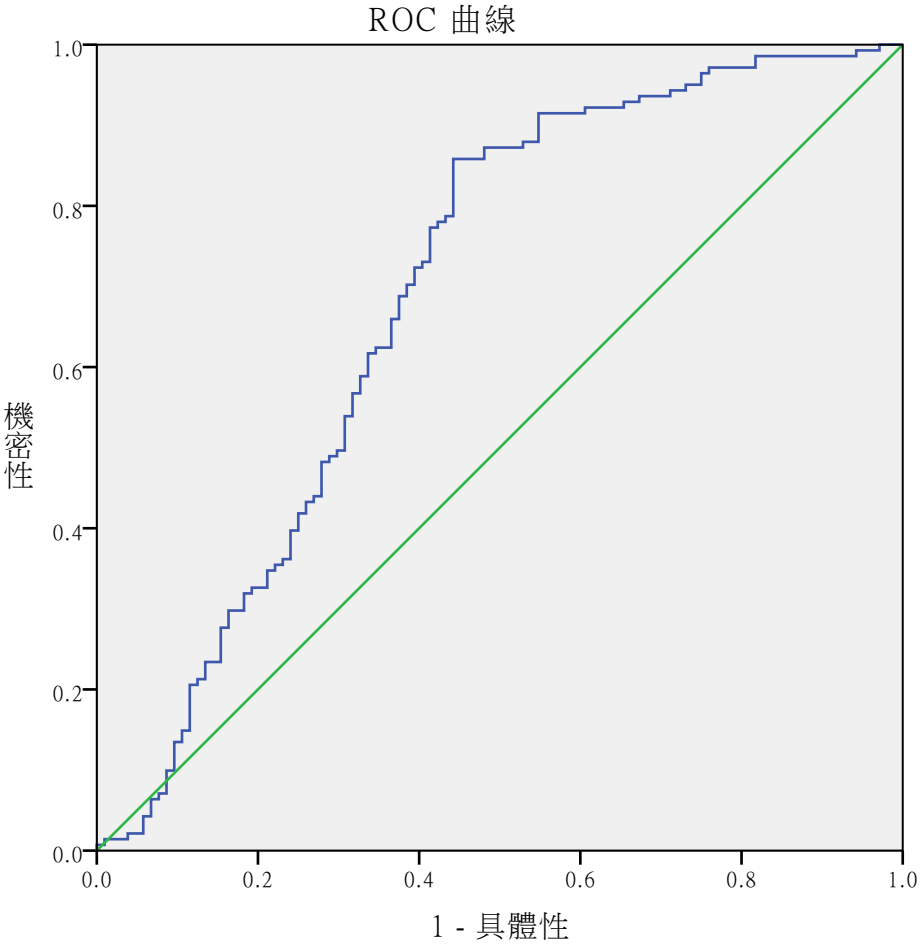

Supplement: Supplementary file 5 [file Data_Sheet_1.ZIP › Supplementary Materials S1/ROC/ROC GSE63060 TURQUIOSE AD-CTL LH.pdf]

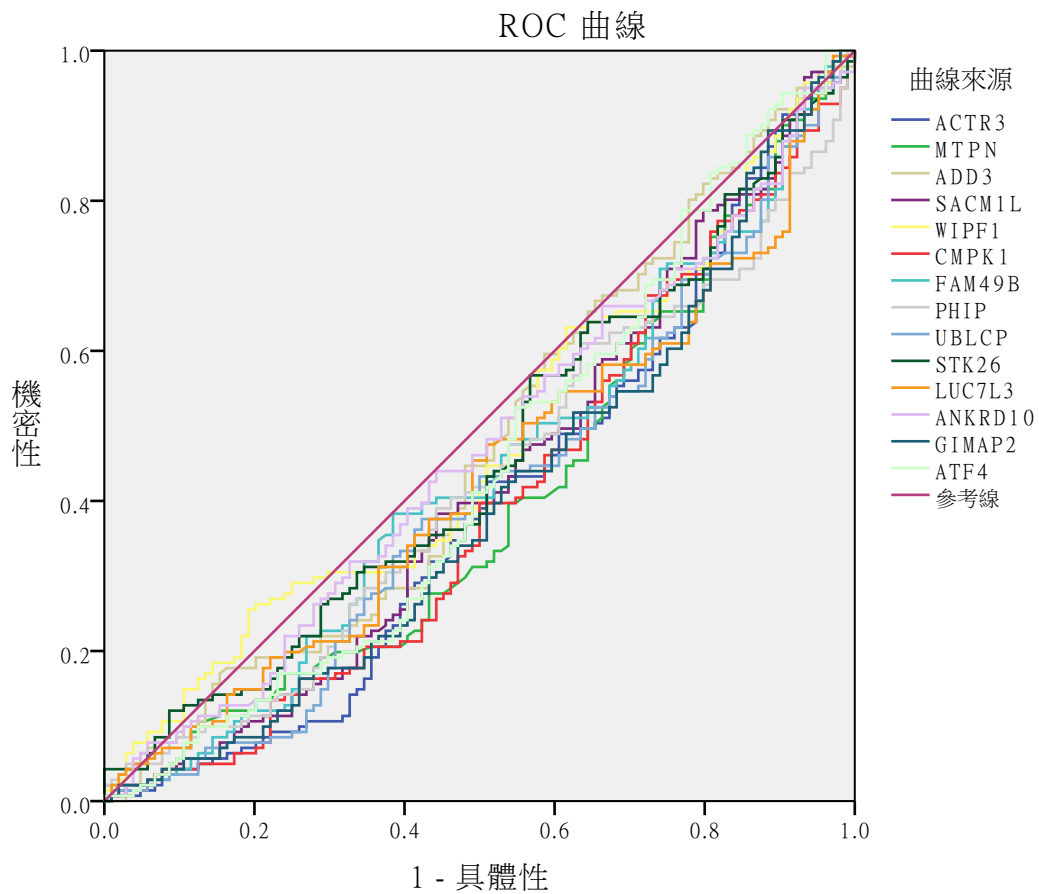

同分產生的對數區段。

Supplement: Supplementary file 5 [file Data_Sheet_1.ZIP › Supplementary Materials S1/ROC/ROC GSE63060 TURQUIOSE AD-CTLDG.pdf]

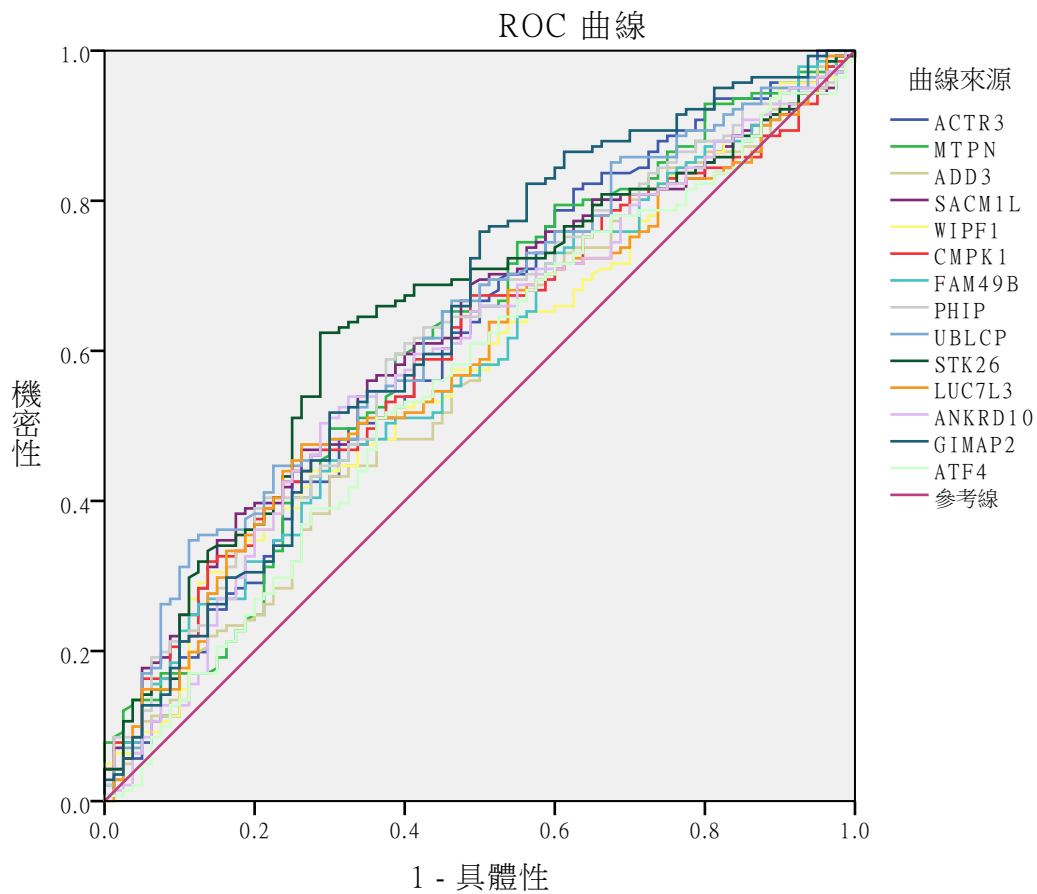

同分產生的對數區段。

Supplement: Supplementary file 5 [file Data_Sheet_1.ZIP › Supplementary Materials S1/ROC/ROC GSE63060 TURQUIOSE AD-MCI DG.pdf]

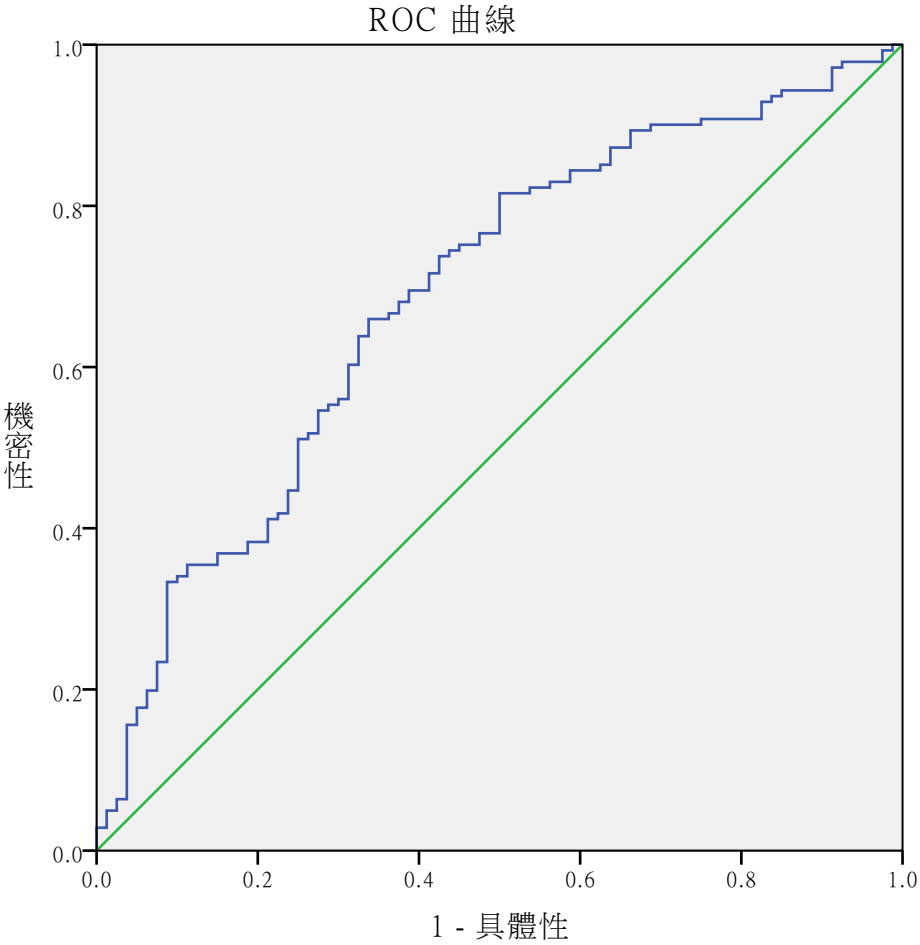

Supplement: Supplementary file 5 [file Data_Sheet_1.ZIP › Supplementary Materials S1/ROC/ROC GSE63060 TURQUIOSE AD-MCI LH.pdf]

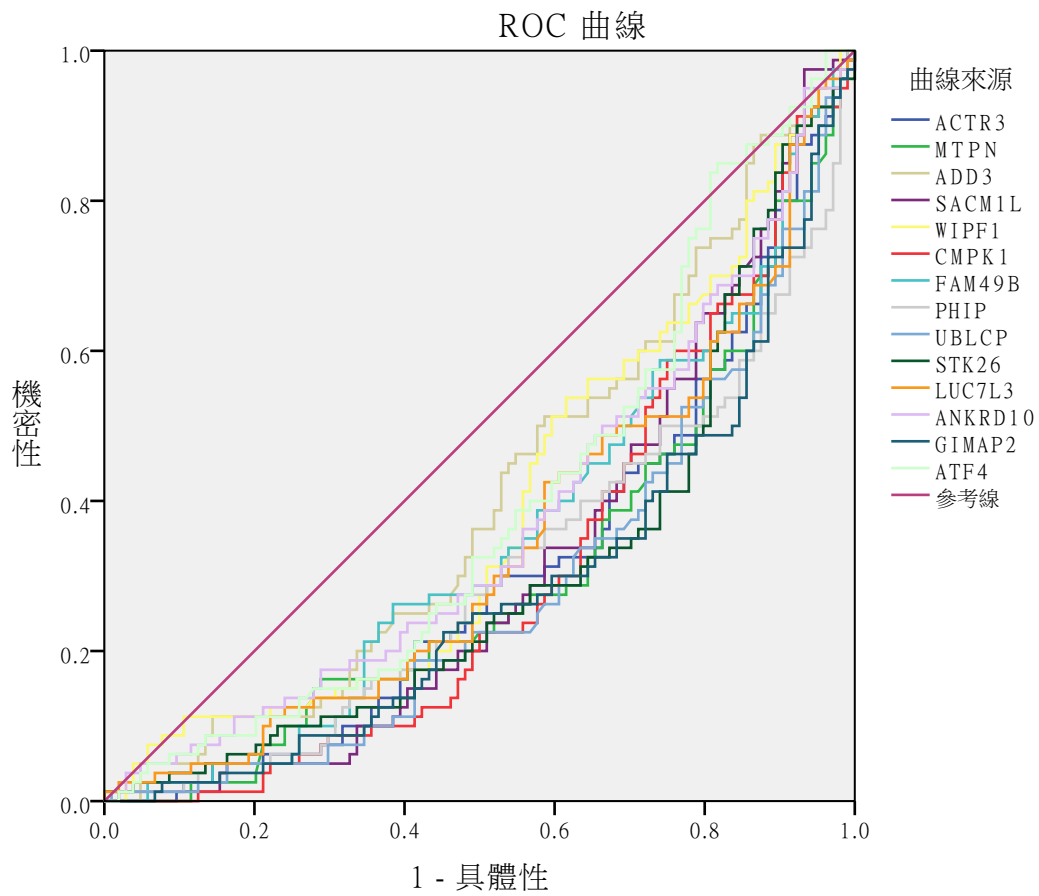

同分產生的對數區段。

Supplement: Supplementary file 5 [file Data_Sheet_1.ZIP › Supplementary Materials S1/ROC/ROC GSE63060 TURQUIOSE MCI-CTL DG.pdf]

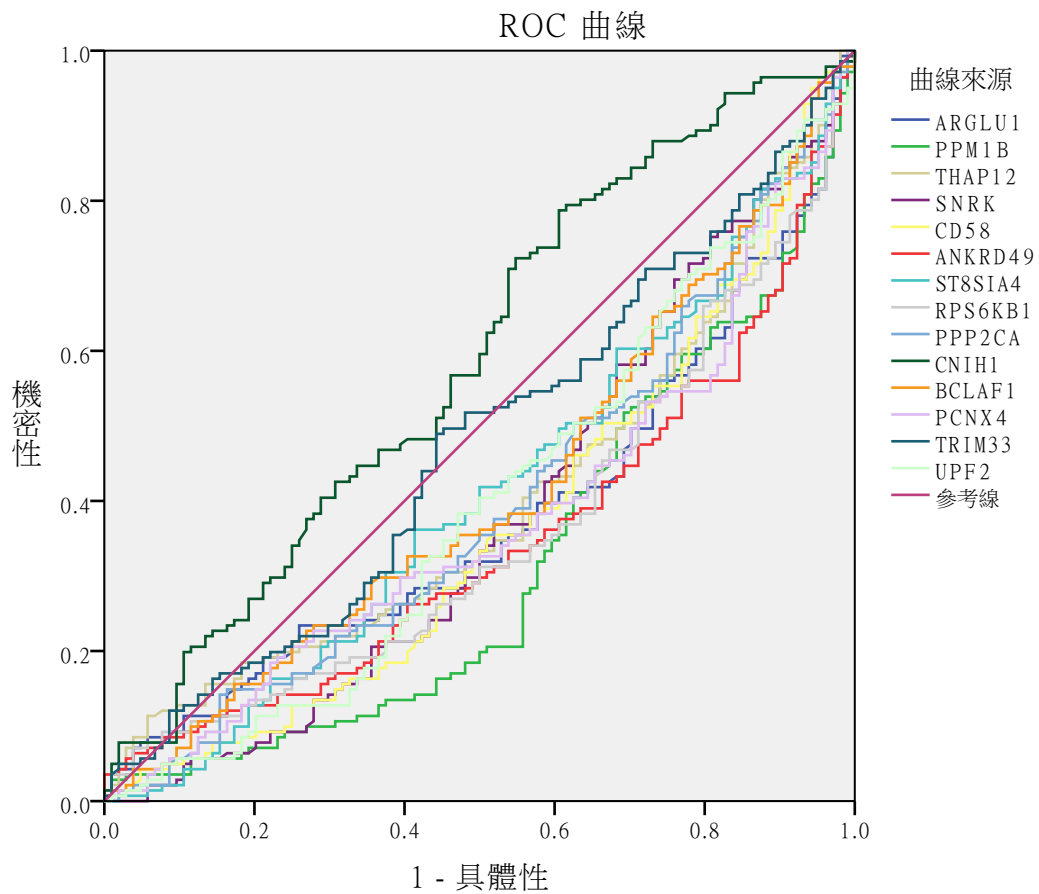

同分產生的對數區段。

Supplement: Supplementary file 5 [file Data_Sheet_1.ZIP › Supplementary Materials S1/ROC/ROC GSE63060 YELLOW AD-CTL DG.pdf]

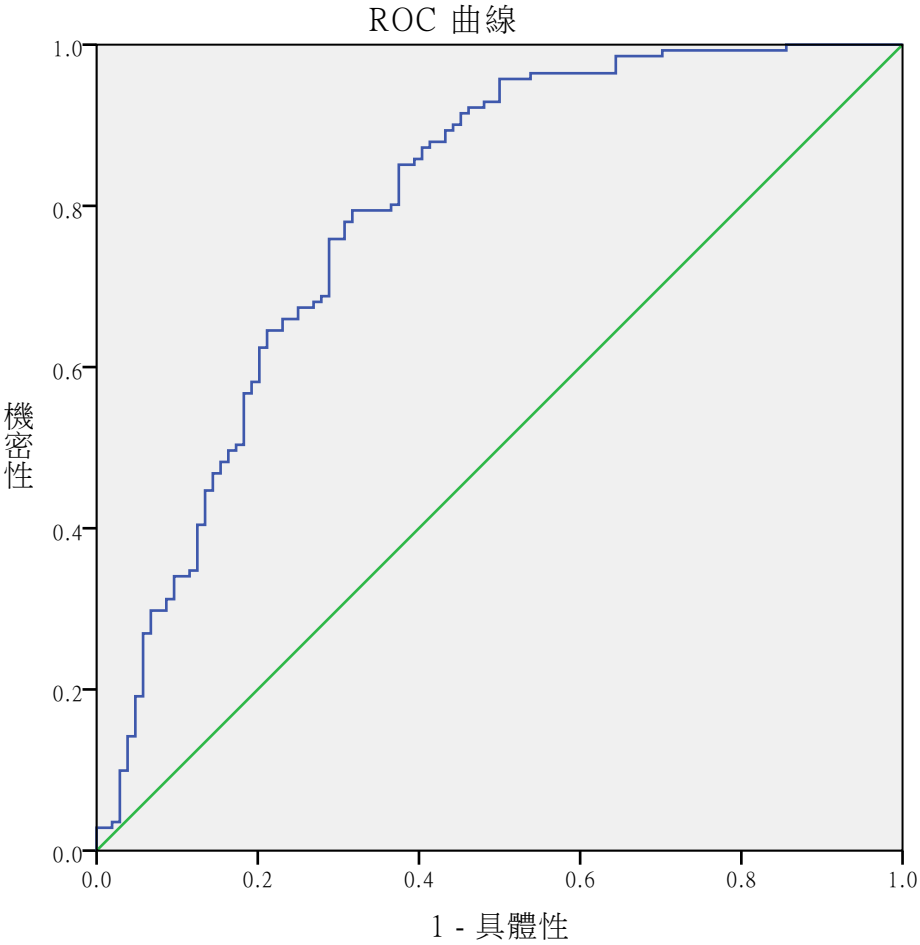

Supplement: Supplementary file 5 [file Data_Sheet_1.ZIP › Supplementary Materials S1/ROC/ROC GSE63060 YELLOW AD-CTL LH.pdf]

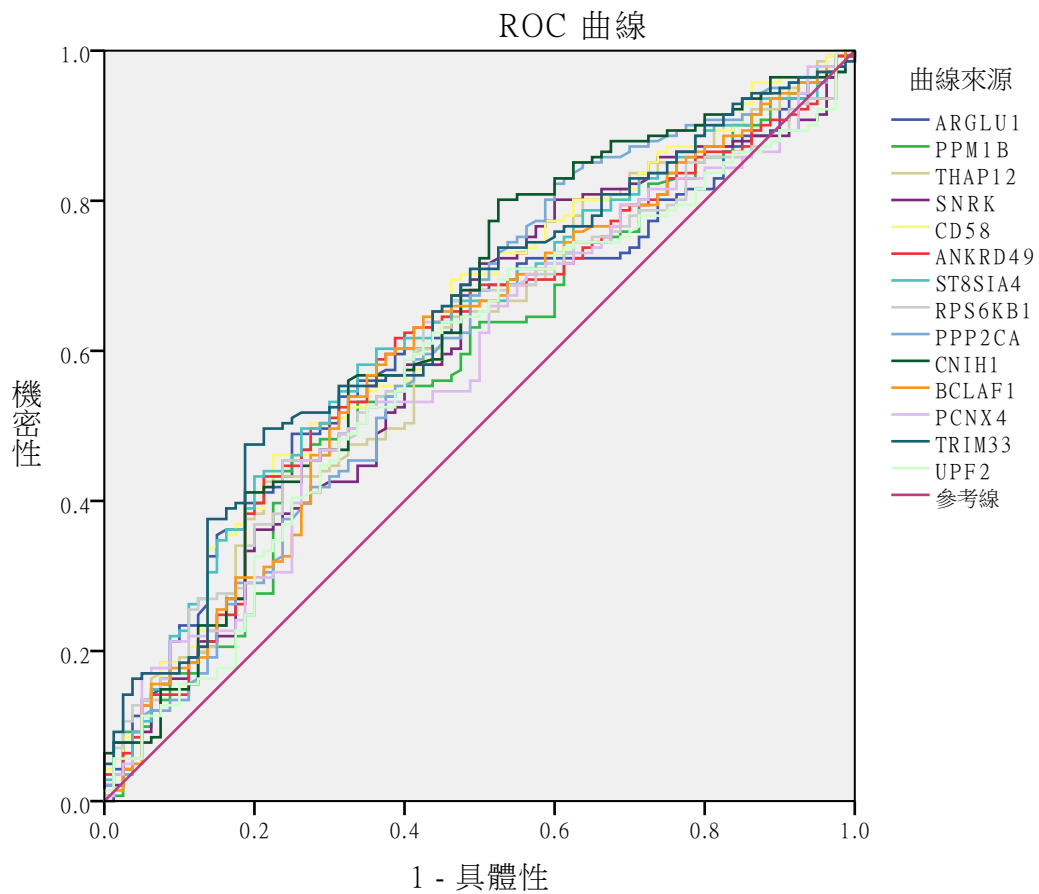

同分產生的對數區段。

Supplement: Supplementary file 5 [file Data_Sheet_1.ZIP › Supplementary Materials S1/ROC/ROC GSE63060 YELLOW AD-MCI DG.pdf]

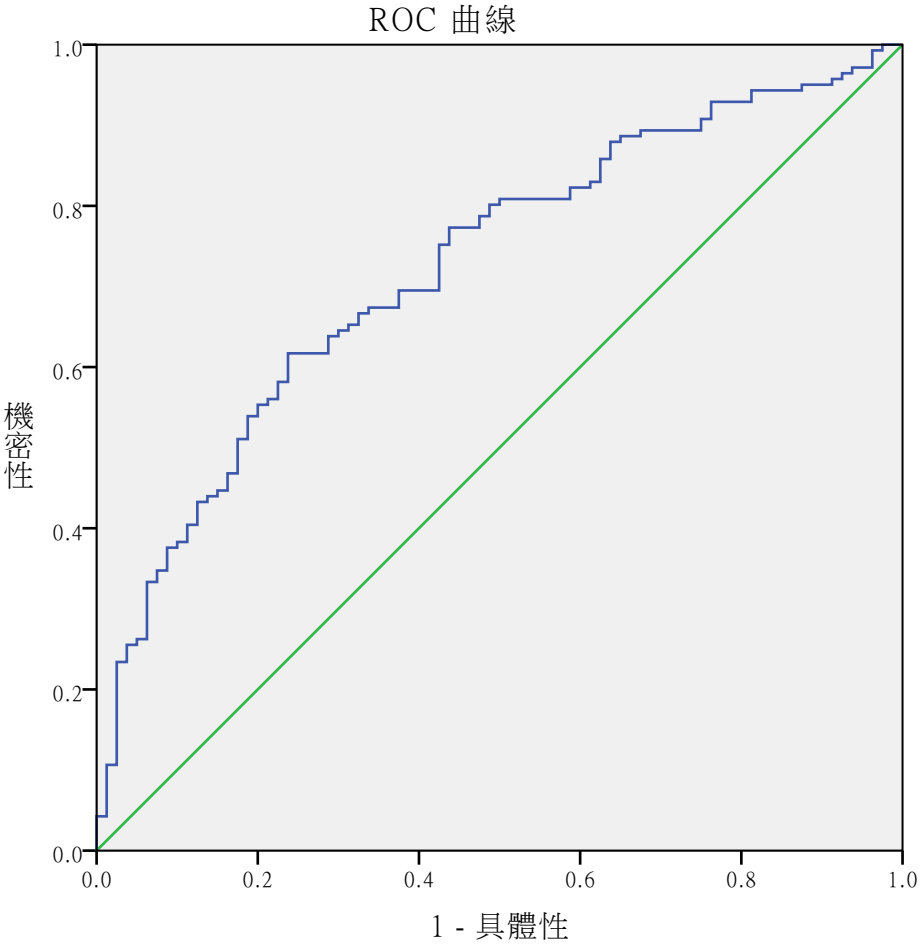

Supplement: Supplementary file 5 [file Data_Sheet_1.ZIP › Supplementary Materials S1/ROC/ROC GSE63060 YELLOW AD-MCI LH.pdf]

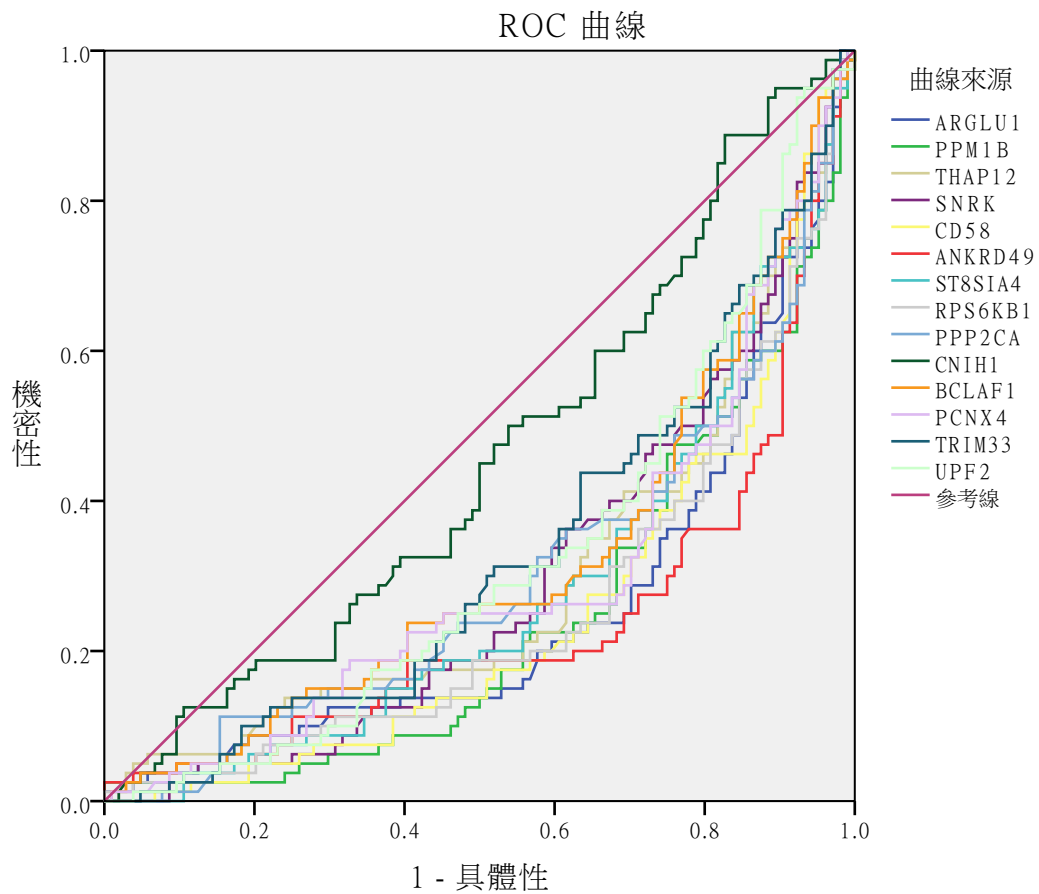

同分產生的對數區段。

Supplement: Supplementary file 5 [file Data_Sheet_1.ZIP › Supplementary Materials S1/ROC/ROC GSE63060 YELLOW MCI-CTL DG.pdf]

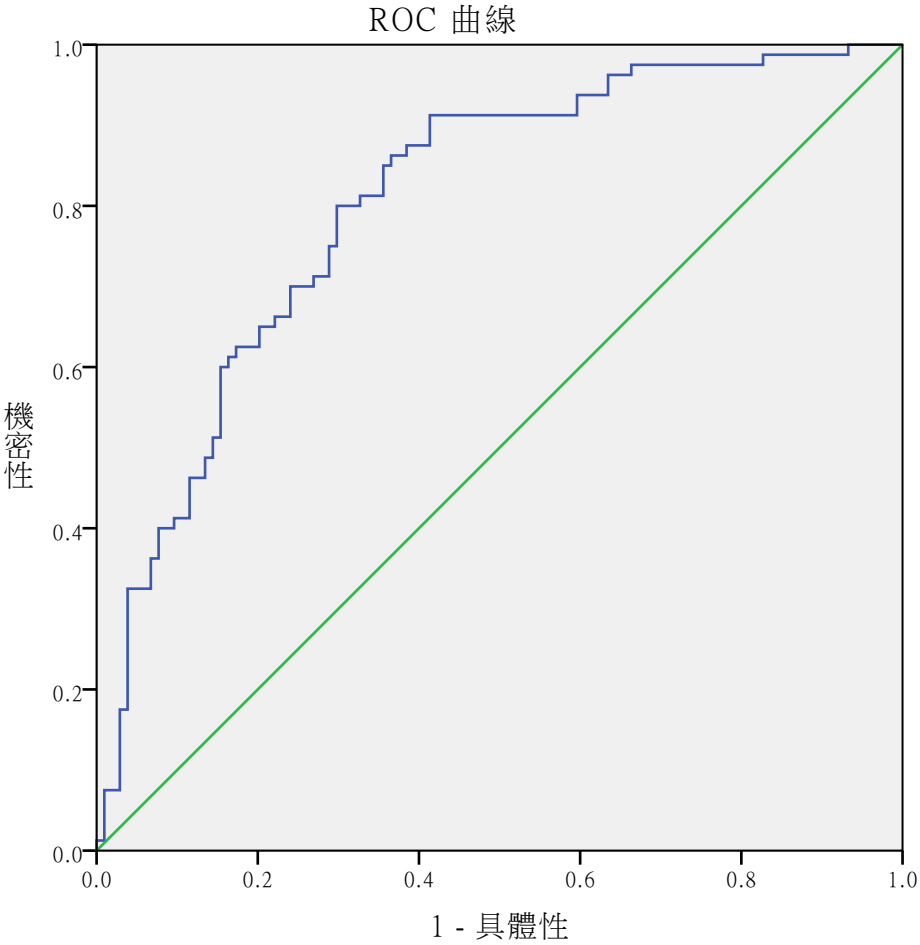

Supplement: Supplementary file 5 [file Data_Sheet_1.ZIP › Supplementary Materials S1/ROC/ROC GSE63060 YELLOW MCI-CTL LH.pdf]

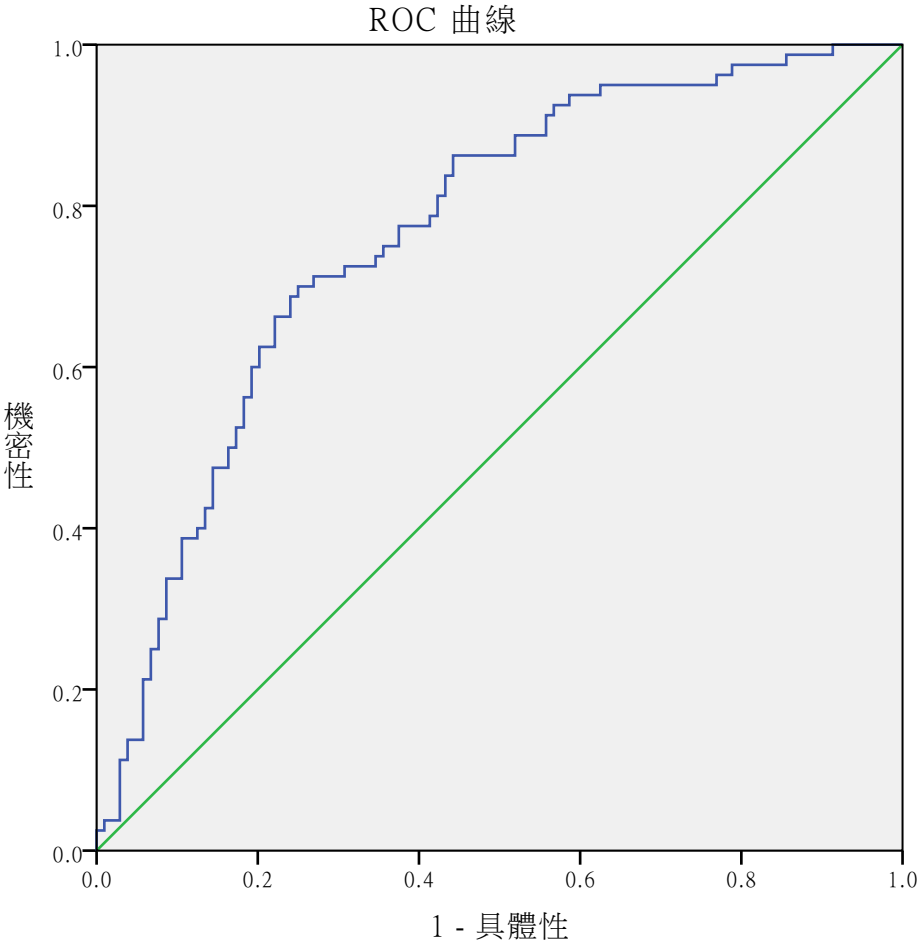

Supplement: Supplementary file 5 [file Data_Sheet_1.ZIP › Supplementary Materials S1/ROC/ROC GSE63060TURQUIOSE MCI-CTL LH.pdf]

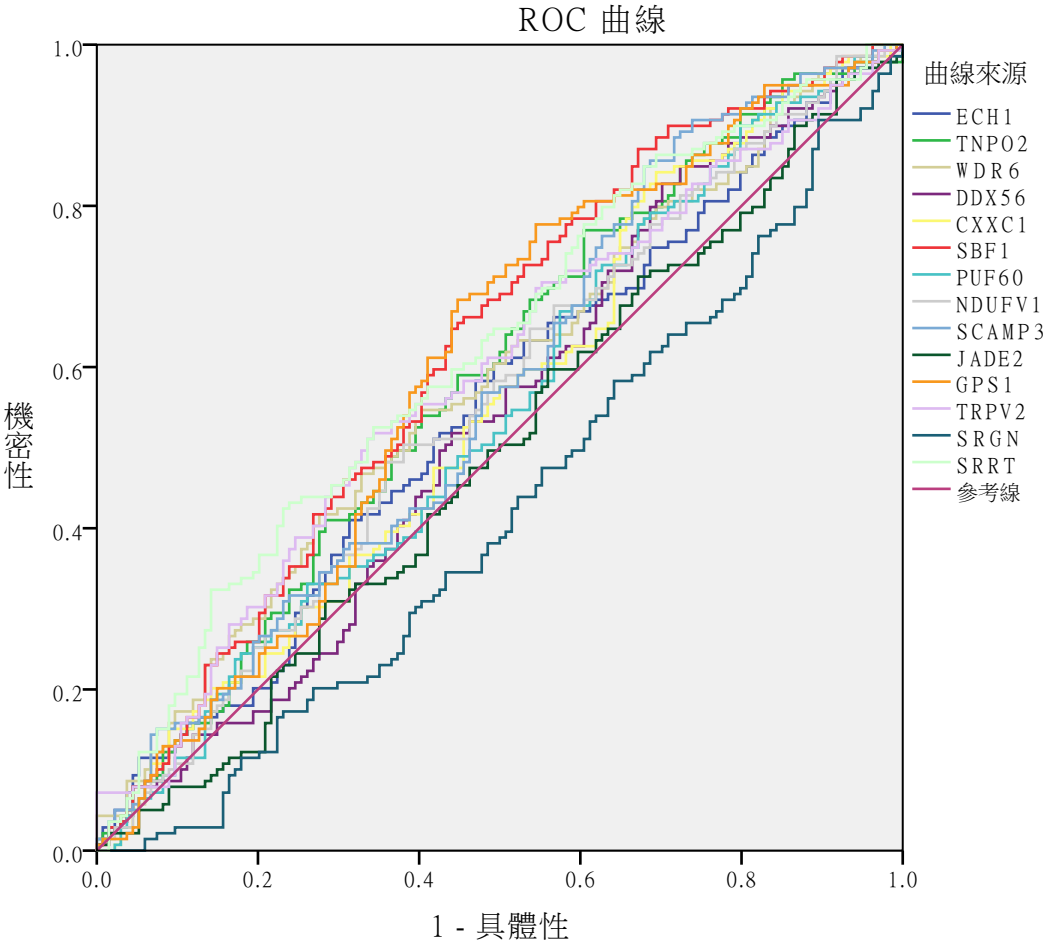

Supplement: Supplementary file 5 [file Data_Sheet_1.ZIP › Supplementary Materials S1/ROC/ROC GSE63061 BLACK AD-CTL DG.pdf]

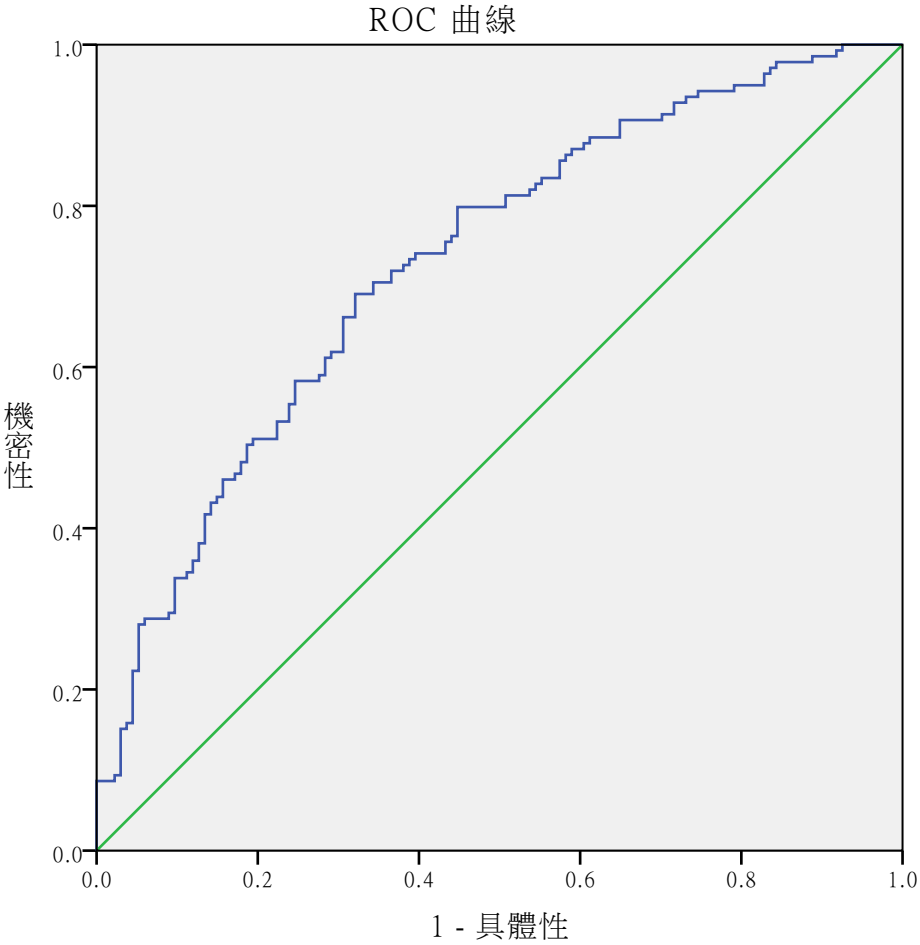

Supplement: Supplementary file 5 [file Data_Sheet_1.ZIP › Supplementary Materials S1/ROC/ROC GSE63061 BLACK AD-CTL LH.pdf]

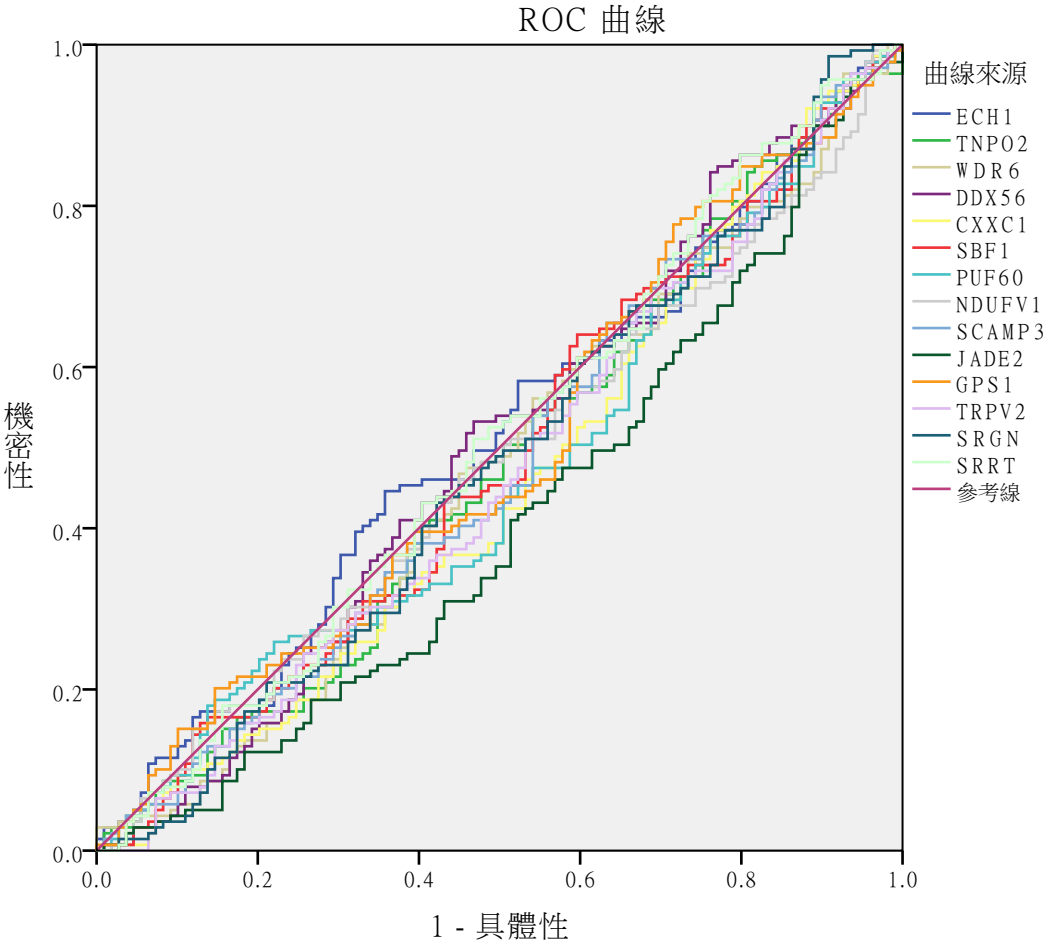

Supplement: Supplementary file 5 [file Data_Sheet_1.ZIP › Supplementary Materials S1/ROC/ROC GSE63061 BLACK AD-MCI DG .pdf]

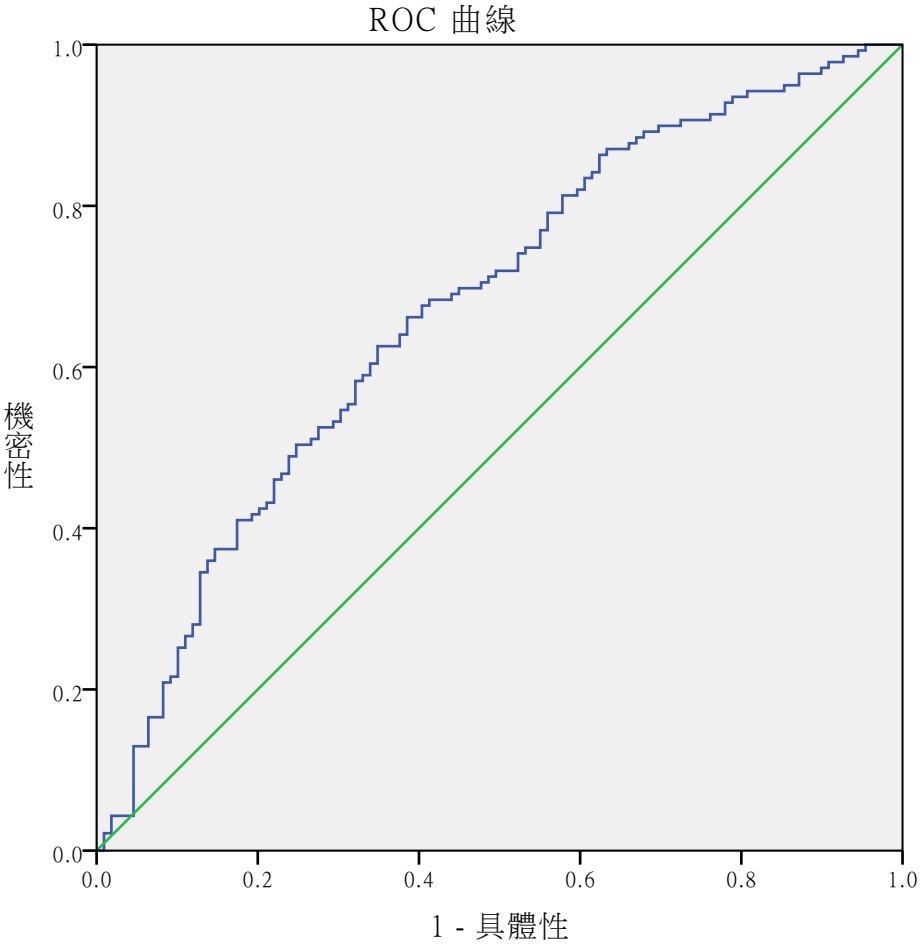

Supplement: Supplementary file 5 [file Data_Sheet_1.ZIP › Supplementary Materials S1/ROC/ROC GSE63061 BLACK AD-MCI LH.pdf]

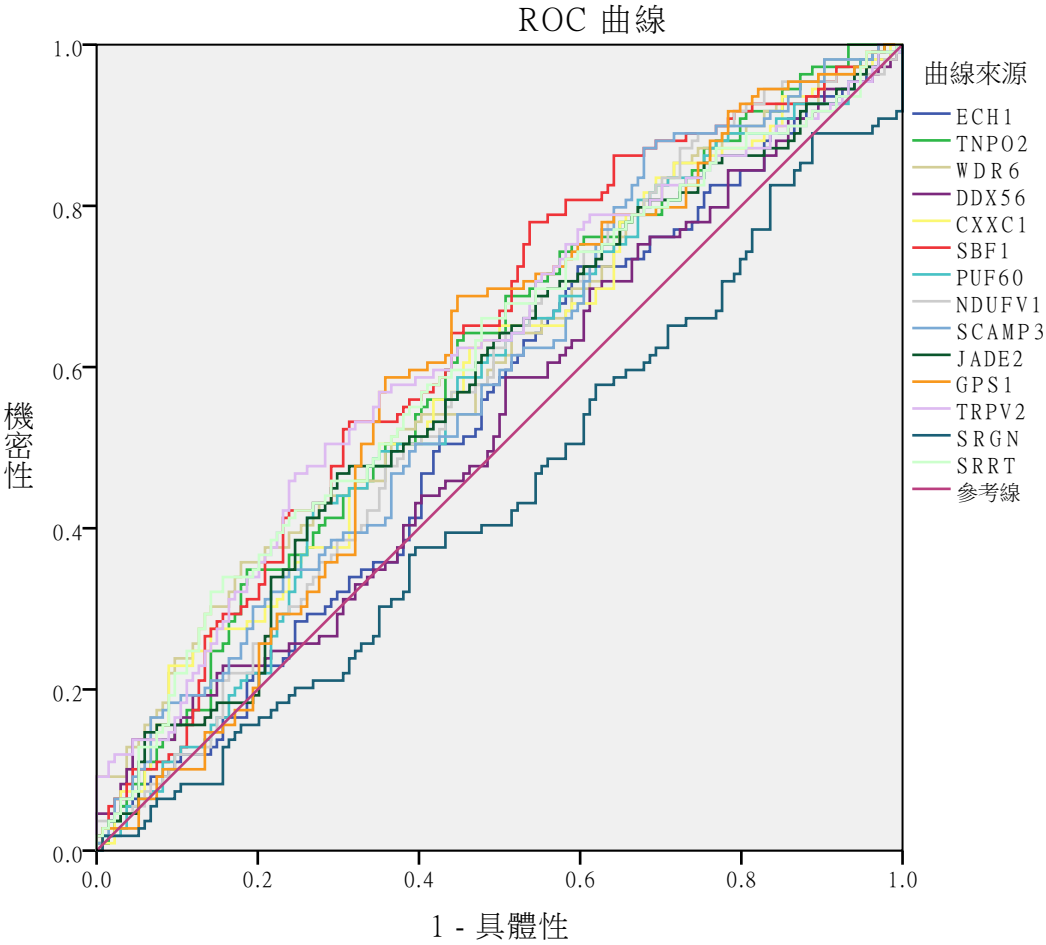

Supplement: Supplementary file 5 [file Data_Sheet_1.ZIP › Supplementary Materials S1/ROC/ROC GSE63061 BLACK MCI-CTL DG pdf.pdf]

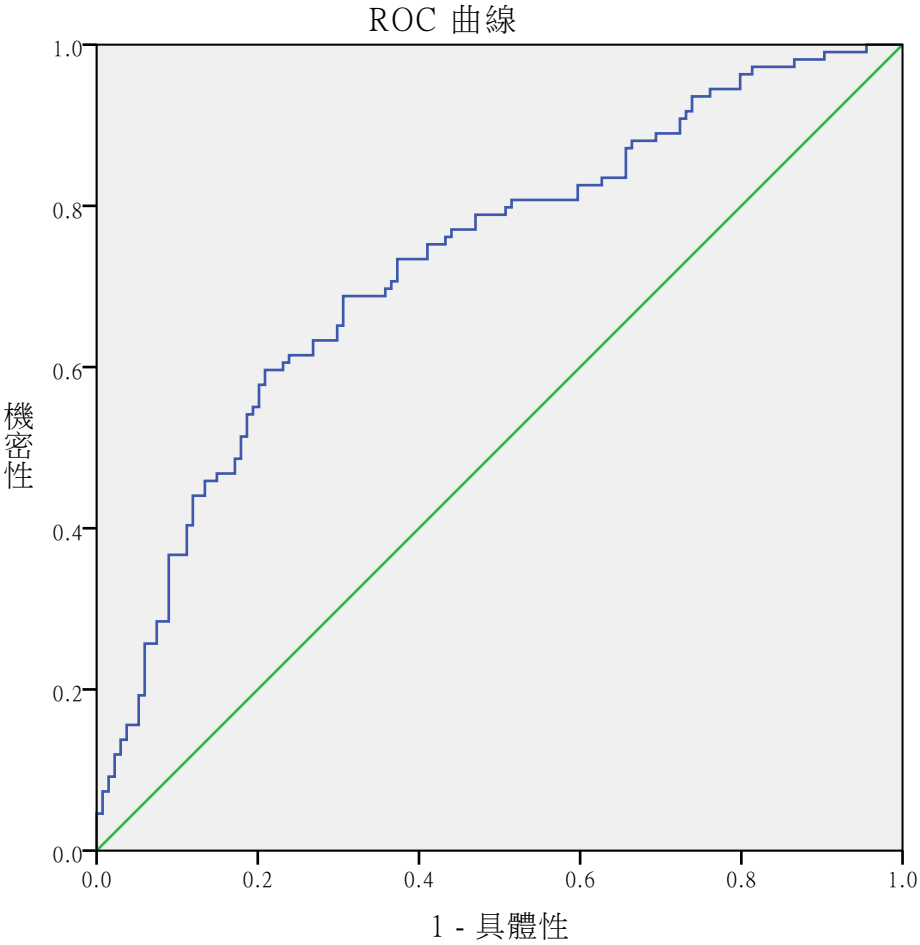

Supplement: Supplementary file 5 [file Data_Sheet_1.ZIP › Supplementary Materials S1/ROC/ROC GSE63061 BLACK MCI-CTL LH .pdf]

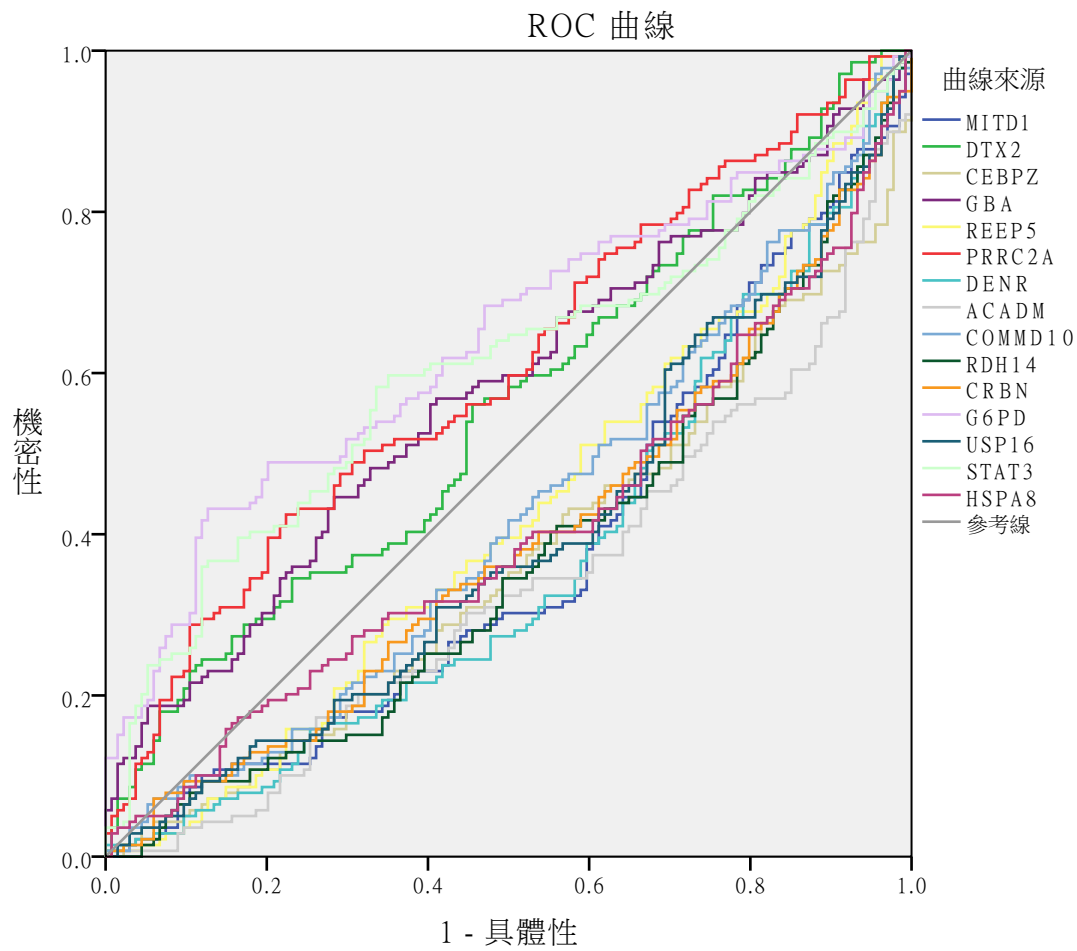

Supplement: Supplementary file 5 [file Data_Sheet_1.ZIP › Supplementary Materials S1/ROC/ROC GSE63061 BLUE AD-CTL DG.pdf]

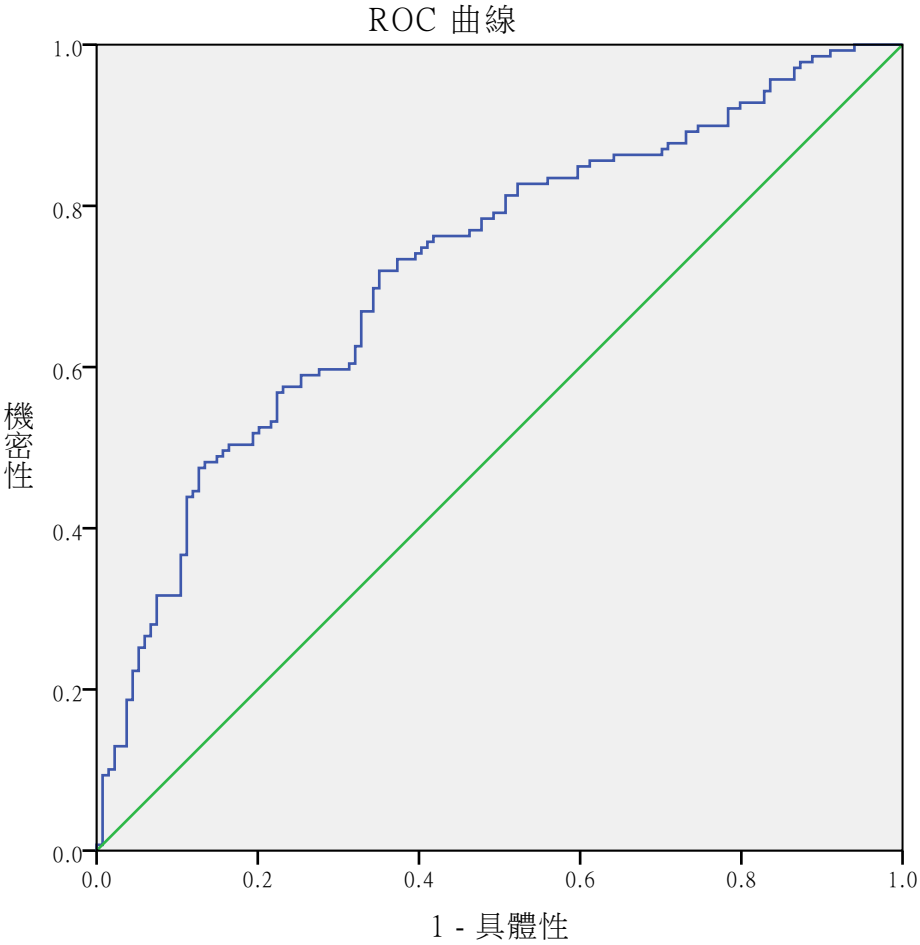

Supplement: Supplementary file 5 [file Data_Sheet_1.ZIP › Supplementary Materials S1/ROC/ROC GSE63061 BLUE AD-CTL LH.pdf]

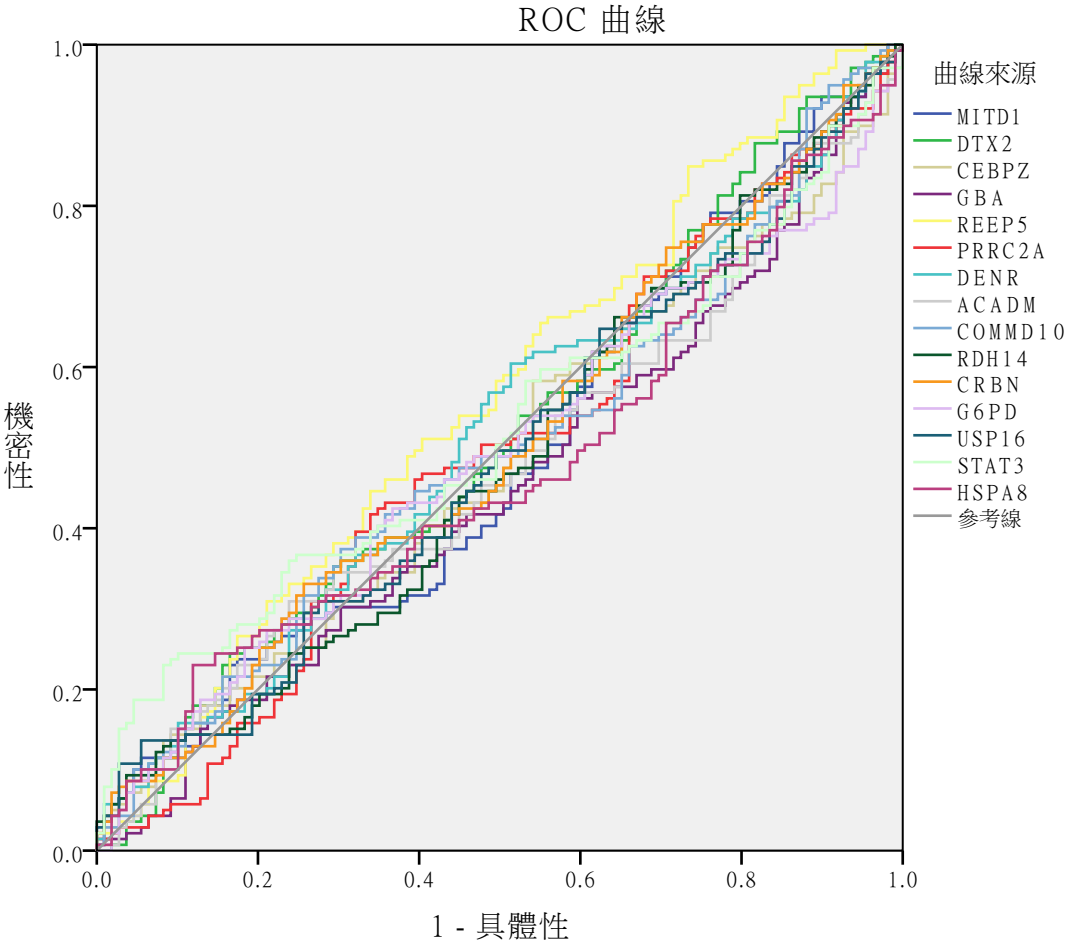

Supplement: Supplementary file 5 [file Data_Sheet_1.ZIP › Supplementary Materials S1/ROC/ROC GSE63061 BLUE AD-MCI DGpdf.pdf]

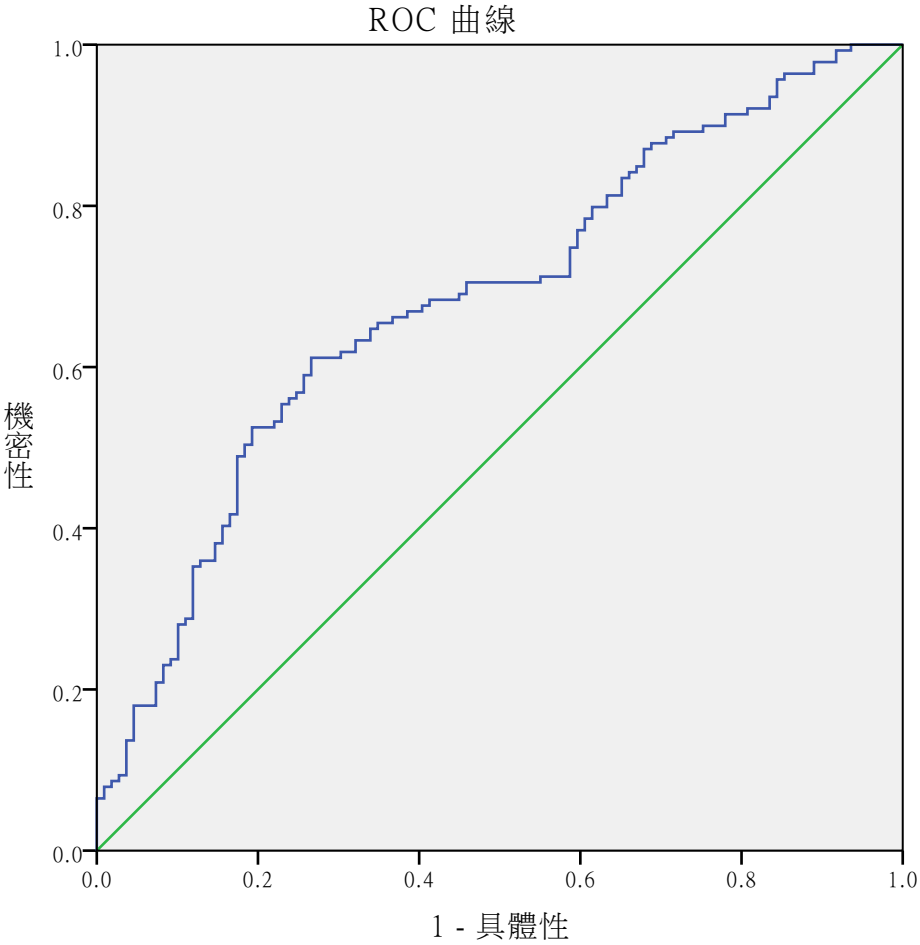

Supplement: Supplementary file 5 [file Data_Sheet_1.ZIP › Supplementary Materials S1/ROC/ROC GSE63061 BLUE AD-MCI LH pdf.pdf]

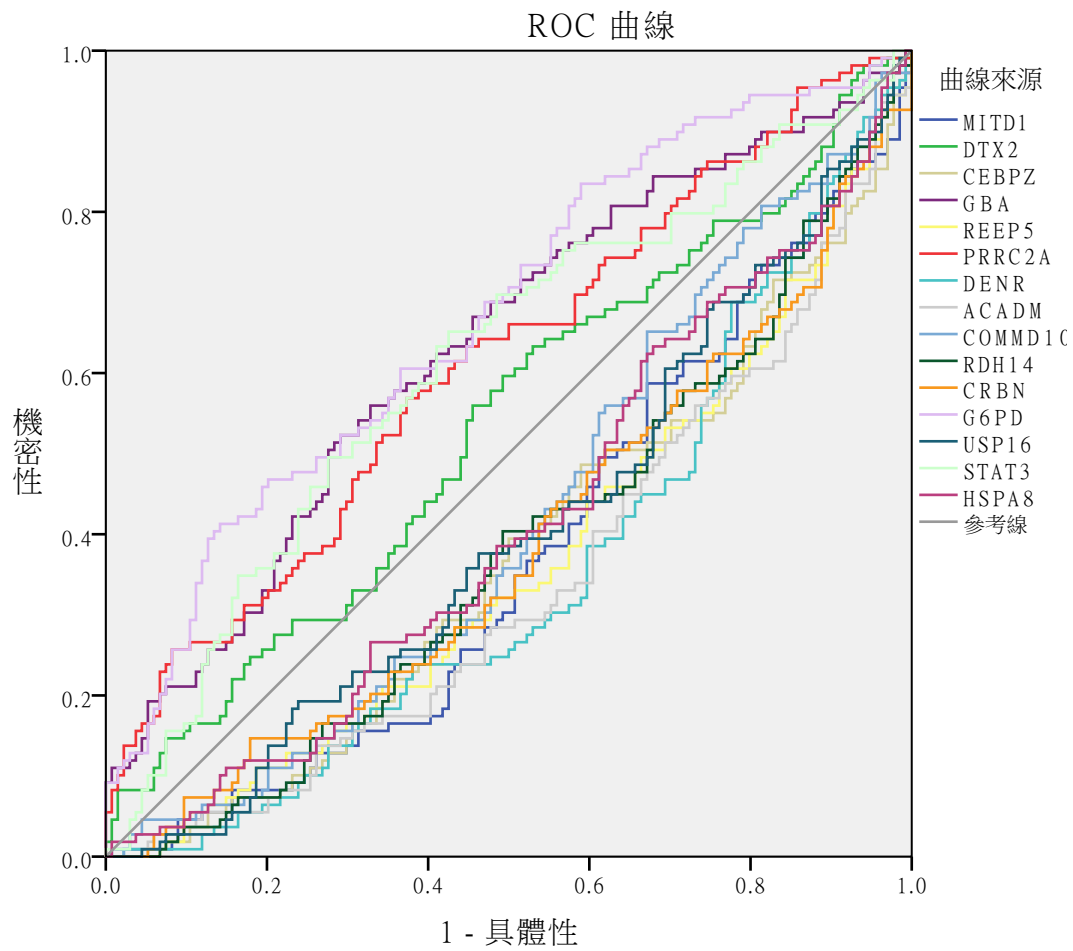

Supplement: Supplementary file 5 [file Data_Sheet_1.ZIP › Supplementary Materials S1/ROC/ROC GSE63061 BLUE MCI-CTL DGpdf.pdf]

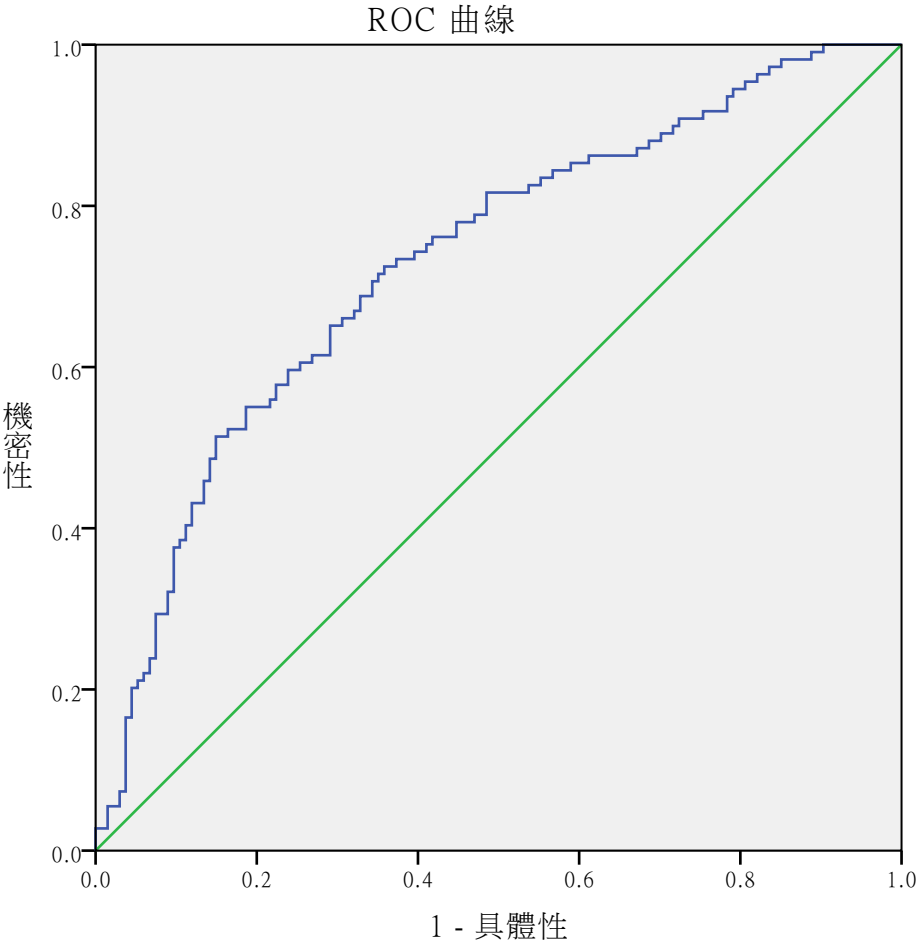

Supplement: Supplementary file 5 [file Data_Sheet_1.ZIP › Supplementary Materials S1/ROC/ROC GSE63061 BLUE MCI-CTL LH .pdf]

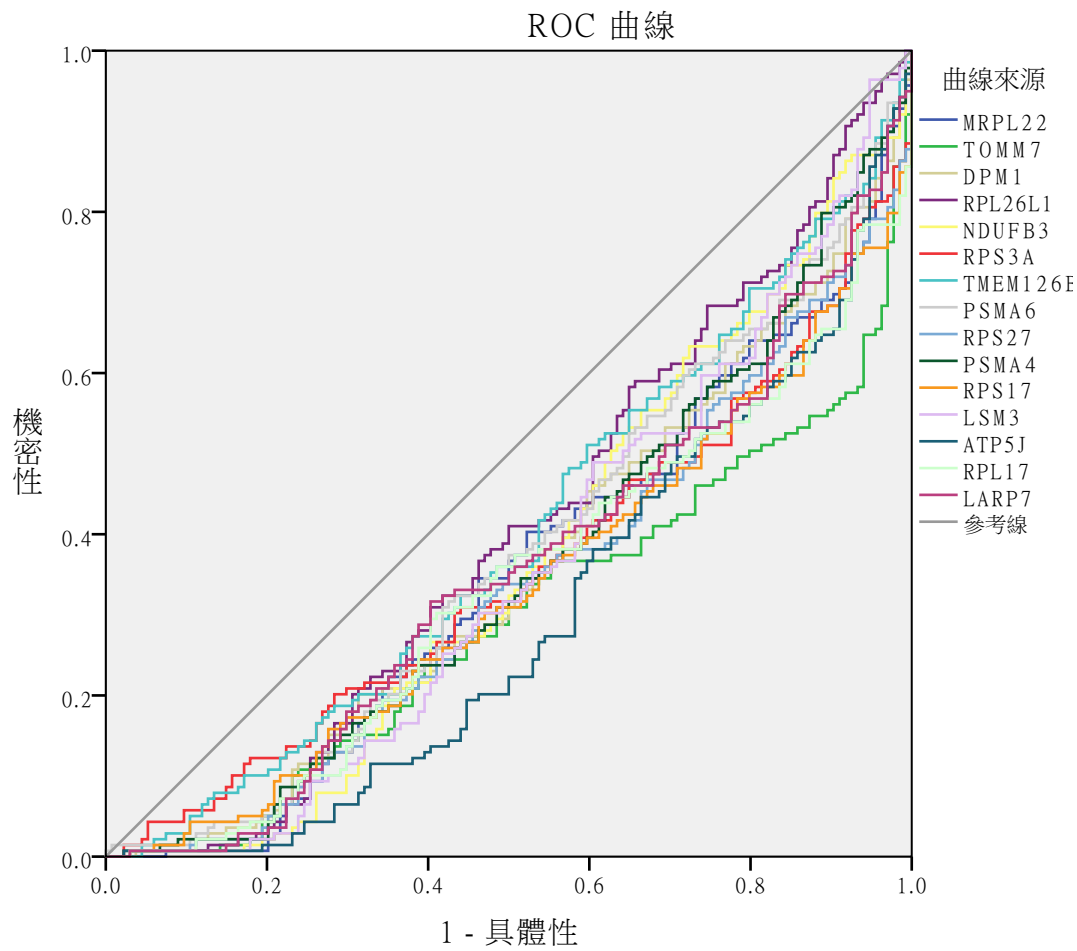

Supplement: Supplementary file 5 [file Data_Sheet_1.ZIP › Supplementary Materials S1/ROC/ROC GSE63061 BROWN AD-CTL DG.pdf]

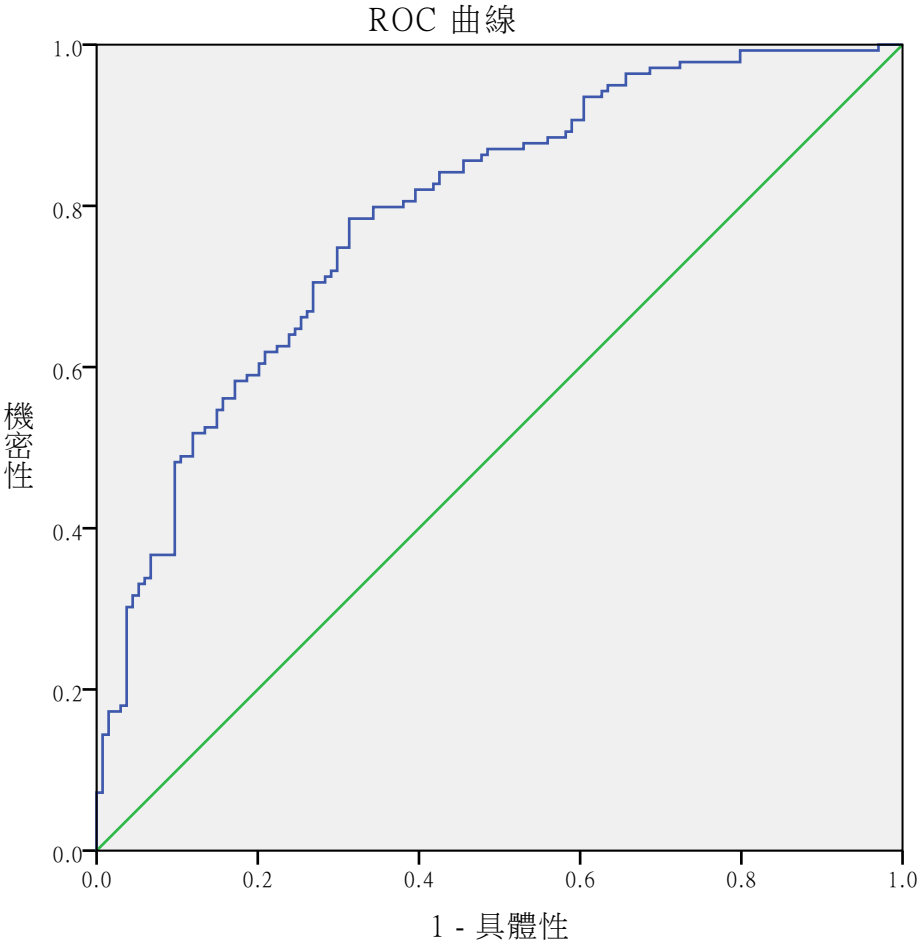

Supplement: Supplementary file 5 [file Data_Sheet_1.ZIP › Supplementary Materials S1/ROC/ROC GSE63061 BROWN AD-CTL LH.pdf]

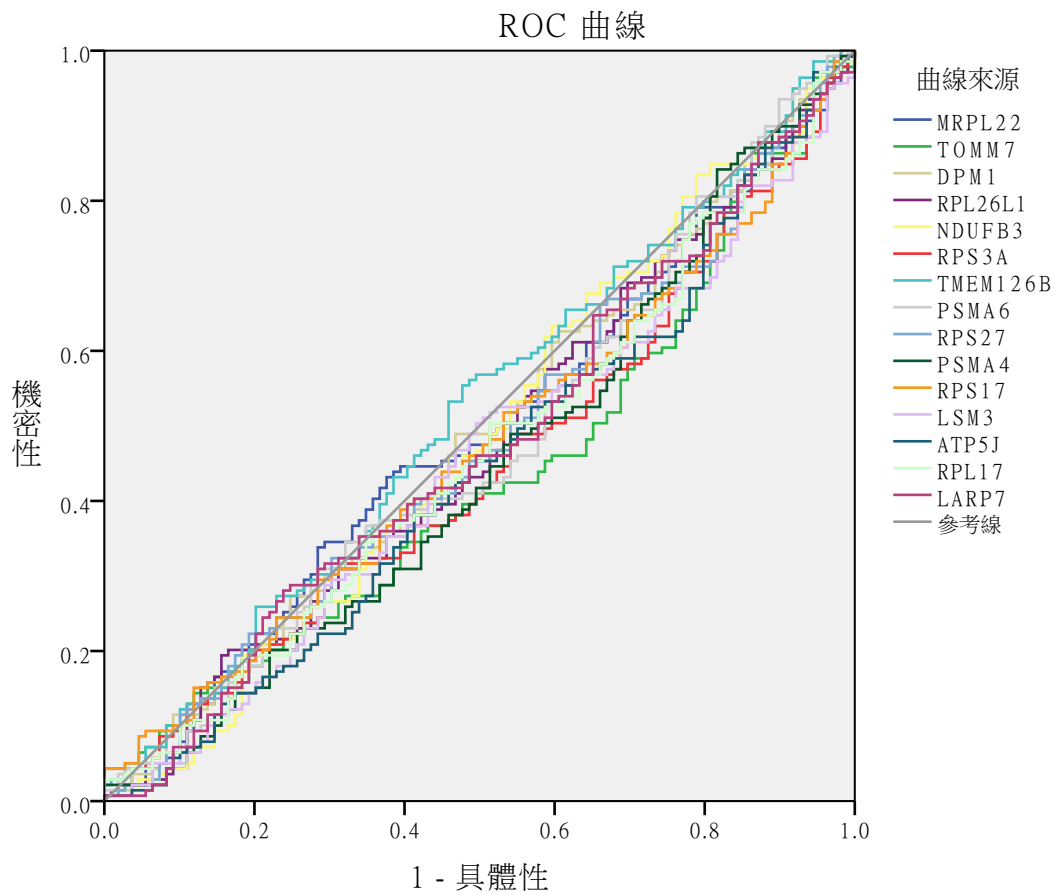

同分產生的對數區段。

Supplement: Supplementary file 5 [file Data_Sheet_1.ZIP › Supplementary Materials S1/ROC/ROC GSE63061 BROWN AD-MCI DG.pdf]

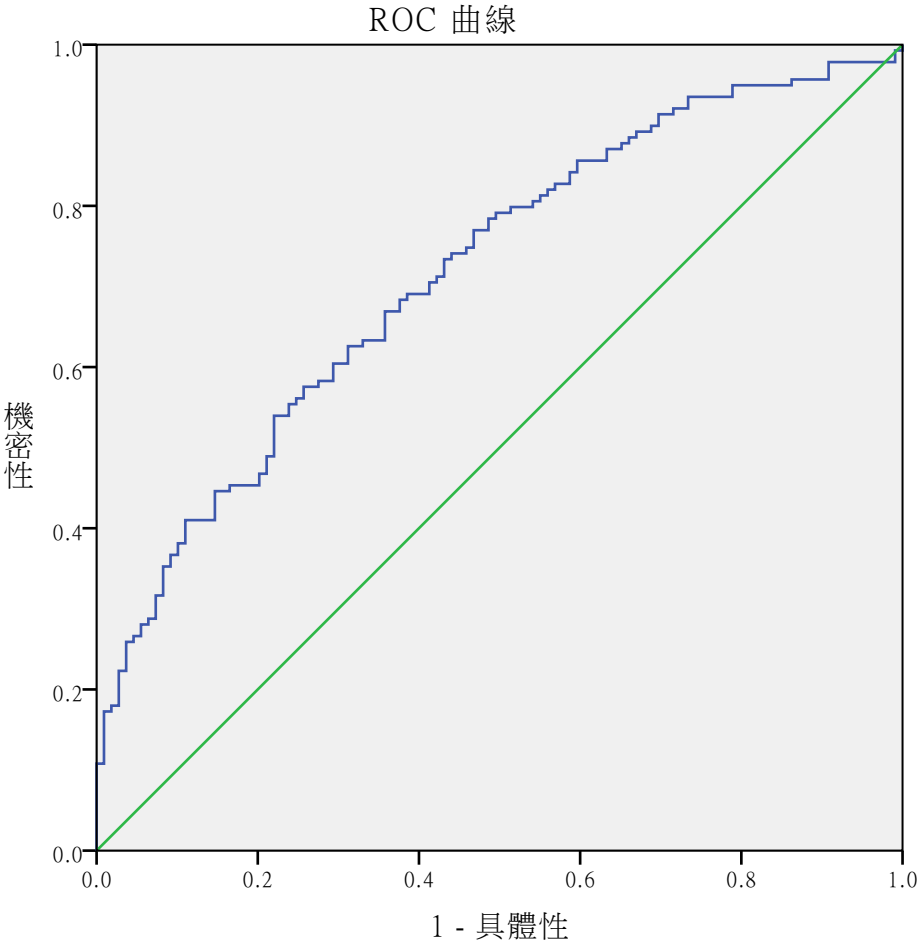

Supplement: Supplementary file 5 [file Data_Sheet_1.ZIP › Supplementary Materials S1/ROC/ROC GSE63061 BROWN AD-MCI LH.pdf]

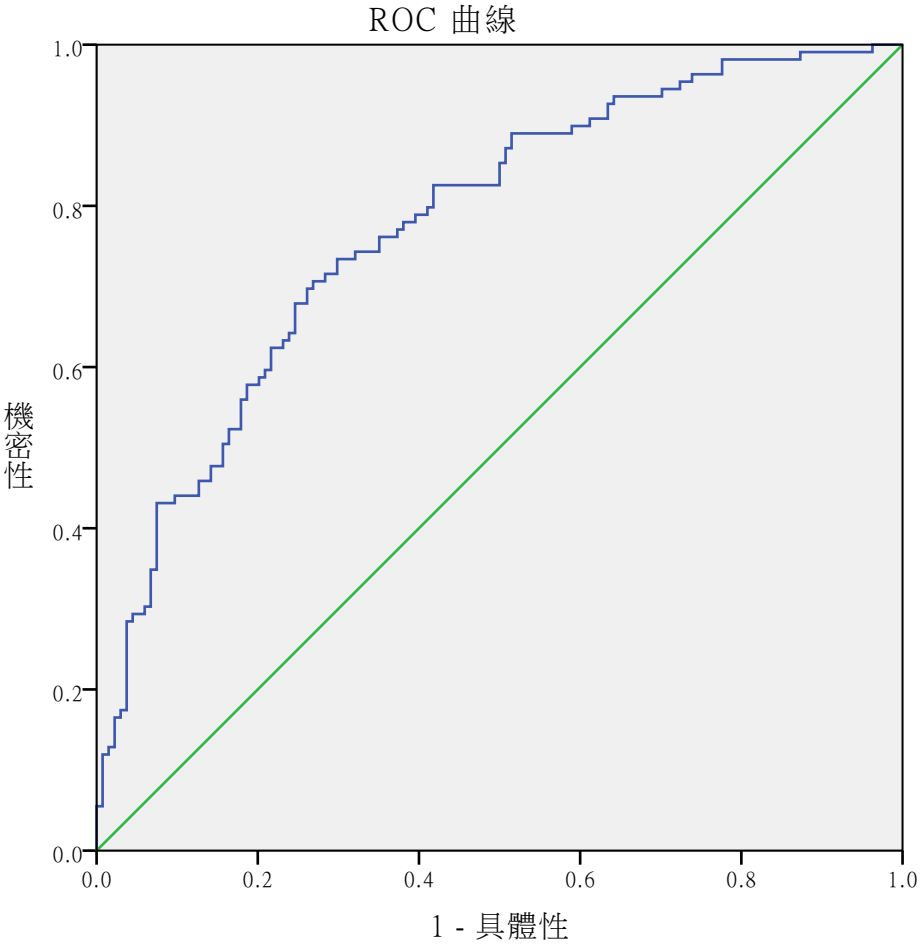

Supplement: Supplementary file 5 [file Data_Sheet_1.ZIP › Supplementary Materials S1/ROC/ROC GSE63061 BROWN MCI-CTL LH.pdf]

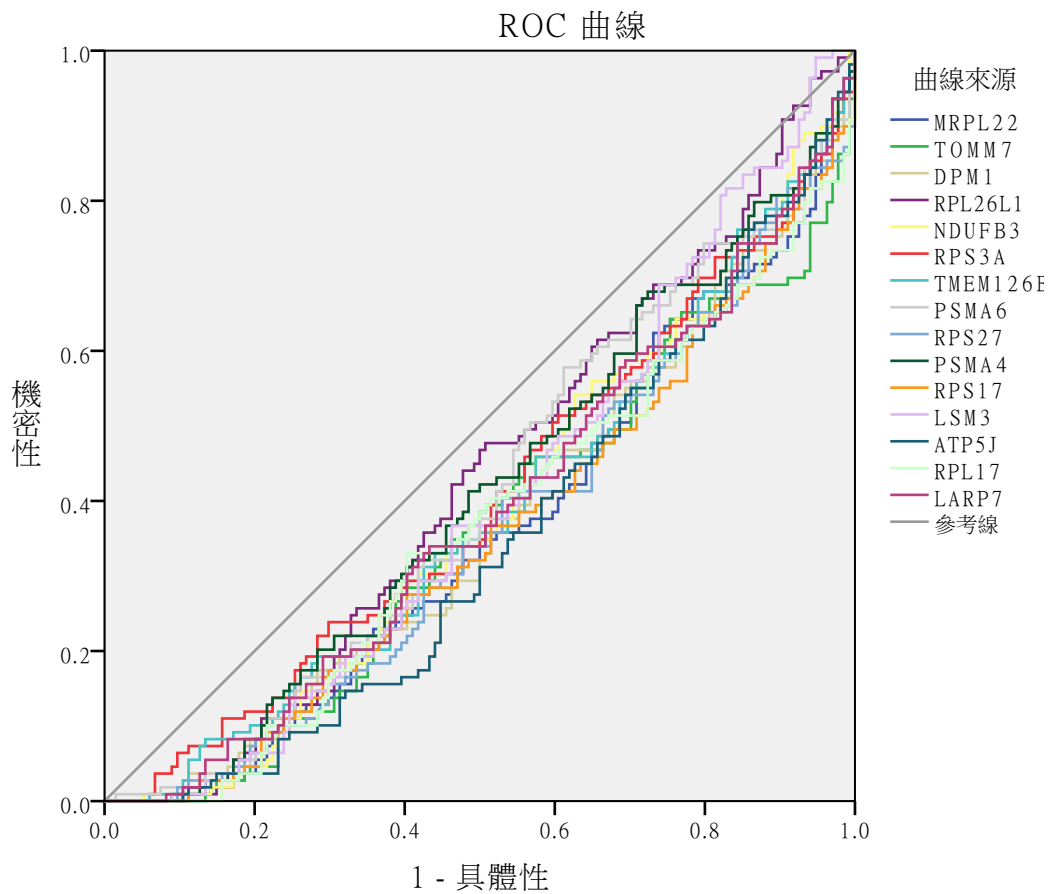

同分產生的對數區段。

Supplement: Supplementary file 5 [file Data_Sheet_1.ZIP › Supplementary Materials S1/ROC/ROC GSE63061 BROWN MCI-CTLDG.pdf]

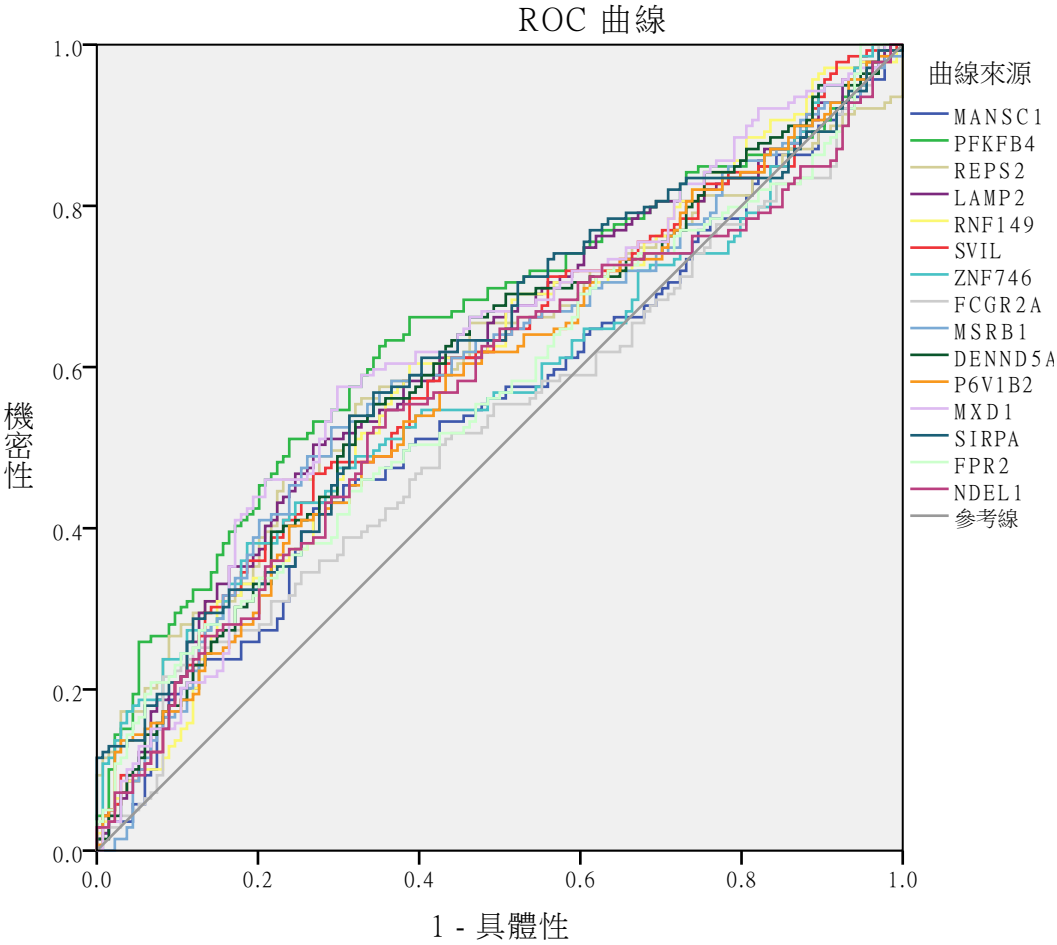

Supplement: Supplementary file 5 [file Data_Sheet_1.ZIP › Supplementary Materials S1/ROC/ROC GSE63061 PINK AD-CTL DG.pdf]

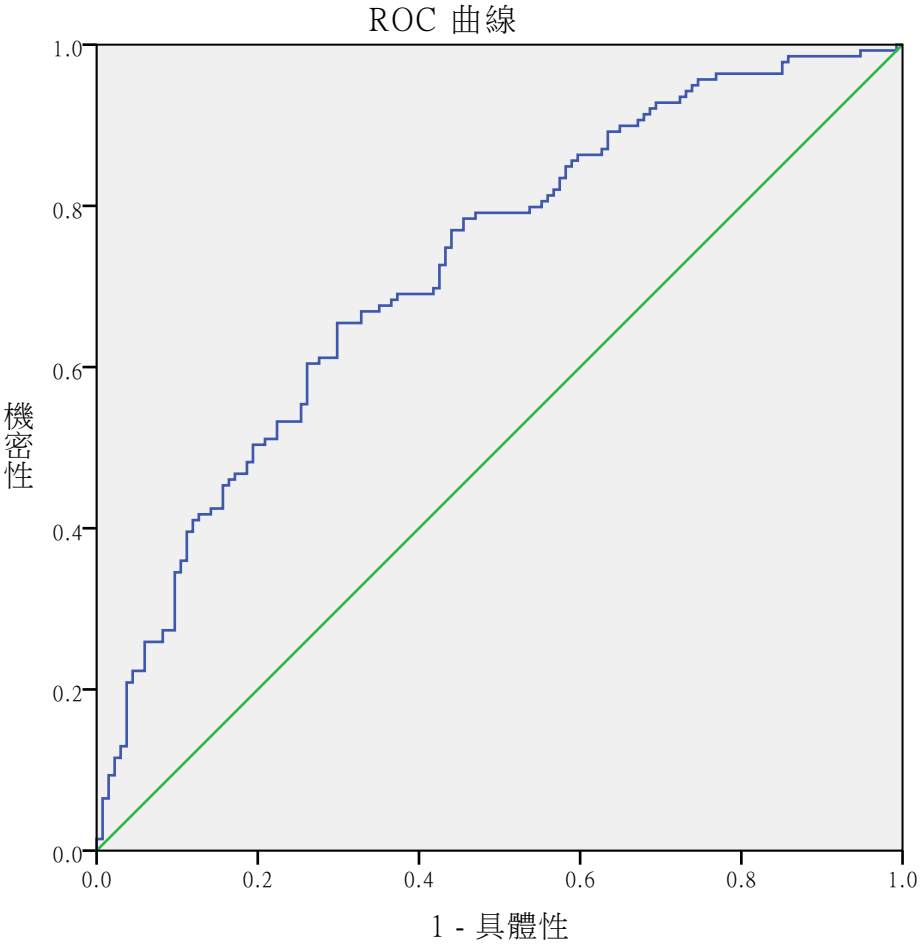

Supplement: Supplementary file 5 [file Data_Sheet_1.ZIP › Supplementary Materials S1/ROC/ROC GSE63061 PINK AD-CTL LH.pdf]

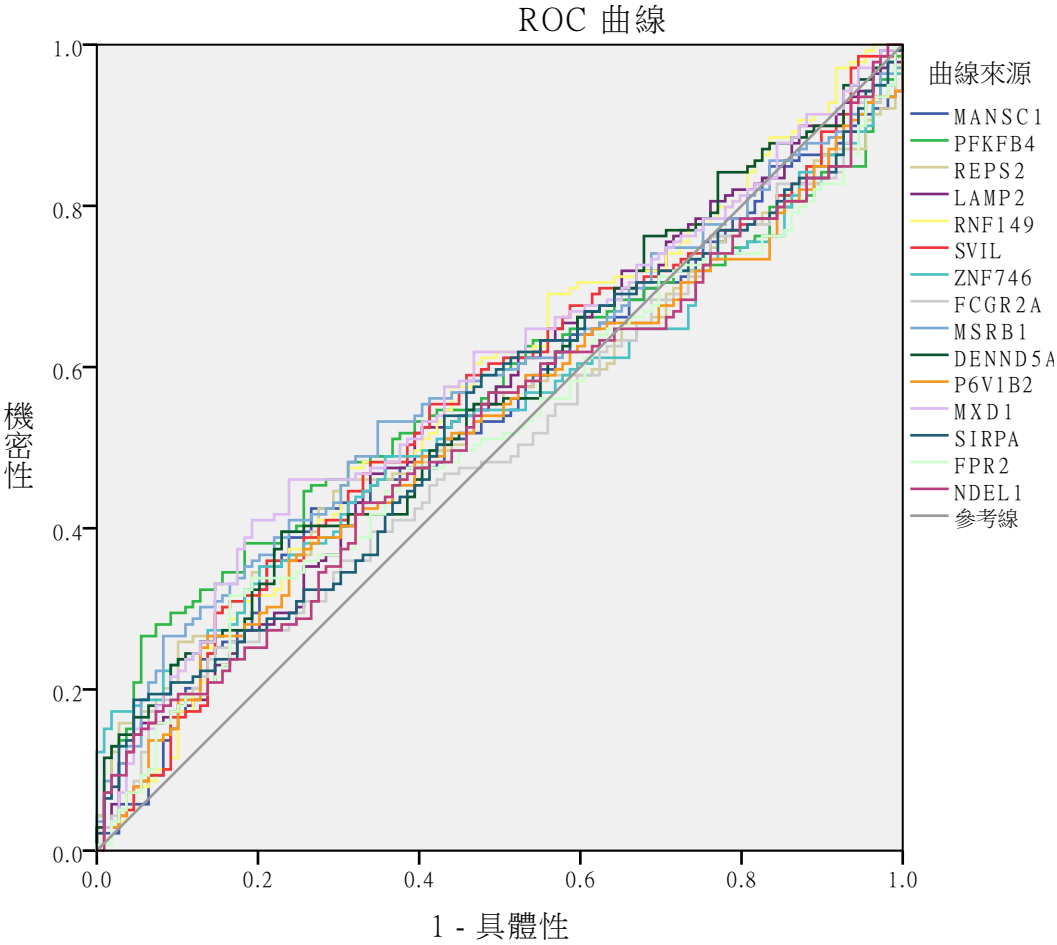

Supplement: Supplementary file 5 [file Data_Sheet_1.ZIP › Supplementary Materials S1/ROC/ROC GSE63061 PINK AD-MCI DG.pdf]

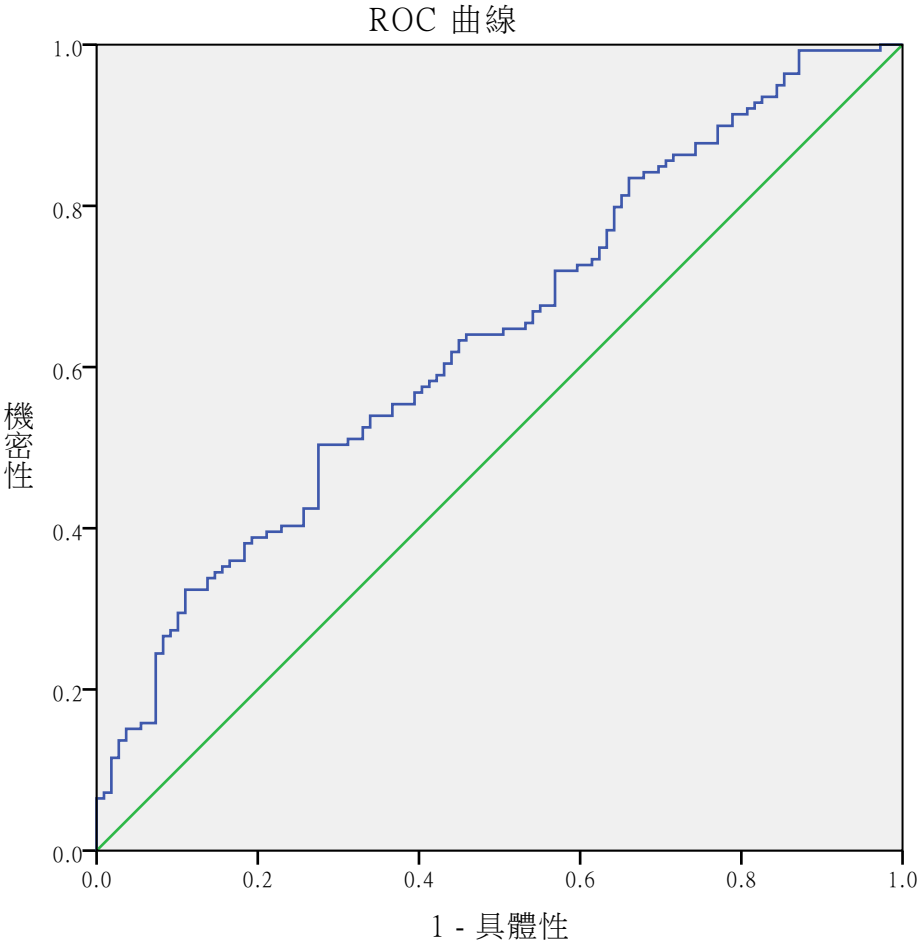

Supplement: Supplementary file 5 [file Data_Sheet_1.ZIP › Supplementary Materials S1/ROC/ROC GSE63061 PINK AD-MCI LH.pdf]

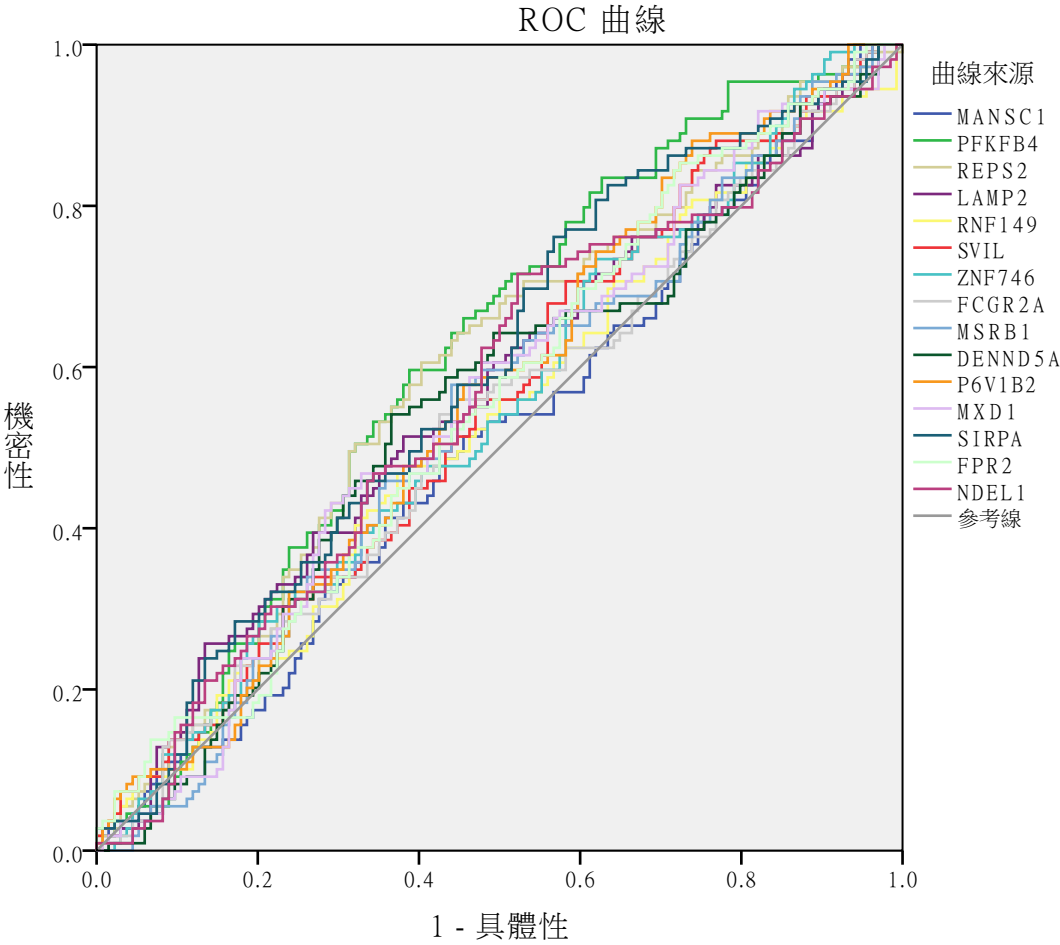

Supplement: Supplementary file 5 [file Data_Sheet_1.ZIP › Supplementary Materials S1/ROC/ROC GSE63061 PINK MCI-CTL DG.pdf]

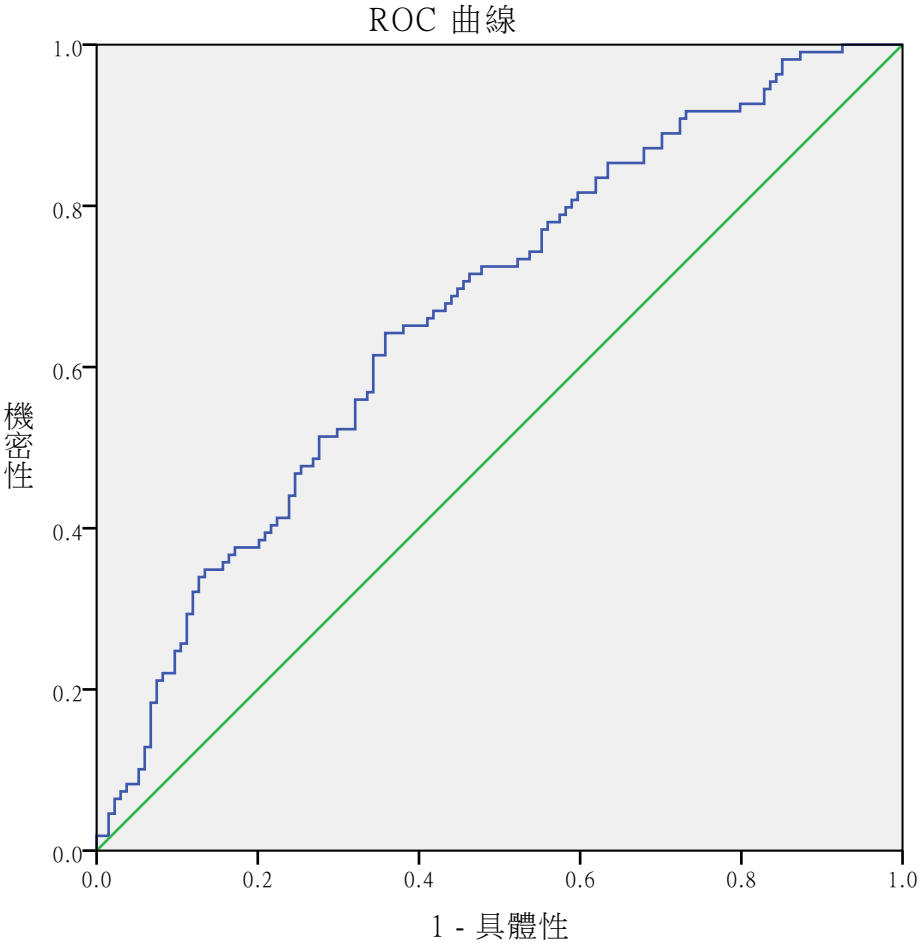

Supplement: Supplementary file 5 [file Data_Sheet_1.ZIP › Supplementary Materials S1/ROC/ROC GSE63061 PINK MCI-CTL LH.pdf]

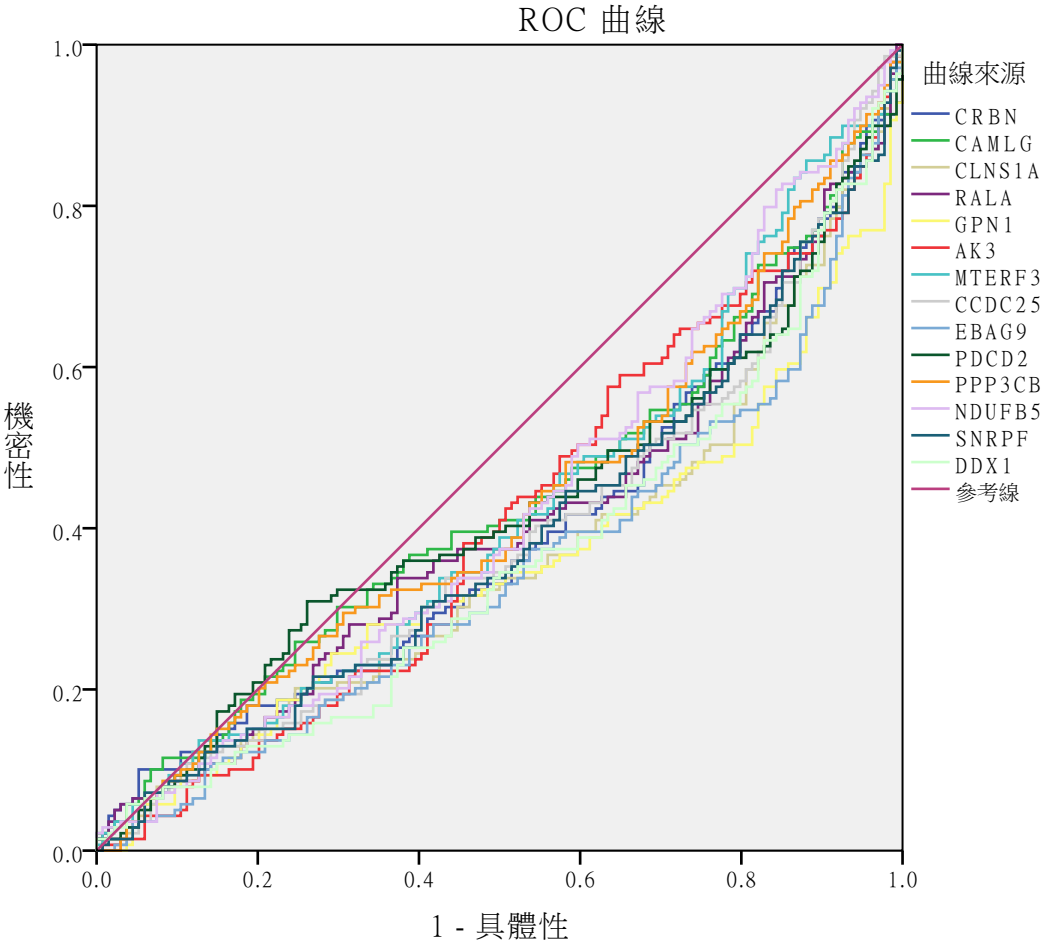

Supplement: Supplementary file 5 [file Data_Sheet_1.ZIP › Supplementary Materials S1/ROC/ROC GSE63061 RED AD-CTL DG.pdf]

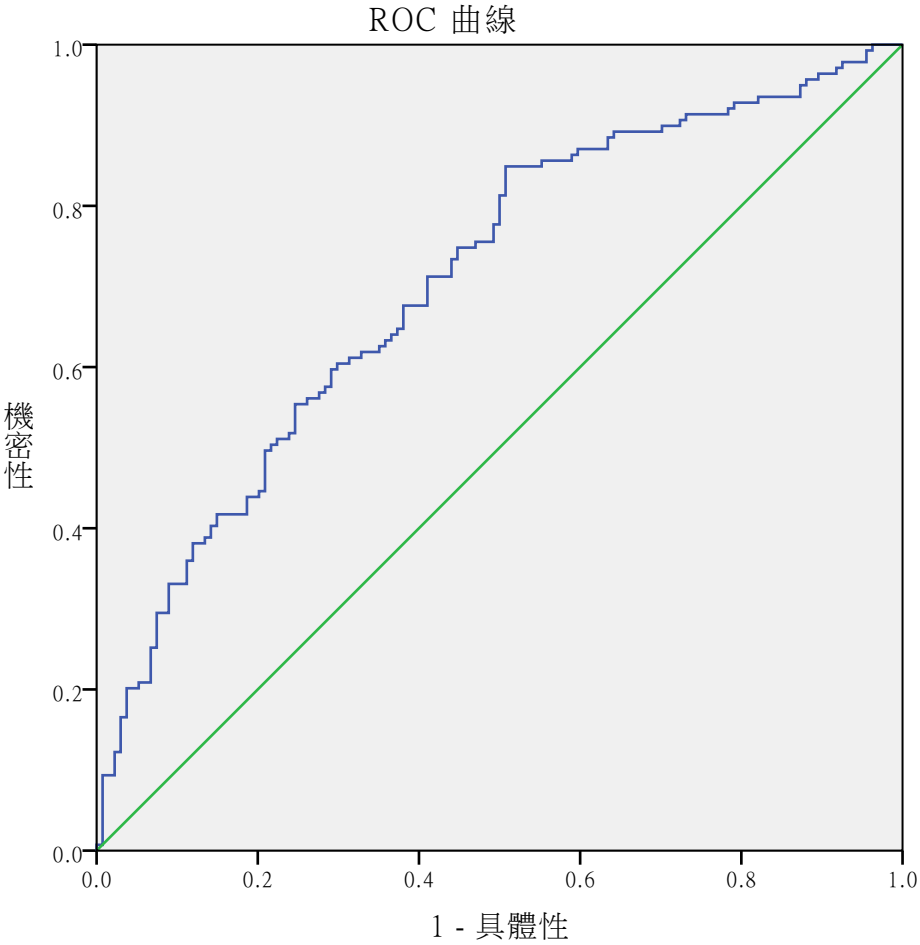

Supplement: Supplementary file 5 [file Data_Sheet_1.ZIP › Supplementary Materials S1/ROC/ROC GSE63061 RED AD-CTLLH.pdf]

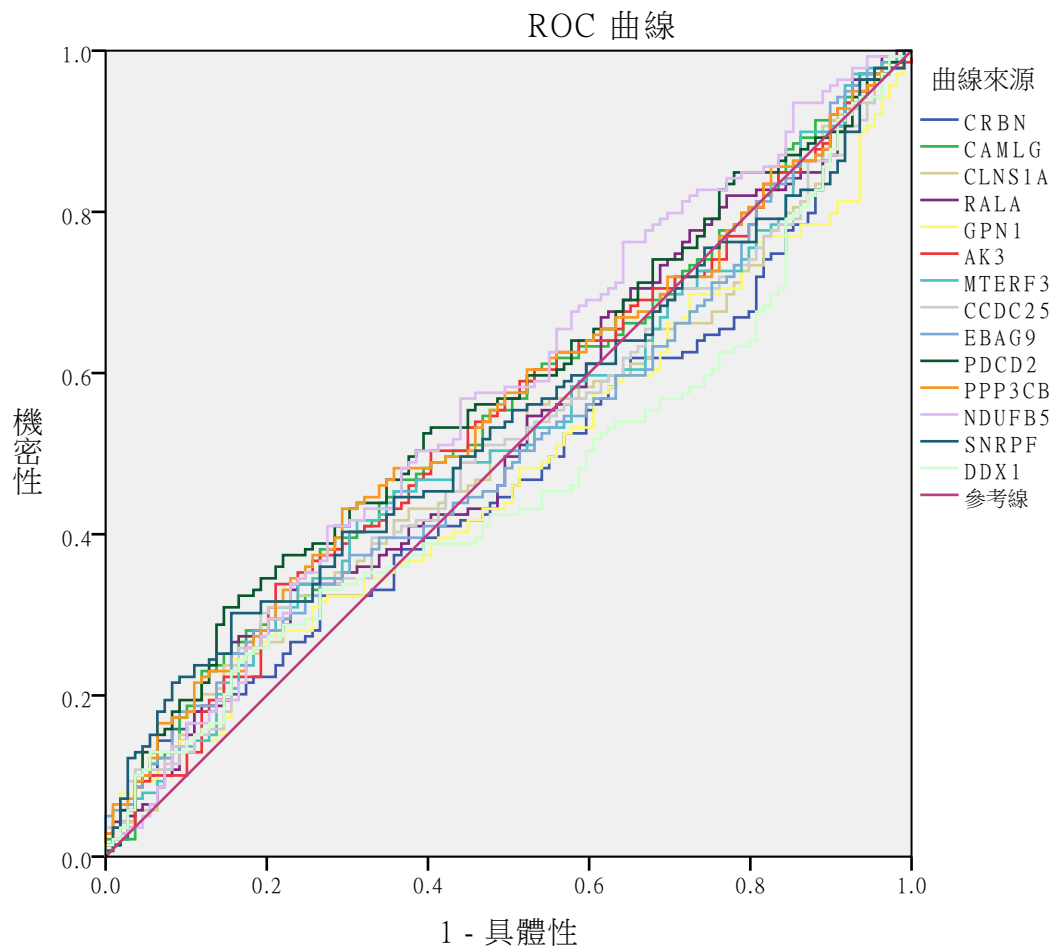

Supplement: Supplementary file 5 [file Data_Sheet_1.ZIP › Supplementary Materials S1/ROC/ROC GSE63061 RED AD-MCI DG.pdf]

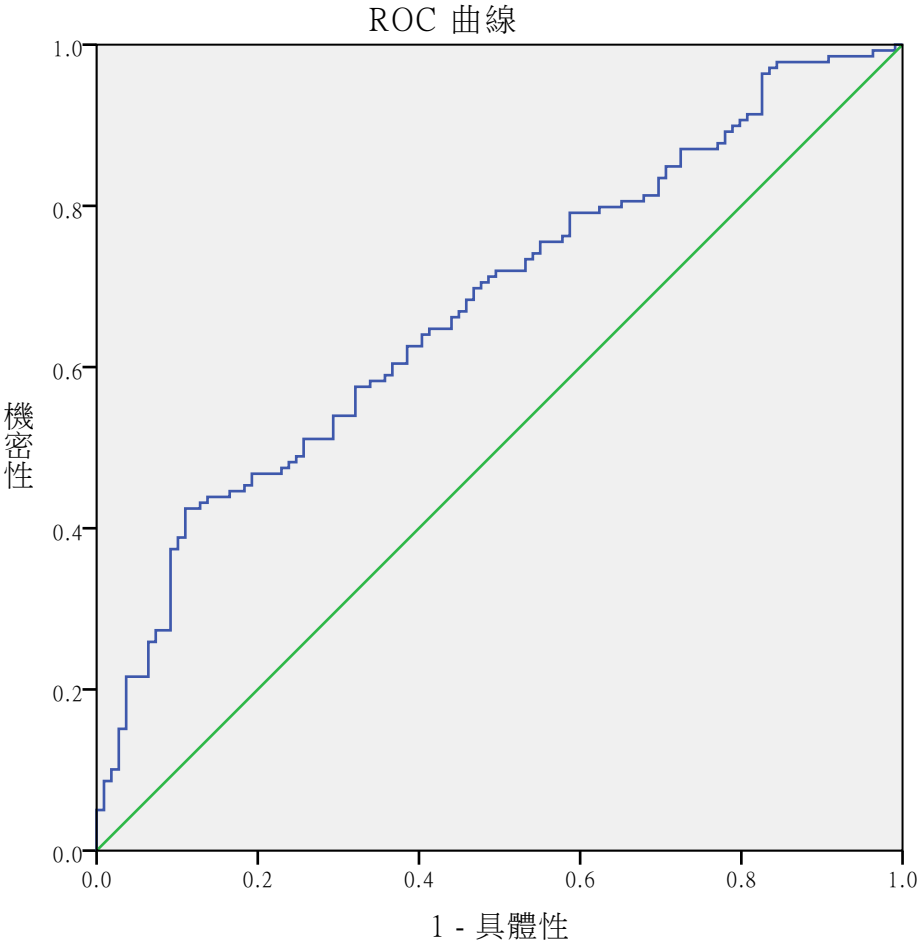

Supplement: Supplementary file 5 [file Data_Sheet_1.ZIP › Supplementary Materials S1/ROC/ROC GSE63061 RED AD-MCI LH.pdf]

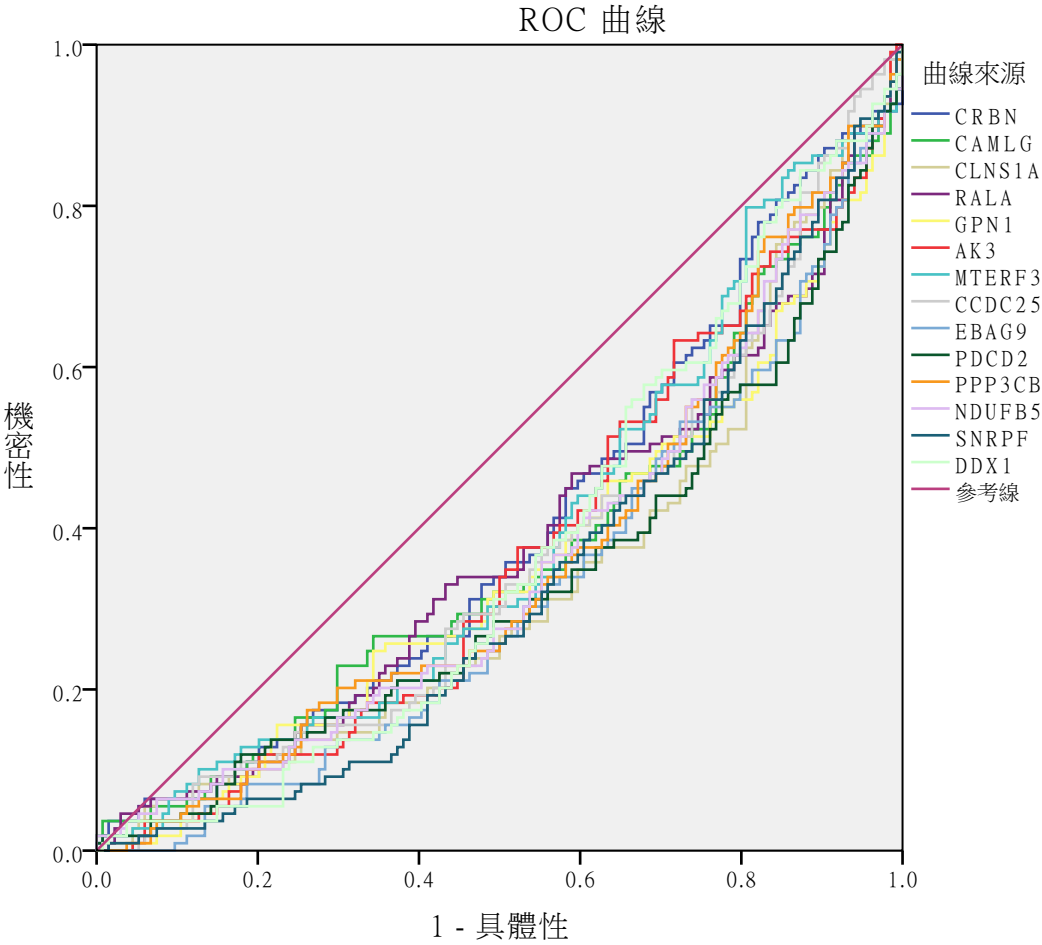

Supplement: Supplementary file 5 [file Data_Sheet_1.ZIP › Supplementary Materials S1/ROC/ROC GSE63061 RED MCI-CTL DG.pdf]

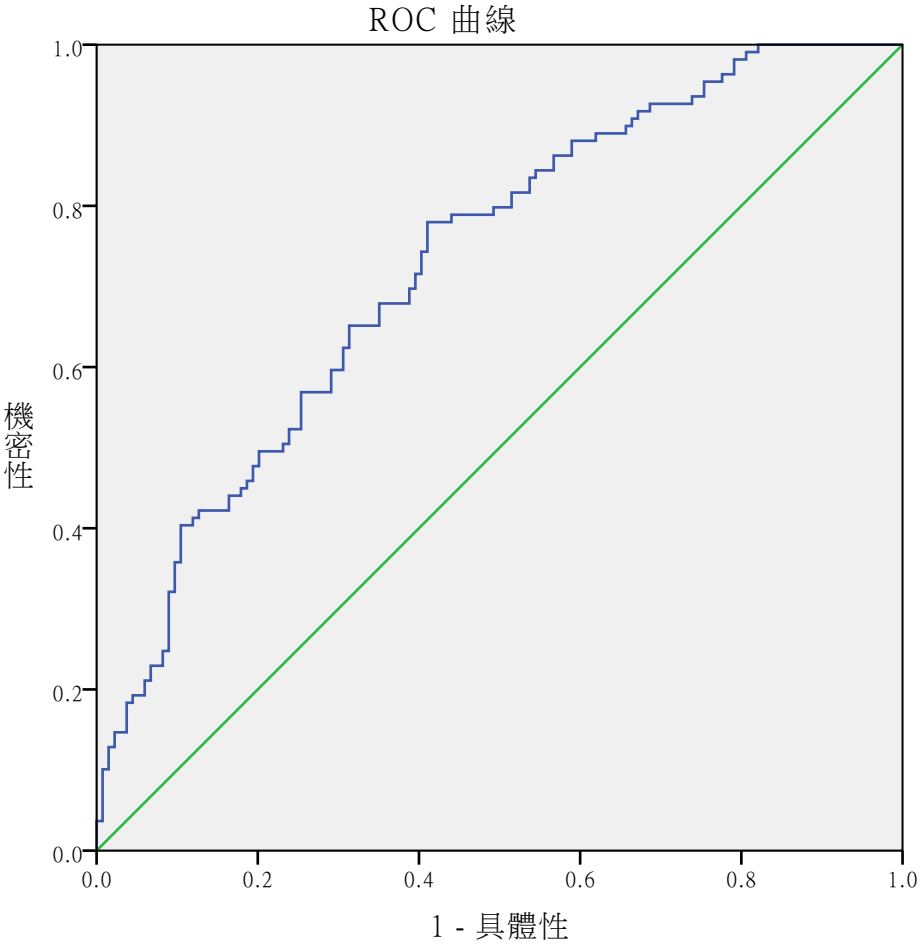

Supplement: Supplementary file 5 [file Data_Sheet_1.ZIP › Supplementary Materials S1/ROC/ROC GSE63061 RED MCI-CTL LH.pdf]

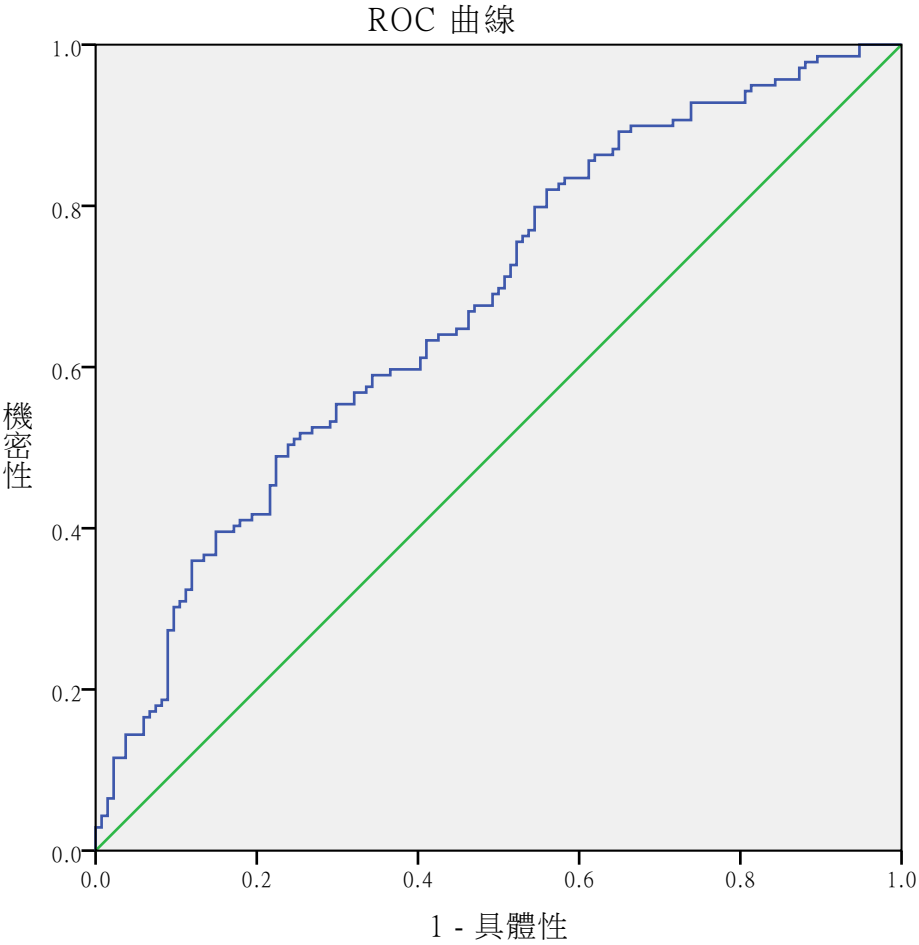

Supplement: Supplementary file 5 [file Data_Sheet_1.ZIP › Supplementary Materials S1/ROC/ROC GSE63061 TURQUIOES AD-CTL LH.pdf]

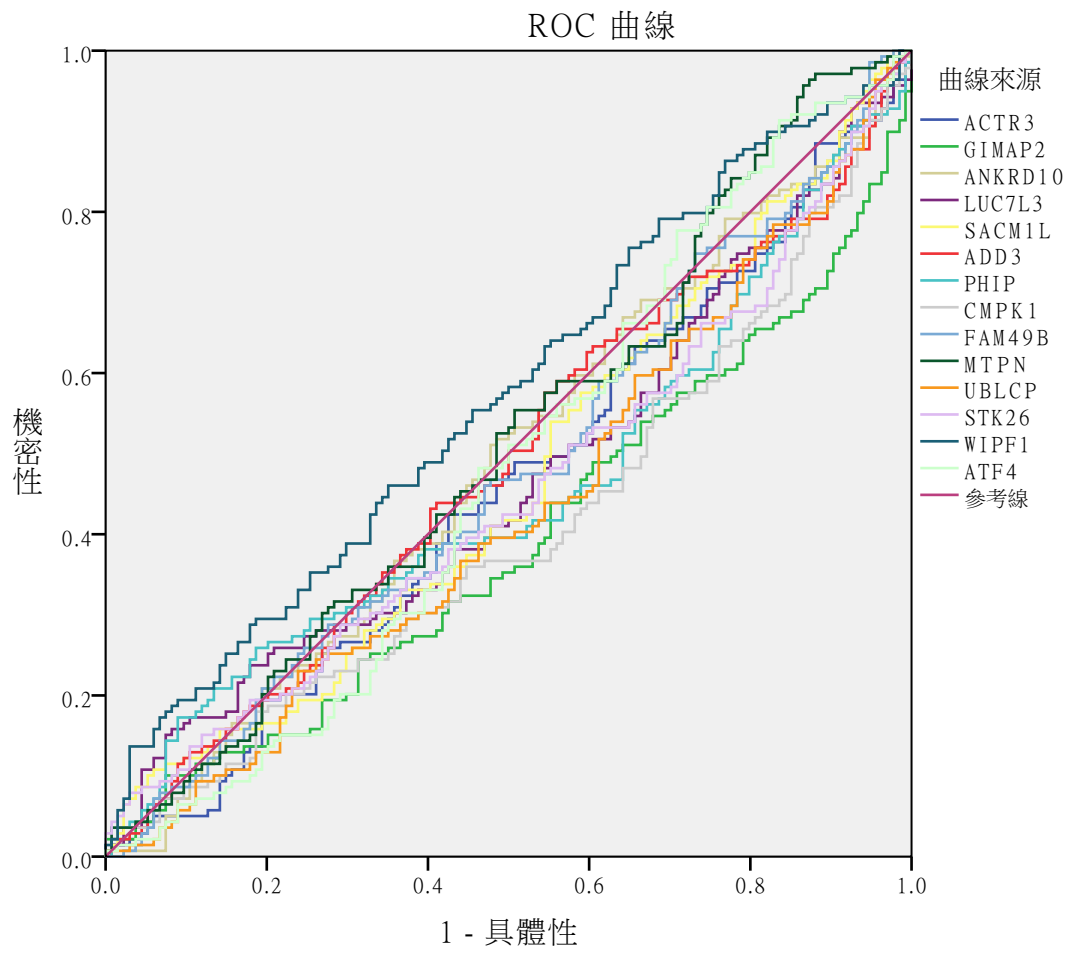

Supplement: Supplementary file 5 [file Data_Sheet_1.ZIP › Supplementary Materials S1/ROC/ROC GSE63061 TURQUIOES AD-CTLDG.pdf]

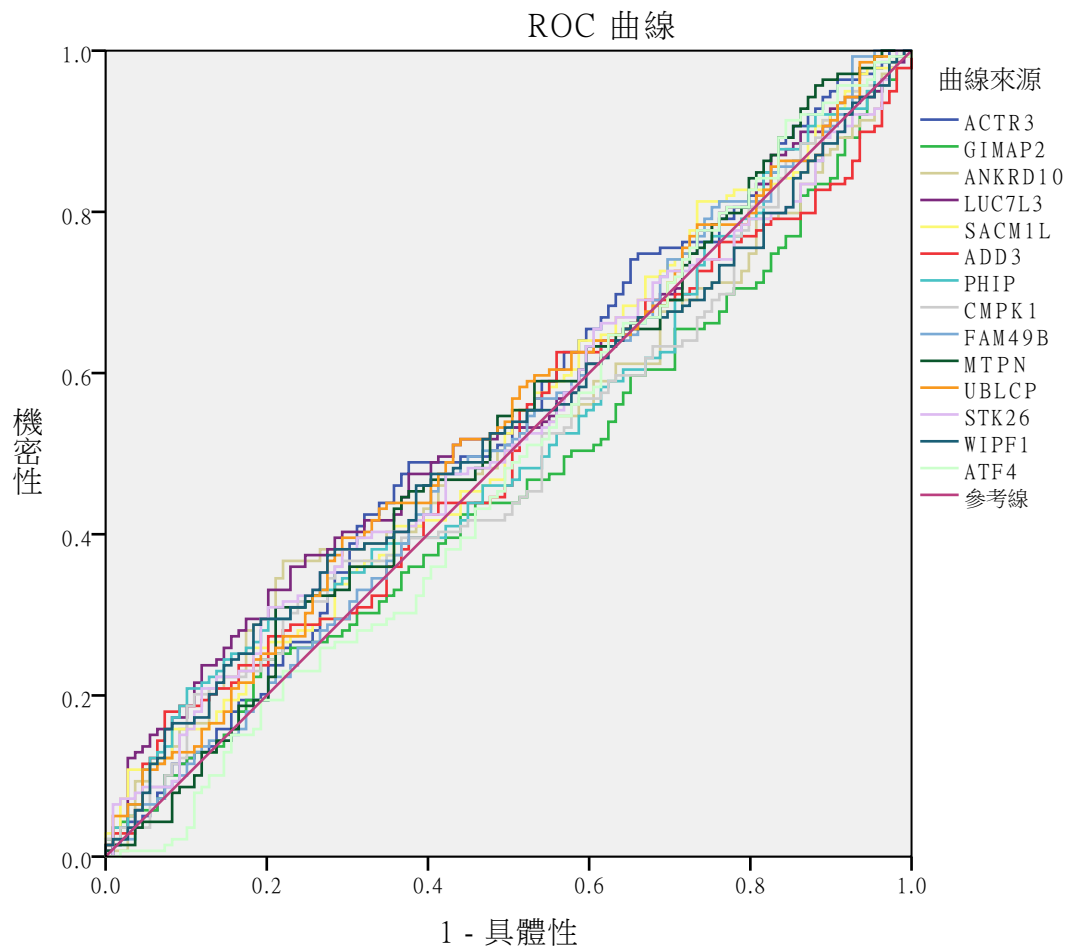

Supplement: Supplementary file 5 [file Data_Sheet_1.ZIP › Supplementary Materials S1/ROC/ROC GSE63061 TURQUIOES AD-MCI DG.pdf]

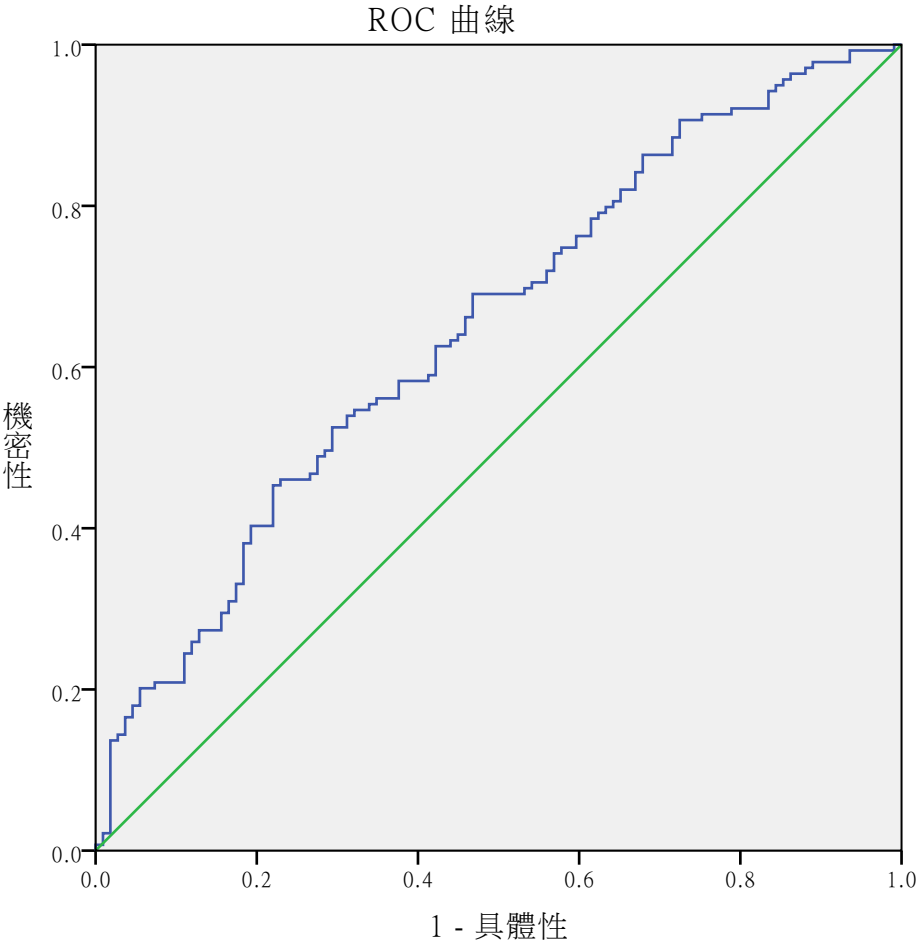

Supplement: Supplementary file 5 [file Data_Sheet_1.ZIP › Supplementary Materials S1/ROC/ROC GSE63061 TURQUIOES AD-MCI LHG.pdf]

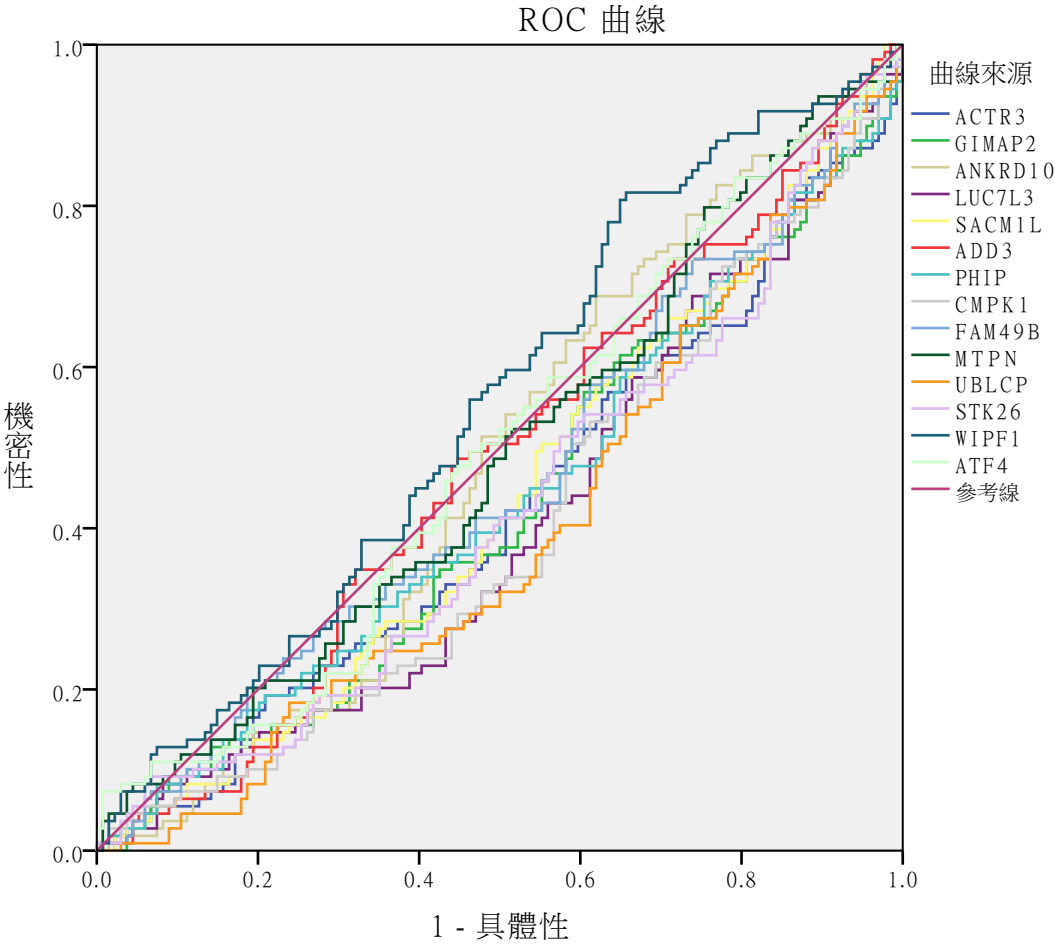

Supplement: Supplementary file 5 [file Data_Sheet_1.ZIP › Supplementary Materials S1/ROC/ROC GSE63061 TURQUIOES MCI-CTL DG.pdf]

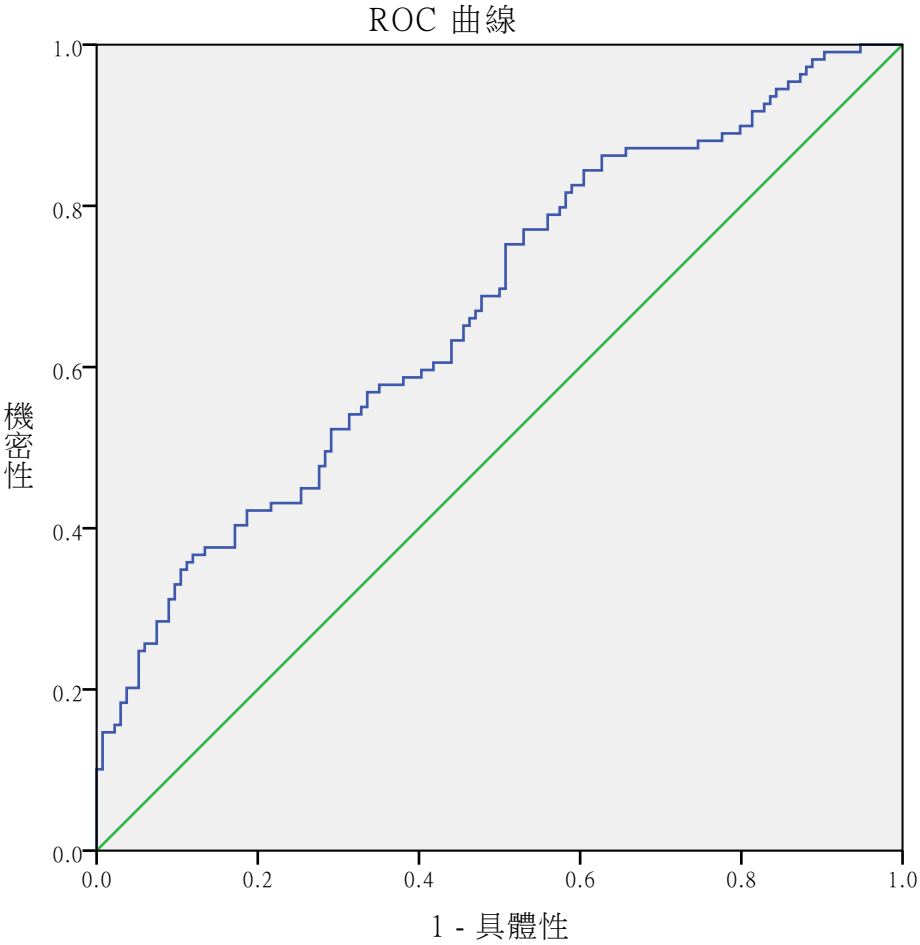

Supplement: Supplementary file 5 [file Data_Sheet_1.ZIP › Supplementary Materials S1/ROC/ROC GSE63061 TURQUIOES MCI-CTL LH.pdf]

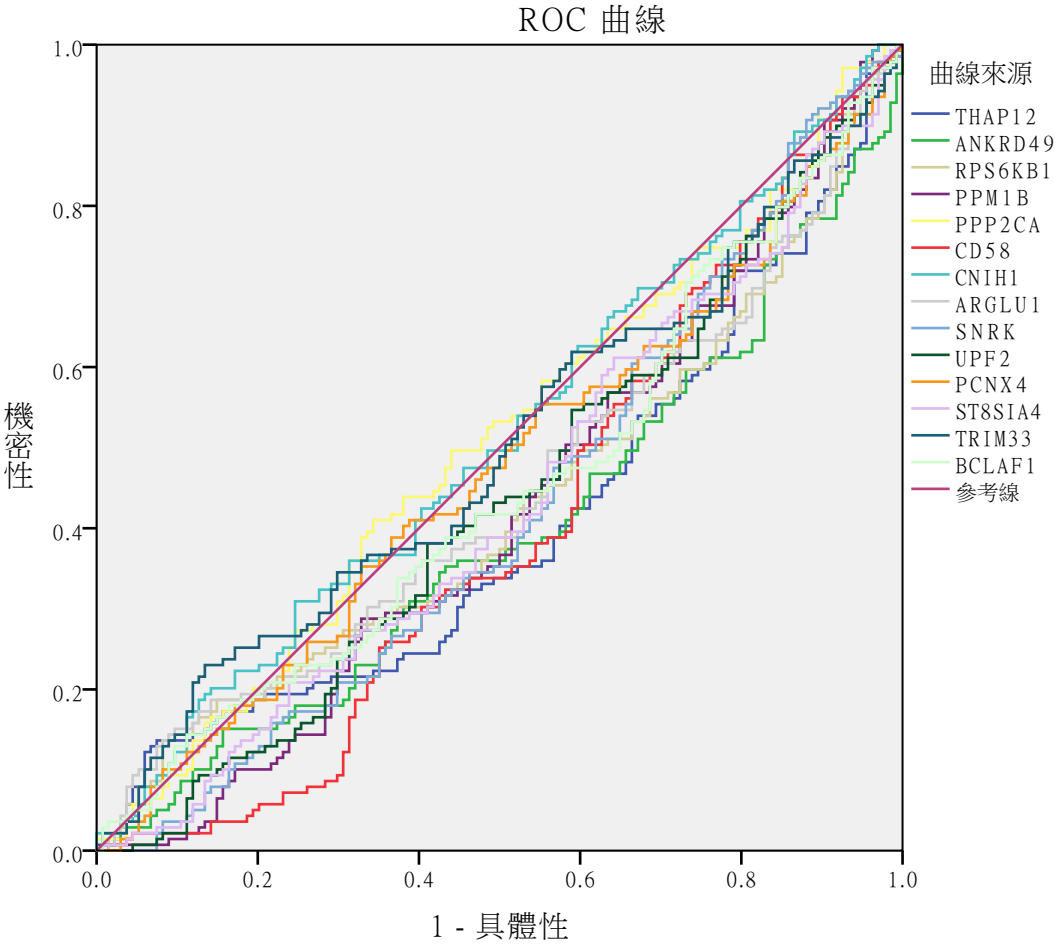

Supplement: Supplementary file 5 [file Data_Sheet_1.ZIP › Supplementary Materials S1/ROC/ROC GSE63061 YELLOW AD-CTL DG.pdf]

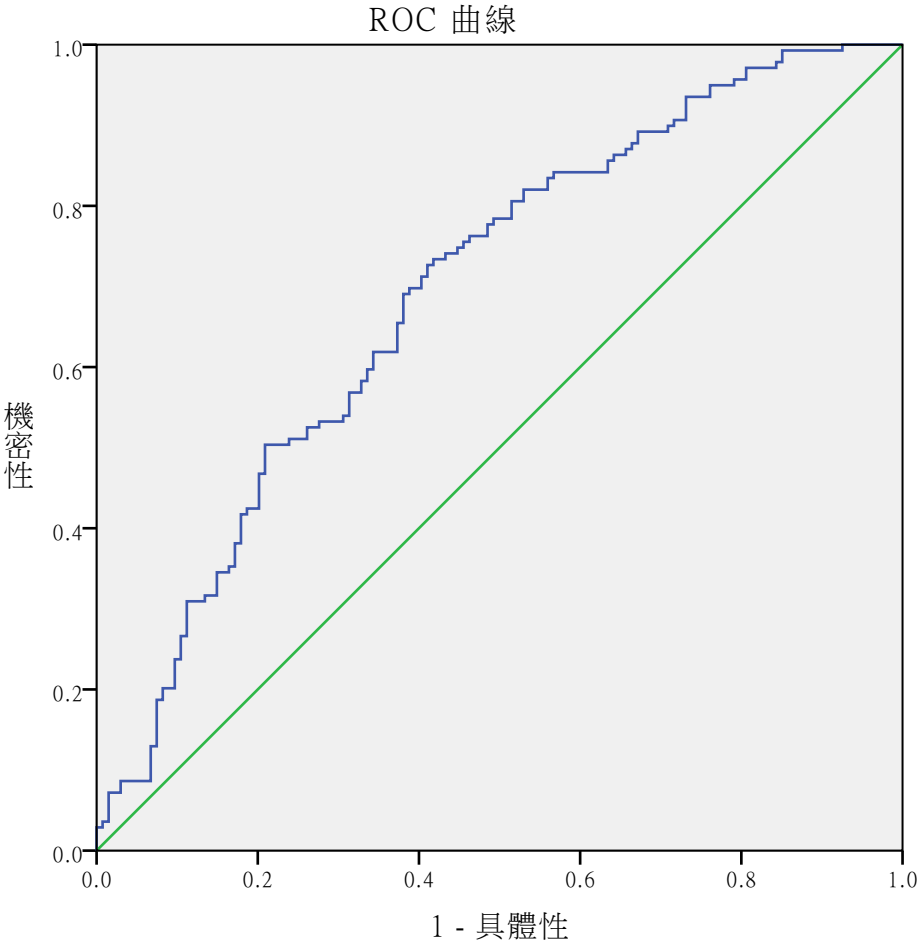

Supplement: Supplementary file 5 [file Data_Sheet_1.ZIP › Supplementary Materials S1/ROC/ROC GSE63061 YELLOW AD-CTL LH.pdf]

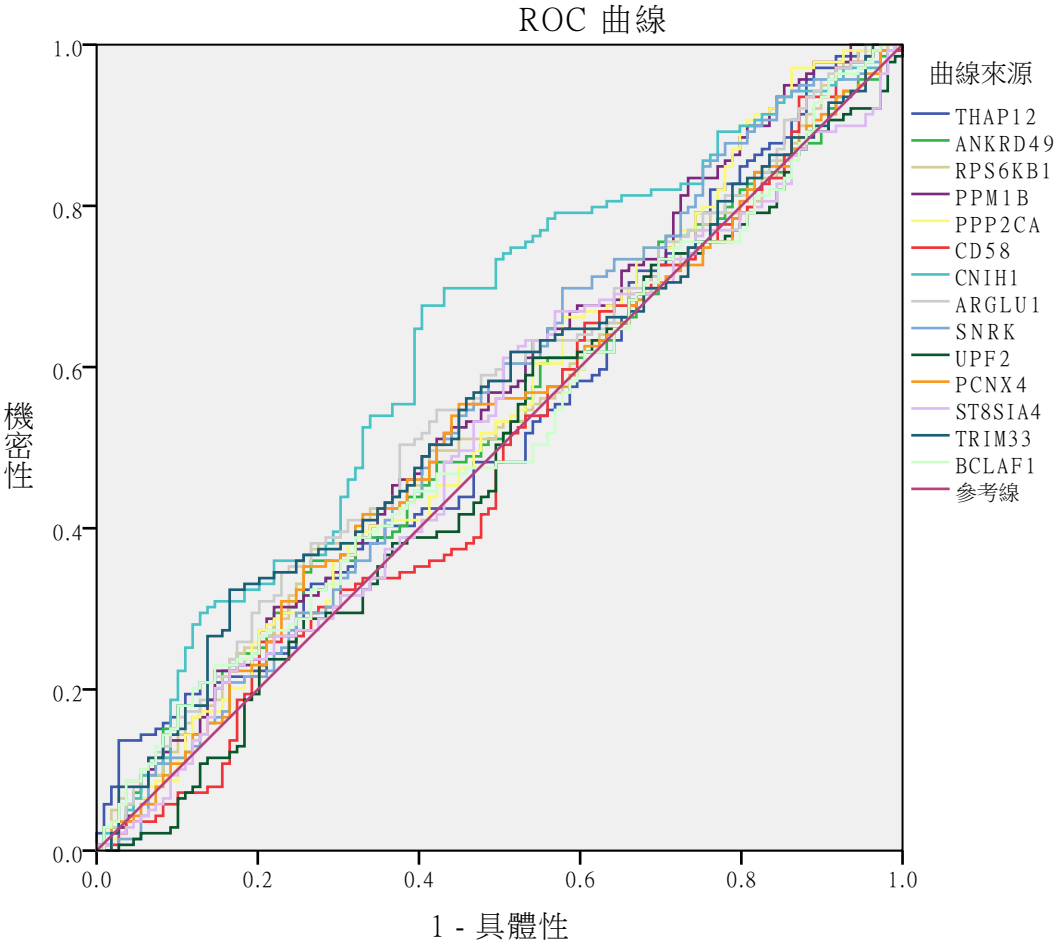

Supplement: Supplementary file 5 [file Data_Sheet_1.ZIP › Supplementary Materials S1/ROC/ROC GSE63061 YELLOW AD-MCI DG.pdf]

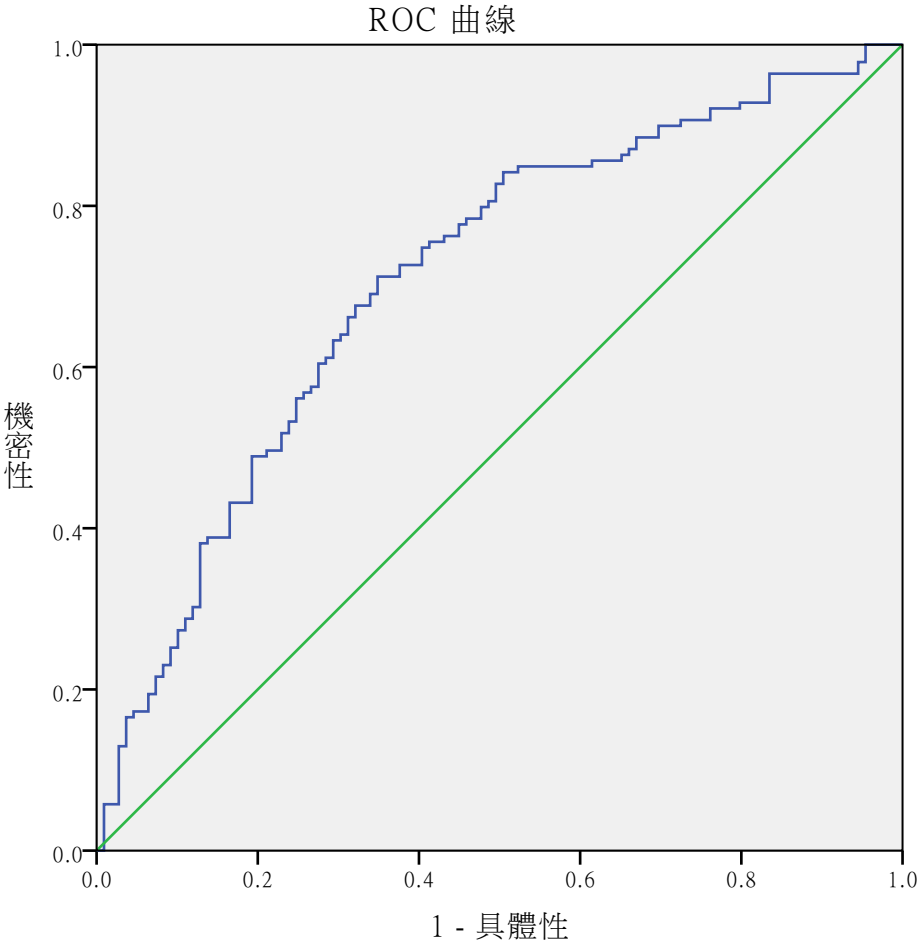

Supplement: Supplementary file 5 [file Data_Sheet_1.ZIP › Supplementary Materials S1/ROC/ROC GSE63061 YELLOW AD-MCI LH.pdf]

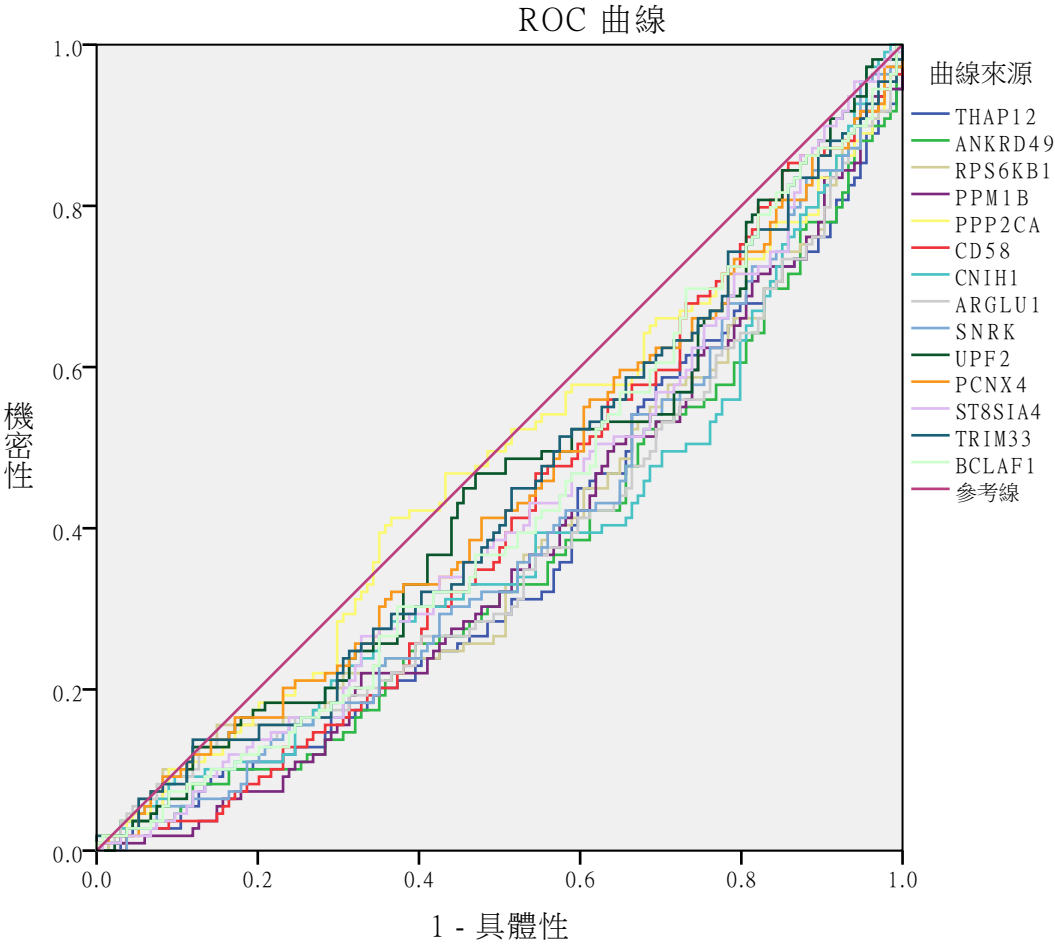

Supplement: Supplementary file 5 [file Data_Sheet_1.ZIP › Supplementary Materials S1/ROC/ROC GSE63061 YELLOW MCI-CTL DG.pdf]

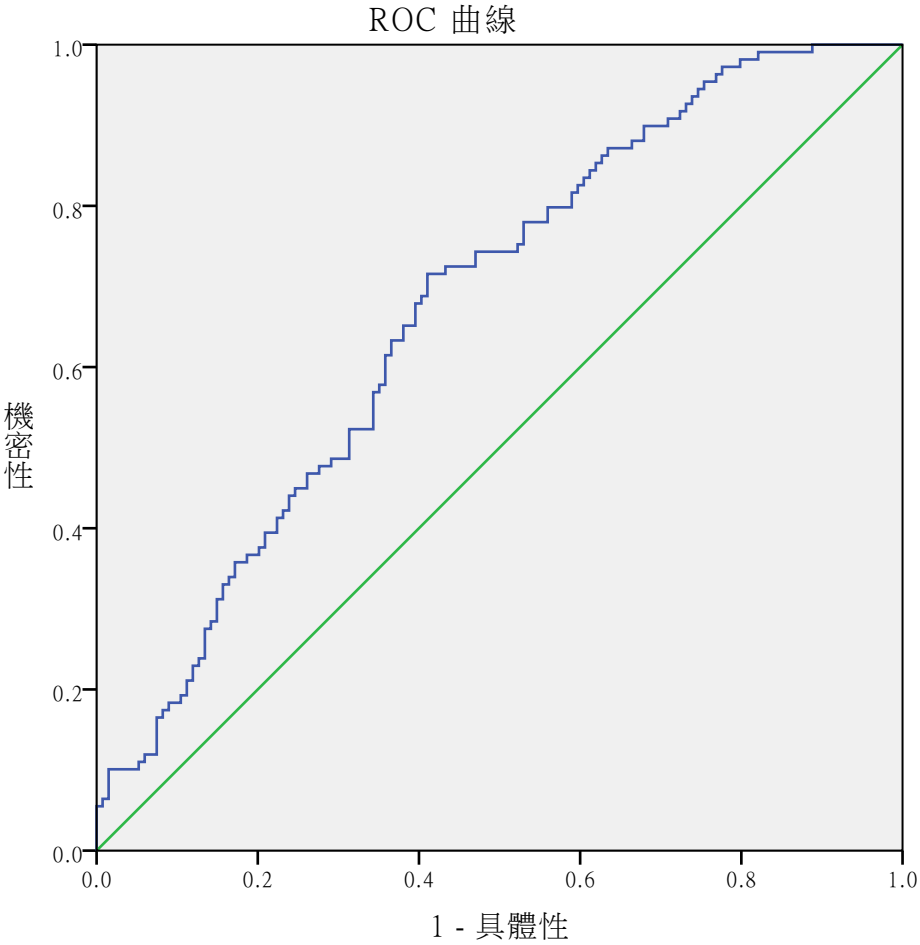

Supplement: Supplementary file 5 [file Data_Sheet_1.ZIP › Supplementary Materials S1/ROC/ROC GSE63061 YELLOW MCI-CTL LH.pdf]

**Module membership vs. gene significance**  
**cor=0.46, p=6.4e-14**

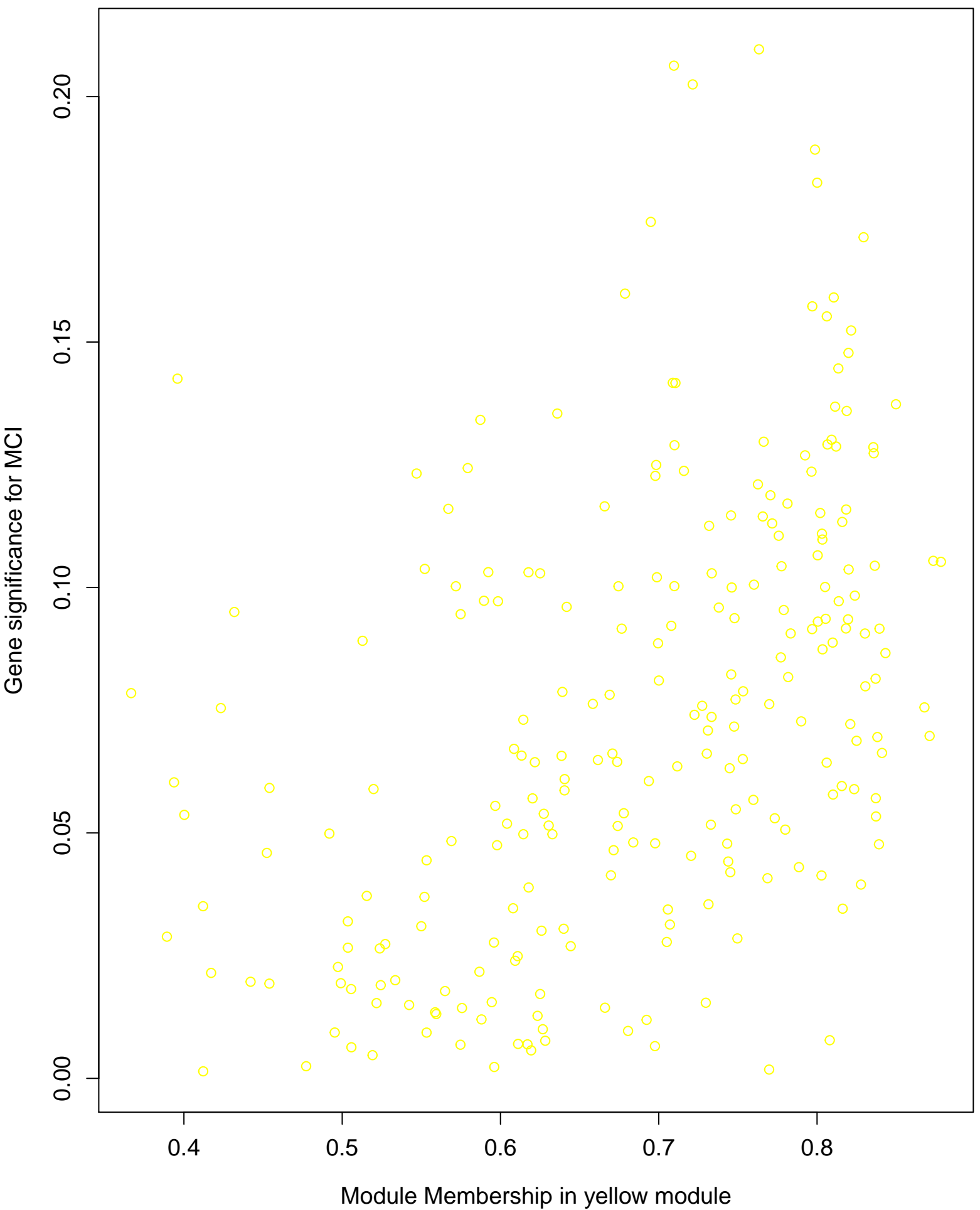

Supplement: Supplementary file 5 [file Data_Sheet_1.ZIP › Supplementary Materials S1/gse63061/yellow mci.pdf]

**Module membership vs. gene significance**  
**cor=0.5, p=1.6e-16**

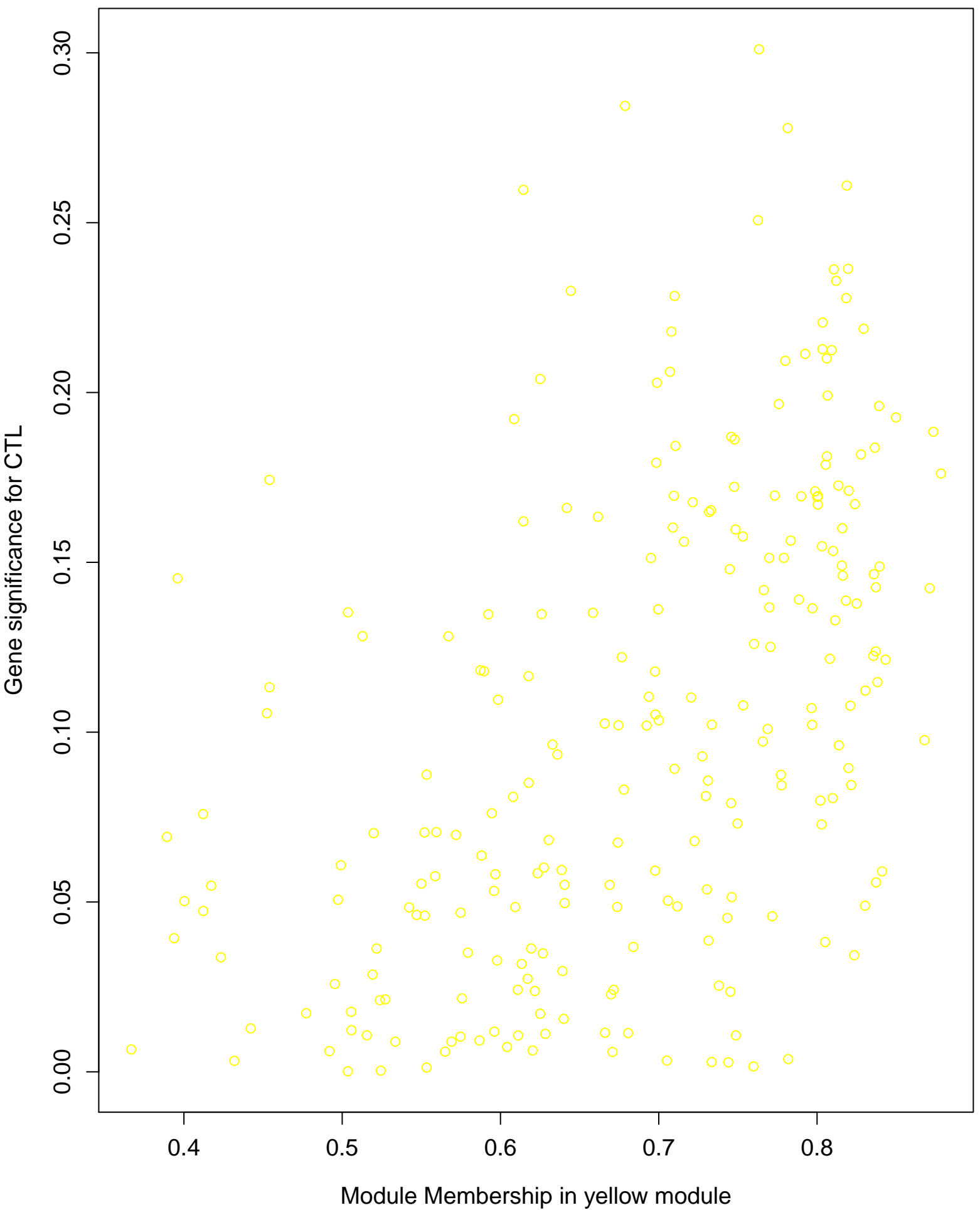

Supplement: Supplementary file 5 [file Data_Sheet_1.ZIP › Supplementary Materials S1/gse63061/yellow CTL.pdf]

**Module membership vs. gene significance**  
**cor=0.39, p=5.9e-66**

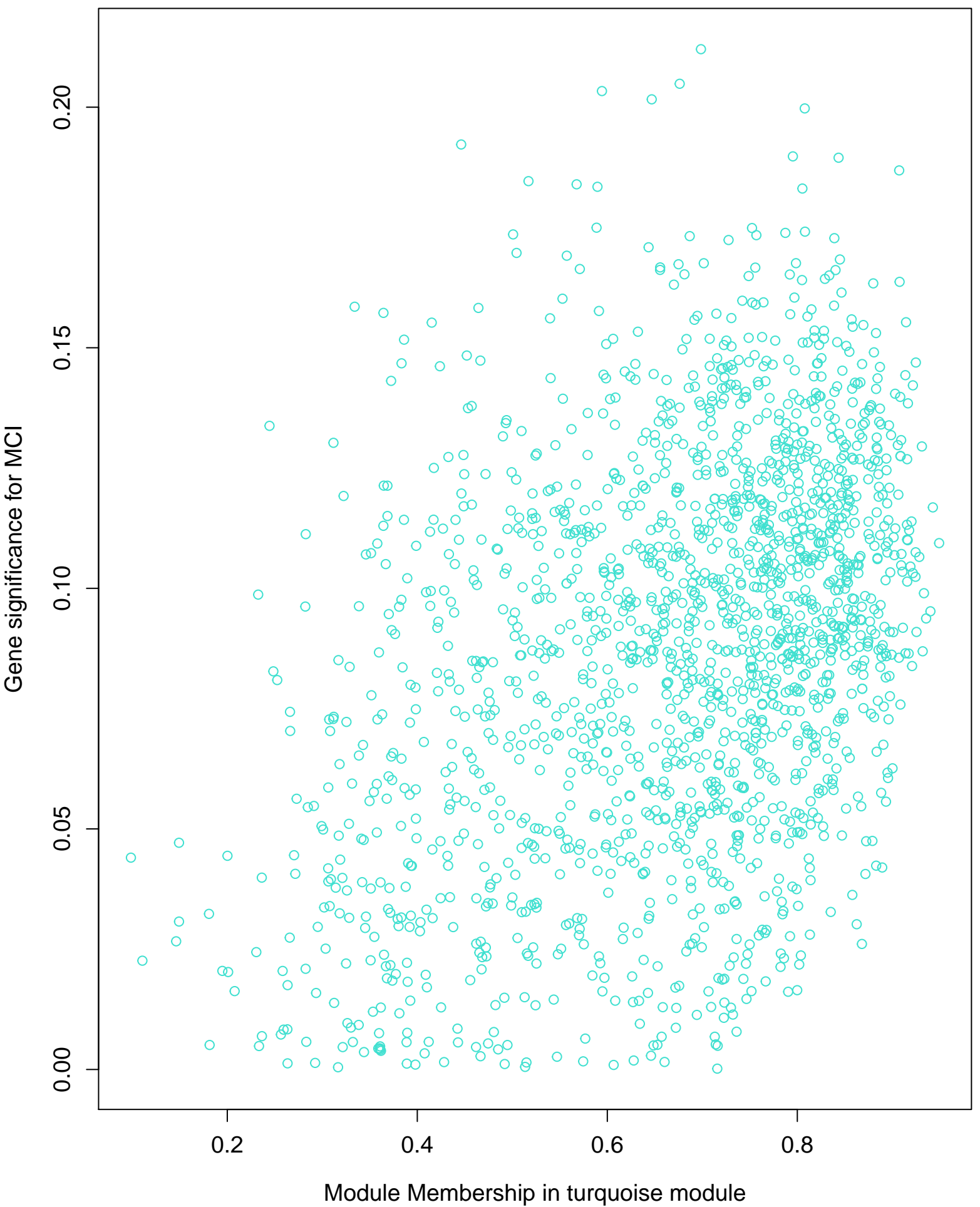

Supplement: Supplementary file 5 [file Data_Sheet_1.ZIP › Supplementary Materials S1/gse63061/turquoise mci.pdf]

**Module membership vs. gene significance**  
**cor=0.35, p=1.3e-52**

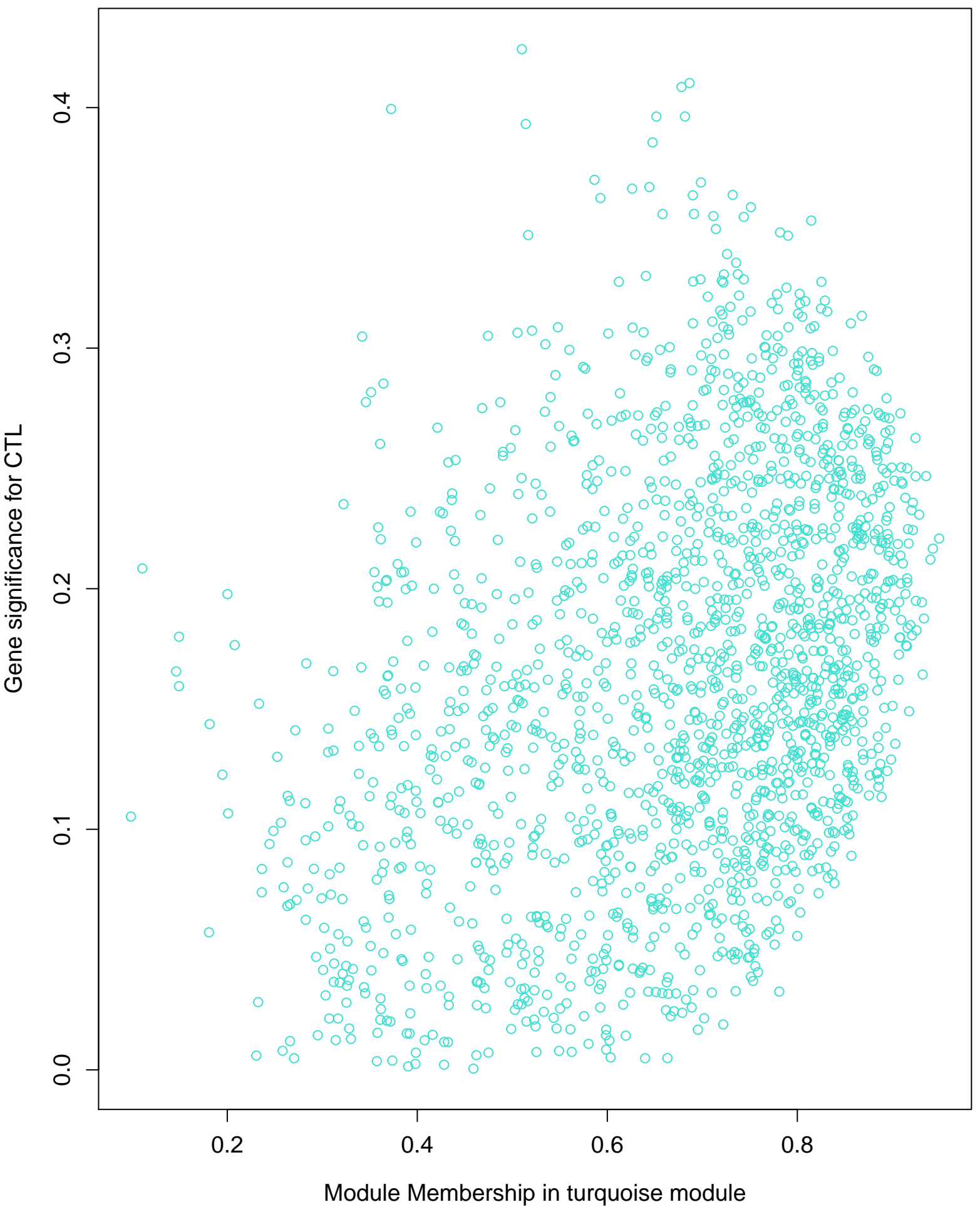

Supplement: Supplementary file 5 [file Data_Sheet_1.ZIP › Supplementary Materials S1/gse63061/turquoise CTL.pdf]

**Module membership vs. gene significance**  
**cor=0.11, p=3.2e-06**

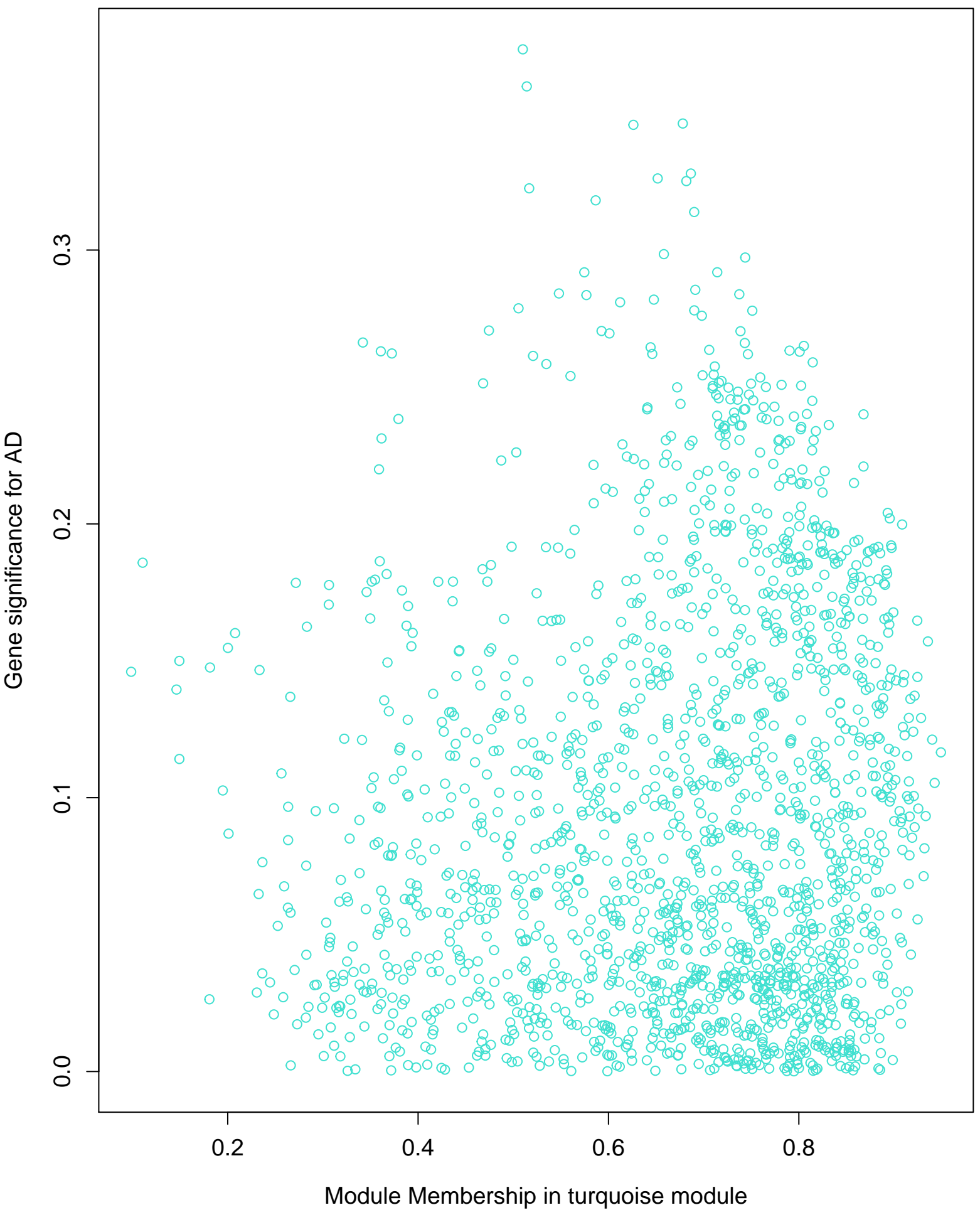

Supplement: Supplementary file 5 [file Data_Sheet_1.ZIP › Supplementary Materials S1/gse63061/turquoise AD.pdf]

**Module membership vs. gene significance**  
**cor=0.25, p=2.5e-18**

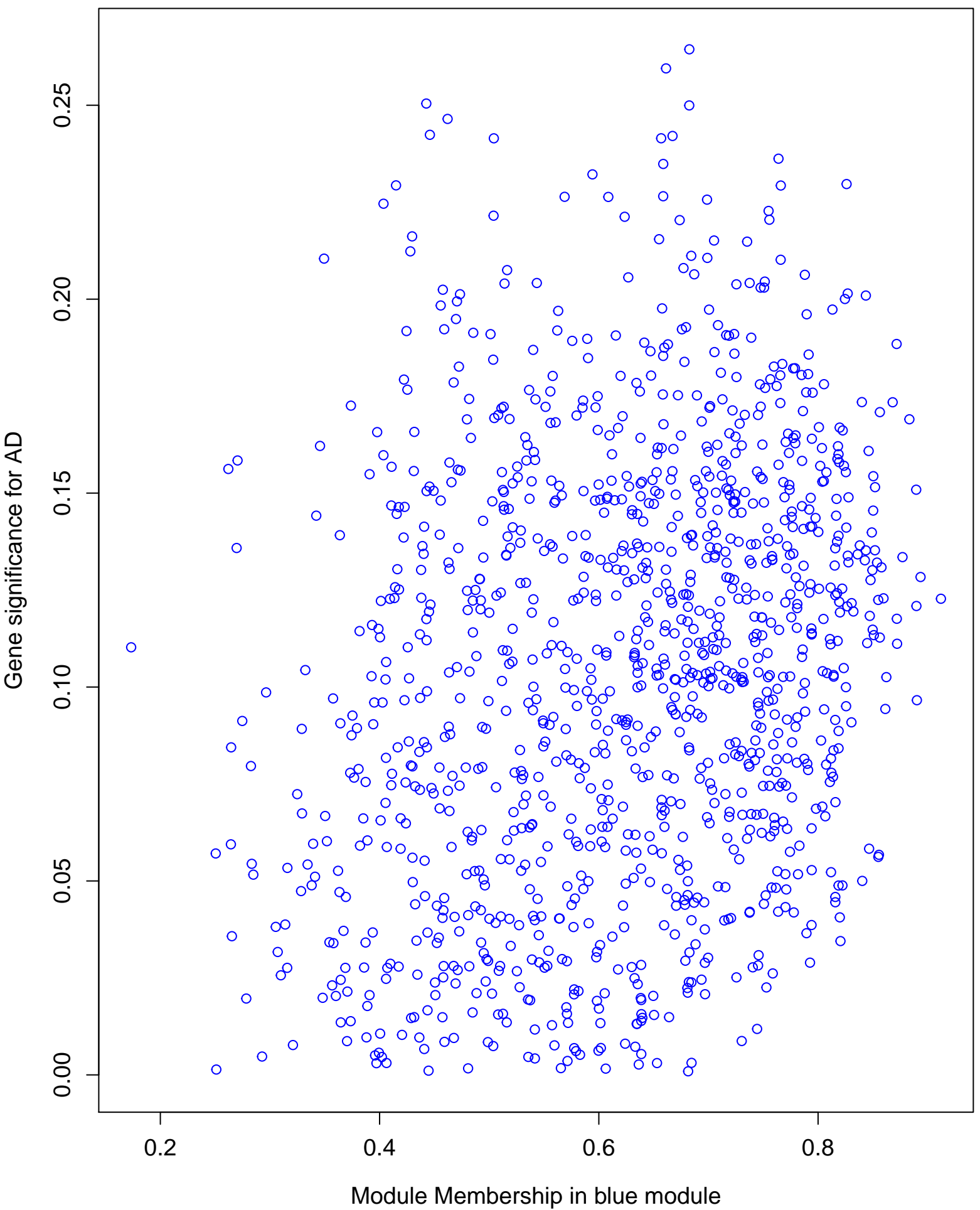

Supplement: Supplementary file 5 [file Data_Sheet_1.ZIP › Supplementary Materials S1/gse63061/blue.pdf]

**Module membership vs. gene significance**  
**cor=0.51, p=2.2e-79**

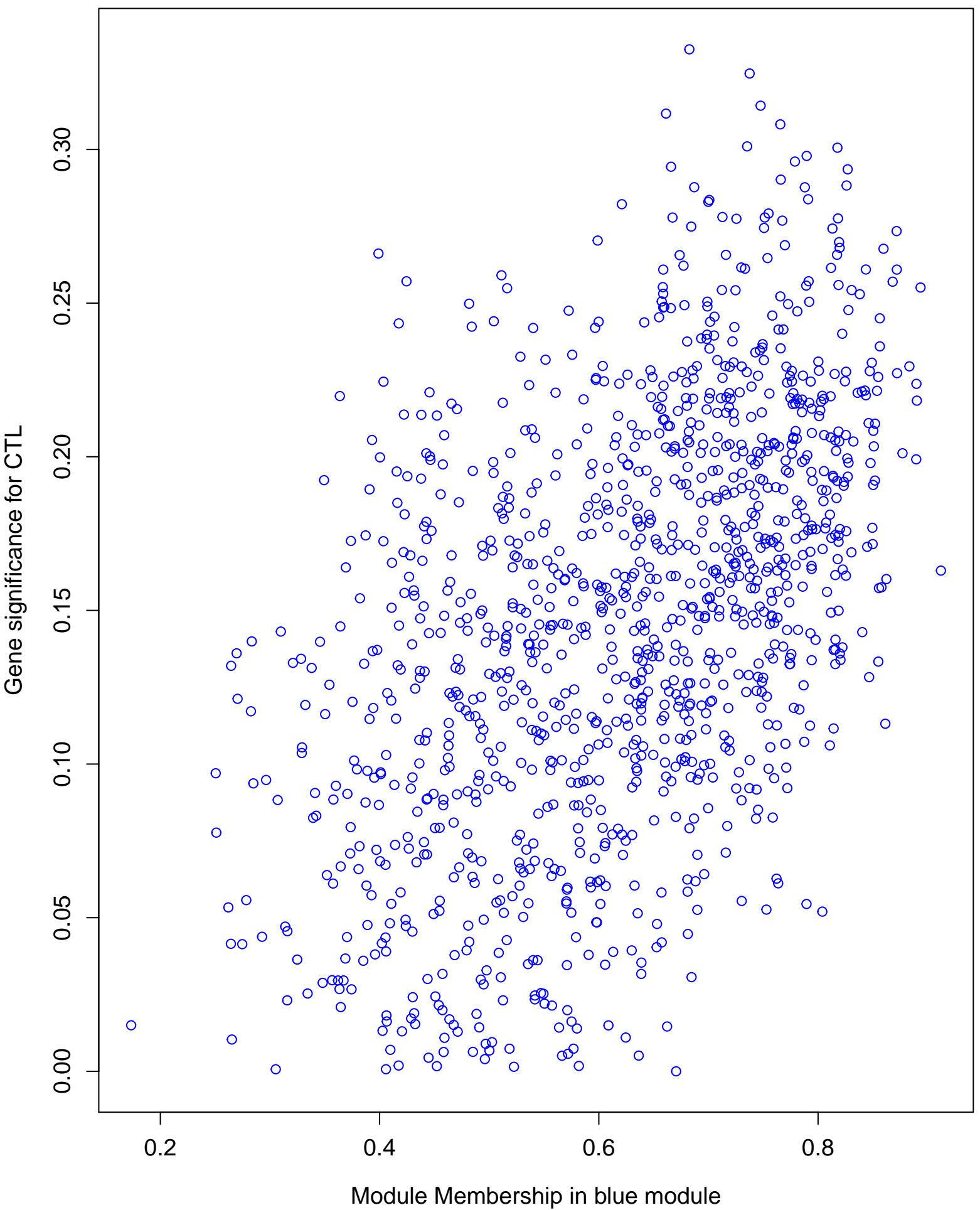

Supplement: Supplementary file 5 [file Data_Sheet_1.ZIP › Supplementary Materials S1/gse63061/blue CTL.pdf]

**Module membership vs. gene significance**  
**cor=0.25, p=2.5e-18**

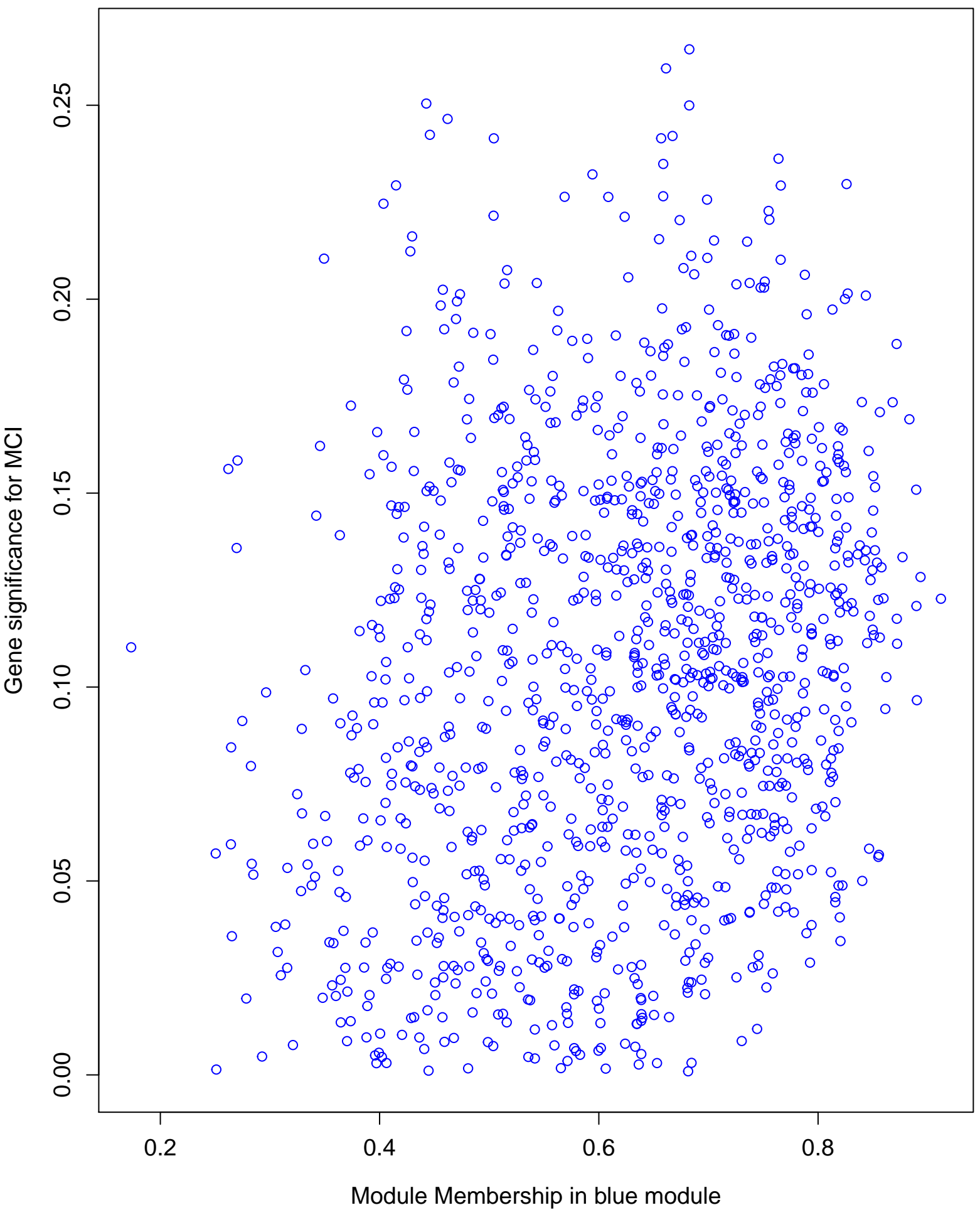

Supplement: Supplementary file 5 [file Data_Sheet_1.ZIP › Supplementary Materials S1/gse63061/blue AD.pdf]

**Module membership vs. gene significance**  
**cor=0.03, p=0.67**

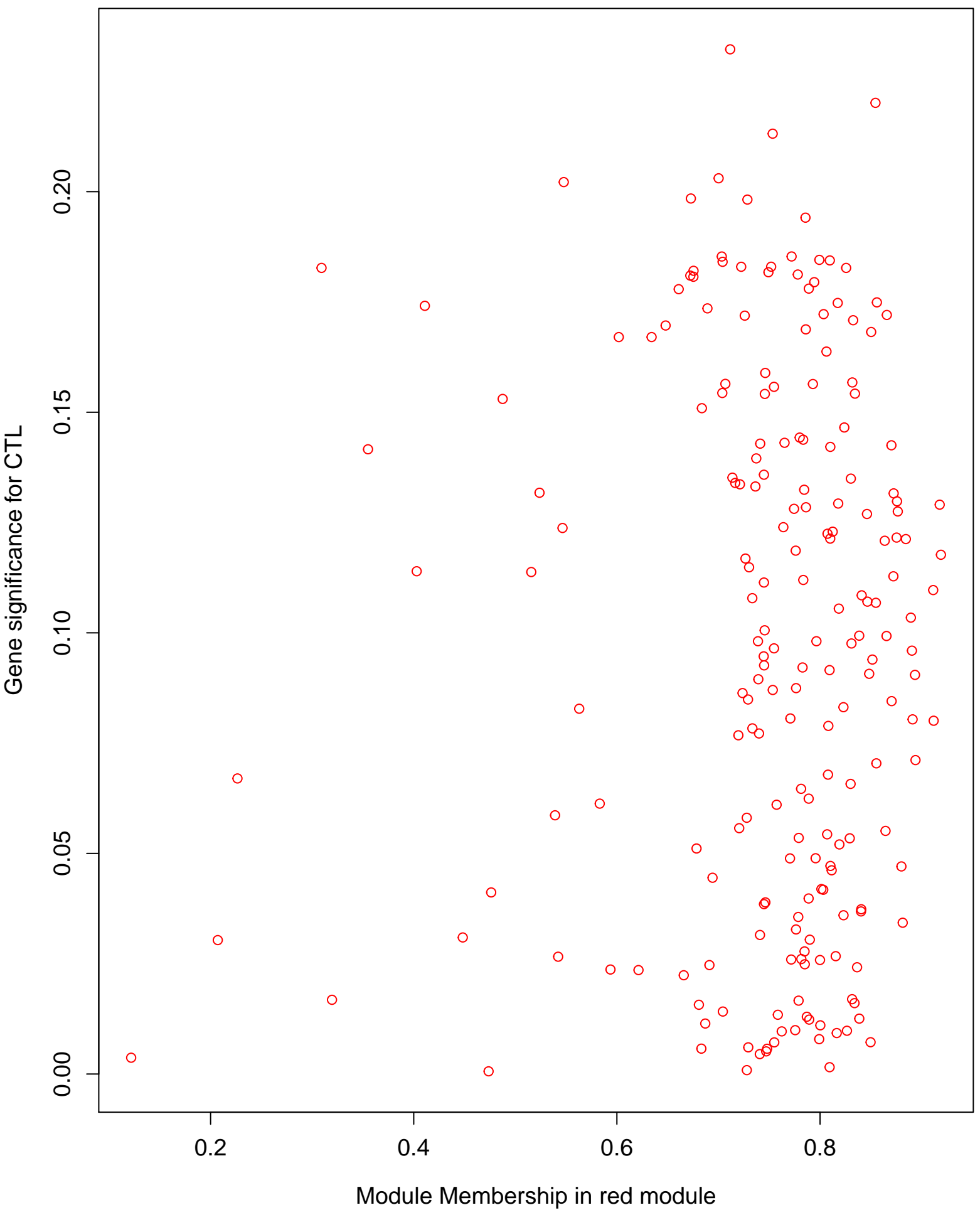

Supplement: Supplementary file 5 [file Data_Sheet_1.ZIP › Supplementary Materials S1/gse63061/RED CTL.pdf]

**Module membership vs. gene significance**  
**cor=0.26, p=0.00015**

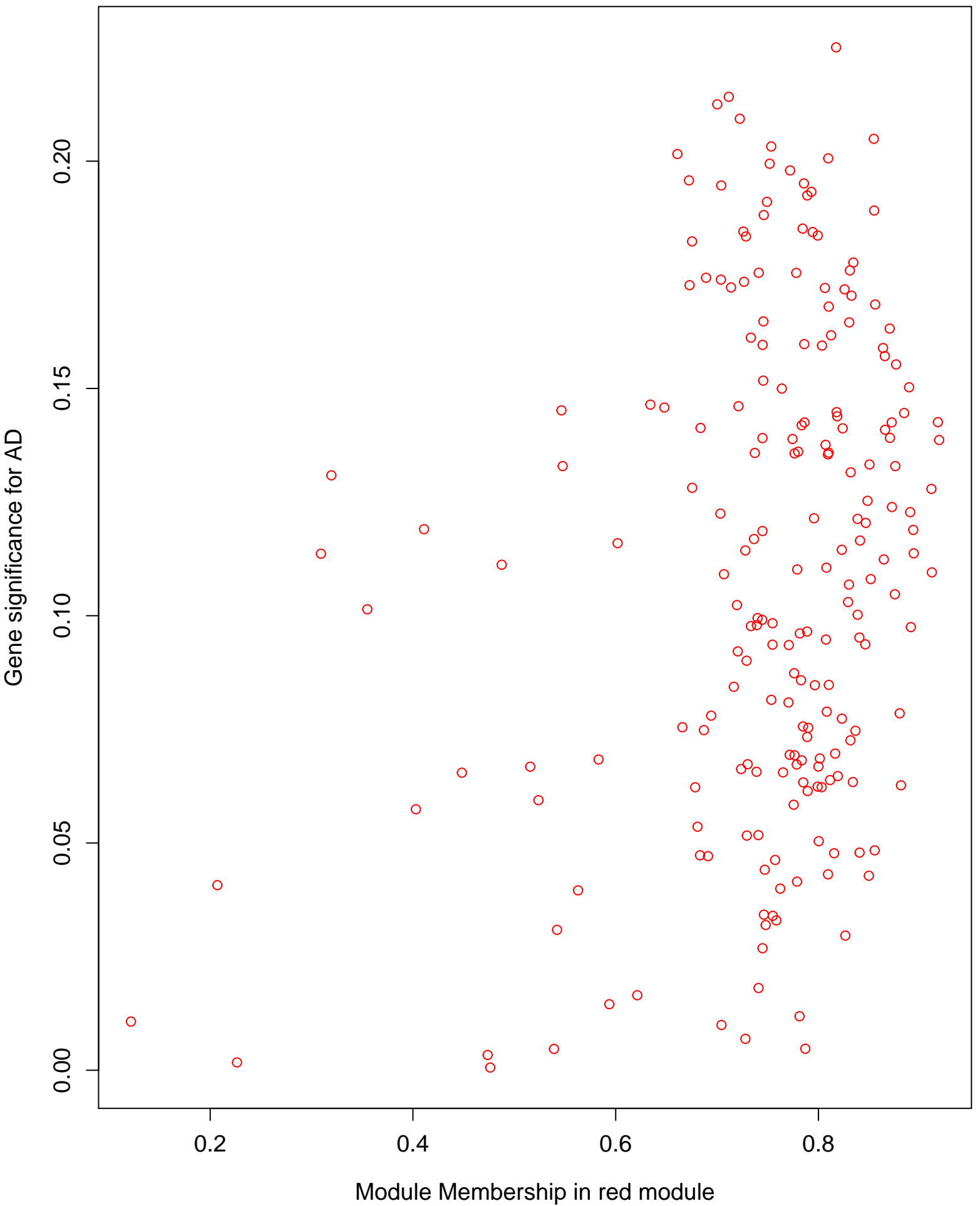

Supplement: Supplementary file 5 [file Data_Sheet_1.ZIP › Supplementary Materials S1/gse63061/RED AD.pdf]

**Module membership vs. gene significance**  
**cor=0.58, p=1e-79**

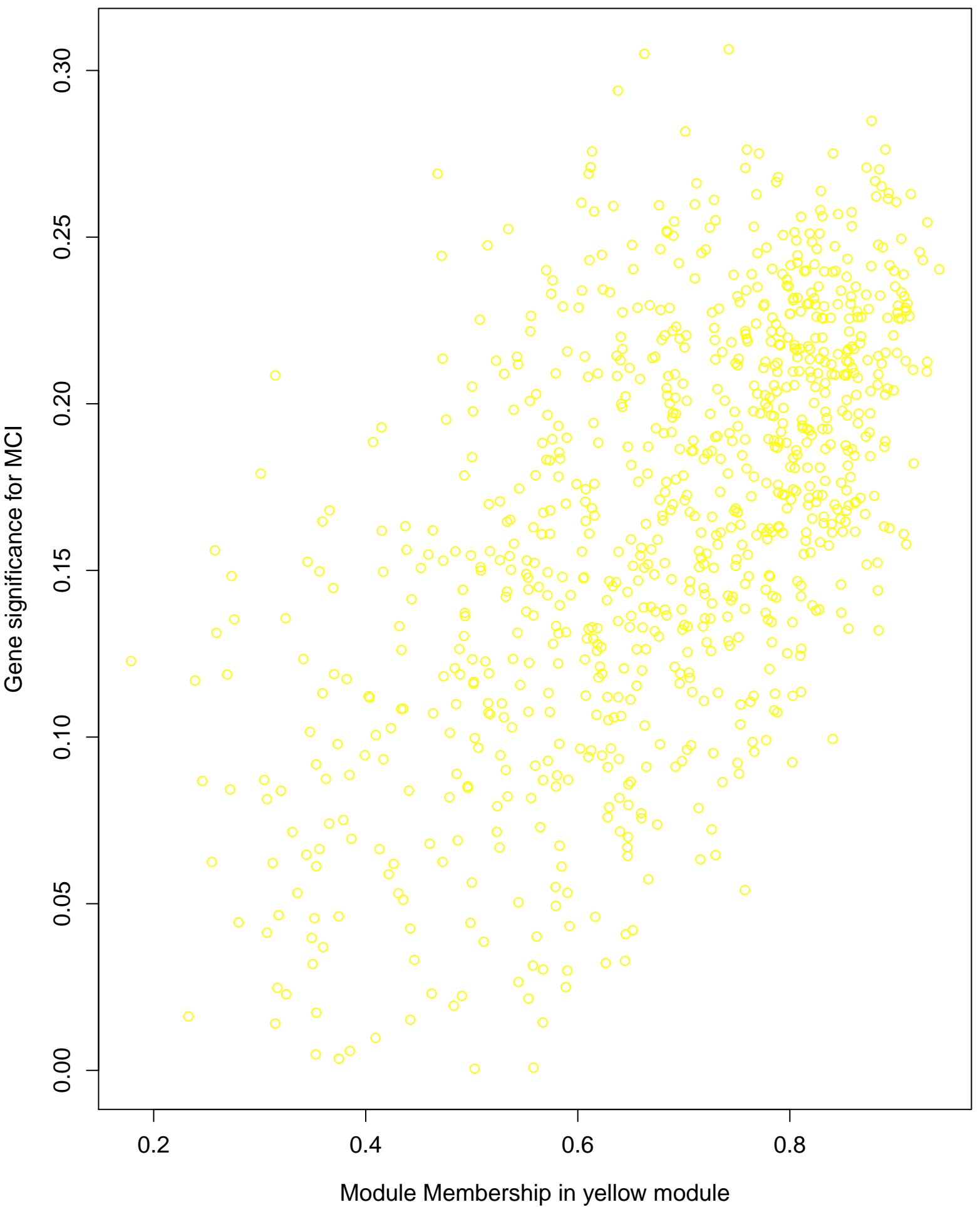

Supplement: Supplementary file 5 [file Data_Sheet_1.ZIP › Supplementary Materials S1/gse63060/yellow mci.pdf]

**Module membership vs. gene significance**  
**cor=0.64, p=2.2e-132**

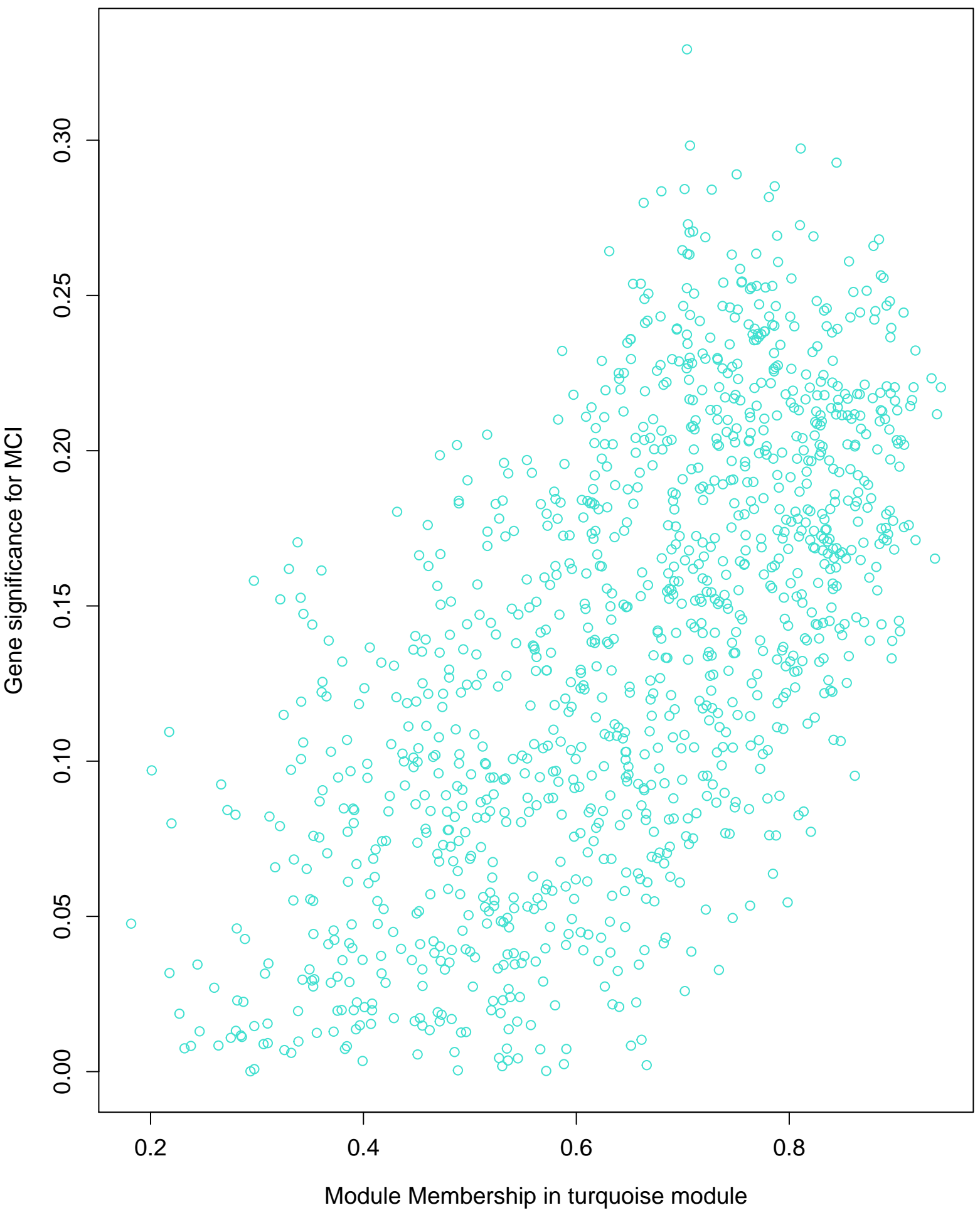

Supplement: Supplementary file 5 [file Data_Sheet_1.ZIP › Supplementary Materials S1/gse63060/turquoise mci.pdf]
