# Supplementary material for: Identification of Temporal Characteristic Networks of Peripheral Blood Changes in Alzheimer’s Disease Based on Weighted Gene Co-expression Network Analysis
Source: Front Aging Neurosci. 2019 May 21;11:83. doi: 10.3389/fnagi.2019.00083 (PMC6537635; doi:10.3389/fnagi.2019.00083)
Supplement: Supplementary file 5 [file Data_Sheet_1.ZIP › Supplementary Materials S1/ROC/ROC GSE63060 BLACK AD-CTL DG BG .pdf]

曲線下的區域

測試結果變數	區域圖	標準錯誤[a]	漸進顯著性[b]	漸進 95% 信賴區間	
				下限	上限
SRGN	.385	.037	.002	.312	.457
WDR6	.657	.036	.000	.587	.727
ECH1	.605	.036	.005	.534	.676
CXXC1	.631	.036	.000	.560	.702
JADE2	.529	.038	.432	.456	.603
TRPV2	.621	.036	.001	.550	.691
PUF60	.585	.037	.023	.512	.658
SBF1	.722	.034	.000	.655	.788
SRRT	.630	.037	.001	.558	.701
NDUFV1	.587	.037	.020	.515	.659
SCAMP3	.552	.037	.163	.480	.624
DDX56	.610	.037	.003	.538	.682
GPS1	.621	.037	.001	.549	.693
TNPO2	.637	.037	.000	.565	.708

測試結果變數: SRGN, WDR6, ECH1, CXXC1, JADE2, TRPV2, PUF60, SBF1, SRRT, NDUFV1, SCAMP3, DDX56, GPS1, TNPO2 在正數實際狀態與負數實際狀態群組之間至少有一個連結空間。統計資料可能有偏差。

a. 在非參數式假設下

b. 空值假設：true 區域 = 0.5
