# Supplementary material for: Identification of Temporal Characteristic Networks of Peripheral Blood Changes in Alzheimer’s Disease Based on Weighted Gene Co-expression Network Analysis
Source: Front Aging Neurosci. 2019 May 21;11:83. doi: 10.3389/fnagi.2019.00083 (PMC6537635; doi:10.3389/fnagi.2019.00083)
Supplement: Supplementary file 5 [file Data_Sheet_1.ZIP › Supplementary Materials S1/ROC/ROC GSE63060 BLACK AD-MCI DG .pdf]

曲線下的區域

測試結果變數	區域圖	標準錯誤[a]	漸進顯著性[b]	漸進 95% 信賴區間	
				下限	上限
SRGN	.656	.038	.000	.582	.731
WDR6	.414	.039	.034	.337	.491
ECH1	.397	.039	.011	.321	.473
CXXC1	.342	.038	.000	.267	.417
JADE2	.332	.038	.000	.257	.406
TRPV2	.367	.039	.001	.291	.443
PUF60	.370	.038	.001	.295	.446
SBF1	.390	.039	.006	.313	.466
SRRT	.428	.039	.077	.351	.506
NDUFV1	.339	.038	.000	.265	.412
SCAMP3	.351	.038	.000	.275	.426
DDX56	.354	.038	.000	.280	.429
GPS1	.342	.037	.000	.269	.416
TNPO2	.364	.038	.001	.290	.439

測試結果變數: SRGN，WDR6，ECH1，CXXC1，JADE2，TRPV2，PUF60，SBF1，SRRT，NDUFV1，SCAMP3，DDX56，GPS1，TNPO2 在正數實際狀態與負數實際狀態群組之間至少有一個連結空間。統計資料可能有偏差。

a. 在非參數式假設下

b. 空值假設：true 區域 = 0.5
