# Supplementary material for: Identification of Temporal Characteristic Networks of Peripheral Blood Changes in Alzheimer’s Disease Based on Weighted Gene Co-expression Network Analysis
Source: Front Aging Neurosci. 2019 May 21;11:83. doi: 10.3389/fnagi.2019.00083 (PMC6537635; doi:10.3389/fnagi.2019.00083)
Supplement: Supplementary file 5 [file Data_Sheet_1.ZIP › Supplementary Materials S1/ROC/ROC GSE63060 BLACK MCI-CTL DG BG.pdf]

曲線下的區域

測試結果變數	區域圖	標準錯誤[a]	漸進顯著性[b]	漸進 95% 信賴區間	
				下限	上限
SRGN	.258	.036	.000	.188	.328
WDR6	.735	.037	.000	.663	.806
ECH1	.711	.038	.000	.637	.786
CXXC1	.762	.035	.000	.692	.831
JADE2	.694	.039	.000	.617	.770
TRPV2	.744	.036	.000	.674	.814
PUF60	.704	.039	.000	.628	.780
SBF1	.794	.033	.000	.730	.858
SRRT	.699	.038	.000	.625	.773
NDUFV1	.736	.037	.000	.664	.808
SCAMP3	.705	.039	.000	.628	.782
DDX56	.734	.036	.000	.663	.805
GPS1	.751	.035	.000	.682	.820
TNPO2	.755	.035	.000	.686	.824

測試結果變數: SRGN, WDR6, ECH1, CXXC1, JADE2, TRPV2, PUF60, SBF1, SRRT, NDUFV1, SCAMP3, DDX56, GPS1, TNPO2 在正數實際狀態與負數實際狀態群組之間至少有一個連結空間。統計資料可能有偏差。

a. 在非參數式假設下

b. 空值假設：true 區域 = 0.5
