# Supplementary material for: Identification of Temporal Characteristic Networks of Peripheral Blood Changes in Alzheimer’s Disease Based on Weighted Gene Co-expression Network Analysis
Source: Front Aging Neurosci. 2019 May 21;11:83. doi: 10.3389/fnagi.2019.00083 (PMC6537635; doi:10.3389/fnagi.2019.00083)
Supplement: Supplementary file 5 [file Data_Sheet_1.ZIP › Supplementary Materials S1/ROC/ROC GSE63060 BLUE AD-CTL DG BG.pdf]

曲線下的區域

測試結果變數	區域圖	標準錯誤[a]	漸進顯著性[b]	漸進 95% 信賴區間	
				下限	上限
REEP5	.347	.036	.000	.276	.418
CEBPZ	.285	.033	.000	.220	.350
CRBN	.319	.034	.000	.251	.386
RDH14	.303	.034	.000	.236	.369
HSPA8	.291	.033	.000	.227	.355
G6PD	.660	.035	.000	.592	.728
STAT3	.654	.035	.000	.585	.723
USP16	.298	.034	.000	.232	.364
DENR	.279	.033	.000	.214	.345
GBA	.692	.034	.000	.625	.758
MITD1	.228	.031	.000	.167	.288
PRRC2A	.650	.035	.000	.581	.719
ACADM	.302	.034	.000	.236	.369
COMMD10	.331	.035	.000	.263	.400
DTX2	.651	.036	.000	.581	.720

測試結果變數: REEP5, CEBPZ, CRBN, RDH14, HSPA8, G6PD, STAT3, USP16, DENR, GBA, MITD1, PRRC2A, ACADM, COMMD10, DTX2 在正數實際狀態與負數實際狀態群組之間至少有一個連結空間。統計資料可能有偏差。

a. 在非參數式假設下

b. 空值假設：true 區域 = 0.5
