# Supplementary material for: Identification of Temporal Characteristic Networks of Peripheral Blood Changes in Alzheimer’s Disease Based on Weighted Gene Co-expression Network Analysis
Source: Front Aging Neurosci. 2019 May 21;11:83. doi: 10.3389/fnagi.2019.00083 (PMC6537635; doi:10.3389/fnagi.2019.00083)
Supplement: Supplementary file 5 [file Data_Sheet_1.ZIP › Supplementary Materials S1/ROC/ROC GSE63060 BLUE AD-MCI DG BG.pdf]

曲線下的區域

測試結果變數	區域圖	標準錯誤[a]	漸進顯著性[b]	漸進 95% 信賴區間	
				下限	上限
REEP5	.633	.039	.001	.557	.709
CEBPZ	.557	.040	.163	.479	.634
CRBN	.597	.039	.017	.520	.674
RDH14	.575	.040	.064	.497	.653
HSPA8	.497	.040	.940	.419	.575
G6PD	.452	.040	.239	.375	.530
STAT3	.496	.041	.923	.417	.576
USP16	.567	.039	.100	.489	.644
DENR	.561	.040	.133	.483	.639
GBA	.458	.040	.301	.380	.536
MITD1	.587	.040	.033	.509	.664
PRRC2A	.403	.040	.016	.324	.481
ACADM	.562	.041	.123	.483	.642
COMMD10	.587	.040	.032	.509	.665
DTX2	.449	.040	.207	.370	.528

測試結果變數: REEP5, CEBPZ, HSPA8, G6PD, STAT3, USP16, DENR, GBA, MITD1, PRRC2A, DTX2 在正數實際狀態與負數實際狀態群組之間至少有一個連結空間。統計資料可能有偏差。

a. 在非參數式假設下

b. 空值假設：true 區域 = 0.5
