# Supplementary material for: Identification of Temporal Characteristic Networks of Peripheral Blood Changes in Alzheimer’s Disease Based on Weighted Gene Co-expression Network Analysis
Source: Front Aging Neurosci. 2019 May 21;11:83. doi: 10.3389/fnagi.2019.00083 (PMC6537635; doi:10.3389/fnagi.2019.00083)
Supplement: Supplementary file 5 [file Data_Sheet_1.ZIP › Supplementary Materials S1/ROC/ROC GSE63060 BLUE MCI-CTL DG BG.pdf]

曲線下的區域

測試結果變數	區域圖	標準錯誤[a]	漸進顯著性[b]	漸進 95% 信賴區間	
				下限	上限
REEP5	.242	.035	.000	.172	.311
CEBPZ	.230	.034	.000	.163	.297
CRBN	.220	.034	.000	.154	.287
RDH14	.230	.035	.000	.162	.298
HSPA8	.271	.038	.000	.196	.345
G6PD	.715	.038	.000	.640	.790
STAT3	.668	.040	.000	.590	.745
USP16	.231	.035	.000	.163	.300
DENR	.233	.035	.000	.165	.301
GBA	.736	.037	.000	.664	.808
MITD1	.173	.031	.000	.112	.234
PRRC2A	.735	.037	.000	.662	.808
ACADM	.257	.038	.000	.184	.331
COMMD10	.261	.037	.000	.188	.334
DTX2	.694	.039	.000	.618	.769

測試結果變數: REEP5，CRBN，RDH14，HSPA8，G6PD，STAT3，USP16，DENR，GBA，MITD1，PRRC2A，ACADM，DTX2 在正數實際狀態與負數實際狀態群組之間至少有一個連結空間。統計資料可能有偏差。

a. 在非參數式假設下

b. 空值假設：true 區域 = 0.5
