# Supplementary material for: Identification of Temporal Characteristic Networks of Peripheral Blood Changes in Alzheimer’s Disease Based on Weighted Gene Co-expression Network Analysis
Source: Front Aging Neurosci. 2019 May 21;11:83. doi: 10.3389/fnagi.2019.00083 (PMC6537635; doi:10.3389/fnagi.2019.00083)
Supplement: Supplementary file 5 [file Data_Sheet_1.ZIP › Supplementary Materials S1/ROC/ROC GSE63060 BROWN AD-CTL DG BG.pdf]

曲線下的區域

測試結果變數	區域圖	標準錯誤[a]	漸進顯著性[b]	漸進 95% 信賴區間	
				下限	上限
TOMM7	.229	.030	.000	.170	.288
RPS3A	.341	.036	.000	.270	.411
RPS17	.281	.034	.000	.215	.348
NDUFB3	.282	.034	.000	.215	.349
RPS27	.299	.035	.000	.231	.367
LSM3	.272	.034	.000	.206	.337
PSMA4	.268	.033	.000	.204	.333
RPL17	.244	.031	.000	.183	.305
PSMA6	.327	.036	.000	.256	.398
DPM1	.256	.032	.000	.193	.319
TMEM126B	.318	.036	.000	.249	.388
MRPL22	.270	.034	.000	.203	.337
ATP5J	.254	.033	.000	.190	.318
RPL26L1	.302	.035	.000	.233	.371
LARP7	.272	.033	.000	.207	.337

測試結果變數: TOMM7, RPS3A, RPS17, NDUFB3, RPS27, LSM3, PSMA4, RPL17, PSMA6, DPM1, MRPL22, ATP5J 在正數實際狀態與負數實際狀態群組之間至少有一個連結空間。統計資料可能有偏差。

a. 在非參數式假設下

b. 空值假設：true 區域 = 0.5
