# Supplementary material for: Identification of Temporal Characteristic Networks of Peripheral Blood Changes in Alzheimer’s Disease Based on Weighted Gene Co-expression Network Analysis
Source: Front Aging Neurosci. 2019 May 21;11:83. doi: 10.3389/fnagi.2019.00083 (PMC6537635; doi:10.3389/fnagi.2019.00083)
Supplement: Supplementary file 5 [file Data_Sheet_1.ZIP › Supplementary Materials S1/ROC/ROC GSE63060 BROWN AD-MCI DG BG.pdf]

曲線下的區域

測試結果變數	區域圖	標準錯誤[a]	漸進顯著性[b]	漸進 95% 信賴區間	
				下限	上限
TOMM7	.536	.040	.373	.457	.615
RPS3A	.586	.040	.035	.507	.664
RPS17	.566	.040	.103	.488	.645
NDUFB3	.603	.040	.011	.525	.681
RPS27	.568	.040	.092	.490	.646
LSM3	.579	.040	.051	.500	.658
PSMA4	.566	.041	.101	.487	.646
RPL17	.495	.040	.896	.416	.574
PSMA6	.626	.039	.002	.549	.702
DPM1	.593	.040	.021	.515	.671
TMEM126B	.607	.039	.008	.530	.683
MRPL22	.540	.040	.328	.461	.619
ATP5J	.508	.041	.837	.427	.589
RPL26L1	.576	.040	.059	.498	.655
LARP7	.547	.041	.243	.467	.627

測試結果變數: TOMM7, RPS3A, RPS17, RPS27, PSMA4, RPL17, PSMA6, DPM1, MRPL22, RPL26L1, LARP7 在正數實際狀態與負數實際狀態群組之間至少有一個連結空間。統計資料可能有偏差。

a. 在非參數式假設下

b. 空值假設：true 區域 = 0.5
