# Supplementary material for: Identification of Temporal Characteristic Networks of Peripheral Blood Changes in Alzheimer’s Disease Based on Weighted Gene Co-expression Network Analysis
Source: Front Aging Neurosci. 2019 May 21;11:83. doi: 10.3389/fnagi.2019.00083 (PMC6537635; doi:10.3389/fnagi.2019.00083)
Supplement: Supplementary file 5 [file Data_Sheet_1.ZIP › Supplementary Materials S1/ROC/ROC GSE63060 BROWN MCI-CTL DG BG.pdf]

曲線下的區域

測試結果變數	區域圖	標準錯誤[a]	漸進顯著性[b]	漸進 95% 信賴區間	
				下限	上限
TOMM7	.207	.033	.000	.143	.271
RPS3A	.258	.036	.000	.187	.329
RPS17	.235	.035	.000	.167	.303
NDUFB3	.221	.034	.000	.155	.287
RPS27	.248	.035	.000	.179	.317
LSM3	.224	.034	.000	.158	.290
PSMA4	.225	.034	.000	.159	.291
RPL17	.245	.036	.000	.175	.316
PSMA6	.239	.035	.000	.171	.307
DPM1	.180	.031	.000	.120	.240
TMEM126B	.236	.035	.000	.167	.304
MRPL22	.247	.035	.000	.177	.316
ATP5J	.252	.035	.000	.183	.321
RPL26L1	.249	.035	.000	.180	.318
LARP7	.240	.035	.000	.172	.308

測試結果變數: TOMM7，RPS3A，RPS17，RPS27，PSMA4，RPL17，PSMA6，MRPL22，RPL26L1 在正數實際狀態與負數實際狀態群組之間至少有一個連結空間。統計資料可能有偏差。

a. 在非參數式假設下

b. 空值假設：true 區域 = 0.5
