# Supplementary material for: Identification of Temporal Characteristic Networks of Peripheral Blood Changes in Alzheimer’s Disease Based on Weighted Gene Co-expression Network Analysis
Source: Front Aging Neurosci. 2019 May 21;11:83. doi: 10.3389/fnagi.2019.00083 (PMC6537635; doi:10.3389/fnagi.2019.00083)
Supplement: Supplementary file 5 [file Data_Sheet_1.ZIP › Supplementary Materials S1/ROC/ROC GSE63060 PINK AD-CTL DG BG.pdf]

曲線下的區域

測試結果變數	區域圖	標準錯誤[a]	漸進顯著性[b]	漸進 95% 信賴區間	
				下限	上限
FPR2	.646	.036	.000	.576	.715
REPS2	.591	.036	.015	.519	.662
MXD1	.574	.036	.047	.503	.646
PFKFB4	.667	.035	.000	.599	.735
MANSC1	.590	.037	.016	.518	.662
LAMP2	.615	.036	.002	.544	.685
RNF149	.544	.037	.237	.472	.617
MSRB1	.602	.036	.006	.531	.673
FCGR2A	.541	.037	.274	.468	.613
SVIL	.582	.037	.029	.510	.653
ZNF746	.665	.035	.000	.597	.733
SIRPA	.618	.036	.002	.547	.688
DENND5A	.588	.036	.019	.516	.659
P6V1B2	.530	.037	.420	.457	.604
NDEL1	.561	.037	.104	.489	.632

測試結果變數: FPR2，REPS2，MXD1，PFKFB4，MANSC1，LAMP2，RNF149，MSRB1，FCGR2A，SVIL，ZNF746，SIRPA，DENND5A，P6V1B2，NDEL1 在正數實際狀態與負數實際狀態群組之間至少有一個連結空間。統計資料可能有偏差。

a. 在非參數式假設下

b. 空值假設：true 區域 = 0.5
