# Supplementary material for: Identification of Temporal Characteristic Networks of Peripheral Blood Changes in Alzheimer’s Disease Based on Weighted Gene Co-expression Network Analysis
Source: Front Aging Neurosci. 2019 May 21;11:83. doi: 10.3389/fnagi.2019.00083 (PMC6537635; doi:10.3389/fnagi.2019.00083)
Supplement: Supplementary file 5 [file Data_Sheet_1.ZIP › Supplementary Materials S1/ROC/ROC GSE63060 PINK AD-MCI DG BG.pdf]

曲線下的區域

測試結果變數	區域圖	標準錯誤[a]	漸進顯著性[b]	漸進 95% 信賴區間	
				下限	上限
FPR2	.546	.041	.252	.466	.627
REPS2	.517	.041	.669	.438	.597
MXD1	.618	.039	.004	.542	.694
PFKFB4	.553	.040	.192	.475	.630
MANSC1	.526	.041	.515	.446	.607
LAMP2	.573	.040	.070	.494	.652
RNF149	.588	.040	.030	.509	.667
MSRB1	.592	.039	.023	.515	.669
FCGR2A	.526	.041	.514	.446	.606
SVIL	.549	.041	.227	.469	.629
ZNF746	.517	.041	.680	.437	.597
SIRPA	.486	.040	.735	.408	.565
DENND5A	.583	.039	.041	.506	.660
P6V1B2	.528	.042	.486	.446	.610
NDEL1	.530	.041	.454	.451	.610

測試結果變數: FPR2, REPS2, MXD1, PFKFB4, MANSC1, LAMP2, RNF149, MSRB1, SVIL, ZNF746, SIRPA, DENND5A, P6V1B2, NDEL1 在正數實際狀態與負數實際狀態群組之間至少有一個連結空間。統計資料可能有偏差。

a. 在非參數式假設下

b. 空值假設：true 區域 = 0.5
