# Supplementary material for: Identification of Temporal Characteristic Networks of Peripheral Blood Changes in Alzheimer’s Disease Based on Weighted Gene Co-expression Network Analysis
Source: Front Aging Neurosci. 2019 May 21;11:83. doi: 10.3389/fnagi.2019.00083 (PMC6537635; doi:10.3389/fnagi.2019.00083)
Supplement: Supplementary file 5 [file Data_Sheet_1.ZIP › Supplementary Materials S1/ROC/ROC GSE63060 PINK MCI-CTL DG BG.pdf]

曲線下的區域

測試結果變數	區域圖	標準錯誤[a]	漸進顯著性[b]	漸進 95% 信賴區間	
				下限	上限
FPR2	.595	.042	.027	.512	.678
REPS2	.579	.043	.067	.495	.662
MXD1	.445	.044	.205	.359	.532
PFKFB4	.633	.041	.002	.553	.713
MANSC1	.560	.043	.165	.476	.644
LAMP2	.532	.044	.462	.446	.617
RNF149	.440	.044	.163	.354	.526
MSRB1	.506	.043	.890	.421	.591
FCGR2A	.512	.044	.773	.427	.598
SVIL	.523	.045	.601	.435	.610
ZNF746	.645	.041	.001	.564	.726
SIRPA	.641	.041	.001	.560	.721
DENND5A	.501	.044	.986	.415	.586
P6V1B2	.501	.044	.987	.414	.587
NDEL1	.525	.044	.556	.439	.611

測試結果變數: FPR2, REPS2, MXD1, PFKFB4, MANSC1, LAMP2, RNF149, MSRB1, FCGR2A, SVIL, ZNF746, SIRPA, DENND5A, P6V1B2, NDEL1 在正數實際狀態與負數實際狀態群組之間至少有一個連結空間。統計資料可能有偏差。

a. 在非參數式假設下

b. 空值假設：true 區域 = 0.5
