# Supplementary material for: Identification of Temporal Characteristic Networks of Peripheral Blood Changes in Alzheimer’s Disease Based on Weighted Gene Co-expression Network Analysis
Source: Front Aging Neurosci. 2019 May 21;11:83. doi: 10.3389/fnagi.2019.00083 (PMC6537635; doi:10.3389/fnagi.2019.00083)
Supplement: Supplementary file 5 [file Data_Sheet_1.ZIP › Supplementary Materials S1/ROC/ROC GSE63060 PINK MCI-CTL LH BG .pdf]

曲線下的區域

測試結果變數:　預測機率

			漸進 95% 信賴區間	
區域圖	標準錯誤[a]	漸進顯著性[b]	下限	上限
.809	.031	.000	.747	.870

a. 在非參數式假設下

b. 空值假設：true 區域 = 0.5
