# Supplementary material for: Identification of Temporal Characteristic Networks of Peripheral Blood Changes in Alzheimer’s Disease Based on Weighted Gene Co-expression Network Analysis
Source: Front Aging Neurosci. 2019 May 21;11:83. doi: 10.3389/fnagi.2019.00083 (PMC6537635; doi:10.3389/fnagi.2019.00083)
Supplement: Supplementary file 5 [file Data_Sheet_1.ZIP › Supplementary Materials S1/ROC/ROC GSE63060 RED AD-CTL DG BG.pdf]

曲線下的區域

測試結果變數	區域圖	標準錯誤[a]	漸進顯著性[b]	漸進 95% 信賴區間	
				下限	上限
CLNS1A	.311	.035	.000	.243	.379
CRBN	.392	.036	.004	.321	.463
NDUFB5	.347	.036	.000	.278	.417
RALA	.322	.035	.000	.253	.390
CAMLG	.395	.036	.005	.324	.467
DDX1	.272	.033	.000	.207	.336
PPP3CB	.381	.036	.001	.310	.452
EBAG9	.311	.034	.000	.243	.378
SNRPF	.354	.036	.000	.285	.424
GPN1	.313	.034	.000	.245	.380
AK3	.344	.035	.000	.274	.413
CCDC25	.364	.036	.000	.293	.434
MTERF3	.309	.034	.000	.242	.377
PDCD2	.385	.037	.002	.313	.457

測試結果變數: CLNS1A, CRBN, NDUFB5, RALA, CAMLG, DDX1, PPP3CB, EBAG9, SNRPF, GPN1, AK3, CCDC25, MTERF3, PDCD2 在正數實際狀態與負數實際狀態群組之間至少有一個連結空間。統計資料可能有偏差。

a. 在非參數式假設下

b. 空值假設：true 區域 = 0.5
