# Supplementary material for: Identification of Temporal Characteristic Networks of Peripheral Blood Changes in Alzheimer’s Disease Based on Weighted Gene Co-expression Network Analysis
Source: Front Aging Neurosci. 2019 May 21;11:83. doi: 10.3389/fnagi.2019.00083 (PMC6537635; doi:10.3389/fnagi.2019.00083)
Supplement: Supplementary file 5 [file Data_Sheet_1.ZIP › Supplementary Materials S1/ROC/ROC GSE63060 RED AD-MCI DG BG.pdf]

曲線下的區域

測試結果變數	區域圖	標準錯誤[a]	漸進顯著性[b]	漸進 95% 信賴區間	
				下限	上限
CLNS1A	.550	.040	.217	.472	.628
CRBN	.549	.040	.226	.471	.627
NDUFB5	.572	.040	.077	.494	.650
RALA	.563	.040	.122	.485	.640
CAMLG	.541	.040	.306	.463	.620
DDX1	.522	.040	.579	.444	.601
PPP3CB	.566	.040	.101	.489	.644
EBAG9	.541	.040	.306	.464	.619
SNRPF	.541	.040	.316	.461	.620
GPN1	.469	.040	.441	.390	.548
AK3	.521	.041	.599	.441	.601
CCDC25	.535	.041	.392	.455	.614
MTERF3	.513	.040	.741	.434	.593
PDCD2	.525	.040	.545	.447	.602

測試結果變數: CLNS1A, CRBN, NDUFB5, RALA, CAMLG, DDX1, PPP3CB, EBAG9, SNRPF, GPN1, AK3, CCDC25, MTERF3, PDCD2 在正數實際狀態與負數實際狀態群組之間至少有一個連結空間。統計資料可能有偏差。

a. 在非參數式假設下

b. 空值假設：true 區域 = 0.5
