# Supplementary material for: Identification of Temporal Characteristic Networks of Peripheral Blood Changes in Alzheimer’s Disease Based on Weighted Gene Co-expression Network Analysis
Source: Front Aging Neurosci. 2019 May 21;11:83. doi: 10.3389/fnagi.2019.00083 (PMC6537635; doi:10.3389/fnagi.2019.00083)
Supplement: Supplementary file 5 [file Data_Sheet_1.ZIP › Supplementary Materials S1/ROC/ROC GSE63060 RED MCI-CTL DG.pdf]

曲線下的區域

| 測試結果變數 | 區域圖 | 標準錯誤[a] | 漸進顯著性[b] | 漸進 95% 信賴區間 | |
|---|---|---|---|---|---|
| | | | | 下限 | 上限 |
| CLNS1A | .259 | .037 | .000 | .186 | .333 |
| CRBN | .338 | .041 | .000 | .257 | .418 |
| NDUFB5 | .288 | .039 | .000 | .212 | .365 |
| RALA | .269 | .038 | .000 | .195 | .343 |
| CAMLG | .354 | .041 | .001 | .273 | .435 |
| DDX1 | .241 | .035 | .000 | .172 | .310 |
| PPP3CB | .317 | .040 | .000 | .240 | .395 |
| EBAG9 | .262 | .037 | .000 | .190 | .334 |
| SNRPF | .320 | .040 | .000 | .242 | .397 |
| GPN1 | .339 | .040 | .000 | .261 | .418 |
| AK3 | .330 | .040 | .000 | .251 | .409 |
| CCDC25 | .330 | .040 | .000 | .252 | .408 |
| MTERF3 | .294 | .039 | .000 | .217 | .370 |
| PDCD2 | .351 | .041 | .001 | .271 | .431 |

測試結果變數: CLNS1A，CRBN，NDUFB5，RALA，CAMLG，DDX1，PPP3CB，EBAG9，SNRPF，GPN1，AK3，CCDC25，MTERF3，PDCD2 在正數實際狀態與負數實際狀態群組之間至少有一個連結空間。統計資料可能有偏差。

a. 在非參數式假設下

b. 空值假設：true 區域 = 0.5
