# Supplementary material for: Identification of Temporal Characteristic Networks of Peripheral Blood Changes in Alzheimer’s Disease Based on Weighted Gene Co-expression Network Analysis
Source: Front Aging Neurosci. 2019 May 21;11:83. doi: 10.3389/fnagi.2019.00083 (PMC6537635; doi:10.3389/fnagi.2019.00083)
Supplement: Supplementary file 5 [file Data_Sheet_1.ZIP › Supplementary Materials S1/ROC/ROC GSE63060 TURQUIOSE AD-MCI DG BG.pdf]

曲線下的區域

測試結果變數	區域圖	標準錯誤[a]	漸進顯著性[b]	漸進 95% 信賴區間	
				下限	上限
ACTR3	.610	.040	.007	.532	.688
MTPN	.613	.040	.005	.535	.690
ADD3	.566	.040	.103	.487	.645
SACM1L	.619	.038	.003	.544	.695
WIPF1	.578	.039	.055	.501	.655
CMPK1	.600	.039	.014	.524	.676
FAM49B	.582	.039	.042	.505	.659
PHIP	.614	.039	.005	.538	.690
UBLCP	.634	.038	.001	.559	.709
STK26	.642	.038	.000	.567	.717
LUC7L3	.586	.039	.034	.509	.662
ANKRD10	.596	.040	.018	.518	.673
GIMAP2	.640	.040	.001	.562	.717
ATF4	.562	.040	.129	.483	.640

測試結果變數: ACTR3，MTPN，ADD3，SACM1L，WIPF1，UBLCP，STK26，GIMAP2，ATF4 在正數實際狀態與負數實際狀態群組之間至少有一個連結空間。統計資料可能有偏差。

a. 在非參數式假設下

b. 空值假設：true 區域 = 0.5
