# Supplementary material for: Identification of Temporal Characteristic Networks of Peripheral Blood Changes in Alzheimer’s Disease Based on Weighted Gene Co-expression Network Analysis
Source: Front Aging Neurosci. 2019 May 21;11:83. doi: 10.3389/fnagi.2019.00083 (PMC6537635; doi:10.3389/fnagi.2019.00083)
Supplement: Supplementary file 5 [file Data_Sheet_1.ZIP › Supplementary Materials S1/ROC/ROC GSE63060 TURQUIOSE MCI-CTL DG BG.pdf]

曲線下的區域

測試結果變數	區域圖	標準錯誤[a]	漸進顯著性[b]	漸進 95% 信賴區間	
				下限	上限
ACTR3	.309	.039	.000	.233	.385
MTPN	.303	.039	.000	.227	.379
ADD3	.406	.042	.029	.324	.488
SACM1L	.316	.039	.000	.240	.393
WIPF1	.388	.042	.009	.306	.470
CMPK1	.303	.038	.000	.228	.378
FAM49B	.349	.040	.000	.270	.428
PHIP	.305	.039	.000	.229	.381
UBLCP	.285	.038	.000	.211	.359
STK26	.306	.039	.000	.228	.383
LUC7L3	.341	.040	.000	.262	.420
ANKRD10	.369	.041	.002	.288	.450
GIMAP2	.283	.038	.000	.208	.357
ATF4	.392	.042	.013	.311	.474

測試結果變數: MTPN，ADD3，SACM1L，WIPF1，FAM49B，UBLCP，LUC7L3，GIMAP2，ATF4 在正數實際狀態與負數實際狀態群組之間至少有一個連結空間。統計資料可能有偏差。

a. 在非參數式假設下

b. 空值假設：true 區域 = 0.5
