# Supplementary material for: Identification of Temporal Characteristic Networks of Peripheral Blood Changes in Alzheimer’s Disease Based on Weighted Gene Co-expression Network Analysis
Source: Front Aging Neurosci. 2019 May 21;11:83. doi: 10.3389/fnagi.2019.00083 (PMC6537635; doi:10.3389/fnagi.2019.00083)
Supplement: Supplementary file 5 [file Data_Sheet_1.ZIP › Supplementary Materials S1/ROC/ROC GSE63060 YELLOW AD-CTL DG BG.pdf]

曲線下的區域

測試結果變數	區域圖	標準錯誤[a]	漸進顯著性[b]	漸進 95% 信賴區間	
				下限	上限
ARGLU1	.377	.036	.001	.308	.447
PPM1B	.316	.034	.000	.248	.383
THAP12	.400	.036	.007	.329	.471
SNRK	.380	.037	.001	.308	.452
CD58	.365	.036	.000	.295	.436
ANKRD49	.346	.035	.000	.278	.415
ST8SIA4	.408	.037	.014	.337	.480
RPS6KB1	.359	.035	.000	.289	.428
PPP2CA	.397	.036	.006	.326	.469
CNIH1	.587	.037	.020	.514	.660
BCLAF1	.415	.037	.023	.343	.487
PCNX4	.376	.036	.001	.306	.446
TRIM33	.473	.037	.474	.400	.546
UPF2	.397	.037	.006	.325	.469

測試結果變數: ARGLU1, PPM1B, THAP12, SNRK, CD58, ST8SIA4, RPS6KB1, PPP2CA, CNIH1, BCLAF1, TRIM33, UPF2 在正數實際狀態與負數實際狀態群組之間至少有一個連結空間。統計資料可能有偏差。

a. 在非參數式假設下

b. 空值假設：true 區域 = 0.5
