# Supplementary material for: Identification of Temporal Characteristic Networks of Peripheral Blood Changes in Alzheimer’s Disease Based on Weighted Gene Co-expression Network Analysis
Source: Front Aging Neurosci. 2019 May 21;11:83. doi: 10.3389/fnagi.2019.00083 (PMC6537635; doi:10.3389/fnagi.2019.00083)
Supplement: Supplementary file 5 [file Data_Sheet_1.ZIP › Supplementary Materials S1/ROC/ROC GSE63060 YELLOW AD-MCI DG BG.pdf]

曲線下的區域

測試結果變數	區域圖	標準錯誤[a]	漸進顯著性[b]	漸進 95% 信賴區間	
				下限	上限
ARGLU1	.611	.038	.006	.536	.687
PPM1B	.584	.040	.038	.506	.662
THAP12	.605	.039	.009	.529	.682
SNRK	.599	.040	.015	.521	.677
CD58	.633	.039	.001	.557	.708
ANKRD49	.606	.039	.009	.529	.683
ST8SIA4	.628	.038	.002	.553	.704
RPS6KB1	.601	.039	.012	.525	.677
PPP2CA	.613	.040	.005	.534	.691
CNIH1	.637	.039	.001	.560	.714
BCLAF1	.604	.039	.010	.527	.681
PCNX4	.581	.039	.044	.504	.659
TRIM33	.642	.038	.000	.568	.717
UPF2	.579	.040	.051	.501	.657

測試結果變數: ARGLU1, PPM1B, THAP12, SNRK, CD58, RPS6KB1, PPP2CA, CNIH1, BCLAF1, PCNX4, TRIM33, UPF2 在正數實際狀態與負數實際狀態群組之間至少有一個連結空間。統計資料可能有偏差。

a. 在非參數式假設下

b. 空值假設：true 區域 = 0.5
