# Supplementary material for: Identification of Temporal Characteristic Networks of Peripheral Blood Changes in Alzheimer’s Disease Based on Weighted Gene Co-expression Network Analysis
Source: Front Aging Neurosci. 2019 May 21;11:83. doi: 10.3389/fnagi.2019.00083 (PMC6537635; doi:10.3389/fnagi.2019.00083)
Supplement: Supplementary file 5 [file Data_Sheet_1.ZIP › Supplementary Materials S1/ROC/ROC GSE63060 YELLOW MCI-CTL DG BG.pdf]

曲線下的區域

測試結果變數	區域圖	標準錯誤[a]	漸進顯著性[b]	漸進 95% 信賴區間	
				下限	上限
ARGLU1	.252	.037	.000	.179	.325
PPM1B	.244	.036	.000	.174	.313
THAP12	.296	.040	.000	.219	.374
SNRK	.293	.038	.000	.218	.368
CD58	.247	.036	.000	.177	.318
ANKRD49	.239	.037	.000	.166	.312
ST8SIA4	.280	.038	.000	.206	.354
RPS6KB1	.254	.037	.000	.182	.327
PPP2CA	.298	.039	.000	.221	.375
CNIH1	.455	.043	.301	.372	.539
BCLAF1	.317	.040	.000	.238	.396
PCNX4	.297	.039	.000	.220	.374
TRIM33	.329	.040	.000	.251	.407
UPF2	.324	.040	.000	.246	.401

測試結果變數: ARGLU1, PPM1B, THAP12, SNRK, CD58, ANKRD49, PPP2CA, CNIH1, BCLAF1, PCNX4, TRIM33, UPF2 在正數實際狀態與負數實際狀態群組之間至少有一個連結空間。統計資料可能有偏差。

a. 在非參數式假設下

b. 空值假設：true 區域 = 0.5
