# Supplementary material for: Identification of Temporal Characteristic Networks of Peripheral Blood Changes in Alzheimer’s Disease Based on Weighted Gene Co-expression Network Analysis
Source: Front Aging Neurosci. 2019 May 21;11:83. doi: 10.3389/fnagi.2019.00083 (PMC6537635; doi:10.3389/fnagi.2019.00083)
Supplement: Supplementary file 5 [file Data_Sheet_1.ZIP › Supplementary Materials S1/ROC/ROC GSE63061 BLACK AD-CTL DG BG.pdf]

曲線下的區域

測試結果變數	區域圖	標準錯誤[a]	漸進顯著性[b]	漸進 95% 信賴區間	
				下限	上限
ECH1	.547	.035	.180	.479	.615
TNPO2	.584	.035	.016	.517	.652
WDR6	.579	.034	.023	.512	.647
DDX56	.531	.035	.379	.462	.600
CXXC1	.549	.035	.161	.481	.618
SBF1	.616	.034	.001	.549	.683
PUF60	.541	.035	.236	.473	.610
NDUFV1	.555	.035	.116	.487	.623
SCAMP3	.567	.035	.055	.499	.635
JADE2	.493	.035	.831	.424	.561
GPS1	.598	.035	.005	.530	.666
TRPV2	.586	.034	.014	.519	.654
SRGN	.421	.034	.023	.353	.488
SRRT	.618	.034	.001	.552	.684

a. 在非參數式假設下

b. 空值假設：true 區域 = 0.5
