# Supplementary material for: Identification of Temporal Characteristic Networks of Peripheral Blood Changes in Alzheimer’s Disease Based on Weighted Gene Co-expression Network Analysis
Source: Front Aging Neurosci. 2019 May 21;11:83. doi: 10.3389/fnagi.2019.00083 (PMC6537635; doi:10.3389/fnagi.2019.00083)
Supplement: Supplementary file 5 [file Data_Sheet_1.ZIP › Supplementary Materials S1/ROC/ROC GSE63061 BLACK AD-MCI DG BG .pdf]

曲線下的區域

測試結果變數	區域圖	標準錯誤[a]	漸進顯著性[b]	漸進 95% 信賴區間	
				下限	上限
ECH1	.512	.037	.745	.440	.584
TNPO2	.477	.037	.537	.404	.550
WDR6	.473	.037	.463	.400	.546
DDX56	.498	.037	.948	.424	.571
CXXC1	.456	.037	.233	.383	.529
SBF1	.483	.037	.641	.410	.556
PUF60	.466	.037	.364	.394	.539
NDUFV1	.476	.037	.521	.404	.548
SCAMP3	.480	.037	.581	.407	.552
JADE2	.410	.037	.015	.338	.482
GPS1	.494	.037	.869	.421	.567
TRPV2	.467	.037	.378	.395	.540
SRGN	.478	.037	.546	.404	.551
SRRT	.505	.037	.894	.432	.578

a. 在非參數式假設下

b. 空值假設：true 區域 = 0.5
