# Supplementary material for: Identification of Temporal Characteristic Networks of Peripheral Blood Changes in Alzheimer’s Disease Based on Weighted Gene Co-expression Network Analysis
Source: Front Aging Neurosci. 2019 May 21;11:83. doi: 10.3389/fnagi.2019.00083 (PMC6537635; doi:10.3389/fnagi.2019.00083)
Supplement: Supplementary file 5 [file Data_Sheet_1.ZIP › Supplementary Materials S1/ROC/ROC GSE63061 BLACK MCI-CTL DG BG.pdf]

曲線下的區域

測試結果變數	區域圖	標準錯誤[a]	漸進顯著性[b]	漸進 95% 信賴區間	
				下限	上限
ECH1	.537	.037	.319	.464	.610
TNPO2	.603	.036	.006	.532	.674
WDR6	.604	.036	.005	.533	.676
DDX56	.532	.037	.391	.459	.605
CXXC1	.590	.037	.016	.519	.662
SBF1	.629	.036	.001	.559	.699
PUF60	.575	.037	.045	.503	.647
NDUFV1	.578	.037	.036	.507	.650
SCAMP3	.585	.036	.023	.513	.656
JADE2	.581	.037	.030	.509	.653
GPS1	.594	.037	.012	.522	.665
TRPV2	.617	.036	.002	.546	.689
SRGN	.443	.037	.128	.370	.516
SRRT	.607	.037	.004	.535	.679

a. 在非參數式假設下

b. 空值假設：true 區域 = 0.5
