# Supplementary material for: Identification of Temporal Characteristic Networks of Peripheral Blood Changes in Alzheimer’s Disease Based on Weighted Gene Co-expression Network Analysis
Source: Front Aging Neurosci. 2019 May 21;11:83. doi: 10.3389/fnagi.2019.00083 (PMC6537635; doi:10.3389/fnagi.2019.00083)
Supplement: Supplementary file 5 [file Data_Sheet_1.ZIP › Supplementary Materials S1/ROC/ROC GSE63061 BLUE AD-CTL DG BG.pdf]

曲線下的區域

測試結果變數	區域圖	標準錯誤[a]	漸進顯著性[b]	漸進 95% 信賴區間	
				下限	上限
MITD1	.376	.034	.000	.309	.442
DTX2	.555	.035	.119	.486	.623
CEBPZ	.366	.033	.000	.300	.431
GBA	.576	.035	.029	.509	.644
REEP5	.422	.035	.026	.355	.490
PRRC2A	.604	.034	.003	.537	.671
DENR	.366	.034	.000	.300	.431
ACADM	.335	.033	.000	.272	.399
COMMD10	.420	.035	.023	.353	.488
RDH14	.368	.034	.000	.303	.434
CRBN	.391	.034	.002	.324	.458
G6PD	.638	.034	.000	.572	.704
USP16	.392	.034	.002	.326	.459
STAT3	.604	.035	.003	.536	.672
HSPA8	.402	.034	.005	.334	.469

a. 在非參數式假設下

b. 空值假設：true 區域 = 0.5
