# Supplementary material for: Identification of Temporal Characteristic Networks of Peripheral Blood Changes in Alzheimer’s Disease Based on Weighted Gene Co-expression Network Analysis
Source: Front Aging Neurosci. 2019 May 21;11:83. doi: 10.3389/fnagi.2019.00083 (PMC6537635; doi:10.3389/fnagi.2019.00083)
Supplement: Supplementary file 5 [file Data_Sheet_1.ZIP › Supplementary Materials S1/ROC/ROC GSE63061 BLUE AD-MCI DG.pdf]

曲線下的區域

測試結果變數	區域圖	標準錯誤[a]	漸進顯著性[b]	漸進 95% 信賴區間	
				下限	上限
MITD1	.490	.037	.782	.417	.562
DTX2	.515	.037	.684	.442	.588
CEBPZ	.484	.037	.661	.412	.556
GBA	.453	.037	.206	.381	.525
REEP5	.562	.037	.095	.490	.634
PRRC2A	.492	.037	.819	.419	.564
DENR	.512	.037	.752	.439	.584
ACADM	.476	.037	.521	.404	.548
COMMD10	.497	.037	.932	.425	.569
RDH14	.482	.037	.629	.410	.555
CRBN	.504	.037	.918	.431	.576
G6PD	.491	.037	.802	.419	.563
USP16	.487	.037	.730	.415	.559
STAT3	.519	.037	.612	.447	.591
HSPA8	.474	.037	.479	.402	.546

a. 在非參數式假設下

b. 空值假設：true 區域 = 0.5
