# Supplementary material for: Identification of Temporal Characteristic Networks of Peripheral Blood Changes in Alzheimer’s Disease Based on Weighted Gene Co-expression Network Analysis
Source: Front Aging Neurosci. 2019 May 21;11:83. doi: 10.3389/fnagi.2019.00083 (PMC6537635; doi:10.3389/fnagi.2019.00083)
Supplement: Supplementary file 5 [file Data_Sheet_1.ZIP › Supplementary Materials S1/ROC/ROC GSE63061 BLUE MCI-CTL DG BGpdf.pdf]

曲線下的區域

測試結果變數	區域圖	標準錯誤[a]	漸進顯著性[b]	漸進 95% 信賴區間	
				下限	上限
MITD1	.375	.036	.001	.305	.445
DTX2	.536	.037	.338	.462	.609
CEBPZ	.372	.036	.001	.302	.442
GBA	.633	.036	.000	.562	.703
REEP5	.365	.036	.000	.295	.435
PRRC2A	.613	.036	.002	.542	.684
DENR	.352	.035	.000	.283	.421
ACADM	.343	.035	.000	.274	.411
COMMD10	.419	.037	.030	.347	.491
RDH14	.381	.036	.001	.311	.452
CRBN	.385	.036	.002	.314	.456
G6PD	.670	.035	.000	.603	.738
USP16	.404	.036	.010	.332	.475
STAT3	.614	.037	.002	.542	.686
HSPA8	.413	.037	.020	.341	.485

a. 在非參數式假設下

b. 空值假設：true 區域 = 0.5
