# Supplementary material for: Identification of Temporal Characteristic Networks of Peripheral Blood Changes in Alzheimer’s Disease Based on Weighted Gene Co-expression Network Analysis
Source: Front Aging Neurosci. 2019 May 21;11:83. doi: 10.3389/fnagi.2019.00083 (PMC6537635; doi:10.3389/fnagi.2019.00083)
Supplement: Supplementary file 5 [file Data_Sheet_1.ZIP › Supplementary Materials S1/ROC/ROC GSE63061 BROWN AD-CTL DG BG.pdf]

曲線下的區域

測試結果變數	區域圖	標準錯誤[a]	漸進顯著性[b]	漸進 95% 信賴區間	
				下限	上限
MRPL22	.349	.033	.000	.285	.413
TOMM7	.295	.031	.000	.234	.356
DPM1	.355	.033	.000	.290	.420
RPL26L1	.402	.034	.005	.335	.469
NDUFB3	.365	.033	.000	.300	.430
RPS3A	.353	.033	.000	.288	.417
TMEM126B	.398	.034	.004	.331	.465
PSMA6	.373	.033	.000	.308	.439
RPS27	.332	.032	.000	.269	.396
PSMA4	.355	.033	.000	.290	.420
RPS17	.331	.032	.000	.267	.394
LSM3	.357	.033	.000	.292	.422
ATP5J	.296	.031	.000	.235	.356
RPL17	.334	.032	.000	.271	.398
LARP7	.353	.033	.000	.289	.418

a. 在非參數式假設下

b. 空值假設：true 區域 = 0.5
