# Supplementary material for: Identification of Temporal Characteristic Networks of Peripheral Blood Changes in Alzheimer’s Disease Based on Weighted Gene Co-expression Network Analysis
Source: Front Aging Neurosci. 2019 May 21;11:83. doi: 10.3389/fnagi.2019.00083 (PMC6537635; doi:10.3389/fnagi.2019.00083)
Supplement: Supplementary file 5 [file Data_Sheet_1.ZIP › Supplementary Materials S1/ROC/ROC GSE63061 BROWN AD-MCI DG BG.pdf]

曲線下的區域

測試結果變數	區域圖	標準錯誤[a]	漸進顯著性[b]	漸進 95% 信賴區間	
				下限	上限
MRPL22	.486	.037	.707	.414	.558
TOMM7	.434	.037	.073	.362	.505
DPM1	.486	.037	.714	.414	.559
RPL26L1	.473	.037	.462	.400	.545
NDUFB3	.483	.037	.647	.410	.556
RPS3A	.446	.037	.145	.374	.518
TMEM126B	.517	.037	.651	.444	.589
PSMA6	.472	.037	.451	.399	.545
RPS27	.472	.037	.453	.400	.544
PSMA4	.446	.037	.146	.374	.519
RPS17	.469	.037	.406	.397	.541
LSM3	.449	.037	.168	.377	.521
ATP5J	.443	.037	.124	.371	.515
RPL17	.462	.037	.298	.389	.534
LARP7	.471	.037	.440	.399	.544

測試結果變數: RPL17 在正數實際狀態與負數實際狀態群組之間至少有一個連結空間。統計資料可能有偏差。

a. 在非參數式假設下

b. 空值假設：true 區域 = 0.5
