# Supplementary material for: Identification of Temporal Characteristic Networks of Peripheral Blood Changes in Alzheimer’s Disease Based on Weighted Gene Co-expression Network Analysis
Source: Front Aging Neurosci. 2019 May 21;11:83. doi: 10.3389/fnagi.2019.00083 (PMC6537635; doi:10.3389/fnagi.2019.00083)
Supplement: Supplementary file 5 [file Data_Sheet_1.ZIP › Supplementary Materials S1/ROC/ROC GSE63061 BROWN MCI-CTL DG BG.pdf]

曲線下的區域

測試結果變數	區域圖	標準錯誤[a]	漸進顯著性[b]	漸進 95% 信賴區間	
				下限	上限
MRPL22	.358	.035	.000	.289	.427
TOMM7	.357	.035	.000	.288	.426
DPM1	.366	.035	.000	.297	.436
RPL26L1	.427	.036	.050	.355	.498
NDUFB3	.383	.036	.002	.313	.453
RPS3A	.400	.036	.007	.328	.471
TMEM126B	.383	.036	.002	.312	.453
PSMA6	.402	.036	.008	.331	.473
RPS27	.359	.035	.000	.290	.427
PSMA4	.406	.036	.012	.335	.478
RPS17	.353	.035	.000	.284	.421
LSM3	.399	.036	.007	.328	.469
ATP5J	.346	.035	.000	.278	.414
RPL17	.365	.035	.000	.295	.434
LARP7	.382	.036	.002	.312	.452

測試結果變數: RPS27 在正數實際狀態與負數實際狀態群組之間至少有一個連結空間。統計資料可能有偏差。

a. 在非參數式假設下

b. 空值假設：true 區域 = 0.5
