# Supplementary material for: Identification of Temporal Characteristic Networks of Peripheral Blood Changes in Alzheimer’s Disease Based on Weighted Gene Co-expression Network Analysis
Source: Front Aging Neurosci. 2019 May 21;11:83. doi: 10.3389/fnagi.2019.00083 (PMC6537635; doi:10.3389/fnagi.2019.00083)
Supplement: Supplementary file 5 [file Data_Sheet_1.ZIP › Supplementary Materials S1/ROC/ROC GSE63061 PINK AD-CTL DG BG.pdf]

曲線下的區域

測試結果變數	區域圖	標準錯誤[a]	漸進顯著性[b]	漸進 95% 信賴區間	
				下限	上限
MANSC1	.547	.035	.184	.478	.615
PFKFB4	.649	.033	.000	.584	.715
REPS2	.607	.034	.002	.540	.675
LAMP2	.615	.034	.001	.548	.681
RNF149	.599	.034	.005	.532	.666
SVIL	.599	.034	.005	.532	.666
ZNF746	.577	.035	.029	.508	.645
FCGR2A	.528	.035	.429	.459	.597
MSRB1	.597	.035	.005	.530	.665
DENND5A	.602	.034	.003	.535	.670
P6V1B2	.582	.034	.019	.514	.650
MXD1	.624	.034	.000	.558	.691
SIRPA	.613	.034	.001	.546	.679
FPR2	.567	.035	.055	.499	.635
NDEL1	.572	.035	.039	.504	.641

a. 在非參數式假設下

b. 空值假設：true 區域 = 0.5
