# Supplementary material for: Identification of Temporal Characteristic Networks of Peripheral Blood Changes in Alzheimer’s Disease Based on Weighted Gene Co-expression Network Analysis
Source: Front Aging Neurosci. 2019 May 21;11:83. doi: 10.3389/fnagi.2019.00083 (PMC6537635; doi:10.3389/fnagi.2019.00083)
Supplement: Supplementary file 5 [file Data_Sheet_1.ZIP › Supplementary Materials S1/ROC/ROC GSE63061 PINK AD-MCI DG BG.pdf]

曲線下的區域

測試結果變數	區域圖	標準錯誤[a]	漸進顯著性[b]	漸進 95% 信賴區間	
				下限	上限
MANSC1	.539	.037	.294	.467	.611
PFKFB4	.570	.036	.060	.498	.641
REPS2	.543	.036	.243	.472	.615
LAMP2	.555	.036	.137	.484	.627
RNF149	.571	.036	.055	.500	.643
SVIL	.557	.037	.123	.485	.629
ZNF746	.540	.036	.284	.468	.611
FCGR2A	.508	.037	.837	.436	.579
MSRB1	.577	.036	.037	.506	.648
DENND5A	.565	.036	.077	.494	.636
P6V1B2	.526	.037	.477	.455	.598
MXD1	.586	.036	.020	.515	.657
SIRPA	.539	.037	.293	.467	.611
FPR2	.518	.037	.623	.446	.590
NDEL1	.524	.037	.509	.453	.596

a. 在非參數式假設下

b. 空值假設：true 區域 = 0.5
