# Supplementary material for: Identification of Temporal Characteristic Networks of Peripheral Blood Changes in Alzheimer’s Disease Based on Weighted Gene Co-expression Network Analysis
Source: Front Aging Neurosci. 2019 May 21;11:83. doi: 10.3389/fnagi.2019.00083 (PMC6537635; doi:10.3389/fnagi.2019.00083)
Supplement: Supplementary file 5 [file Data_Sheet_1.ZIP › Supplementary Materials S1/ROC/ROC GSE63061 PINK MCI-CTL DG BG.pdf .pdf]

曲線下的區域

測試結果變數	區域圖	標準錯誤[a]	漸進顯著性[b]	漸進 95% 信賴區間	
				下限	上限
MANSC1	.510	.037	.780	.437	.583
PFKFB4	.616	.036	.002	.546	.686
REPS2	.590	.037	.015	.519	.662
LAMP2	.564	.037	.085	.492	.637
RNF149	.531	.037	.413	.457	.604
SVIL	.550	.037	.179	.478	.623
ZNF746	.544	.037	.240	.471	.616
FCGR2A	.528	.037	.461	.454	.601
MSRB1	.538	.037	.313	.465	.610
DENND5A	.548	.037	.202	.474	.621
P6V1B2	.561	.037	.101	.489	.633
MXD1	.553	.037	.158	.480	.625
SIRPA	.590	.036	.016	.518	.661
FPR2	.555	.037	.139	.483	.627
NDEL1	.561	.037	.101	.488	.634

a. 在非參數式假設下

b. 空值假設：true 區域 = 0.5
