# Supplementary material for: Identification of Temporal Characteristic Networks of Peripheral Blood Changes in Alzheimer’s Disease Based on Weighted Gene Co-expression Network Analysis
Source: Front Aging Neurosci. 2019 May 21;11:83. doi: 10.3389/fnagi.2019.00083 (PMC6537635; doi:10.3389/fnagi.2019.00083)
Supplement: Supplementary file 5 [file Data_Sheet_1.ZIP › Supplementary Materials S1/ROC/ROC GSE63061 RED AD-CTL DG BG.pdf]

曲線下的區域

測試結果變數	區域圖	標準錯誤[a]	漸進顯著性[b]	漸進 95% 信賴區間	
				下限	上限
CRBN	.392	.034	.002	.325	.459
CAMLG	.431	.035	.049	.363	.499
CLNS1A	.364	.034	.000	.298	.430
RALA	.400	.034	.004	.333	.468
GPN1	.351	.033	.000	.286	.417
AK3	.408	.034	.008	.341	.475
MTERF3	.418	.035	.020	.351	.486
CCDC25	.385	.034	.001	.318	.452
EBAG9	.350	.033	.000	.285	.415
PDCD2	.419	.035	.020	.350	.487
PPP3CB	.426	.035	.034	.358	.494
NDUFB5	.423	.035	.029	.356	.491
SNRPF	.387	.034	.001	.320	.454
DDX1	.359	.033	.000	.294	.425

a. 在非參數式假設下

b. 空值假設：true 區域 = 0.5
