# Supplementary material for: Identification of Temporal Characteristic Networks of Peripheral Blood Changes in Alzheimer’s Disease Based on Weighted Gene Co-expression Network Analysis
Source: Front Aging Neurosci. 2019 May 21;11:83. doi: 10.3389/fnagi.2019.00083 (PMC6537635; doi:10.3389/fnagi.2019.00083)
Supplement: Supplementary file 5 [file Data_Sheet_1.ZIP › Supplementary Materials S1/ROC/ROC GSE63061 RED AD-MCI DG BG.pdf]

曲線下的區域

測試結果變數	區域圖	標準錯誤[a]	漸進顯著性[b]	漸進 95% 信賴區間	
				下限	上限
CRBN	.480	.037	.587	.408	.552
CAMLG	.546	.037	.212	.475	.618
CLNS1A	.506	.037	.862	.434	.578
RALA	.525	.037	.499	.453	.597
GPN1	.480	.037	.582	.408	.551
AK3	.540	.037	.285	.468	.611
MTERF3	.520	.037	.594	.448	.592
CCDC25	.510	.037	.786	.438	.582
EBAG9	.510	.037	.790	.438	.582
PDCD2	.568	.036	.066	.497	.639
PPP3CB	.551	.036	.167	.480	.623
NDUFB5	.569	.037	.062	.497	.641
SNRPF	.536	.037	.331	.464	.608
DDX1	.464	.037	.337	.392	.537

a. 在非參數式假設下

b. 空值假設：true 區域 = 0.5
