# Supplementary material for: Identification of Temporal Characteristic Networks of Peripheral Blood Changes in Alzheimer’s Disease Based on Weighted Gene Co-expression Network Analysis
Source: Front Aging Neurosci. 2019 May 21;11:83. doi: 10.3389/fnagi.2019.00083 (PMC6537635; doi:10.3389/fnagi.2019.00083)
Supplement: Supplementary file 5 [file Data_Sheet_1.ZIP › Supplementary Materials S1/ROC/ROC GSE63061 RED MCI-CTL DG BG.pdf]

曲線下的區域

測試結果變數	區域圖	標準錯誤[a]	漸進顯著性[b]	漸進 95% 信賴區間	
				下限	上限
CRBN	.398	.036	.006	.327	.469
CAMLG	.372	.036	.001	.301	.443
CLNS1A	.338	.035	.000	.269	.407
RALA	.377	.036	.001	.306	.448
GPN1	.352	.035	.000	.283	.422
AK3	.372	.036	.001	.302	.441
MTERF3	.389	.036	.003	.318	.461
CCDC25	.363	.036	.000	.293	.433
EBAG9	.324	.034	.000	.257	.391
PDCD2	.331	.035	.000	.263	.400
PPP3CB	.361	.036	.000	.291	.431
NDUFB5	.359	.036	.000	.289	.429
SNRPF	.332	.034	.000	.265	.400
DDX1	.373	.036	.001	.303	.443

a. 在非參數式假設下

b. 空值假設：true 區域 = 0.5
