# Supplementary material for: Identification of Temporal Characteristic Networks of Peripheral Blood Changes in Alzheimer’s Disease Based on Weighted Gene Co-expression Network Analysis
Source: Front Aging Neurosci. 2019 May 21;11:83. doi: 10.3389/fnagi.2019.00083 (PMC6537635; doi:10.3389/fnagi.2019.00083)
Supplement: Supplementary file 5 [file Data_Sheet_1.ZIP › Supplementary Materials S1/ROC/ROC GSE63061 TURQUIOES AD-CTLDG BG.pdf]

曲線下的區域

測試結果變數	區域圖	標準錯誤[a]	漸進顯著性[b]	漸進 95% 信賴區間	
				下限	上限
ACTR3	.458	.035	.230	.390	.526
GIMAP2	.393	.034	.002	.327	.460
ANKRD10	.490	.035	.768	.421	.558
LUC7L3	.466	.035	.334	.397	.535
SACM1L	.467	.035	.351	.399	.536
ADD3	.486	.035	.681	.417	.554
PHIP	.456	.035	.212	.387	.525
CMPK1	.408	.034	.008	.340	.475
FAM49B	.476	.035	.486	.407	.544
MTPN	.511	.035	.743	.443	.580
UBLCP	.433	.035	.055	.365	.501
STK26	.452	.035	.168	.383	.520
WIPF1	.571	.035	.042	.504	.639
ATF4	.479	.035	.548	.410	.548

a. 在非參數式假設下

b. 空值假設：true 區域 = 0.5
