# Supplementary material for: Identification of Temporal Characteristic Networks of Peripheral Blood Changes in Alzheimer’s Disease Based on Weighted Gene Co-expression Network Analysis
Source: Front Aging Neurosci. 2019 May 21;11:83. doi: 10.3389/fnagi.2019.00083 (PMC6537635; doi:10.3389/fnagi.2019.00083)
Supplement: Supplementary file 5 [file Data_Sheet_1.ZIP › Supplementary Materials S1/ROC/ROC GSE63061 TURQUIOES AD-MCI DG BG.pdf]

曲線下的區域

測試結果變數	區域圖	標準錯誤[a]	漸進顯著性[b]	漸進 95% 信賴區間	
				下限	上限
ACTR3	.538	.037	.303	.466	.611
GIMAP2	.464	.037	.334	.392	.536
ANKRD10	.513	.037	.727	.441	.585
LUC7L3	.548	.037	.192	.477	.620
SACM1L	.530	.037	.422	.458	.602
ADD3	.506	.037	.880	.434	.578
PHIP	.513	.037	.725	.441	.585
CMPK1	.498	.037	.954	.425	.570
FAM49B	.514	.037	.695	.442	.587
MTPN	.526	.037	.484	.453	.599
UBLCP	.536	.037	.325	.464	.608
STK26	.524	.037	.524	.452	.596
WIPF1	.523	.037	.539	.451	.595
ATF4	.487	.038	.731	.414	.561

a. 在非參數式假設下

b. 空值假設：true 區域 = 0.5
