# Supplementary material for: Identification of Temporal Characteristic Networks of Peripheral Blood Changes in Alzheimer’s Disease Based on Weighted Gene Co-expression Network Analysis
Source: Front Aging Neurosci. 2019 May 21;11:83. doi: 10.3389/fnagi.2019.00083 (PMC6537635; doi:10.3389/fnagi.2019.00083)
Supplement: Supplementary file 5 [file Data_Sheet_1.ZIP › Supplementary Materials S1/ROC/ROC GSE63061 TURQUIOES MCI-CTL DG BG.pdf]

曲線下的區域

測試結果變數	區域圖	標準錯誤[a]	漸進顯著性[b]	漸進 95% 信賴區間	
				下限	上限
ACTR3	.421	.037	.035	.349	.493
GIMAP2	.424	.037	.043	.352	.497
ANKRD10	.480	.037	.592	.407	.553
LUC7L3	.406	.037	.011	.334	.477
SACM1L	.433	.037	.072	.361	.505
ADD3	.478	.037	.561	.405	.551
PHIP	.436	.037	.086	.363	.509
CMPK1	.401	.036	.008	.329	.472
FAM49B	.458	.037	.258	.385	.531
MTPN	.485	.037	.692	.412	.558
UBLCP	.395	.036	.005	.324	.466
STK26	.420	.037	.032	.348	.492
WIPF1	.550	.037	.177	.478	.623
ATF4	.492	.037	.836	.419	.565

a. 在非參數式假設下

b. 空值假設：true 區域 = 0.5
