# Supplementary material for: Identification of Temporal Characteristic Networks of Peripheral Blood Changes in Alzheimer’s Disease Based on Weighted Gene Co-expression Network Analysis
Source: Front Aging Neurosci. 2019 May 21;11:83. doi: 10.3389/fnagi.2019.00083 (PMC6537635; doi:10.3389/fnagi.2019.00083)
Supplement: Supplementary file 5 [file Data_Sheet_1.ZIP › Supplementary Materials S1/ROC/ROC GSE63061 YELLOW AD-CTL DG BG.pdf]

曲線下的區域

測試結果變數	區域圖	標準錯誤[a]	漸進顯著性[b]	漸進 95% 信賴區間	
				下限	上限
THAP12	.405	.035	.007	.337	.473
ANKRD49	.396	.034	.003	.329	.463
RPS6KB1	.430	.035	.045	.362	.498
PPM1B	.420	.035	.022	.352	.487
PPP2CA	.505	.035	.888	.436	.574
CD58	.403	.034	.006	.336	.471
CNIH1	.511	.035	.744	.443	.580
ARGLU1	.443	.035	.106	.375	.512
SNRK	.425	.035	.032	.357	.493
UPF2	.434	.035	.060	.366	.502
PCNX4	.469	.035	.379	.401	.538
ST8SIA4	.433	.035	.057	.365	.501
TRIM33	.494	.035	.865	.425	.563
BCLAF1	.450	.035	.152	.381	.518

a. 在非參數式假設下

b. 空值假設：true 區域 = 0.5
