# Supplementary material for: Identification of Temporal Characteristic Networks of Peripheral Blood Changes in Alzheimer’s Disease Based on Weighted Gene Co-expression Network Analysis
Source: Front Aging Neurosci. 2019 May 21;11:83. doi: 10.3389/fnagi.2019.00083 (PMC6537635; doi:10.3389/fnagi.2019.00083)
Supplement: Supplementary file 5 [file Data_Sheet_1.ZIP › Supplementary Materials S1/ROC/ROC GSE63061 YELLOW AD-CTL LH BG.pdf]

曲線下的區域

測試結果變數:　預測機率

| 區域圖 | 標準錯誤[a] | 漸進顯著性[b] | 漸進 95% 信賴區間 | |
|---|---|---|---|---|
| | | | 下限 | 上限 |
| .692 | .032 | .000 | .629 | .754 |

a. 在非參數式假設下

b. 空值假設：true 區域 = 0.5
