# Supplementary material for: Identification of Temporal Characteristic Networks of Peripheral Blood Changes in Alzheimer’s Disease Based on Weighted Gene Co-expression Network Analysis
Source: Front Aging Neurosci. 2019 May 21;11:83. doi: 10.3389/fnagi.2019.00083 (PMC6537635; doi:10.3389/fnagi.2019.00083)
Supplement: Supplementary file 5 [file Data_Sheet_1.ZIP › Supplementary Materials S1/ROC/ROC GSE63061 YELLOW AD-MCI DG BG.pdf]

曲線下的區域

測試結果變數	區域圖	標準錯誤[a]	漸進顯著性[b]	漸進 95% 信賴區間	
				下限	上限
THAP12	.529	.037	.439	.456	.601
ANKRD49	.528	.037	.448	.456	.600
RPS6KB1	.534	.037	.351	.462	.607
PPM1B	.557	.037	.121	.485	.630
PPP2CA	.541	.037	.272	.468	.614
CD58	.496	.037	.907	.422	.569
CNIH1	.626	.036	.001	.556	.697
ARGLU1	.558	.037	.120	.486	.629
SNRK	.548	.037	.196	.475	.621
UPF2	.491	.037	.802	.418	.564
PCNX4	.526	.037	.491	.453	.598
ST8SIA4	.515	.037	.685	.442	.588
TRIM33	.551	.037	.166	.480	.623
BCLAF1	.520	.037	.596	.447	.592

a. 在非參數式假設下

b. 空值假設：true 區域 = 0.5
