# Supplementary material for: Identification of Temporal Characteristic Networks of Peripheral Blood Changes in Alzheimer’s Disease Based on Weighted Gene Co-expression Network Analysis
Source: Front Aging Neurosci. 2019 May 21;11:83. doi: 10.3389/fnagi.2019.00083 (PMC6537635; doi:10.3389/fnagi.2019.00083)
Supplement: Supplementary file 5 [file Data_Sheet_1.ZIP › Supplementary Materials S1/ROC/ROC GSE63061 YELLOW MCI-CTL DG BG.pdf]

曲線下的區域

測試結果變數	區域圖	標準錯誤[a]	漸進顯著性[b]	漸進 95% 信賴區間	
				下限	上限
THAP12	.369	.036	.000	.299	.439
ANKRD49	.361	.036	.000	.292	.431
RPS6KB1	.389	.037	.003	.317	.460
PPM1B	.371	.036	.001	.301	.440
PPP2CA	.467	.038	.380	.394	.541
CD58	.412	.036	.018	.340	.483
CNIH1	.382	.037	.002	.311	.454
ARGLU1	.378	.036	.001	.306	.449
SNRK	.388	.036	.003	.317	.458
UPF2	.443	.037	.129	.370	.516
PCNX4	.442	.037	.121	.369	.515
ST8SIA4	.416	.037	.024	.344	.488
TRIM33	.436	.037	.086	.363	.509
BCLAF1	.422	.037	.036	.350	.494

a. 在非參數式假設下

b. 空值假設：true 區域 = 0.5
