# Supplementary figures and images for: Identification of Temporal Characteristic Networks of Peripheral Blood Changes in Alzheimer’s Disease Based on Weighted Gene Co-expression Network Analysis (part 2 of 2)
Source: Front Aging Neurosci. 2019 May 21;11:83. doi: 10.3389/fnagi.2019.00083 (PMC6537635; doi:10.3389/fnagi.2019.00083)

**Module membership vs. gene significance**  
**cor=0.21, p=4.7e-07**

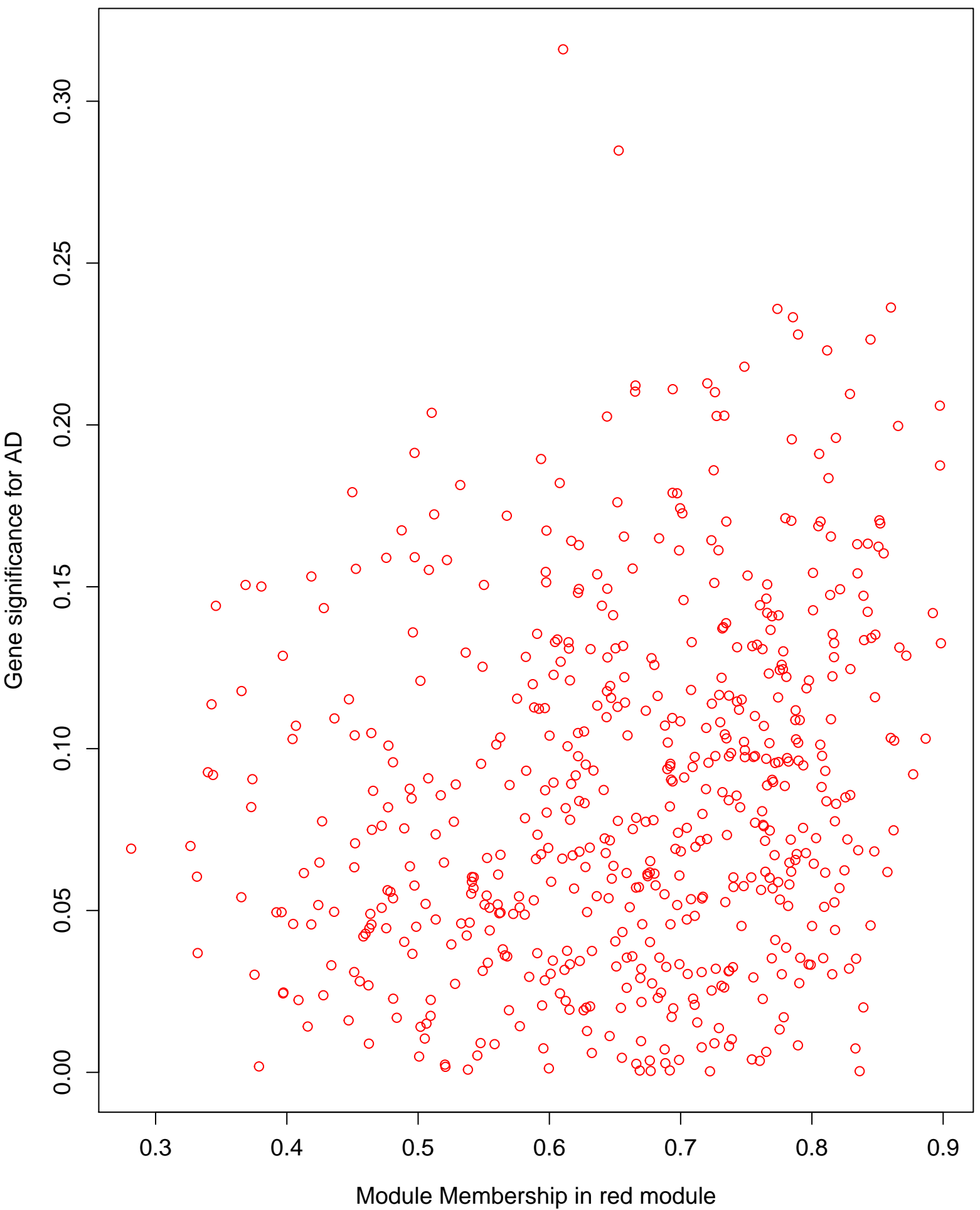

Supplement: Supplementary file 5 [file Data_Sheet_1.ZIP › Supplementary Materials S1/gse63060/red ad.pdf]

**Module membership vs. gene significance**  
**cor=0.19, p=0.016**

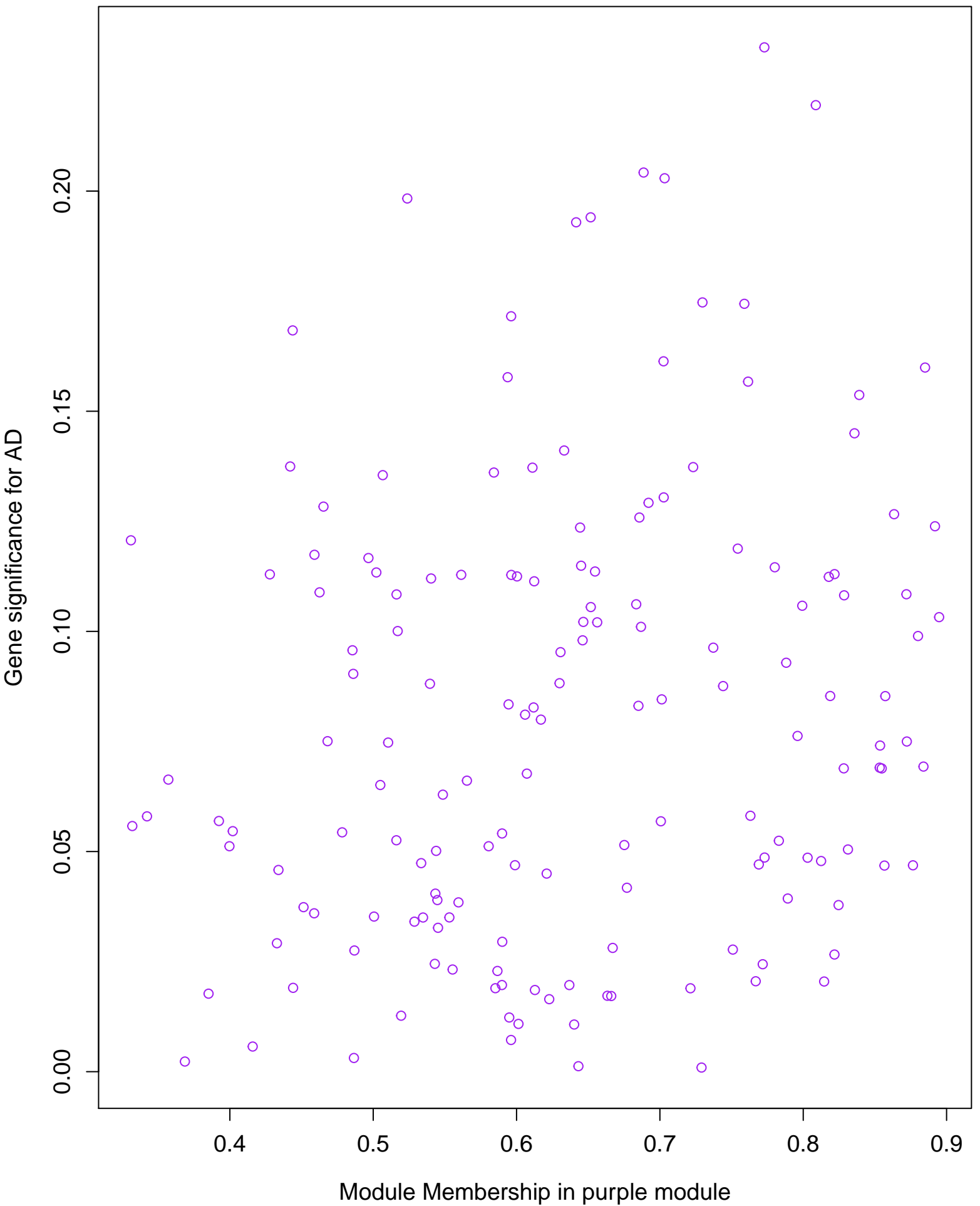

Supplement: Supplementary file 5 [file Data_Sheet_1.ZIP › Supplementary Materials S1/gse63060/purple ad.pdf]

**Module membership vs. gene significance**  
**cor=0.21, p=2.1e-05**

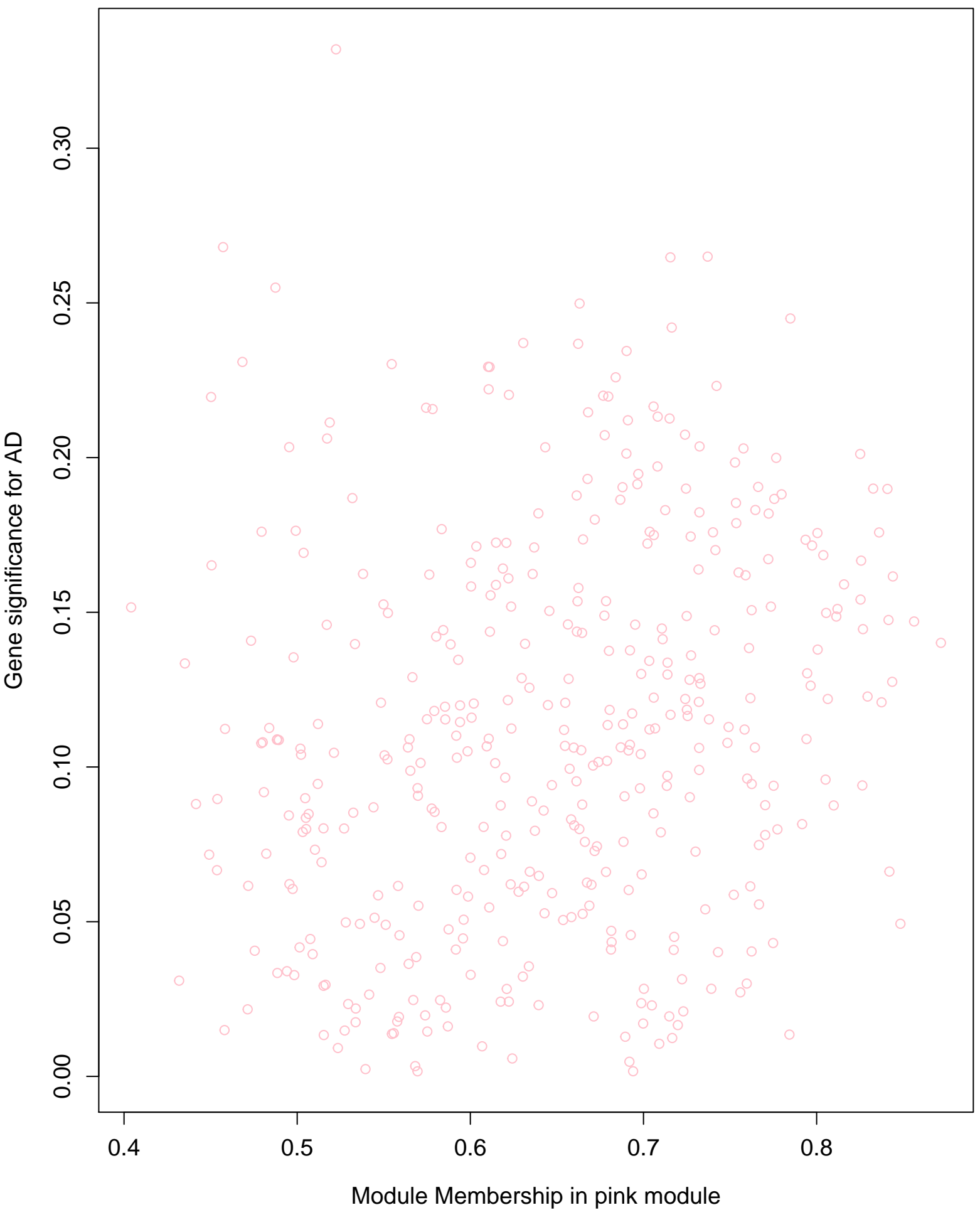

Supplement: Supplementary file 5 [file Data_Sheet_1.ZIP › Supplementary Materials S1/gse63060/pink ad.pdf]

**Module membership vs. gene significance**  
**cor=0.65, p=1.4e-119**

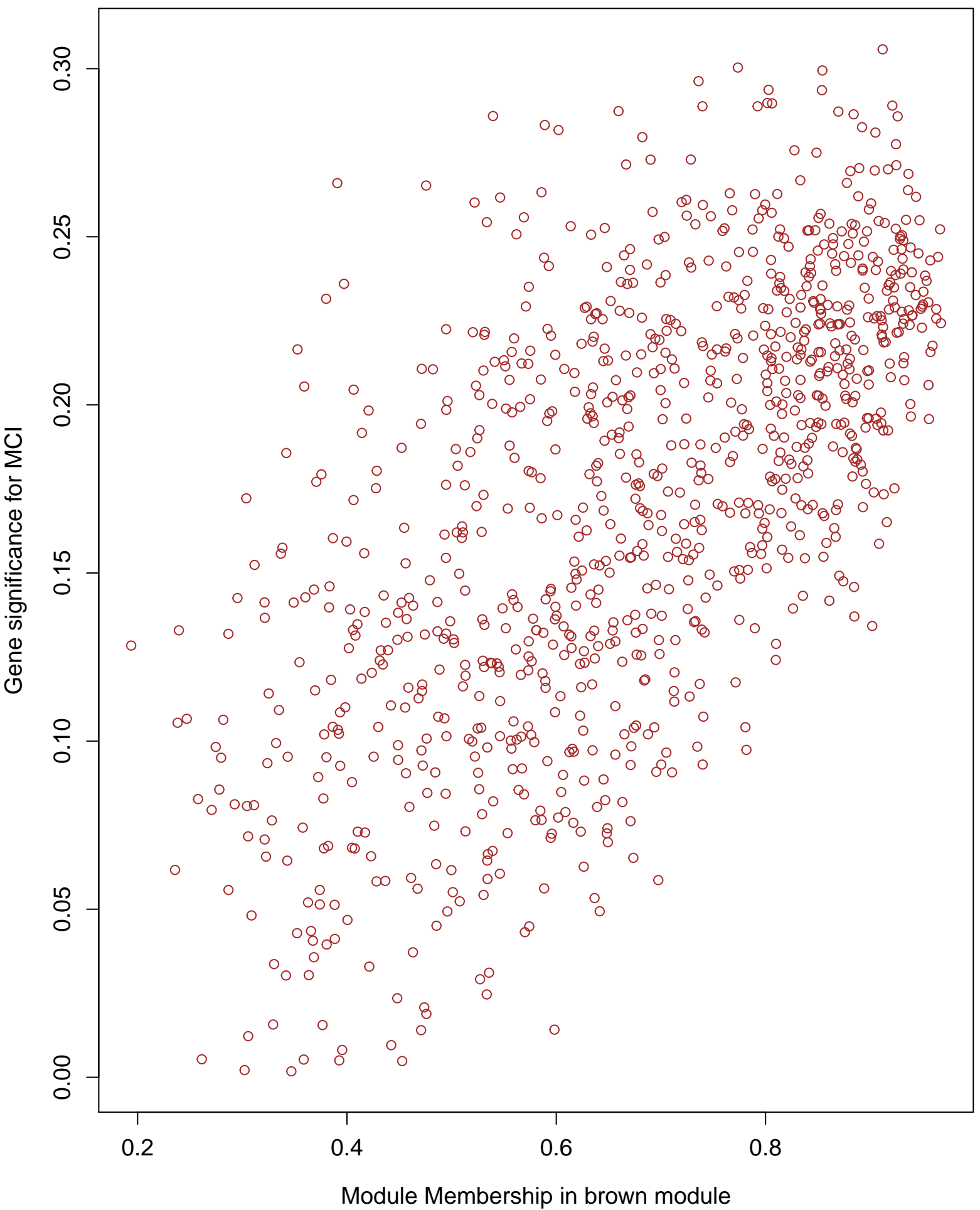

Supplement: Supplementary file 5 [file Data_Sheet_1.ZIP › Supplementary Materials S1/gse63060/brown mci.pdf]

**Module membership vs. gene significance**  
**cor=0.5, p=1.5e-63**

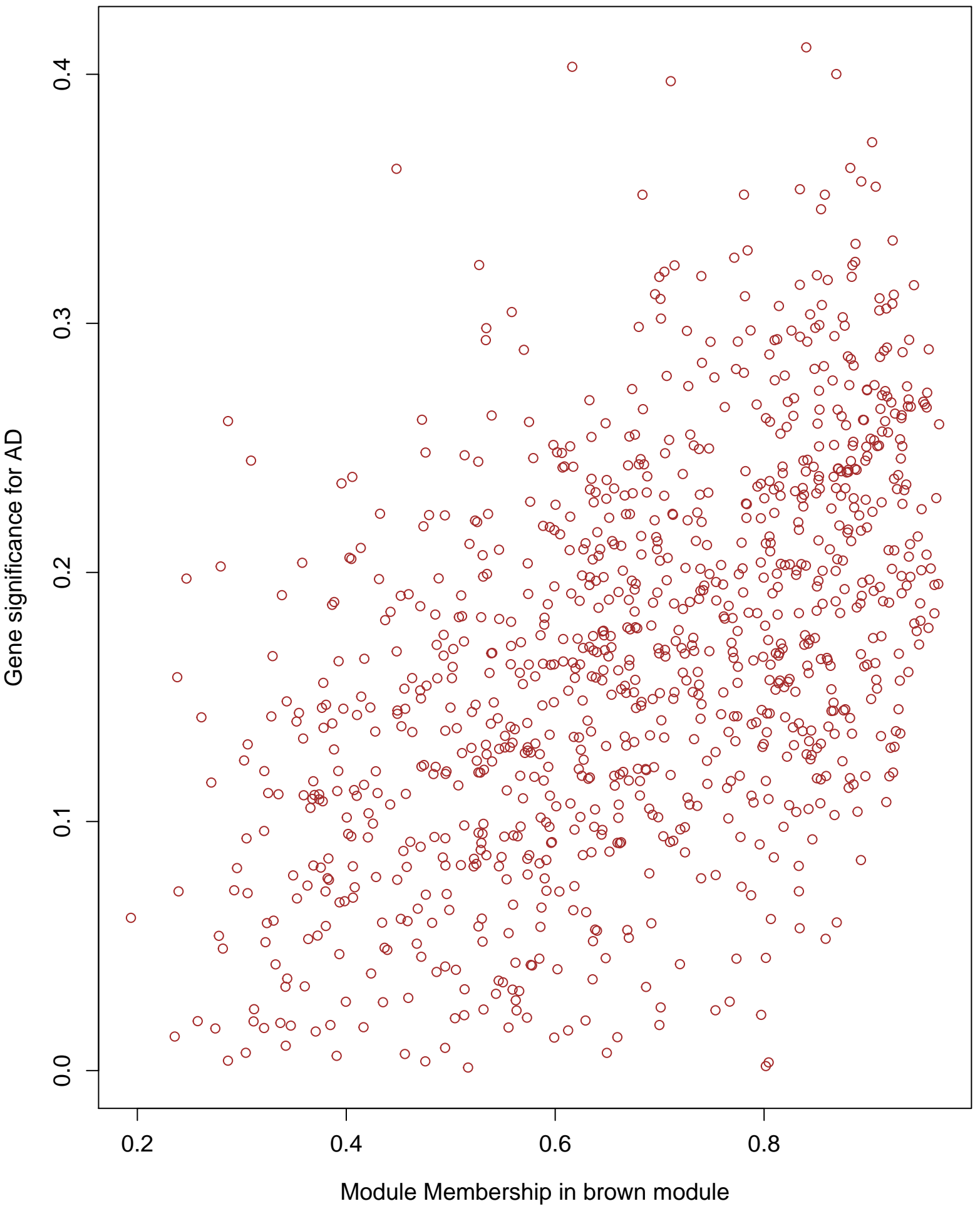

Supplement: Supplementary file 5 [file Data_Sheet_1.ZIP › Supplementary Materials S1/gse63060/brown ad.pdf]

**Module membership vs. gene significance**  
**cor=0.4, p=3e-39**

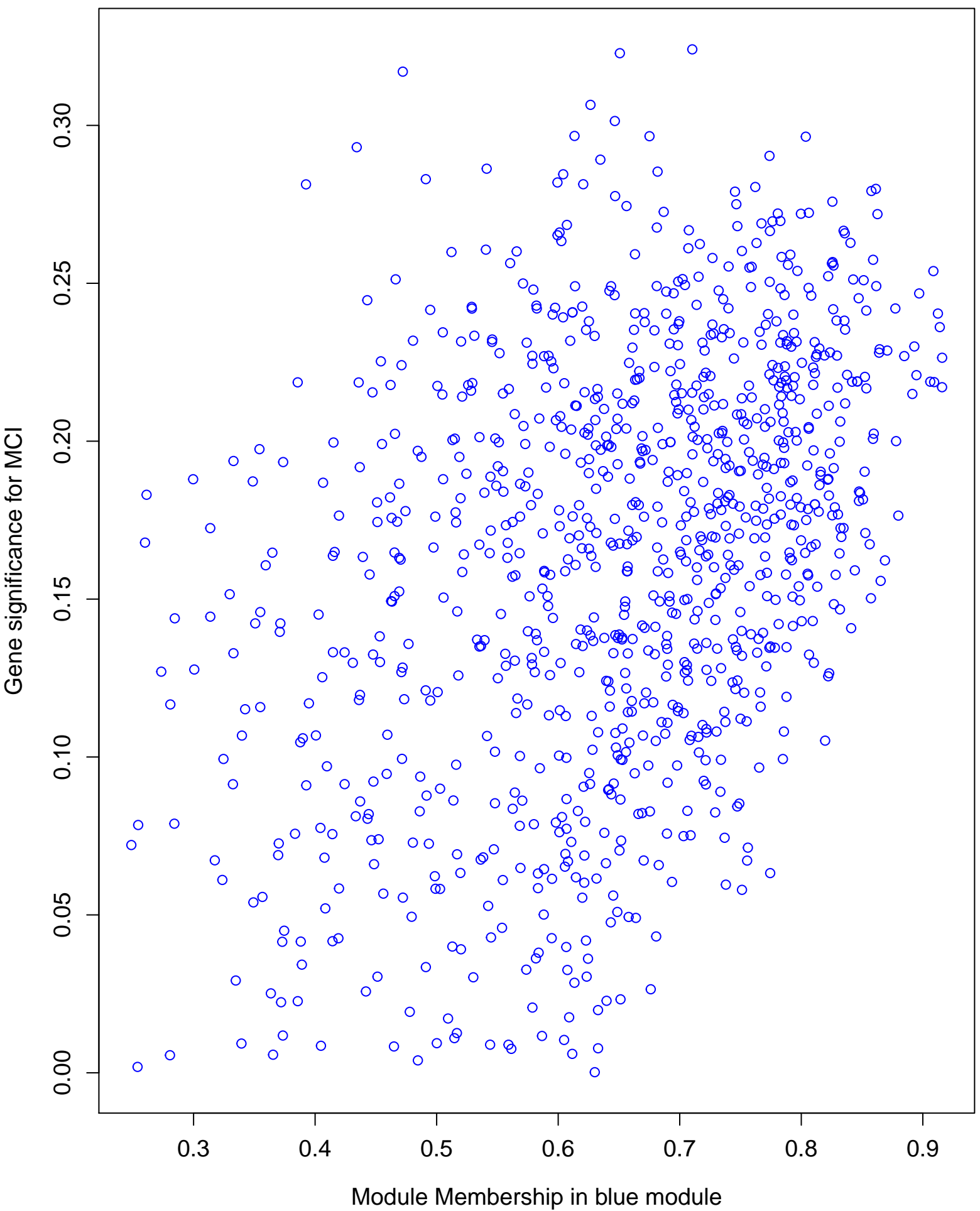

Supplement: Supplementary file 5 [file Data_Sheet_1.ZIP › Supplementary Materials S1/gse63060/blue mci.pdf]

**Module membership vs. gene significance**  
**cor=0.16, p=4.3e-07**

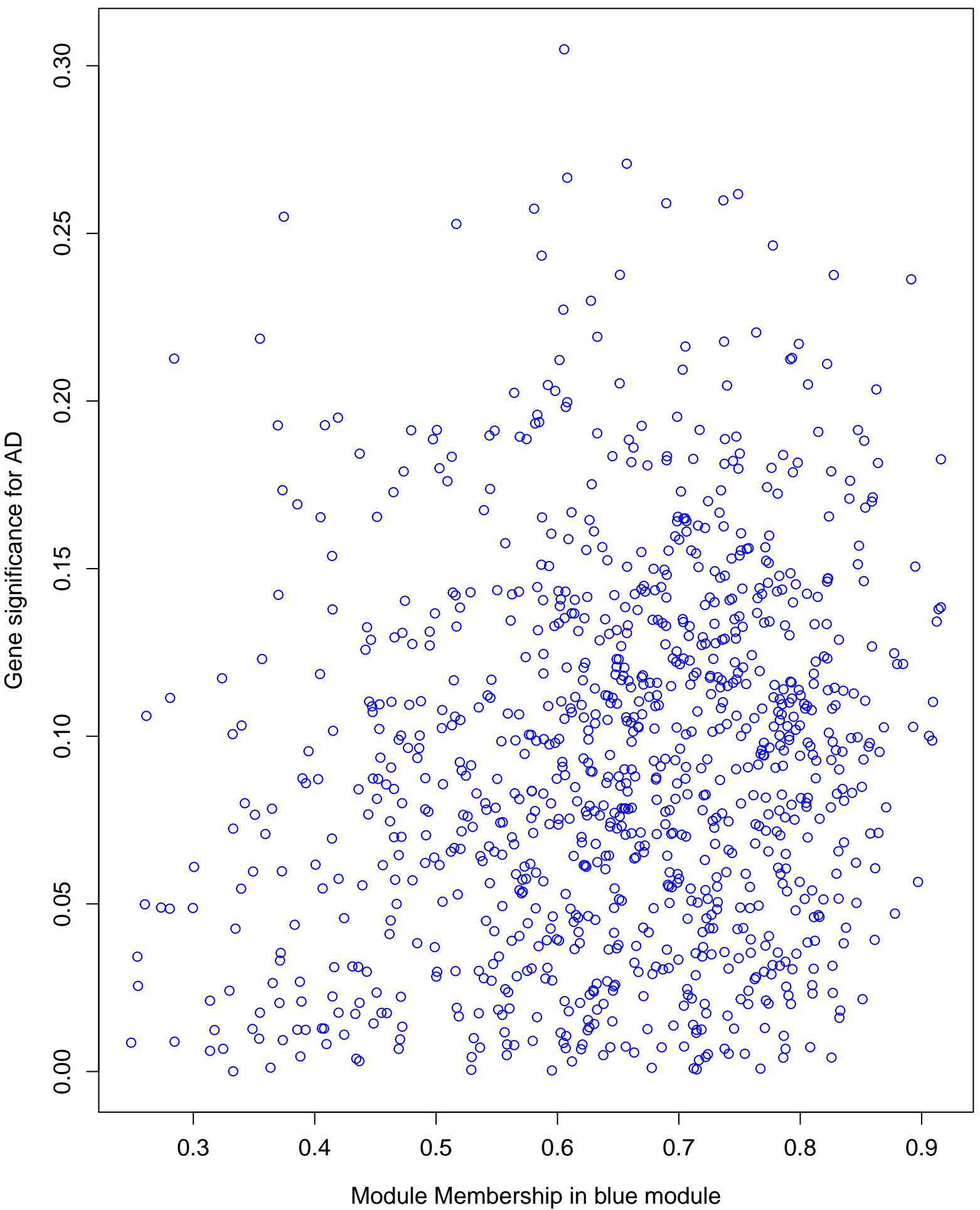

Supplement: Supplementary file 5 [file Data_Sheet_1.ZIP › Supplementary Materials S1/gse63060/blue ad.pdf]

**Module membership vs. gene significance**  
**cor=0.61, p=1.8e-51**

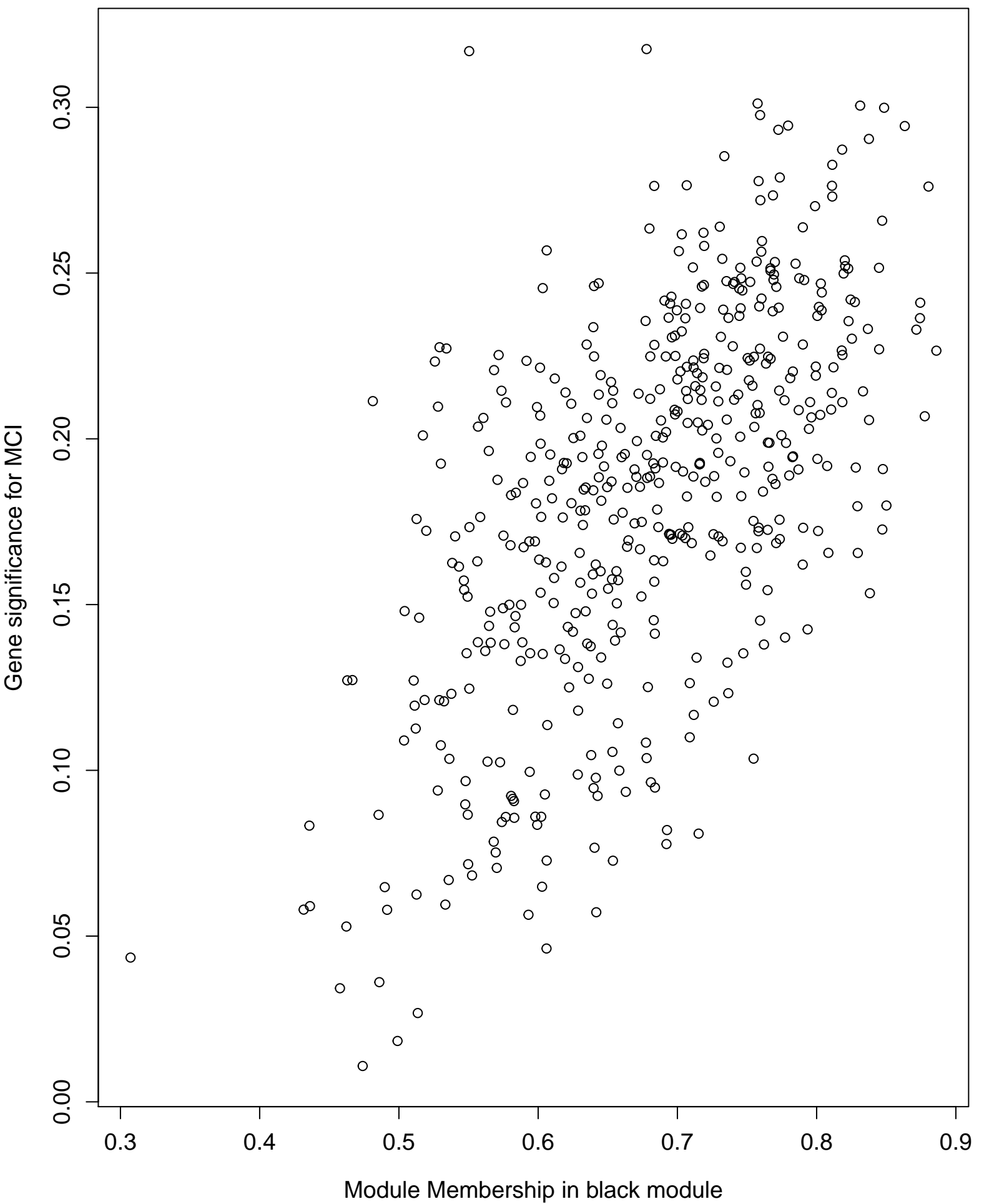

Supplement: Supplementary file 5 [file Data_Sheet_1.ZIP › Supplementary Materials S1/gse63060/black mci.pdf]
